# Pursuing excitonic energy transfer with programmable DNA-based optical breadboards†

**DOI:** 10.1039/d0cs00936a

**Published:** 2023-10-24

**Authors:** Divita Mathur, Sebastián A. Díaz, Niko Hildebrandt, Ryan D. Pensack, Bernard Yurke, Austin Biaggne, Lan Li, Joseph S. Melinger, Mario G. Ancona, William B. Knowlton, Igor L. Medintz

**Affiliations:** a Department of Chemistry, Case Western Reserve University Cleveland OH 44106 USA; b Center for Bio/Molecular Science and Engineering Code 6900 USA igor.medintz@nrl.navy.mil; c Electronics Science and Technology Division, Code 6800, U.S. Naval Research Laboratory Washington DC 20375 USA; d Department of Chemistry, Seoul National University Seoul 08826 South Korea; e Department of Engineering Physics, McMaster University Hamilton L8S 4L7 Canada; f Micron School of Materials Science & Engineering, Boise State University Boise, ID 83725 USA bknowlton@boisestate.edu; g Center for Advanced Energy Studies Idaho Falls ID 83401 USA; h Department of Electrical and Computer Engineering, Florida State University Tallahassee FL 32310 USA

## Abstract

DNA nanotechnology has now enabled the self-assembly of almost any prescribed 3-dimensional nanoscale structure in large numbers and with high fidelity. These structures are also amenable to site-specific modification with a variety of small molecules ranging from drugs to reporter dyes. Beyond obvious application in biotechnology, such DNA structures are being pursued as programmable nanoscale optical breadboards where multiple different/identical fluorophores can be positioned with sub-nanometer resolution in a manner designed to allow them to engage in multistep excitonic energy-transfer (ET) *via* Förster resonance energy transfer (FRET) or other related processes. Not only is the ability to create such complex optical structures unique, more importantly, the ability to rapidly redesign and prototype almost all structural and optical analogues in a massively parallel format allows for deep insight into the underlying photophysical processes. Dynamic DNA structures further provide the unparalleled capability to reconfigure a DNA scaffold on the fly *in situ* and thus switch between ET pathways within a given assembly, actively change its properties, and even repeatedly toggle between two states such as on/off. Here, we review progress in developing these composite materials for potential applications that include artificial light harvesting, smart sensors, nanoactuators, optical barcoding, bioprobes, cryptography, computing, charge conversion, and theranostics to even new forms of optical data storage. Along with an introduction into the DNA scaffolding itself, the diverse fluorophores utilized in these structures, their incorporation chemistry, and the photophysical processes they are designed to exploit, we highlight the evolution of DNA architectures implemented in the pursuit of increased transfer efficiency and the key lessons about ET learned from each iteration. We also focus on recent and growing efforts to exploit DNA as a scaffold for assembling molecular dye aggregates that host delocalized excitons as a test bed for creating excitonic circuits and accessing other quantum-like optical phenomena. We conclude with an outlook on what is still required to transition these materials from a research pursuit to application specific prototypes and beyond.

## Abstract 2

Nanoscale dye-based excitonic systems assembled on DNA origami in solution excited by a laser. Dyes engage in cascaded FRET with exciton movement guided by programmed elements engaging in homo- and hetero-energy transfer.

## Graphical abstract

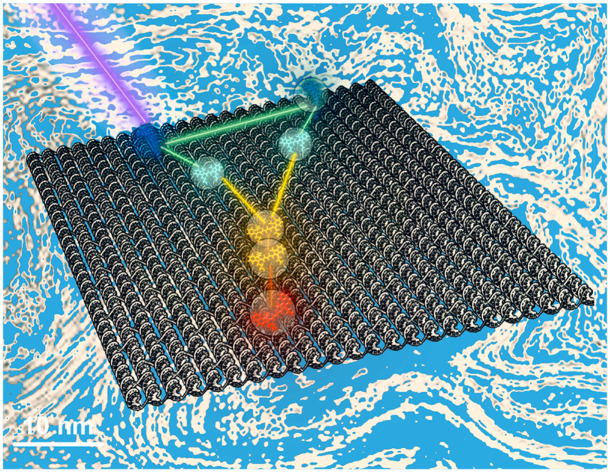

## 1. Introduction

**Fig. 1:**
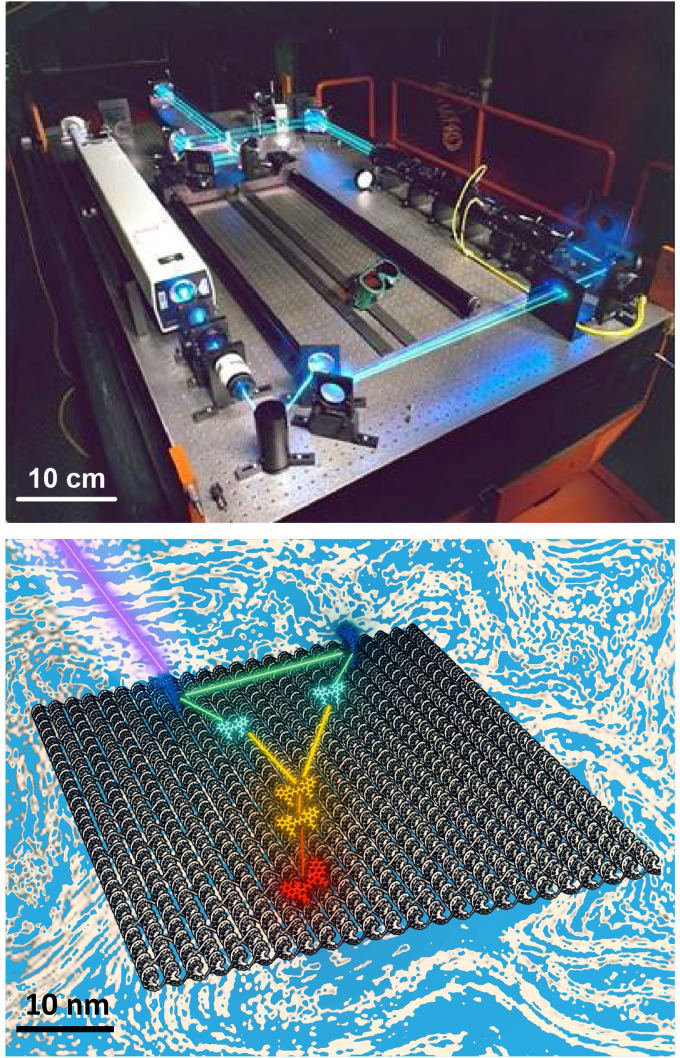
Visual comparison of a ‘macroscale’ optical table hosting a laser setup to a simulated nanoscale dye-based excitonic relay system assembled on a DNA sheet in solution (blue to represent the aqueous environment) and which is being excited by an external laser. For the latter, a series of dyes are placed on the DNA sheet such that they engage in cascaded FRET with the movement of excitons guided by programmed elements that engage in both homo- and hetero energy transfer. For simplicity, dye placement in schematic does not conform to actual FRET requirements. Not all ET pathways including some homoFRET pathways are shown for simplicity. The ability to move and fix active optical elements across the table is mirrored in the addressability of individual DNA strands on the structure aforement. Top laser table image reproduced from https://en.wikipedia.org/wiki/Optical_table#/media/File:Laser.jpg (Tom Trower-NASA/Ames Research Center, in the public domain). Bottom schematic by D. Mathur.

The ability to efficiently harvest, control, and direct light in the nanoscale regime remains a major focus in materials science and especially nanotechnology. Harnessing these capabilities will, in turn, drive development of next generation technologies for data storage, communications, computing, visual display materials, energy generation, (bio)imaging, and help create ‘smart’ stand-alone sensors to name but a few examples from a continuously growing and potentially vast application space.^1^ The ongoing creation of new nanomaterials with unique optical properties such as quantum dots (QDs), plasmonically-active and upconversion nanoparticles (NPs), carbon allotropes, and emissive gold nanoclusters are also contributing to this endeavor.^2–5^ The benchmark ‘technology’ that remains the undisputed model for emulation in the quest to control light movement on the nanoscale is that of how plants and other organisms engage in photosynthesis.^6,7^ Reducing this process to a simplistic description: broad spectrum light is absorbed by chromophores such as chlorophyll embedded in protein scaffolds and then transferred as excitons to reaction centers, where they are converted to chemical energy that can be stored for later use by the organism. Importantly, the transport of excitonic energy within these multistep-linked processes occurs with near unity quantum efficiency and, while still under debate, there is evidence that suggests that nature may exploit coherent quantum processes to help accomplish this.^7–9^ One of the keys to achieving this remarkable quantum efficiency is Nature's ability, developed over eons of evolutionary selection, to precisely organize chromophores into networks with optimal electronic coupling for efficient energy transfer (ET). This typically means that the chromophores are at exactly the right sub-nanometer distance from each other with the right ratios of donor (D)/acceptor (A) molecules and with exquisite control over their orientation(s) relative to each other for optimal excitonic dipole coupling. However, much still remains to be understood about photosynthesis and how its component parts function in an integrated and seamless manner. Moreover, researchers have yet to demonstrate anything remotely close to consistently approaching unity efficiencies in any laboratory-engineered systems that exploit the same ET physics. What would clearly help accelerate our ability to understand and harness these processes is an experimental format that allows researchers to test and recapitulate the key ET steps with an ability to rapidly and easily rearrange in a modular and arbitrary manner the optically-active components and the scaffolding they are displayed on as desired. In essence, what is suggested is a nanoscale optical breadboard with all the same functionality offered by a conventional macroscale optical table but with the capability to rapidly switch between experimental configurations at the molecular level in a similar plug-and-play format (see Fig. 1).

To understand what is being specifically asked for here it is instructive to examine what such an idealized nanoscale breadboard will need to provide. Using organic dye molecules as an example of an optically-active component, the most critical need is that the spacing between each dye must be intimately controlled with sub-nanometer resolution since any changes in this separation range can result in dramatic changes to ET and optical output. The main goal is to be able to position the dyes close enough to each other to enable strong dipolar coupling yet still far enough away to prevent overlap of electron orbitals unless that is actually desired. Likewise, one must have control over the orientation and rotational flexibility of the dyes. The scaffolding material itself must provide for almost any arbitrary 1-, 2-, or 3-dimensional architecture to be quickly assembled and, moreover, be easily redesigned and reconfigured dynamically *in situ* to change dye choice and dye placement along with even altering the host structures' underlying configuration. Perhaps equally important, fabrication should be efficient, reproducible, scalable, facile to implement, and affordable. It would also be extremely useful to expand beyond just organic dyes and access the growing library of photoactive nanomaterials currently available (*e.g.* QDs and other nanoscale ‘dot’ materials, NPs, metal chelates, noble metal nanoclusters, *etc.*) with the same aforementioned capabilities maintained. Although top-down fabrication, layer-by-layer (LBL) assembly, photoactive dendrimer chemistry, and even (fluorescent) protein scaffolding approaches have provided access to some of these properties, none has yet been able to deliver even a plurality of the aforementioned attributes.^10,11^ For all intents and purposes, this roadblock essentially remained in place until the advent of DNA nanotechnology.

As pioneered by Seeman in the 1980s, and after undergoing a continuous and concerted maturation process, DNA nanotechnology now defines a growing area of materials science that harnesses the structural properties of DNA (and other nucleic acids such as RNA) to self-assemble static and dynamic molecular scale structures and devices.^12,13^ At its heart, assembly of these structures is driven by the canonical Watson–Crick–Franklin base-pairing between the nucleotidyl nitrogenous bases, where cytosine (C) pairs with guanine (G) by 3 hydrogen bonds and the adenine (A) pairs to thymine (T) by 2 hydrogen bonds. By designing specific complementary DNA sequences to pair up with each other by hybridization and using careful stoichiometry to drive the assembly reaction forward, formation of double strand (ds) DNA, junctions, crosslinks, crossovers, and many other designer motifs cumulatively come together in an orchestrated manner to give rise to designer hierarchical nanoscale structures of increasing complexity. These can range from relatively simple planar structures such as 2-dimensional rectangles to active 3-dimensional assemblies such as boxes with hinged lids that open and close on cue when driven by addition of exogenous DNA strands that initiate a series of specifically targeted strand-displacements.^14^ These structures are typically classed by the characteristic assembly motif that is utilized including, for example, DNA origami, DNA bricks, tiles, *etc.*, and are designed on mostly open source software (*e.g.*, caDNAno).^12,15–18^ More pertinently, given their ability to assume almost any conceivable nanoscale shape in conjunction with their many other unique physicochemical properties of interest (*vide infra*), it has now become evident that, although more work is still needed, designer DNA structures can now function to a great extent as the nanoscale optical breadboards described above. Fig. 1 is meant to visually depict this concept and compares a ‘macroscale’ optical table hosting a laser setup with a nanoscale dye-based excitonic or FRET relay system assembled on a DNA origami sheet in solution where a D dye is excited by an external laser and then transfers energy to downstream A dyes by both homo- and heteroFRET. The size difference between the two structures approaches eight orders of magnitude and this is well illustrated by the fact that ∼6 × 10^14^ of the bottom DNA origami sheets would be required to cover the surface area of the top optical table, assuming side-by-side packing of a *ca.* 3 × 1.25 meter optical tabletop.

New optical phenomena are already being pursued using DNA scaffolding. For example, noble metal and other NPs are being arranged on DNA in an effort towards creating metamaterials with interesting chiroplasmonic and signal enhancement properties, *albeit* in a slightly different manner than that envisioned for pursuing efficient ET. In this context, DNA scaffolding provides the ability to bring gold NPs, nanorods and other NPs into very close proximity with each other to permit formation of plasmonic hotspots.^19–26^ It has been repeatedly confirmed in this context that DNA's prescriptiveness uniquely allows for nanoscale changes in material positioning across multiple parallel structures and this enables a direct comparison between theoretical prediction and experimental observation in a facile manner. An overview of these and similarly related applications are available in several excellent reviews and are not discussed further.^10,27^ However, it is important to appreciate that the NP sizes in the majority of these examples far exceeds that of a nucleotide or even a small dsDNA sequence, sometimes by orders of magnitude. Thus, many-times the NPs display the DNA around themselves where it is used to link them together or arrange them into defined patterns. Here, the DNA can, for all intents and purposes, be considered the connective tissue that links the large NPs together if you will.

Here, we provide a state-of-the-art review on the growing role of structural DNA technology as a nanoscale optical breadboard for hosting multistep excitonic ET configurations using primarily sequentially placed fluorophores as the optically active material, unless otherwise stipulated. We constrain ourselves to multichromophore – multistep configurations *(i.e.*, displaying two or more FRET steps) and where the organic dyes are covalently attached to DNA, or in certain cases where other fluorescent moieties, be it a NP or a protein, are covalently attached to DNA. Although certainly fascinating and quite related, for the purposes of focus and brevity, we do not focus extensively on structures that utilize DNA to ‘stack’ and form excimers and exciplexes from, for example, porphyrins and polycyclic aromatics such as pyrenes or phenanthrenes along with limiting inclusion of DNA intercalating dyes and fluorescent nucleosides. These materials and their contributions to DNA photonics are highlighted in other excellent reviews.^28–33^ Beyond a few pertinent examples, we also avoid the far more complex processes of how nanoplasmonics interacts with FRET in these structures; the interested reader is referred to several detailed treatises on the subject.^34–37^ We begin by providing a brief primer on how different fluorophores can be chemically incorporated into DNA structures and the ET processes relevant to this discussion that are manifest within such assemblies. Starting with the initial demonstrations that opened this field, the development of subsequent generations is followed with the creation of more complex systems incorporating a growing numbers of ET steps. More importantly, we highlight how DNA design plasticity in conjunction with the lessons learned from prior examples have helped increase the overall end-to-end efficiency achievable as the number of ET steps and scaffold complexity were expanded. Potential applications forming the impetus behind each configuration are discussed along with the growing variety of fluorophore materials finding utility in these structures, however, the primary focus is on the materials, the ET processes manifest on them, and the lessons they have provided. In some cases, a particular study is mentioned in two different sections as we focus on different aspects or phenomena in that example, such as the presence and contributions of homo- *versus* heteroFRET. Looking to the future, we also delve in depth into the growing efforts to utilize DNA scaffolding for assembling molecular dye aggregates that host delocalized excitons; these are being pursued towards creating excitonic circuits and accessing other quantum-like optical phenomena. For this area, we also provide relevant background and formalism on the relevant ET processes along with discussion of the detailed modeling and simulation required to properly understand these processes. Focus is also devoted to describe the more complex spectroscopic experiments required to probe these ultrafast photophysical processes. We conclude with an outlook on remaining issues and what may be expected in both the short- and long term. Lastly, we note that the use of DNA and other biomaterials in this role has been highlighted previously and we build from these excellent reviews.^11,13,28,29,31,38–48^ Given the growing breadth of this field, we also acknowledge that we cannot discuss every example and our apologies are extended for any and all omissions.

## 2. Background

The many interconnected variables that contribute to the design parameters that govern the configurations achievable within these hybrid biomaterials should be carefully considered by experimentalists in both setting up a given assembly and then interpreting the subsequent results. We begin by providing a brief primer on some of these such that the subsequent discussion of progress in this area can be properly appreciated by the reader. This includes: (i) an overview of DNA as a unique modular nanoscale scaffolding material; (ii) how DNA is synthesized and organic dyes are incorporated into a nascent oligonucleotide as this directly influences how that dye will function; (iii) how other fluorophores beyond organic dyes can be attached to DNA assemblies; (iv) some basics on FRET, the role of molecular dynamic and numerical simulations in predicting and interpreting results, and why there is so much concern about dipole orientation; and (v) an overview of the formalism that governs delocalized excitonic phenomena in these structures with the pertinent figures of metric.

### 2.1. DNA-assembling the optical nanoscale breadboard

#### 2.1.1. DNA as a modular scaffold

Beyond its ability to store genetic information, and now even digital information,^49^ DNA also has many useful physicochemical characteristics to offer from a materials perspective.^50,51^ DNA is biocompatible, non-toxic, and its chemistry along with its primary structure is well known.^52^ DNA can be joined together into an almost endless polymer that is only limited by the ability to actually (synthetically) accomplish this. The DNA itself can be synthesized using organic chemistry and also by biochemical means with enzymes at relatively low cost. There are also many available enzymatic and other chemistries to modify it. For the current purposes, its ability to assemble into programed secondary, tertiary, and quaternary structures is particularly relevant. Further, DNA tolerates immobilization on a wide variety of surfaces along with going from aqueous phase to being dried or even frozen almost endlessly. DNA is also tolerant of heat, cold, and many environmental or chemical insults along with being stable in the presence of many organics.^50,51^ In the right circumstances, it can even remain stable and recoverable for hundreds of thousands of years; this has allowed for impactful discoveries about human history, as highlighted by the 2022 Nobel Prize in Physiology or Medicine awarded to Svante Pääbo for his research into genomes of extinct hominins and human evolution.^53,54^ Here, we limit our coverage of DNA and DNA nanotechnology to the information on synthesis and modification with fluorophores needed to appreciate DNA and its inherent versatility as an optical scaffold. For more extensive presentations of the subject the interested reader is referred to a number of excellent reviews.^12,13,43,46,55,56^

**Fig. 2:**
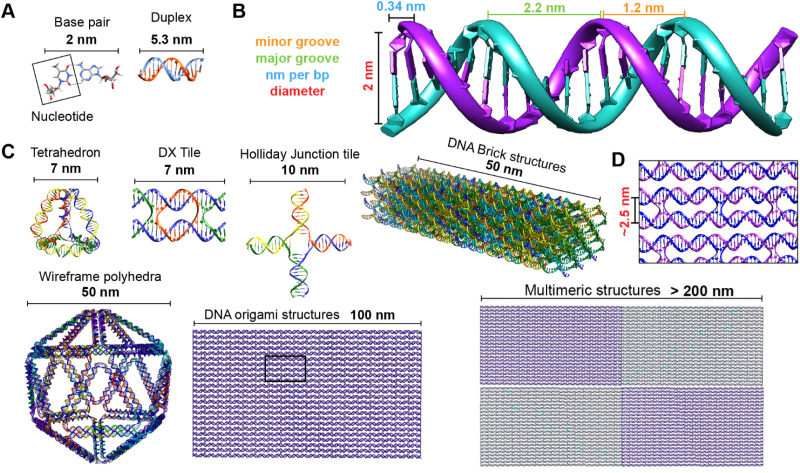
Representative DNA structures highlighting size, increasing complexity, and select properties of what some different assembly approaches can provide. (A) The nucleobases on two nucleotides hydrogen-bonded to each other (2 nm wide) and a 16 bp dsDNA sequence (B-form DNA) that is 5.3 nm long. (B) Other notable dimensions of a dsDNA include the major and minor grooves and the spacing between adjacent base pairs. (C) Top: Representative DNA tetrahedron,^64^ a DNA double crossover tile (DX tile),^58^ a Holliday junction (HJ),^65^ and a DNA block assembled using the DNA brick approach.^18^ Bottom: left to right shows a wireframe polyhedral,^17^ a rectangular DNA origami,^15,21^ and a tetraplex of linked rectangular origami. (D) Magnified inset from the rectangular DNA origami in (C) showing the typical inter-helical spacing within a DNA nanostructure.

Driven by fundamental base pair complementarity and other non-covalent interactions between single strands of DNA (ssDNA), synthetic DNA nanostructures can be designed to self-assemble and form two- and three-dimensional shapes and architectures that range from a few nanometers to nearly a millimeter in size, see Fig. 2. To accomplish this, ssDNAs are programmed to possess inter- or intra-strand complementarity to achieve a desired architecture and this is driven by a few overarching design guidelines, namely that of DNA tiles, DNA origami, bricks, or polyhedral wireframes. DNA tiles were originally introduced in 1981, wherein a small set of short ssDNA strands possessing 2–3 domains (or complementary subsequences) were designed to be complementary to each other.^57^ Self-assembly of these ssDNA strands resulted in a DNA tile with 4 dsDNA arms in the form of an artificial Holliday junction (HJ). The DNA tile could be modified to create multi-arm junction tiles, also bearing short “sticky” domains to bind to other DNA tiles and create higher-order networks of DNA tiles.^58^ DX tiles are a particular tile archetype wherein two antiparallel ssDNA strands are held together with shorter ssDNA strands in a series of single or double crossover junctions.^59^ In 2006, Rothemund's seminal work introduced DNA origami wherein a long ssDNA, referred to as a scaffold, is “folded” into a target shape as driven by a set of short ssDNA strands called staples (Fig. 2).^15^ DNA origami nanostructures are typically created using a circular viral ssDNA such as, for example, the ss m13mp18 bacteriophage genome as the scaffold strand to which 100–300 staple strands are bound thereby inducing the scaffold to resolve into a rasterized pattern of parallel double helices bound together *via* double crossover junctions.^15,16,60–62^ In 2012, a set of ssDNA strands called DNA bricks were first designed to assemble into a three-dimensional block “canvas” from which arbitrary shapes could then be “carved out” by identifying the requisite subset of DNA bricks.^18^ Like origami, DNA bricks also self-assemble to create a rasterized parallel helical pattern joined by multiple single crossover junctions. A similar approach was introduced in 2012, called DNA *scaples*, which also utilizes a pool of short ssDNA strands to create DNA origami-like nanostructures.^63^ DNA scaples eliminate the dependency on a scaffold strand by replacing it with a set of short ssDNA strands while preserving the underlying design of a DNA origami structure. In some sense, DNA bricks and scaples are a blend of DNA tiles and origami techniques. The DNA origami approach was further expanded in 2016 by creating DNA nanostructures with wireframe polyhedral architectures.^17^ Such wireframe-based nanostructures are not limited to rasterized parallel double helices but can conform to any three-dimensional layout. Given these, and the many other available DNA assembly approaches, the scope of feasible nanoarchitectural complexity that can now be accessed with designer DNA structures is vast.

Identifying the appropriate design technique for creating an optical nanoscale breadboard from synthetic DNA often depends on the physical and chemical environment along with functionality expected for that breadboard in the downstream application. Each technique is useful within a range of size and complexity. DNA tiles are well suited to create small (10–20 nm) repeating units that could be assembled into higher-order (two-dimensional) networks. Assembly efficacy of DNA tiles reduces with increasing size of the higher-order tile network and it is difficult to contain the size of the network due to the nature of DNA tiles; strands to block edge tiles from binding to more DNA tiles are required.^66^ The other approaches to DNA nanostructure design have a broader scope in size and complexity, ranging from 10 nm to 1 μm^67^ but require custom-length scaffolds.^68^ Parallel helix bundle and wireframe polyhedral DNA origami techniques form with over 90% efficiency while bricks and scaples are up to 40% efficient in their formation. In all but the wireframe polyhedra, the density of DNA is high and offers more sites per 10 nm to attach optically active species. For example, a wireframe DNA icosahedron has 78% of its DNA on its surface whereas a DNA origami sphere is 100% surface covered at roughly similar overall diameter. While these examples are hollow by design, a DNA block made using brick strands of the same size would approach having 100% of its DNA within its volume. In DNA origami, of course, it is more challenging to chemically modify the scaffold strand with fluorescent molecules (given that it is long and usually not chemically synthesized) but in the case of DNA bricks there are, in theory, twice the number of available sites to chemically modify a resultant optical nanoscale breadboard. Control over the underlying sequence is also a determining factor as optically active molecules can be sensitive to the presence of guanine bases in their immediate vicinity.^69^ Current DNA synthesis technology still finds it challenging to synthesize a long ss scaffold strand (>500 bases) *de novo* but synthesizing short ssDNA strands (<50 bases) with specific sequences (that constitute DNA bricks, tiles, and scaples) remains straightforward, efficient, and inexpensive. The different approaches to assemble DNA nanostructures discussed here are compatible with each other as well thereby expanding their applicability within hybrid DNA constructs; thus, it is possible to create a hybrid DNA origami nanostructure that also possesses elements of DNA bricks, *etc.* if needed.

The process itself of designing and assembling DNA breadboards (or other desired architectures) across the different design strategies is versatile and enables rapid transition from conceptualization to implementation. *In silico* programs are available for designing and predicting the physical properties of DNA nanostructures.^70^ DNA nanostructures can be designed using the software programs caDNAno,^16^ Tiamat,^71^ DAEDALUS,^17^ Adenita,^72^ and Perdix,^73^ for example. In the case of DNA origami, the main output of these tools is a set of ssDNA (staple) strands that will predictably fold a given scaffold into the desired DNA structure in solution. A benefit of using many of the aforementioned programs is that most, if not all, will provide the strands in a format that facilitates ordering from a commercial vendor, or uploading the sequence to a synthesizer control program. Actual assembly process(es) entails combining the constituent ssDNA strands in a cation-enriched buffered solution of pH 7–8. Typically, equimolar quantities of ssDNA strands are mixed together in 10–20 mM MgCl_2_ containing buffer solution. For DNA origami and wireframe structures, the assembly kinetics are driven forward by adding 5- to 10-fold excess staple strands relative to the scaffold strand concentration. The solution of DNA strands is then thermally annealed by heating first to 80–90 °C for 5 min to melt any preformed structures followed by a slow and programmed cool-down to room temperature or to refrigeration. The self-assembly process, in fact, occurs around a specific temperature depending largely on the physical complexity of the DNA nanostructure,^74^ and this makes it possible to rapidly anneal a DNA nanostructure isothermally at a specific temperature in a mere few minutes rather than using a lengthy thermal annealing program.^75^ However, some complex structures may require extended annealing programs to optimize formation and these are usually determined empirically. Once annealed, the DNA breadboards can be purified and characterized by a number of established techniques as summarized previously.^76^ More importantly, depending upon the concentration of reactants used along with the number of replicate reactions set up in parallel to it, the yield for a given DNA structure can be achieved with copy numbers approaching a significant fraction of Avogadro's number (*i.e.* millimoles).

#### 2.1.2. DNA synthesis

When the first DNA strands were chemically synthesized over 65 years ago,^77^ the state of the field was dramatically different from where we find ourselves today. For example, it took one scientist over 2 years of effort to generate a small oligomer of less than 50 bases.^78^ More importantly, barring just a few forward looking researchers, the scientific world could not envision the practical value of synthesizing DNA in the lab let alone fathom the broad interdisciplinary sciences that it would facilitate beyond genetic engineering to now include nanomaterials science.^79^ The versatility of DNA in both these roles can be directly attributed to the rapid development of a chemical approach for oligonucleotide synthesis involving phosphoramidite precursors and a solid-state support.^80,81^ This synthesis paradigm achieved the primary requirements of any technology to be viable, namely, high yield, speed, and programmability, making it possible for anyone to rapidly synthesize a 20 mer in a matter of hours using a commercial synthesizer no bigger than a child's dollhouse (*e.g.* a Barbie Dreamhouse Dollhouse with dimensions of ∼0.9 × 1 × 0.8 m^3^ L×W×H) just by following an established protocol. Commercialization has injected high-throughput engineering into the technology, bringing down the cost of synthesis from several dollars a base 25 years ago to a few cents per base *ca.* 5 years ago and to now less than a tenth of cent per base for bulk synthesis from select commercial vendors.

**Fig. 3:**
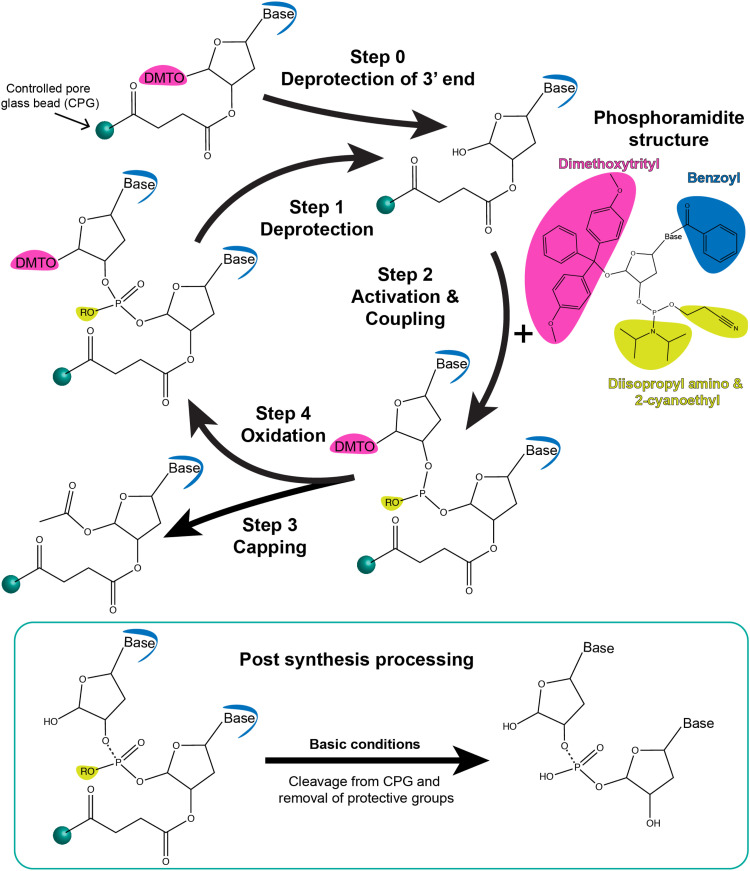
Schematic showing step-wise oligonucleotide synthesis *via* phosphoramidite solid-state chemistry. Synthesis is carried out by the addition of one nucleotide per coupling cycle, as shown here. Step 0: the dimethoxytrityl (DMT) protective group of the 3′end base that is bound to the controlled pore glass bead (CPG) is removed using deblocking reagents such as 3% trichloroacetic acid in dichloromethane. Step 1: The same process occurs during synthesis in which the DMT group on the last nucleotide attached to the CPG-oligo is deprotected. Step 2: Representative structure of the phosphoramidite derivative of a nucleotide is shown. It contains protective groups, namely, DMT (pink), benzoyl (blue), and diisopropyl amino and 2-cyanoethyl moieties (yellow). The next nucleotide-phosphoramidite is mixed with activating reagent such as 5-ethylthio-1*H*-tetrazole and introduced to the CPG for coupling. The activator renders the nucleotide-phosphoramidite reactive at the 5′ P-atom which leads to a phosphodiester bond with the last nucleotide on the CPG. Step 3: Unsuccessful coupling reactions are left with a reactive hydroxide group on the 5′ end of the CPG beads. These failed reactions are capped to replace the hydroxide with acetic anhydride using capping reagents such as 1-methylimidazzole in tetrahydrofuran/pyridine and tetrahydrofuran/acetic anhydride. Step 4: The oxidation state of the 5′ atom is changed from +3 to +5 using oxidizing reagent such as iodine in tetrahydrofuran/water/pyridine. The process is repeated until all nucleotides of a desired sequence are coupled from the 3′ end to the 5′ end. Below: Post-synthesis processing includes cleavage and release of the oligonucleotide from the CPG beads as well as hydrolysis of the bases and 5′ P atoms to remove the corresponding protective groups (shown in yellow and blue). Highly basic conditions such as ammonium hydroxide (7–30%) are typically used. The oligonucleotide can be used as is or further purified using reverse phase chromatography from any partial and incomplete sequences. Modified bases and dyes are inserted into the sequence using their phosphoramidite derivatives.

Phosphoramidite chemistry on a solid-state support, summarized in the schematic shown in Fig. 3, is based on synthesizing the oligonucleotide in a stepwise manner from monomeric forms of each of the nucleotides.^81^ Monomers other than phosphoramidites have recently been developed as well that follow the same process of oligo synthesis.^82^ Each monomeric phosphoramidite is an unreactive (but highly water sensitive) version of the nucleotide where the reactive amine, hydroxyl, and phosphate groups on the nucleotide are rendered inactive *via* protective groups such as benzoyl, dimethoxy- or monomethoxytrityl, diisopropylamino and 2-cyanoethyl groups, respectively. During synthesis, the protecting groups on the sugar hydroxyl and phosphate are systematically removed for coupling and at the end of the synthesis the protective groups on the bases are removed. Phosphoramidite DNA synthesis generally builds oligos from the 3′ to 5′ end and involves four steps to add one nucleotide; these are 1-deprotection, 2-activation and coupling, 3-capping, and 4-oxidation (Fig. 3). The most commonly used solid-state supports for synthesis are commercial controlled pore glass (CPG) beads (5 to 15 μm size) upon which the 3′ end nucleotides are already pre-attached and from which the nascent oligonucleotide is chemically grown in a stepwise manner. During the first deprotection step, the trityl group protecting the 5′ carbon on the sugar of the previous base is removed by trichloroacetic acid (TCA) to render it reactive. Second, tetrazole-catalyzed activation of the hydroxyl on the new phosphoramidite takes place followed by coupling *via* what at the end will be a stable phosphodiester bond. Thirdly, any free hydroxyl groups that failed to couple are capped by acetylation to block the synthesis of oligos that are missing one nucleotide. Lastly, iodine is introduced to stabilize the newly formed phosphite to form the phosphodiester bond. This stepwise process is then repeated iteratively to incorporate the different bases that make up the target oligonucleotide sequence.

After the addition of the terminal 5′ nucleotide, the oligos are chemically cleaved from the CPG beads, the remaining base detritylated, and the oligo deprotected *via* NH_4_OH to yield the desired ss oligonucleotide product. Subsequent purification is usually performed *via* desalting, gel electrophoresis, and/or high performance liquid chromatography (HPLC). High-throughput approaches using an aminopropylated silica gel and chemoselective ligation are also being formulated for purifying synthetic DNA from solid-state supports.^83,84^ Most DNA synthesizers allow for switching between a range of 0.05, 0.1, 0.2, 1, and 10 μmole synthetic scales with available commercial reagents including the CPGs matched to these.^85^ Phosphoramidite chemistry also comes with the inherent benefit that trityl removal during deprotection step can be monitored either by absorbance or electrochemistry to give a real-time readout of the efficiency of each step and an estimate of the final yield. For 20-mer oligos, yields of 99% are achievable and it is feasible to synthesize oligos 150 to 200 bases in length but with exponentially diminishing yields. Nevertheless, recovering 1% of a 150 mer oligo synthesized at a 1 μmole scale will still yield a considerable amount of usable DNA (∼15 nanomoles).

A whole new alphabet of DNA structural and chemical analogues has also been developed. These includes peptide nucleic acid (PNA),^86^ pseudocomplementary peptide nucleic acids (pcPNA),^87^ locked nucleic acid (LNA),^88^ bridged nucleic acid (BNA),^89^ glycol nucleic acid (GNA),^90^ and morpholino oligonucleotides.^91^ Each of these analogues offers different benefits such as resistance to nuclease activity or higher melting temperatures; others have elegantly reviewed the chemistry and utility of such analogues.^92–95^ Synthesis of DNA analogues varies with type, but many of these analogues are easily incorporated into canonical DNA sequences using corresponding phosphoramidite monomers for solid-state synthesis.^96,97^ Incredibly, DNA translation into these analogues can also be done thereby expanding the field of sequence-controlled polymers.^98^ For high-yield DNA synthesis beyond the scales described here (yield in grams), interest is now turning to enzyme or bacterial based chemical processes.^68,99^

#### 2.1.3. Dye incorporation

**Fig. 4:**
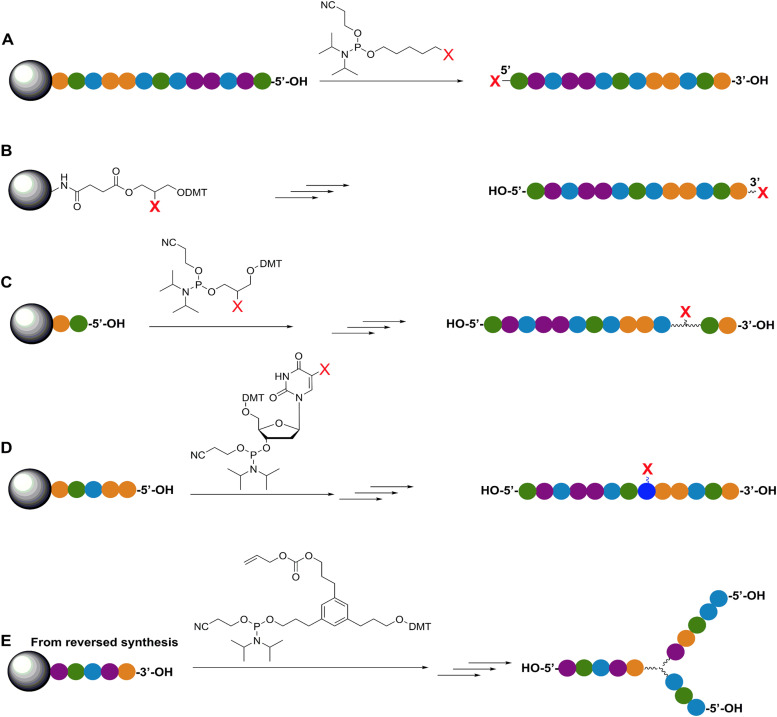
Representative in-synthesis modifications of DNA (denoted as X). (A) Scheme showing the general strategy for 5′-modification of DNA using a modified phosphoramidite without hydroxyl-handle for further chain elongation. (B) 3′-modification of DNA using a modified solid support. (C) Internal modification of DNA using a non-nucleobase phosphoramidite containing the modification. (D) Internal modification of DNA using a modified nucleobase in order to maintain Watson–Crick–Franklin base pairing in the oligonucleotide product. In this case modified dT is shown, but all four bases can in principle be employed, although dT and dA remain the most popular sites for modification. Note that the oligonucleotide products on the right-hand side are rotated 180° to display the sequences in the 5′ to 3′ direction. (E) Branching modifications can also be employed in DNA synthesis. Reproduced with permission from ref. 51 Copyright 2019 American Chemical Society.

**Fig. 5:**
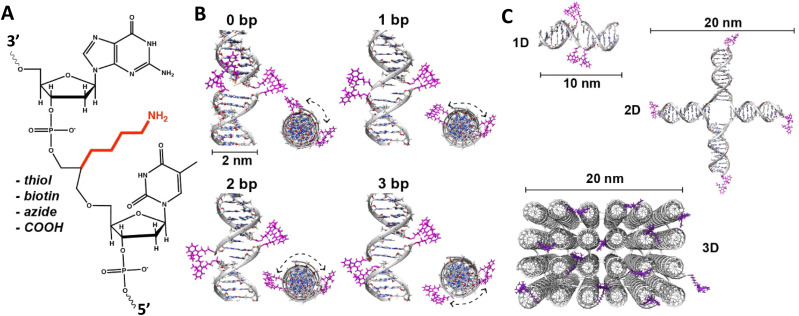
DNA modification and multidye placement on increasingly complex structures. (A) ssDNA synthesized to display a primary amine (internal universal-link amino modifier) on the end of a 4 carbon linker. This would then be labeled by amino-reactive dyes such as those functionalized with N-hydroxysuccinimidyl esters. Other common groups inserted for chemical modification are also listed. (B) Dye (pink) insertion into complementary strands with the number of bps separating the dyes indicated to highlight the effect of dsDNA (silver) twist (C) Dye placement highlighted on increasing larger and complex DNA structures including a 1D ds, a 2D HJ, and a 3D DNA brick-based structure where the dyes are found across an internal plane. Models in (B) and (C) were created in UCSF Chimera.^102^

As mentioned, a key requirement for achieving programmable optically active DNA structures is that of precisely controlling how the dyes are positioned within the assembly, and thus the chemistry utilized to insert them into DNA sequences is an important consideration. Fig. 4 depicts schematically the most common DNA dye insertion and attachment approaches currently utilized, with many of the chemical precursors available through commercial oligonucleotide vendors.^51,100^ As phosphoramidite chemistry is somewhat modular in nature, it inherently allows for the insertion of phosphoramidite dye derivatives into a nascent DNA strand during oligo synthesis at the 5′ or 3′ ends and also internally in a manner analogous to a nucleotide monomer. For 5′ and internal coupling, there are phosphoramidite derivatives that are available for dye attachment to the DNA phosphate backbone *via* phosphodiester chemistry (Fig. 4A and C) or derivatives thereof where the dye is conjugated to a chemical handle on a modified dT nucleotide (Fig. 4D). It is also possible to purchase a universal CPG support and start with the modified dT nucleotide at the 3′ to insert a 3′ dye *via* the dT ‘handle’ using standard post-synthesis chemistry. For 3′ attachment, commercial CPG derivatives are available with the dye already attached to the beads (Fig. 4B). However, not all dyes are stable under highly basic conditions, which is a requirement of post-synthesis cleavage of oligos from CPG beads. Furthermore, not all dyes are capable of yielding a viable phosphoramidite derivative. In such cases, an alternative approach is to introduce functional groups during synthesis by using modified nucleotide phosphoramidites. For example, individual deoxynucleoside triphosphates (dNTPs) can be chemically functionalized to display an appropriate target group,^101^ and then be included in the DNA synthesis at the desired position. Using these precursors, reactive groups or chemical handles such as amines, thiols, and carboxyls could be inserted into the DNA during- or post-synthesis (see Fig. 5A) and then conjugated with the target dye downstream of oligo purification using standard bioconjugation techniques.

An important consideration arising from this type of chemistry is the length of linker now present between the DNA and the dye attachment point, more on this immediately below. Table S1 (ESI†) summarizes the more common types of reactive groups and dyes used, their availability as phosphoramidite derivatives or as modified dNTPs and some examples of their application. Lastly, it is worth considering that in many cases two or more dyes (or other functional groups such as 2× amine or a mixed amine/thiol) can be inserted in DNA during synthesis, however, this will reduce yield and certainly increase cost. Placement of multiple dyes into opposite strands of ds sequences and other higher order DNA assemblies needs to further account not only for the space between the dyes but also for the inherent twist and rotation of the DNA itself, see Fig. 5B for a simplified ds structure that highlights this point. Note that the dye structures are not shown in the manner that they would actually assume in solution. The size and complexity of a DNA assembly and further whether it is 1D, 2D, or 3D in nature (Fig. 5C) can further complicate available choices for dye placement since inappropriate placement can destabilize critical portions of an assembly or force an unintended or undesired configuration.

**Fig. 6:**
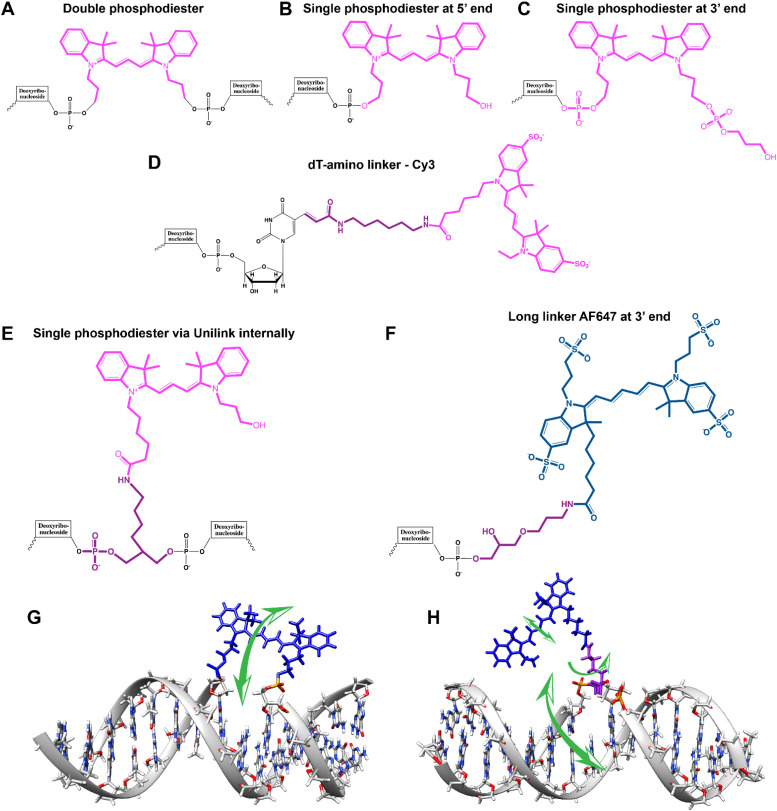
Representative DNA–dye conjugation strategies. (A) Double phosphodiester linkages (at 3′ and 5′ ends) for internally coupling a Cy3 dye (pink) for instance – to the oligonucleotide phosphate backbone. (B) End-coupling the Cy3 to the 5′ end of the oligo. (C) End-coupling a Cy3 to the 3′ end of the oligo. Note, often the 3′ end-coupled dye could have an addition C3 spacer group on the non-attached propyl chain that supports attachment to the CPG bead during synthesis. (D) Internally- or end-coupling Cy3 *via* a C6 amide linkage onto an amine-modified dT nucleotide. This labeling would usually occur post-synthesis using a N-hydroxysuccinimide (NHS-ester) activated version of the dye. (E) Internally coupling the dye but using a short C3 amide linkage and single point of dye attachment to an amine inserted into the DNA during synthesis. This would also be post-synthesis. (F) Internally coupling a different dye – Alexa Fluor 647 (indigo) to an amine on a linker inserted during synthesis – but using a longer linker. (G) The double phosphodiester linkage will severely restrict rotational motion in the dye – Cy5 (blue). (H) A single phosphodiester linkage allows for far more rotation about the linkage as well as along the polymethine bridge of Cy5. The former could promote dye interaction with the major groove while the latter could lead to photoisomerization.

Ideally, dye insertion would significantly constrain the dye's freedom of movement and orientation within any DNA assembly (unless movement is desired) and, in turn, potentially allow for control over dipole orientation especially relative to other dyes. As shown in Fig. 6, current dye attachment chemistries may not allow for this level of control (less than 1 nm) due to the presence of extended aliphatic linkers between the dye and/or its point of attachment on the DNA strand. Many dyes are available for insertion as a double phosphoramidite, which means that they will replace a nucleotide in the sequence and be attached to the sequence at both (the equivalent of their 3′ and 5′) ends. This can be a preferred insertion strategy as it will constrain the dye the most and also allow for the most accurate inference of dye position in any ds structure.^103^ Other single point insertions or post-synthesis labeling with a dye typically add an extended linker between the DNA backbone and the dye and this allows for a far greater range of movement in any final DNA structure, see Fig. 6E–H. With any of these approaches, in the worst case the linkers would allow completely free motion, and would thus imply a randomness in the positions and orientations of the dyes. However, it has been found that the existence of the DNA scaffold can itself introduce a significant constraint. The Acuna group has recently demonstrated that the DNA scaffold itself can be used to determine fluorophore orientation, with up to 15° precision, by using polarization microscopy in combination with DNA-PAINT superresolution microscopy to determine the orientation of the underlying DNA nanostructure itself.^104^ Even more interesting is that it may also be able to help address the challenge of orienting the dyes.^105,106^ They showed how single Cy3 and Cy5 dye molecules could be incorporated in a DNA origami nanostructure with controlled orientation by doubly linking them to oligonucleotide strands that were hybridized while leaving key bases unpaired in the scaffold. Increasing the number of bases unpaired induced a stretching of the fluorophore linkers and reduced its freedom of mobility, which left more space for the fluorophore to accommodate and find different sites for interaction with the DNA. After exploring structures with 0, 2, 4, 6, and 8 bases unpaired, they found extreme orientations for 0 and 8 unpaired bases, which corresponded to the dye molecules being perpendicular and parallel to the DNA double-helix, respectively.^105^ Work from Cervantes-Salguero and colleagues was realized in parallel and obtained similar results.^106^

It is also important to consider that despite the movement allowed by many of the dye insertion or conjugation chemistries, in many cases the dyes will relax into a preferred configuration relative to the localized DNA sequence. For example, Ouellet *et al.* showed *via* single molecule FRET (smFRET) experiments that cyanine dyes attached to the 5′-termini of DNA and RNA with C_3_ linkers stack on the ends of the helix, potentially orienting their transition moment.^107^ Such structural orientation was later confirmed with X-ray crystallographic analysis.^108^ Interestingly, it has been suggested that placing cyanine dyes at the end of DNA may even stabilize duplex structures.^109^ In contrast, internally-labeled Cy3 dyes adopted very different conformations in DNA duplexes based on the presence of weaker A–T or stronger C–G bonds present in the flanking bases along with whether a second dye was present directly across from it on the complementary strand; in this case extended molecular dynamic (MD) simulations in conjunction with UV-vis absorption measurements proved critical to making the correct structural determination.^110^ To overcome many of the issues surrounding lack of control over rigid dye constraints on DNA scaffolds, several groups have turned to synthesizing their own dyes in the form of custom phosphoramidite derivatives which are then incorporated during standard DNA synthesis as described above.^111,112^ This can be a challenging undertaking as it requires specialized multistep synthetic chemistry with highly labile reagents. Other approaches look to rigidify the DNA scaffold itself which then exerts a constraining influence on the dye.^113^ There are other important points to be considered with dye insertion chemistry including the fact that proximal nucleotides can quench dyes in a manner that is still not predictable, though C and G bases more often appear to be the primary culprits here.^69^ Additionally, depending on the complexity of the final desired architecture in conjunction with the number of oligos that are required to construct it and how they align with each other, the assembly can experience ‘breathing’, *i.e.*, spontaneous-transient local conformational fluctuations within dsDNA, around nicks, ends, and where dyes and other molecules distort complementarity.^114^ Overall, the key points to appreciate here are that the ideal level of constraint over dye movement on DNA structures can be hard to achieve but there exist several ways to compensate for this. There are also excellent resources available to help choose a dye for a particular application.^115–118^

#### 2.1.4. Attachment of other fluorophores to DNA

From a FRET perspective, the ability of one fluorophore to couple with another very different fluorescent material is dictated by their respective photophysical properties along with the ability to achieve sufficient proximity between the two. In most cases, FRET itself is agnostic as to what the materials are so long as the excitons can couple, and this opens up the possibility of mixing and matching different fluorophores on DNA scaffolding in pursuit of new applications. A comprehensive discussion of the number of different fluorescent material families (*e.g.*, discrete organic dyes, metal chelates, polymers, fluorescent proteins, NPs) now available along with how to attach them to individual DNA sequences or within DNA structures is clearly beyond the current text. The interested reader is referred to some of the many detailed reviews that focus just on this aspect.^51,84,95,119–121^ However, a cross-section of some of the issues, guiding principles, and working rules of thumb are still worth iterating here as they can help focus the key questions to ask when designing a photonic wire assembly and their consideration will ultimately help dictate the performance obtained in the final construct.

First, and foremost, is that of control over as many variables as possible so that as much as possible is known or can be inferred about the position of a given fluorophore within a DNA construct regardless of its properties.^122^ As discussed above, many of the activated dyes and functional groups available for insertion into nascent DNA or post-synthetically come with a range of linker or spacer lengths (see Fig. 6). For example, if an amine within a DNA sequence is to be post-synthetically modified with an NHS-ester activated dye, then picking from amongst amine modifications (with C_3_, C_6_, C_12_ carbon linkers) and activated dyes that have the shortest linkers is probably the best approach as it will constrain the range of movement of the dye within the final DNA assembly. Sometimes the nature of the linkage chemistry itself may impinge on subsequent application. For example, azide–alkyne cycloaddition can produce relative short linkages which is desirable, but the use of a Cu^I^ catalyst and/or heating can chemically degrade dye properties.^123^ More recently developed Cu-free cycloaddition chemistries using strained cyclooctynes may be a more viable alternative.^124^ It is worth noting that some of the functional groups, such as the azide and alkyne, would first have to be introduced to the respective dye and DNA by conventional NHS-chemistry.^124^ Similarly, chemoselective ligation between aldehyde and hydrazine functionalities takes place in the presence of a vast excess of 100 mM aniline catalyst which can also quench fluorescence and so careful purification now becomes essential.^125^

Although biotin–streptavidin binding is one of the most facile and accessible bioconjugation chemistries in general, it is not very ‘controllable’ since streptavidin is tetravalent and is also quite large with a mass of ∼53 kDa (∼5 nm globular dia.) and avidin has a positive charge which will interact with DNA's negative charge (see also below).^119^ If unavoidable, smaller monomeric avidin may be a better choice along with use of recombinant deglycosylated Neutravidin.^119^ Fluorescent NPs such as QDs can be powerful components in these assemblies but most are far larger than dyes and even the DNA assembly itself, and this factor must be carefully considered since, in many cases, the results may be inverted where multiple DNA constructs may actually be displayed around the NP itself.^64,126^ Placing multiple NPs in close proximity to each other on a DNA are probably not feasible due to steric hinderance. Due to the limitations of NP bioconjugation chemistry, attaching a DNA to a given particle surface may require modifying both the NP and the DNA with some tertiary chemistry that then facilitates the desired linkage.^119,127^ A first approach with NPs in many examples is often relying on commercial streptavidin conjugates that are then paired with biotinylated DNA. Beyond the issues described above, this can also drive crosslinking, aggregate formation, and ultimately precipitation if NP-DNA stoichiometry is not carefully balanced.^41^ Luminescent gold and other noble metal nanocrystals that emit in the near-IR are an interesting recent development and their small size (<2 nm dia.) is driving interest especially given the dearth of FRET accepting molecules in this portion of the spectrum.^5^ As some noble metal NPs such as those based on silver can rapidly oxidize, storage time may now become a consideration. Additionally, it is not well appreciated that the dative thiol–noble metal interaction that is commonly exploited for attachment to such materials can have a high on–off rate in practice. Modifying a DNA to display a dithiol rather than a monothiol for attachment to such materials can help address this issue especially since dithiols can be opened and directly bind to gold surfaces.^128^ Trying to incorporate fluorescent and other carbon allotropes such as carbon nanotubes or buckeyballs into an assembly will have to consider not only their size but also their lack of intrinsic solubility or colloidal stability and the amount of modification chemistry needed to functionally join them.^129^ Interestingly, some carbon allotropes naturally bind to DNA and this may help facilitate formation of a FRET assembly.^130^ The issue may now become that of the DNA wrapping itself around the allotrope in an unpredictable and uncontrollable manner.

As intimated, the foregoing is certainly not comprehensive but rather serves to outline that incorporation of each fluorophore type, including even the main organic dyes under discussion below, brings with it a series of issues that should be carefully considered to optimize construction of the intended photonic wire and ultimately the FRET functionality within that wire. In other words – how something is made will dictate how it works and DNA photonic wires are no different. Some careful forethought on initial considerations can go a long way to help alleviate a lot of downstream troubleshooting and the need for redesign.

## 3. Excitonic energy transfer

### 3.1. Förster resonance energy transfer

The phenomena primarily displayed in the majority of examples discussed here outside of the sections focused on delocalized excitons is that of near field FRET.^117,118,131,132^ In FRET, an electronic excitation that is located on the initial donor (D) fluorophore will transfer its energy to the acceptor (A) molecule. Though A molecules need not be fluorescent,^133^ in general the resulting FRET-sensitized emission from the A makes FRET easier to observe and confirm.^118,134^ In the FRET regime, D–A molecular transition dipoles are only weakly coupled, resulting in excitons that are located on individual fluorophores; the excitons are described as hopping from one fluorophore to the next in this process. As an aid to understanding the work described here, key concepts, definitions, formulae, and common metrics used in describing FRET especially across multiple ET steps in DNA structures are briefly introduced.

For a discrete D–A pair interacting in the Förster regime, the energy transfer rate (*k*_FRET_) is given by:1
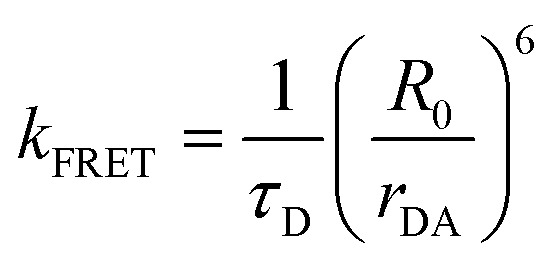
where *τ*_D_ is the lifetime of a D exciton in the absence of A and *r*_DA_ is the separation distance between the chromophores.^117,131,135^ The quantity *R*_0_ is the Förster distance, sometimes referred to as the Förster radius, corresponding to the separation distance at which a discrete D–A has a FRET efficiency = 50%. *R*_0_ provides a guide to the effectiveness of a particular D–A pair, with larger values resulting in more efficient FRET. *R*_0_ generally ranges from 2 to 10 nm and this distance also loosely reflects the rule of thumb that observable and quantifiable FRET will occur between a given D–A pair over a range of 0.5 × *R*_0_-to-1.5 × *R*_0_; other estimates put this range at 0.2-to-2.0 × *R*_0_ depending upon fluorophores.^117^*R*_0_ can be estimated, in nm, by the individual D and A properties as defined by:

2 *R*_0_ = 0.02108 × (*κ*^2^*Q*_D_*n*^−4^*J*)^1/6^

where *Q*_D_ is the D fluorescence quantum yield, *n* is the medium's index of refraction, *J* is the overlap integral, and *κ*^2^ is the dipole orientation factor which is given by:

3 *κ*^2^ = [sin *θ*_D_ sin *θ*_A_ cos *ϕ* − 2 cos *θ*_D_ cos *θ*_A_]^2^

where the angles *θ*_D_, *θ*_A_, and *ϕ* specify the relative orientation of the D–A transition dipoles.^117,136^*J* is the measure of the overlap of the D emission spectrum with the A absorbance, and can be determined using:4
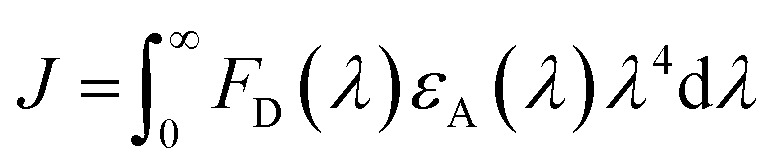
where *F*_D_(*λ*) is the normalized D emission spectrum, *ε*_A_(*λ*) is the A extinction coefficient in units of M^−1^ cm^−1^, and *λ* is the wavelength in nm. This choice of units results in *J* often being reported in nm^4^ M^−1^ cm^−1^, though we note that the units can be simplified and as such *J* is sometime reported in M^−1^ cm^3^ as well, in which case the constant in eqn (2) should be changed to a value of 978.^131^ FRET efficiency (*E*_FRET_) is given by:5
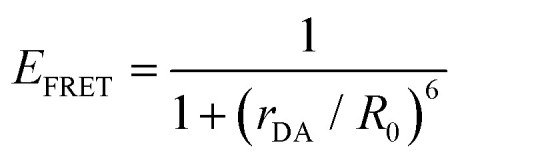
and is typically measured from the D's emission (*I*^D^) in the presence (*I*^D^_DA_)/absence of A (*I*^D^_D_) or equivalently the D's excited-state lifetime (*τ*^D^) in the same configuration with:6
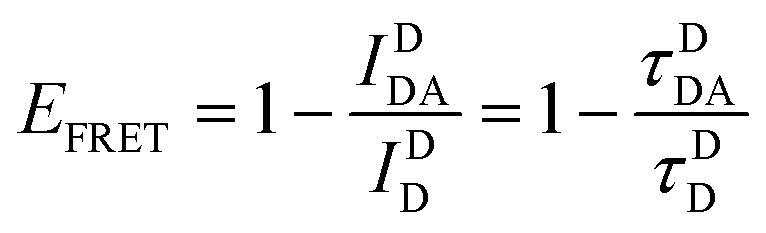


Eqn (6) assumes that there are no changes in the inherent *Q*_D_ in the presence of A; this is generally valid for simple dye-pairs, but cannot always be discounted.^137^*E*_FRET_ can also be estimated from the D-sensitized A emission component (*I*^A^_DA_), the A emission in the absence of D (*I*^A^_A_), and *I*^D^_D_ as well as *I*^D^_DA_, as seen in eqn (7). The equation presents two forms of sensitized emission that may be preferential depending on the instrumental or experimental set-up, *e.g.* spectroscopy or microscopy. However, this requires accounting for the ratio of *Q*_A_ and *Q*_D_, full integration of the D and A emission spectra (or equivalent corrections), and the wavelength dependence of the fluorescence spectrometer, and as such will often have greater uncertainty.^138^ In general sensitized emission is utilized more in microscopy applications. For a full breakdown of suggested corrections for quantitative FRET we direct the reader to the excellent review by Müller *et al.*^139^7
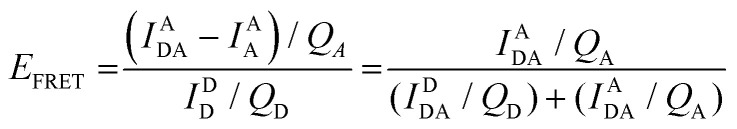
FRET is not limited to single D–A pairs, conceptually the equations can be expanded to multi-acceptor systems without much difficulty. The generic equation when each A can be considered an individual FRET A,^140^*i.e.*, all fluorophores are sufficiently spaced to avoid any strong coupling, is given by eqn (8):8
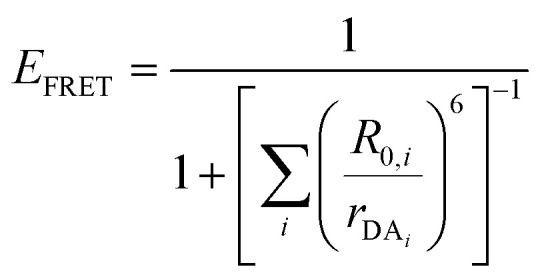
In this equation each D–A pair has its own *r*_DA*i*_ and *R*_0,*i*_. There are particular solutions of this equation that apply to common physical systems including a centrosymmetric distribution of equivalent acceptors (*e.g.*, QD D surrounded by organic dye As) or a point D to plane of equivalent A (*e.g*,. cellular membrane labeling).^135,141,142^ For deeper insight into the intricacies of FRET systems, the interested reader is directed to the many available specialized treatises.^40,118,131,132,134,135,143,144^

The above equations, even when involving multiple acceptors, deal primarily with a single FRET step. In theory, each FRET occurrence is independent of the preceding and/or subsequent ET steps; the efficiency of a FRET cascade should therefore be a simple multiplication of each individual *E*_FRET_ component. This, of course, assumes that a D does not bypass a proximal A relay dye and instead interact with another A located at a further distance in a D → Relay → A system.^145^ In practice when describing and quantifying FRET-based energy transfer through multichromophore structures, assumptions such as the equivalence of the fluorophores may not be valid. Oftentimes interactions with the medium can create unfavorable orientations or energetic trap states. Since it is often not feasible to characterize each fluorophore within a system, measurements that encompass the overall photophysical performance, such as end-to-end ET efficiency (*E*_ee_) and anywhere-to-end ET efficiency (*E*_ae_) are often used. These metrics are not-concerned with the intermediate/relay dyes but focus only on the initial D (denoted D′) and the final A (denoted A′) of the cascade with D′ having the highest energy fluorescence emission and A′ the lowest energy fluorescence emission. *E*_ae_ is defined as:9
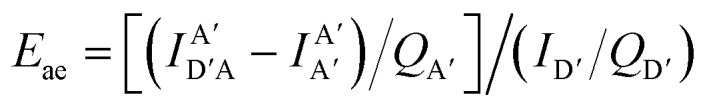
Here *E*_ae_ represents the fraction of excitons that reach A′ as a function of the photons initially absorbed by D′ at a specific wavelength. Because downstream relay fluorophores can themselves absorb excitation photons, there are occasions in which *E*_ae_ > 1.0. To account for this effect and to compare systems with varying number of relay dyes, the use of the *E*_ee_ parameter is preferred and is based on:10
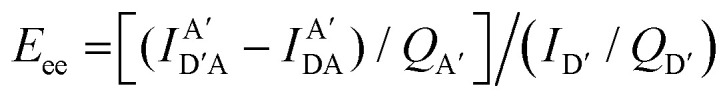
The difference in these formulas is that in *E*_ae_ the emission of A′ when only A′ is present is subtracted whereas in *E*_ee_ the emission of A′ when all (intermediary) dyes except D′ are present is subtracted. Essentially *E*_ee_ corrects for any downstream excitation and, as such, if an excitation wavelength could be accessed where only D′ was excited it would be expected that *E*_ae_ = *E*_ee_. This correction can be accomplished with multiple approaches including: a purely experimental strategy where each control structure is measured,^146^ a semi-analytical method that considers individual FRET efficiencies and respective extinction coefficients,^147^ or multi-wavelength excitation strategies.^148,149^ For eqn (6)–(10), it should be assured that each D is linked to at least one A because free Ds (in case there is an excess of Ds or Ds are too far away from As) also contribute to the measured emission, which is used to calculate the FRET efficiencies.

Other figures of merits commonly utilized to characterize molecular photonic nanostructures arise from their use as photonic antennae. These parameters measure characteristics broader than just *E*_FRET_ and can be dependent on the absorption cross-section of the structures, yet the structures' ultimate FRET capabilities are still deterministic of their final values. The two most common are the antenna effect (AE)^150^ and the antenna gain (AG).^151^11
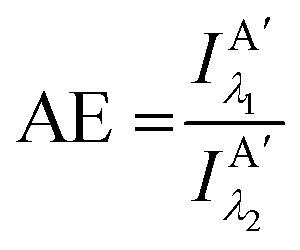
12
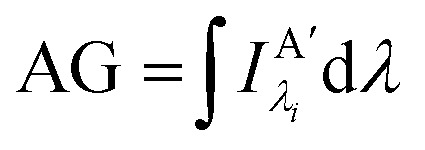
AE provides the relative A′ emission generated through sensitized emission (*λ*_1_ excitation generally chosen at the peak D′ absorbance) compared to that obtained by choosing *λ*_2_ for direct excitation of A′. AG is in concept very similar, with the distinction being that it integrates the A′ emission obtained by excitation throughout the entire spectrum. This is a better representation of the antenna working under white light irradiation. In practice, AG is often normalized to an arbitrary structure within the study to provide context for improvement or for comparison purposes. For further discussion of FRET and FRET formalism, the interested reviewer is referred to several more detailed discussions.^117,118,131,132^

### 3.2. Dipole–dipole orientation, kappa squared (*κ*^2^), and use of averaging

**Fig. 7:**
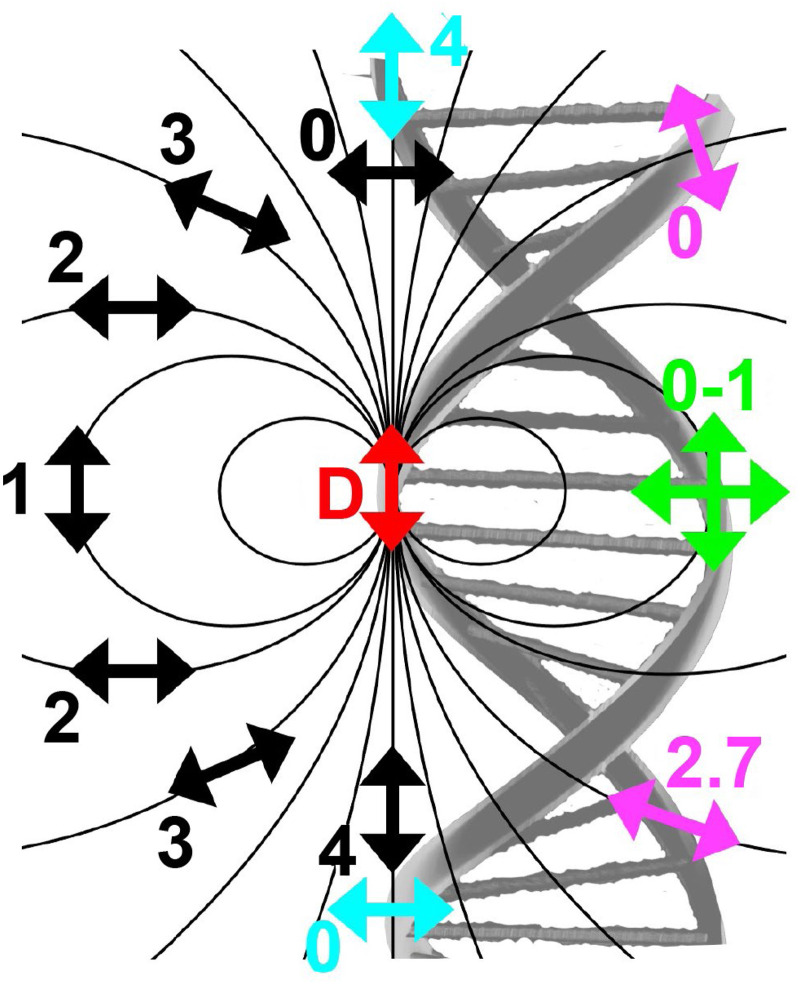
Schematic representation of relative orientations of transition dipole moments of D emission (D-red double-headed arrow) and A absorption. The left side (black double-headed arrows) represents orientation in free space. The right side (colored double-headed arrows) represents attachment of D and As to DNA (orientations were chosen randomly to show values between 0 and 4). Same colors represent same D–A distances. Green arrows represent random orientation. The value of *κ*^2^ (numbers next to the A arrows) depends on the angle between the transition moment of A and the electric field of D at the location of A (*ω*) and the angle between the transition moment of D and the line connecting the centers of D and A (θ). *κ*^2^ = (1 + 3 cos^2 ^*θ*) cos^2 ^*ω*.

As mentioned above, FRET is not only dependent on D–A separation distance and energetic resonance (*i.e.*, D–A spectral overlap), it is also dependent on the orientation of the D emission and A absorption transition dipole moments. In FRET, the D is initially excited and the transition dipole moment (*i.e.*, electric dipole moment associated with the transition from the excited to the ground state) of the oscillating dipole creates an electric field. The three-dimensional orientation of the A defines how well the transition dipole moment of A aligns with the electric field of the D. The orientation factor *κ*^2^ (*cf.*eqn (2), (3), and (13)) can take values between 0 and 4, which correspond to perfect misalignment and perfect alignment, respectively. Van der Meer, arguably one of the most motivated proponents for a correct application of *κ*^2^, uses the analogy of transmitting radio signals in the near-field.^136,152,153^ Not only power (corresponding to D QY and A absorption cross section), distance, and resonance are important but also the orientation of the sender (corresponding to D) and that of the receiver (corresponding to A). Fig. 7 illustrates how the relative orientations of the D and A transition dipole moments influence *κ*^2^ in free space or as attached to DNA.

**Fig. 8:**
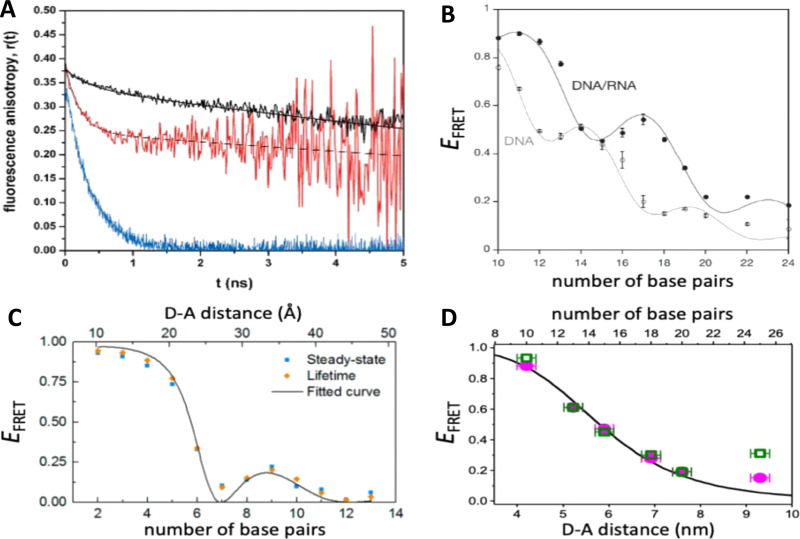
Distance and orientation dependence of FRET. (A) Time-resolved fluorescence anisotropy of free Cy3 (blue: fast rotation), Cy3 attached to dsDNA through a C6 flexible linker (red: fast and slow rotation), and DNA backbone internal modification (black: slow rotation). Reproduced with permission from ref. 154 Copyright 2009 American Chemical Society. (B) *E*_FRET_ for Cy3–Cy5-labeled dsDNA and hybrid RNA/DNA duplexes as a function of duplex length. Due to the rigidly stacked dyes on the duplex ends and the double-helix structure of the duplexes, *E*_FRET_ shows distance (1/*r*_DA_^6^) and orientation (changing *κ*^2^) dependence, resulting in a decreasing “bouncing ball” type curve, which is different for B-form dsDNA and A-form hybrid RNA/DNA. Reproduced with permission from ref. 155 Copyright 2008 by The National Academy of Sciences. (C) DNA with firmly incorporated fluorescent base analogues as D and A shows a similar “bouncing ball” type *E*_FRET_ distance-orientation dependence due to the DNA double-helix structure. Reproduced with permission from ref. 156 Copyright 2017 American Chemical Society. (D) *E*_FRET_ for Tb-Cy5.5-labeled dsDNA, and PL lifetimes of Tb (green open squares) and Cy5.5 (magenta dots) as a function of duplex length. Due to the long excited-state lifetime of Tb (∼2.7 ms) and its isotropic emission, FRET in the DNA double helix is only distance dependent and dynamic averaging with a constant orientation factor (*κ*^2^ = 2/3) becomes applicable. Significant uncertainties become only visible for very long D–A distances beyond 9 nm. Reproduced with permission from ref. 157 Copyright, 2017, John Wiley & Sons.

DNA (or RNA) represents some of the best scaffolding systems to experimentally show the actual influence of D–A orientation on *E*_FRET_. Organic dyes can be attached almost anywhere on DNA and the double-helical structure rotates the dyes while, at the same time, displacing them. Using time-resolved fluorescence anisotropy (*r*(t)) measurements, Levitus and coworkers showed that the rotational freedom of a Cy3 dye in solution can be significantly reduced by attaching it to DNA, see Fig. 8A. Internal DNA-labeling with Cy3 *via* a six-carbon flexible linker significantly slowed down rotation speed, 36% fast and 64% slow decay of *r*(*t*), whereas incorporation into the DNA backbone almost completely rigidified the Cy3 (15% fast and 85% slow decay of *r*(*t*)).^154^ When Cy3 (as D) and Cy5 (as A) were both rigidly incorporated into complementary ssDNA strands and hybridization placed them at different distances and angles to each other, *κ*^2^ values of up to ∼3.5 could be accomplished. The authors also took into account the change of Cy3 QY when it was attached to DNA. QY also directly influences *E*_FRET_ (*via* the Förster distance, eqn (2)) and can therefore become another uncertainty when measuring D–A distances with FRET.

Lilley *et al.* terminally attached Cy3 (D) and Cy5 (A) to the 5′ ends of dsDNA or DNA/RNA duplexes, such that both dyes were predominantly stacked. Thus, the length of the dsDNA would change both the D–A distance and D–A orientation.^155^ Using steady-state fluorescence spectroscopy at the ensemble level and TIRF microscopy for smFRET analysis, they measured Cy3–Cy5 FRET base pair (bp) by bp in 10 to 25 bp DNA duplexes. The results clearly showed *E*_FRET_ dependence on both distance and orientation. *E*_FRET_ decreased proportional to 1/*r*_DA_^6^ but with a very obvious periodical modulation caused by changes to unfavorable dipole orientation as a function of the intrinsic DNA helix rotation, leading to a “bouncing ball” type dependence of *E*_FRET_ with increasing distance (Fig. 8B). Interestingly, *E*_FRET_ measurements could also reveal the different helical geometries of dsDNA (B form) and DNA-RNA (A form), which resulted in distinct “bouncing ball” curves. See also work in the same vein from the Asanuma Group.^111^

Wilhelmsson and coworkers used FRET between two different fluorescent C or A base analogues to also investigate the distance and orientation dependent FRET in DNA.^156,158^ Different ssDNA with a blue (∼420 ± 40 nm) emitting A analogue (qAN1-D) were hybridized to complementary ssDNA with a blue (∼430 ± 40 nm) absorbing analogue (qA_nitro_-A), which resulted in dsDNAs with D–A distances between 2 and 15 bps. Both steady-state and time-resolved fluorescence spectroscopy again revealed the combined distance-orientation dependence of *E*_FRET_ (Fig. 8C), which also confirmed the high affinity incorporation of the fluorescent base analogues into the DNA. Similar to DNA-labeled dyes, qAN1 showed different fluorescence QYs in ssDNA (∼21%) and dsDNA (∼11%). In addition, the different positions of qAN1 in the dsDNA also slightly modified the QY, which also had to be accounted for.

The near-ideal situations just discussed involve optimal combinations of dyes and dye attachments to the DNA. In the opposite limit where the DNA offers little control over dye orientation, it becomes more appropriate to analyze the data with some form of orientation averaging. The most commonly used averaging approach is that of dynamic averaging, for which *κ*^2^ = 2/3.^159^ This value corresponds to D–A systems, in which both D and A rotate freely at a rate that is much faster than the excited-state lifetime of the D. As discussed above, rotation kinetics can be verified by time-resolved fluorescence anisotropy measurements and, depending on the attachment approach, the linker, and the fluorophore, such rotation can be fast, slow, or a mixture or both (*cf.*Fig. 8A). The other averaging regime is referred to as static averaging, for which *κ*^2^ can take values between 0 and 4 when all orientations are present (in an ensemble system) but D and A are hardly rotating during the D's excited-state lifetime.^159,160^ For such averaging, *κ*^2^ depends on the distance, such that it is 0 or close to 0 for short distances, then increases with D–A distance, and finally levels off at a value of 2/3 for large distances. Viable FRET systems can also exist in between the two regimes.^161^

In most well-defined DNA scaffolded systems, *κ*^2^ will either take discrete values that can change with the attachment position in/on the DNA, which can be accounted for by a periodic change of *κ*^2^ with increasing D–A distance as discussed for the “bouncing ball” curve of *E*_FRET_ in Fig. 8B/C, or it can be approximated by the dynamic averaging regime (*κ*^2^ = 2/3). To a good approximation, the dynamic averaging regime can be attained when dye stacking (at the ends of DNA duplexes - *vide supra*) is avoided and flexible linkers are used. In a large multi-lab study, Hellenkamp *et al.* attached Atto and Alexa Fluor dyes *via* C_2_ or C_6_ amino linkers to different internal T bases in DNA and investigated the distance dependence of *E*_FRET_ with intensity-based smFRET.^162^ Fluorescence lifetime and anisotropy measurements were used to verify the QY stability and sufficient mobility (fast rotation) of the dyes at the different positions in the DNA. The estimation of *κ*^2^ = 2/3 introduced an uncertainty of ∼5% to the determination of *R*_0_ and *E*_FRET_ could be determined with standard deviations between ±0.02 and ±0.05. This seminal blind study undertaken by 20 different labs led by the Seidel Group confirmed that the dynamic averaging regime could be successfully applied to such dye-DNA FRET systems. Indeed, Seidel and coworkers have been instrumental in providing guidance on how to choose between static and dynamic dipole averaging assumptions in these configurations.^162,163^

Another possibility to justify dynamic averaging in fluorophore-labeled DNA systems is the use of fluorophores with long luminescence lifetime. In one example, Hildebrandt *et al.* conjugated a Tb complex (D) and a Cy5.5 dye (A) to the 5′ ends of complementary ssDNAs of different lengths. The hybridized dsDNAs with D–A distances ranging between 10 and 25 bps were then investigated by time-resolved fluorescence spectroscopy in solution at low nanomolar DNA concentrations.^157^ Although an orientation dependence of *E*_FRET_ could have been expected due to the 5′ end attachment and possible stacking, the extremely long excited-state lifetime of the Tb complex D (∼2.7 ms, against which even slow molecular rotation will be significantly faster) resulted in a typical 1/*r*_DA_^6^ distance dependence but without the “bouncing ball” type orientation dependence (Fig. 8D).

In summary, although there have been a few demonstrations where purposeful control of dye orientation on DNA was attained,^104–106^ in general, controlling dye orientation on DNA (and other scaffolds) still remains very difficult. Achieving either the dynamic averaging limit or highly oriented dye pairs where *κ*^2^ is known remains crucial for a precise determination of the D–A distance. Further, developing ways to control dye orientations on DNA structures can potentially enable FRET over larger (D–A orientation toward *κ*^2^ = 4) or shorter (D–A orientation toward *κ*^2^ = 0) distances or for on–off FRET switching.

### 3.3. Molecular dynamics and energy transfer modeling

Given the complexity of DNA-organized dye networks, computational modeling can often contribute to improved understanding in two different ways. The first is that it can allow us to better appreciate the reality of the nanostructures at the atomic scale, with the main tool here being atomistic Molecular Dynamics (MD). Second, it can be used to model the energy transfers of interest within the dye networks, both for predicting what can be expected under ideal conditions and to aid in interpreting non-idealities in experimental data.

As with most of nanoscience, physical characterization represents a major challenge when investigating DNA-organized dye networks due to their nanoscopic dimensions and spacings.^20,76,164^ Direct methods such as X-ray diffraction (XRD)^165^ and cryoTEM^18^ are attractive because of their potential for high resolution, but they require highly repeatable structures (with the possible exception of single-shot diffraction of high-intensity free-electron laser pulses),^166^ tend not to look at the native conformations of most interest (*e.g.*, freely rotating in an aqueous environment at room temperature), and generally demand costly in-house expertise and instrumentation. AFM is a more economical tool for direct evaluation, but its two-dimensional nature, sample invasive quality, and limited lateral resolution usually restrict it to no more than providing preliminary assessments. Lastly, the ETs between dyes can themselves serve as an indirect tool for dimensional characterization, with FRET itself being well known for its capacity as a so-called spectroscopic ruler.^131,167^ In fact, in studying biological structures this method has become quite valuable,^163^ especially when based on smFRET, either with diffusing species or in a total internal reflection fluorescence (TIRF) configuration.^168^ But it is important to recognize that the situation of biological structures is rather different from that of the biomimetic dye assemblies of interest here. In particular, in biology the structures are not usually objects to be engineered, but instead are well defined by genetics, *e.g.*, by their amino acid sequence in the most common case of proteins. In addition, mechanistic information is often critical, *e.g.*, tracing the conformational changes in enzymes that are essential to understanding their activity, and this is not generally accessible by XRD or cryoTEM. But FRET is too cumbersome and with too many assumptions to be of much value when doing exploratory engineering of DNA structures for use as scaffolds for large numbers of dyes – though this could change in the future if the artificial structures become more sophisticated and/or dynamical behavior becomes of interest. So instead, we look to numerical modeling for assistance, using MD to supply approximate information at the atomic scale (*vide infra*). This is feasible because of the sophistication of present-day MD and because the sizes of the DNA-organized FRET networks are rarely so large as to make computational analysis too intensive or completely impractical. In the latter case, coarse-grained approaches may be more suited to, at a minimum provide, some useful information. Nevertheless, given the many assumptions and parameterizations of the MD, and especially the uncertainty that comes from incomplete knowledge of the parameters for the dye force field, one needs to be alert to the potential errors.

As for simulating the energy transfers within the dye networks, which model to use depends on the strength of the coupling between the dyes. Simplest is the case of weak coupling where the dipole–dipole interaction dominates and the associated FRET transfers are well described by Förster theory.^117,131^ When dye molecules are at close range the coupling becomes stronger, one goes beyond the FRET regime and the physics becomes much more complex.^169^ This regime requires a more exact treatment of the Coulomb coupling between dye molecules.^170,171^ In addition, effects such as through-bond energy (Dexter) transfer, coherent energy transfer,^172^ charge transfer, vibronic coupling, heat bath effects, *etc.*, some of which are described below, may also become important.

#### 3.3.1. Molecular dynamics simulations of dye position and orientation in DNA-based networks

Any MD simulation of a DNA-based dye network starts with an initial set of atomic coordinates. As illustration, in some of our previous work we used CaDNAno^16^ for creating the DNA structure and UCSF Chimera^102^ for adding the dyes.^103,113,173^ To account for the crucial effect of the surrounding water molecules and counter-ions, we employ an explicit treatment in which the water molecules and counter-ions are distributed initially at random at the desired concentration within a simulation box.^113,174^ This explicit approach increases the number of atoms in the simulation by about an order of magnitude and makes the simulation significantly more costly computationally than the alternative implicit solvent approach. All of the constituents are taken to interact *via* prescribed force fields, and for us the associated models were those of Amber99sb for the DNA,^175^ generalized Amber force field (GAFF)^176^ for the dyes, and transferable intermolecular potential 3P (TIP3P) with a 20 mM MgCl_2_ buffer for the solvent.^177^ Periodic boundary conditions were assumed, and thermostat and barostat conditions were applied in order to maintain the desired macroscopic environment. The MD simulation then proceeded by integrating the Newtonian equations of motion forward in time; in ref. 103, 113 and 173 this was done using Gromacs with a 2 fsec time step.^178^ When run for a ‘sufficient’ enough time it is presumed that the computed history will fully explore the phase space, thereby providing (ergodic hypothesis) a complete *albeit* approximate characterization of the DNA construct's statistical mechanics.

**Fig. 9:**
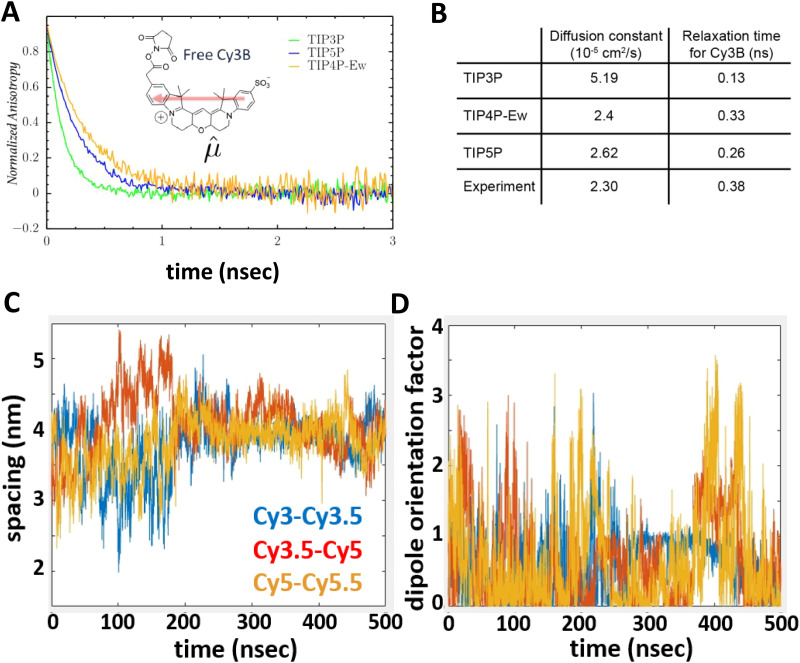
Molecular dynamics (MD) derived results for a free or DNA-bound Cy molecule in solution assuming different water models. (A) Computed transients in the fluorescence anisotropy. (B) MD-derived diffusion constants and relaxation times compared with experimental values. Reproduced with permission from ref. 113 Copyright 2020 American Chemical Society. MD-derived histories for the spacing (C) and dipole orientation factor (D) of the nearest-neighbor dye pairs in a linear DNA construct labeled with four dyes, see Fig. 22 for a schematic of the structure. Reproduced with permission from ref. 173 Copyright, 2021, John Wiley & Sons.

To underscore the importance for physical fidelity of using well-tested models in an MD simulation, we show results in Fig. 9,^113^ in which the fluorescence anisotropy of a free Cy3B dye was simulated using various models for the water.^131,177^ The anisotropy of a free dye relaxes *via* rotational diffusion of the dye and so it is not surprising to see that the MD-simulated anisotropy decay is sensitive to the choice of the water model. From the comparative data listed in Fig. 9B it is evident that some models give much better agreement with experiment than others do. This is an important point to consider, as these types of analyses are not trivial. As a second illustrative example, we discuss the simulation of a linear arrangement of four dyes (Cy3, Cy3.5, Cy5, Cy5.5) with each dye attached at the 3′ end of a constituent DNA oligo of a DX crossover.^173,179^ See Fig. 22 for a schematic of the structure and its related text for a discussion of the FRET characteristics observed in that configuration.^173^ MD-derived histories of the spacing and *κ*^2^ parameter for each pair of nearest-neighbor dyes are given in Fig. 9C and D where we recall that *κ*^2^ depends on the relative dipole angles according to:^117,131^

13 *κ*^2^ = (cos *θ*_T_ − 3 cos *θ*_D_ cos *θ*_A_)^2^

where *θ*_T_ is the angle between the transition dipoles of the two dyes and *θ*_D_ and *θ*_A_ are the angles between the dipoles and the vector joining the two dyes (eqn (13) is an alternative form of eqn (3)). While the results in Fig. 9C and D are representative of what is often seen in such simulations, it is important to note that one sometimes observes step-like “jumps” in the transients even at long times (*e.g.*, associated with reversals in the orientation of a given dye's linker) that leaves the question of whether the simulation has been run long enough to capture its full behavior. In any event, most prominent in Fig. 9C are the initial transients in the dye spacings of about 200 ns duration that presumably represent the equilibration of the structure from its arbitrary initial state. In contrast, the *κ*^2^ plot in Fig. 9D shows no clear sign of an initial transient, with strong fluctuations seen throughout the simulation. This suggests that this particular DNA scaffold (with its dye attachments) is not effective at constraining the dipole orientations of the dyes.

**Fig. 10:**
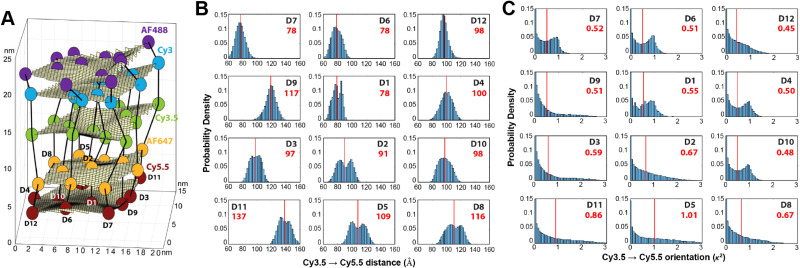
Molecular dynamic (MD) analysis of a DNA block scaffolded multi-dye structure. (A) Dye positions as estimated from the MD simulation with the small spheres representing the dyes. In the ideal crystal the planes would be flat and the vertical lines (that approximate every other constituent duplexes in the block) would be straight. The axes dimensions are in nm. See Fig. 27 for a schematic of the structure. MD estimated distribution of (B) distances and (C) dipole orientation factor between a central Cy3.5 and the plane of Cy5.5 dyes. Reproduced with permission from ref. 103 Copyright 2021 American Chemical Society.

As a third illustration of MD analysis, we summarize the modeling aspects from a FRET network assembled in a DNA brick based architecture that arranged planes of D–A dyes next to each other as a testbed for systematically investigating FRET in the sheet regime (Fig. 10). See also Fig. 27 and related text for a description.^103^ Interestingly, this example is near the limit of practicality for all-atom MD. As shown in Fig. 27A, the DNA block nanostructure approximates a rectangular prism with dimensions of about 10 × 15 × 40 nm^3^, and in its maximal configuration it can host 60 dyes of 5 different types (*viz.*, AF488, Cy3, Cy3.5, AF647, and Cy5.5) that are respectively organized into 5 sheets. Experiments ranged the full gamut of dye loading ratios including for cases where the dye networks consisted of just two dye types arrayed in a pair of quasi-parallel sheets (with up to 12 dyes per sheet). The MD simulation of the DNA structure, shown in Fig. 10A, and its surrounding solvent was quite computationally intensive and this limited the total time for which the system could be interrogated. Whether this duration was sufficient to capture the slowest fluctuations of the structure, *e.g.*, associated with the AF647 dye that had an especially long linker, is not known (see chemical structure in Fig. 6F). Greatly magnifying the computational needs was the fact that we wished to study many different versions of the same structure hosting various different dye arrangements. To reduce the associated computer time/cost, two simplifications were therefore introduced in the model. First, weak harmonic springs were attached to a few outer P atoms in order to prevent the entire DNA structure from rotating, thereby allowing the simulation box to be smaller without introducing spurious boundary effects. Second, instead of multiple MD simulations of the full structure, only a single one was performed with one copy of each different dye assembled along the central DNA helix of the rectangular prism block (as shown in Fig. 27B), and then results for all other dye configurations were generated by extrapolation. This was done by relying on the one simulation to give the history of the attachment point of each dye linker to the DNA block, and then adding in the motion of the corresponding centerline dye relative to its attachment point (at a random time so there are no spurious correlations) and rotated to match the local orientation. The main and hopefully small error made by this scheme is that the perturbing effect of having the non-centerline dyes (as shown in Fig. 27C) on the DNA motion is neglected. With these shortcuts, the single simulation that was run involved approximately 900 000 atoms and to simulate 1 μsec of real time required roughly one month on a large CPU cluster.^103^

Using these procedures, the MD-estimated average positions of the dyes in the DNA block structure with its full complement of 60 dyes shown in of Fig. 10A. As is evident from the figure, while the scaffold preserves the basic arrangement, large deviations from regular order are seen. Of more interest from the perspective of FRET is the degree to which the DNA scaffold controls the interdye spacings and relative dipole orientations. As an example of such a calculation, data for the distributions of these quantities are shown in Fig. 10B and C for the case of a single Cy3.5 dye situated in front of a “sheet” of 12 Cy5.5 dyes, labeled D1 to D12, and with the Cy3.5 placed directly opposite dye D1. The vertical red lines in these plots give the ensemble averages of the Cy3.5–Cy5.5 spacings and the *κ*^2^ value. As seen, the distributions of the spacings, while not ideal, are fairly tight (roughly ± 1 nm) and the orientational distributions are clearly not random because of dye-DNA interactions. These estimates argue that the structure is indeed organizing the dyes to a substantial degree. However, while the interdye spacings generally grow as one moves away from the center (D1) as expected, the particular dyes D6 and D7 are not in their proper positions, instead being roughly the same average distances from the Cy3.5 dye as the D1 dye. As a result, they can be expected to make a larger than expected contribution to potential FRET processes.

**Fig. 11:**
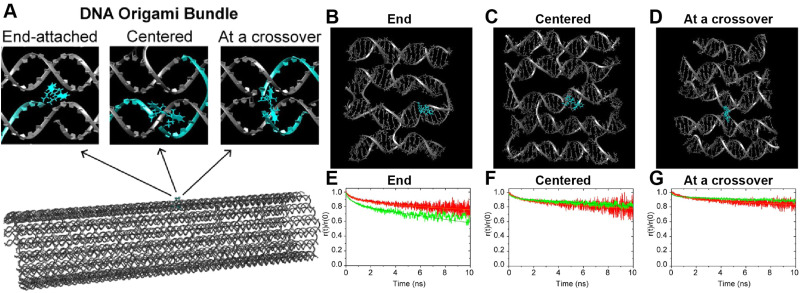
MD analysis of dye orientation in a DNA origami bundle. (A) Schematic of the DNA bundle (grey) with one staple strand (cyan) including a dye in three configurations, namely, End-attached (*A*_bundle_), Centered (*E*_bundle_), and At a crossover (*F*_bundle_). (B)–(D) Snapshots of the local bundle structures (*A*_bundle_, *E*_bundle_ and, *F*_bundle_, respectively) from the MD simulations. The position of the Cy3 molecule is shown in cyan color. (E)–(G) Comparison of simulated (green curves) and experimental (red curves) normalized time-resolved anisotropy for *A*_bundle_, *E*_bundle_, and *F*_bundle_. Solid curves are fits to the data. Reproduced with permission from ref. 113 Copyright 2021 American Chemical Society.

A further illustration of MD analysis considers an example wherein experiment and simulation were used to examine in more detail the ability of a DNA structure to control dye position and orientation, this time using a stiffer origami-based 30-helix DNA bundle as the structure (Fig. 11).^113^ Here, a single Cy3 dye is incorporated into the structure, and the focus was on comparing how constrained the dye was when attached at various locations and using different conjugation chemistries. Experimentally the Cy3 local environment and rotational flexibility were probed using lifetime and anisotropy measurements for three different attachment locations including the “End-attached” or *A*_bundle_: Cy3 replaces a 5′ end base (and attached *via* a single phosphodiester linkage); “Centered” or *E*_bundle_: Cy3 replaces an internal base away from a crossover; “At a crossover” or *F*_bundle_: replaces an internal base at a crossover (and attached *via* two phosphodiester links in the latter two cases), see Fig. 11A. Comparison of the MD computed (Fig. 11B–D) anisotropy transients for the fast decay associated with local dye motion and that of a slow decay associated with collective rotation of the entire construct showed good correspondence to that observed experimentally with some variation in the associated time constants (Fig. 11E–G).

**Fig. 12:**
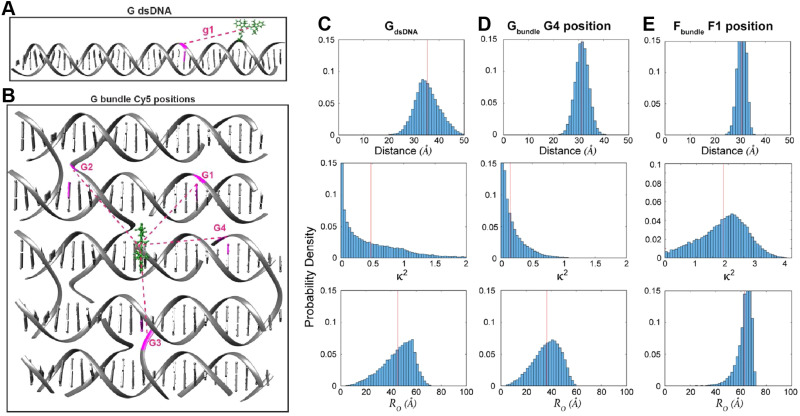
MD-derived dye distance and orientation probability distributions. (A) A linear duplex and (B) DNA bundle shown in Fig. 11 were compared for the dye spacings, dipole orientation factors and *R*_0_ values for Cy3–Cy5 pairs. The Cy3 position is shown in green whereas Cy5 was simulated in particular locations namely g1 in the “G dsDNA” duplex and G1 through G4 sites in the DNA bundle fragment shown in (B). The inter-dye distance, orientation factor, and Förster radius change is shown for (C) g1 site, (D) G4 site, and (E) F1 site (the structure is not shown here). Reproduced with permission from ref. 113 Copyright 2021 American Chemical Society.

Control of the dye locations and dipole orientations is important for raising the efficiency of synthetic light harvesting (LH) structures operating in the FRET regime, and it becomes essential if one hopes to access quantum coherent behaviors seen in biological analogs.^180^ As a study in the FRET context, we artificially created a Cy3–Cy5 FRET pair by generating an MD history for an artificial Cy5 dye using the same method discussed in association with Fig. 10. In this study we used the DNA bundle shown in Fig. 11; three examples of results appear in Fig. 12 where we have plotted the MD-calculated distributions of the interdye spacings (top panel in Fig. 12C–E), *κ*^2^ (middle panel), and the *R*_0_ (bottom panel).^117,131^Fig. 12D (from a site illustrated in Fig. 12B within what the authors refer to as the *G*_bundle_) is information for a case where the Cy3 and Cy5 are inserted (each as a double phosphodiester linkage to the DNA backbone) between bases at the crossover, whereas the case in the right column (derived from Fig. 11 crossover design called the *F*_bundle_) has the dyes replacing a base at the crossover. Fig. 12A and C (*G*_dsDNA_) is a control where the dyes are instead attached between bases of a dsDNA. Clearly, these differences in the dye attachments have major consequences for the behavior. First, using the bundle as the scaffold rather than the dsDNA obviously results in much less variation in the distribution of the interdye spacing. Also, the plots of the *κ*^2^ distribution show that the dipole orientation for the dsDNA is much more random than those for the bundle cases, with the *G*_bundle_ distribution skewed toward lower values (because the two dipoles tend to be held in an unfavorable orientation) whereas the *F*_bundle_ favors higher values (because the dipoles tend to be favorably oriented). These differences result in the *R*_0_ of the *G*_bundle_ not only being smaller than that for the *F*_bundle_, but even for *G*_dsDNA_, and they highlight the fact that in controlling the relative dipole orientations, we also want the angles to be in a favorable relationship so that the *κ*^2^ values will be elevated. Overall, these MD simulations demonstrate that DNA scaffolding does have the potential to provide optimal dye orientations if the attachment points can be well chosen. However, much further work will be needed to fully elucidate this capability and to show that it can allow biological performance levels to be realized in artificial structures.^180^

#### 3.3.2. FRET simulations – ideal case

For simulating ETs in the Förster regime, the chromophores are treated pairwise with the transition rate associated with the dipole–dipole interaction given by eqn (1). For arbitrary distributions of dyes with known locations and dipole orientations, one analyzes the energy propagation from initial excitation to final emission by solving (usually numerically) coupled rate equations that include all possible transitions. The variables in these equations are probabilities of local excitation and, if integrated over time, they produce an algebra whose solution can be used to estimate ET efficiency quantities such as *E*_ae_ (eqn (9)) and *E*_ee_ (eqn (10)).

We term such FRET simulations “ideal” when the values of the dye positions and *κ*^2^ are assumed *a priori* rather than being obtained either from experiments or from MD simulations. These ideal simulations are deemed useful as a way of providing a basic projection of the behavior to be expected from a given design. In a typical treatment, the dye attachment points are assumed to be those dictated by an idealized, well-ordered scaffold design (*e.g.*, as laid out in CaDNAno, see for example Fig. 27A), and then the dyes are located either at these precise positions or displaced by some random distance and direction as permitted by a flexible linker with possible steric restrictions.^181^ The dipole orientations are generally taken to be randomly distributed in orientation and treated in either of two tractable limits. As mentioned previously, the first “dynamic” limit assumes the dipoles re-orient rapidly on the time scale of the energy transfer(s) so that the average value can be used in computing the FRET. In this case, the dipole orientation factor *κ*^2^ takes on the well-known constant value of 2/3, thus simplifying the calculations.^117,131^ The “static” limit assumes the opposite case in which the dipoles reorient slowly on the time scale of the energy transfers. As a result, at any given time each dipole will have a set orientation and the relative orientations of each pair of dyes will determine the value of *κ*^2^ at that instant for that pair.^117,118,136,160,181^ Implemented in a Monte Carlo procedure, *E*_FRET_ values for many such instantiations are then computed, each with a different set of random dipole orientations, and then by averaging over the ensemble we obtain the ideal *E*_FRET_ for that structure in the static limit. The dynamic limit is very often assumed for its simplicity, however, in most cases the static limit provides a better representation of reality as can be demonstrated with anisotropy measurements.^103^

**Fig. 13:**
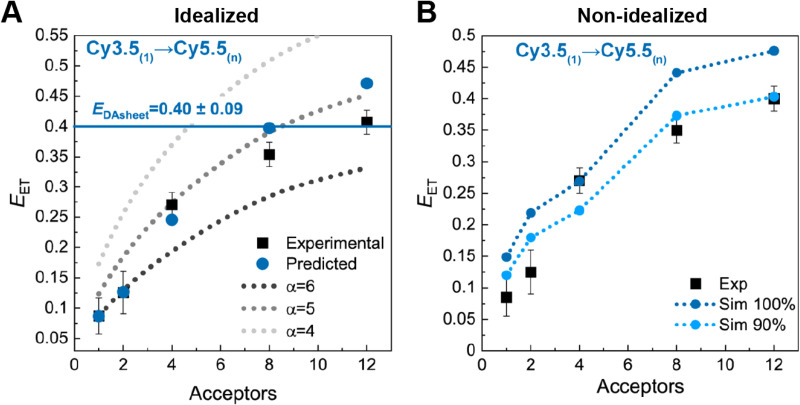
Ideal and non-ideal FRET simulations. (A) Experimental FRET efficiencies for transfer from a single Cy3.5 to a variable number of Cy5.5s arrayed in a plane compared with ideal simulations assuming dynamic dipoles. For the simulations the distance between the Cy3.5 and the plane is assumed to be 8.6 nm, while the distances between Cy5.5s is 5.6 nm. (B) Non-ideal simulations using the MD-derived spacings and orientations. One calculation assumes all dyes are present (100% Sim) while the other assumes a DNA structural assembly yield of 90%. See Fig. 27 for a schematic of the structure from which this data is derived. Reprinted with permission from ref. 103 Copyright 2021 American Chemical Society.

Results of some ideal FRET simulations are shown in Fig. 13A using again the structure described below in Fig. 27 and for the case of a single Cy3.5 dye positioned in front of various numbers of Cy5.5 dyes arrayed in a “sheet” configuration (as was analyzed using MD in Fig. 10). Shown in Fig. 13A are experimental results for how the *E*_FRET_ rises as more Cy5.5 acceptors are added to the scaffold. For performing the corresponding ideal FRET simulations we assume the distance between the Cy3.5 dye and the adjacent Cy5.5 dye is 8.6 nm, while the spacings between Cy5.5 dyes in the sheet are assumed to be a regular 5.6 nm. As seen, the ideal *E*_FRET_ values calculated do not compare well with the experiment for which the efficiency is seen to rise much faster as Cy5.5 dyes are added. The poor agreement suggests that one or more of the assumptions of the ideal case are substantially in error, which is not surprising given the positional discrepancies seen when one compares the ideal geometry in Fig. 27A with the MD-simulated average dye positions of Fig. 10A.

#### 3.3.3. FRET simulations – non-ideal case

Non-ideal FRET simulations carry out the same computation as in the ideal case except that they attempt to incorporate information on the actual distribution of dye positions and dipole orientations as determined/constrained by the DNA scaffold and by the linker chemistries. As noted above, this information cannot be fully derived at present from experimental methods, and therefore one can resort to MD simulations for insight. Taking the MD history to provide a full sampling of the phase space, one can then introduce this information into a Monte Carlo simulation in a static dipole approximation. That is, one could randomly select a time in the MD transient and use the positions and orientations of all the dyes at that instant to compute an *E*_FRET_. After enough such samples have been gathered, *E*_FRET_ in the static limit is obtained by an ensemble average. Results from such calculations for the structure of Fig. 27A (and with certain simplifying assumptions as noted earlier) are shown in Fig. 13B along with the same experimental dataset as that used in Fig. 13A. The estimates now rise much more quickly as the number of added Cy5.5 dyes increases. The main reason is the same as that noted above, namely that two of the Cy5.5 dyes being added are much closer to the Cy3.5 than the design suggested they should be. The MD-computed *E*_FRET_ (indigo curve) are in fact higher than experiment, and this can plausibly be explained as the result of non-ideal formation yield of the entire DNA–dye structure. Experimentally we estimated the number of missing or inactive dyes to be no smaller than about 10%, and so a second simulation curve computed by assuming that any particular dye is present with a probability of 90% is also plotted. This result (blue curve) agrees quite well with experiment and thus argues in favor of this explanation.^103^ The interested reader is referred to several other more technical treatises for in-depth discussions of various approaches to non-ideal simulation.^117,163,168,182–185^

### 3.4. Exciton delocalization

Molecular dye aggregates can form delocalized collective excitations, shared in a wave-like manner, known as molecular or Frenkel excitons that exhibit unique optical properties. They are observed in natural light-harvesting systems and are fundamental to a number of potential applications of interest including artificial light harvesting (LH),^186–191^ organic optoelectronics,^192^ sensing,^193–195^ nanoscale computing,^196–201^ room temperature quantum entanglement,^202,203^ and room temperature quantum computing.^202–204^

Based on the work of Childs *et al.*,^205^ several of the authors of this publication (Yurke and Knowlton) have postulated that certain exciton parameters describing dye aggregate behavior provide the necessary means to establish room temperature quantum computing.^206,207^ DNA nanotechnology provides a means to organize the requisite dyes with precise control into a variety of dye aggregate architectures that allow for the specific tuning of their unique optical properties as pointed out in previous sections. In this section covering exciton delocalization, molecular or Frenkel exciton theory is described using the insight from Kasha and coworkers,^208–214^ and others.

#### 3.4.1. Frenkel molecular exciton theory and key parameters

Frenkel Exciton Theory, otherwise known as Molecular Exciton Theory, describes exciton delocalization and exciton–exciton interaction in molecular aggregates. We describe the key parameters present in the Frenkel Exciton model that include *J* (excitonic hopping parameter), which is based on the transition dipole moment (*μ*) of each dye and describes the delocalization of a single exciton in a wave-like manner over a DNA-templated aggregate; the less known, yet fundamentally crucial, *K* (biexciton interaction energy), which is induced solely by the difference in static dipole moment (Δ*d*) of each dye; and *Δ*, the single site exciton–exciton interaction energy (interaction energy of two excitons both on one site). The reader should note that the ‘*J’* here is distinct from that of the *J* designating the FRET overlap integral above, and is typically utilized in subsequent context as ‘*J*_*m*,*n*_’ where the subscripts denote dye positions (see below). Because excitons have bosonic properties, they can be described as either hard core (*i.e.*, two excitons cannot reside on the same dye) or soft-core bosons (*i.e.*, two excitons can reside on the same dye).^215–217^ The aim of this section is to describe the details of *J*, *K*, and *Δ* and highlight their potential utility within the context of this discussion.

##### 3.4.1.1 Single-molecule energy eigenstates

Here, we consider an aggregate comprised of *N* three-energy level dyes in which only the interactions between pairs of dyes are allowed (*i.e.*, between the dye at site *m* and the dye at site *n*).^216,218^ The single dye energy eigenstates are represented by three wave functions (Table 1): (1) the ground state wave function here taken to be a singlet state, *ϕ*^(g)^_*m*_, (2) the singly excited state wave function, here taken to be the lowest singlet state, *ϕ*^(e)^_*m*_, and (3) the double-excited state wave function, here taken to be the next higher singlet state, *ϕ*^(f)^_*m*_ where *m* denotes the dye at site *m*. Invoking the Heitler–London approximation,^214,216^ the ground state of the aggregate is given by a direct product of the ground states of all dyes, *Φ*_g_, and the single-exciton basis is constructed by replacing one of the dye ground states by its excited state and is given by *Φ*_e*m*_. The wave functions are schematically depicted in Table 1.

**Table 1:** Three level site basis states

	Ground state	Single-excited dye	Two dye singly excited	Doubly excited dye
Wave function:	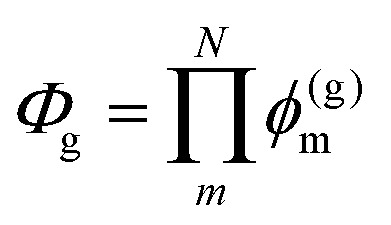	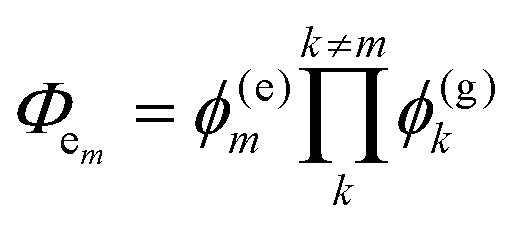	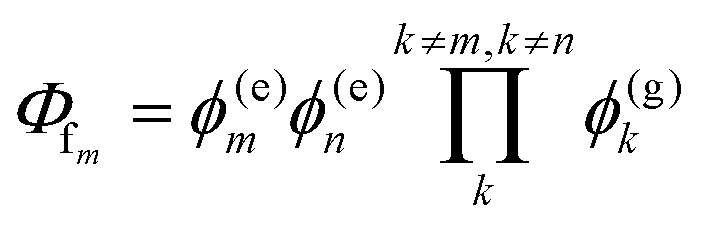	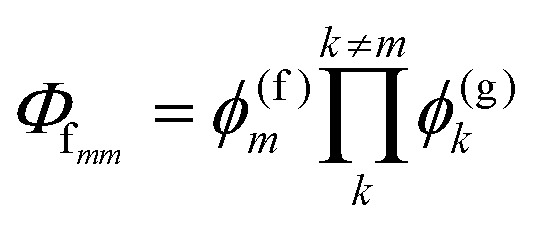
Ground state and creation operators:	*Φ*_g_	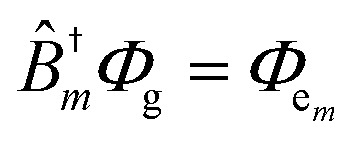	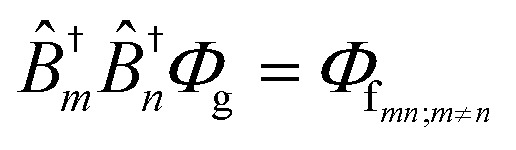	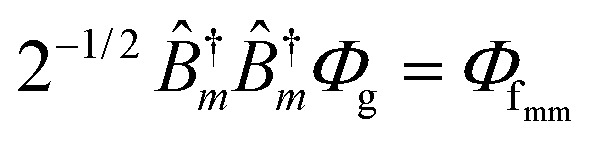

##### 3.4.1.2 Multi-exciton Hilbert space

Note that exciton states that exhibit exciton delocalization are created by the interaction of two (or more) strongly coupled dyes resulting in excited states that are split (*i.e.*, Davydov splitting) as compared to the monomer state (see schematics in Table 2).^208–214^ Focusing on dye dimer aggregates, the exciton states can be described by two-exciton basis states that come in two forms: a dye (*m*) that is doubly excited (*Φ*_f*mm*_), and two dyes (*m* & *n*) that are singly excited (*Φ*_f*mn*_), which are shown in Table 1. This model contains *N* singly excited states and *M* = *N*(*N* + 1)/2 doubly excited states. An approximation is often made for the states where a dye molecule that is doubly excited is excluded. This is the hard-core boson approximation (*i.e.*, two excitons cannot reside on the same dye). Inclusion of these states, as done here, constitutes the soft-boson approximation (*i.e.*, two excitons can reside on the same dye).^215,216^ Schematics of the exciton energy states are also shown in Table 2.

**Table 2:** Frenkel exciton Hamiltonian energies and exciton energy schematics

	Excited monomer	Single-excited dye	Two dye singly excited	Doubly excited dye
Schematic of exciton states in dye m or dyes m & n or m or n:	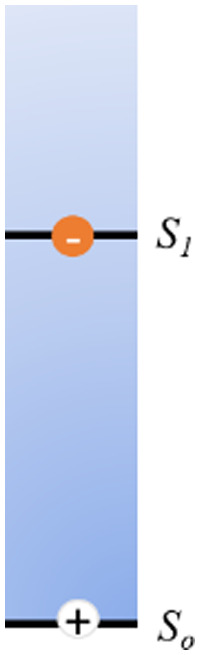	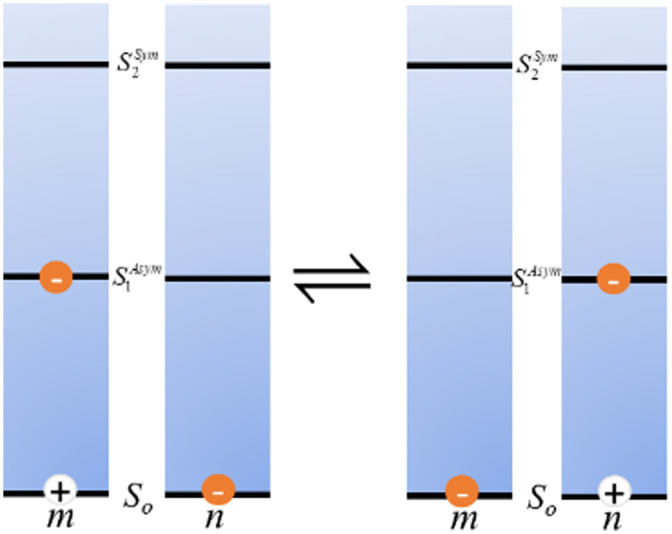	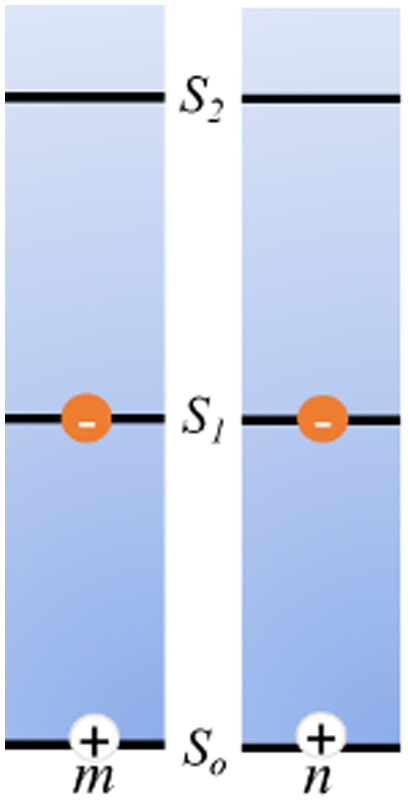	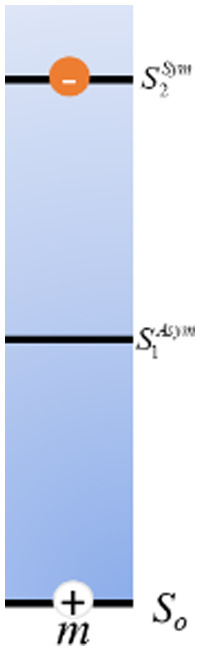
Key parameters:	*ε*_*m*_	*J*_m,n_, *μ*_*m*_, *μ*_*n*_	*K*_*m*,*n*_, Δ*d*_*m*_, Δ*d*_*n*_	*Δ*_*m*_
Key parameter of the exciton Hamiltonian:	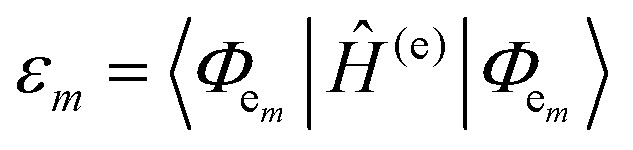	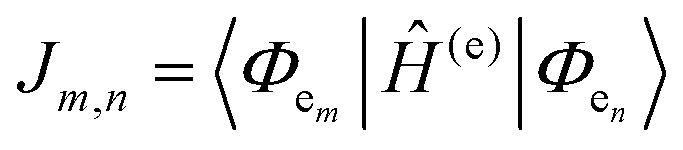	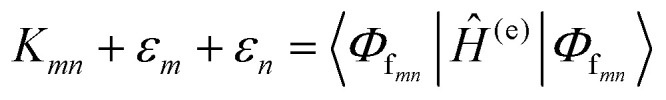	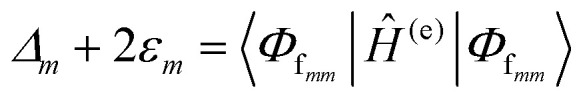

##### 3.4.1.3 Frenkel Hamiltonian governing a system of Frenkel (molecular) excitons

The behavior of Frenkel excitons is well approximated by an augmented Frenkel Hamiltonian;^215–221^ this includes the excitonic hopping parameter *J*_*m*,*n*_ (*i.e.*, a single exciton transition dipole–dipole coupling between dyes on sites *m* and *n*) leading to resonant exciton hopping,^222^ and the exciton–exciton interaction energies *K*_*m,n*_ (interaction energy of two excitons, one each on dye sites *m* and *n*). An augmented Frenkel Hamiltonian of this form is given by:14
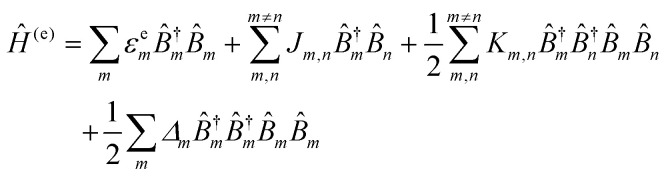
where *ε*^e^_*m*_ is the monomer transition energy (see ref. 220) of a single excited dye (excited monomer: S_0_ → S_1_) on the *m* site, *Δ*_*m*_ is single site exciton–exciton interaction energy (interaction energy of two excitons both on site *m*), and *B̂*^†^_*m*_*B̂*_*m*_ are the bosonic exciton creation and annihilation operators, respectively, on site *m*.

##### 3.4.1.4 Dipole approximation, definitions of *J*_*m*,*n*_, *K*_*m,n*_, and Δ*d*

When intermolecular distances are greater than dye size, the dipole approximation for molecular charge densities can be further invoked such that the *J*_*m*,*n*_ and *K*_*m*,*n*_ couplings can be expressed in dipole–dipole interaction form.^215–217,223^*J*_*m*,*n*_ becomes the intermolecular dipole–dipole interaction between the molecular transition dipoles, *μ*_*m*_ and *μ*_*n*_, for the dyes at sites *m* and *n*, which is given by:15
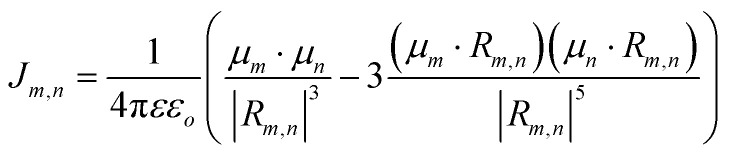
where *R*_*m*,*n*_ is the vector connecting dyes at sites *m* and *n*. *K*_*m*,*n*_ involves the difference between the excited state and ground state static (*i.e.*, permanent) dipoles, Δ*d*_*m*_ and Δ*d*_*n*_, also known as the difference dipoles, for the dyes at site *m* and *n*, and is given by:16
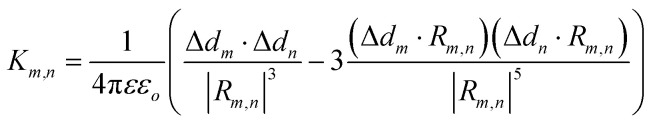
and thus *K*_*m*,*n*_ is zero for a molecule without a static (*i.e.*, permanent) difference dipole moment (*e.g.*, a symmetric dye, Δ*d* = 0). Although eqn (15) and (16) provide straightforward expressions to give an indication of the parameters on which they depend, for intermolecular distances that are less than the dye size (*i.e.*, the dipole–dipole approximation fails), more accurate expressions for *J*_*m*,*n*_ and *K*_*m*,*n*_ are necessary such as the extended dipole approximation.^171,224^

##### 3.4.1.5 The Hamiltonian for vibrons

**Fig. 14:**
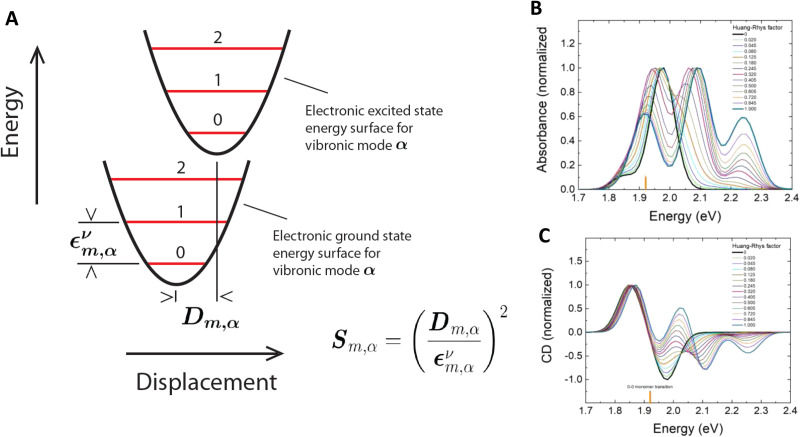
KRM Model Simulations. (A) Graphical representation of the quantities *ε*^v^_*m*,*α*_ and *D*_*m,α*_ for vibronic mode *α* of dye *m*. *ε*^v^_*m*,*α*_ and *D*_*m,α*_ are extracted from optical data using the KRM Model Simulation Tool mentioned in the subsequent sections on exciton delocalization. Calculated (B) normalized absorbance spectra for increasing H-R factors starting from zero and (C) normalized CD spectra. Orientations and monomer properties held fixed. The vertical orange stick represents the 0-0 monomer transition energy of 1.92 eV. Unless the absorbance and CD are not perfectly electronic in nature (black curves) some degree of vibronic coupling is involved. Reprinted with permission from ref. 224 Copyright 2021 American Chemical Society.

The augmented Frenkel Hamiltonian expression (eqn (14)) describes only the electronic aspects of excitons and thus does not include the coupling effects of the vibronic quanta (*i.e.*, vibrons) to the excitons of the system. The Hamiltonian for the vibrons, using a Holstein-like version,^225^ and their coupling to the excitons can be expressed as:17
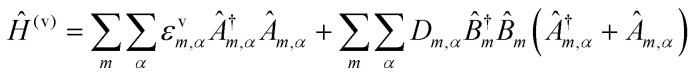
where *Â*^†^_*m*,*α*_ is the vibron creation operator, *Â*_*m*,*α*_ is the vibron annihilation operator, *ε*^v^_*m*,*α*_ is the corresponding vibron energy, and *D*_*m*,*α*_ is the displacement parameter (units of energy) between the electronic ground state and the electronic excited state harmonic oscillator potentials, which are all for vibronic mode *α* on the dye at site *m*.^226^ The meaning of *ε*^v^_*m*,*α*_ and *D*_*m*,*α*_ is illustrated schematically in Fig. 14A where *ε*^v^_*m*,*α*_ is the difference in energy between two neighboring vibronic energy states while *D*_*m*,*α*_ is the difference between the electronic ground and excited state minimums. The square of the ratio of *D*_*m*,*α*_ to *ε*^v^_*m*,*α*_ is described by the Huang–Rhys factor, *S*_*m,n*_.^227–229^ Each vibration mode *α* has its own Huang–Rhys factor *S*_*m*_. The formula for the Huang–Rhys factor for mode *α* on site *m* is expressed as:

18 *S*_*m*,*α*_ = (*D*_*m*,*α*_/*ε*^v^_*m*,*α*_)^2^

It is useful to note that Huang–Rhys factors can be obtained from femtosecond vibrational coherence measurements.^230^ Including both the electronic and vibronic parts of the Hamiltonian by summing eqn (14) and (17) yields an augmented Frenkel Hamiltonian, or Frenkel–Holstein Hamiltonian, and is given by:19
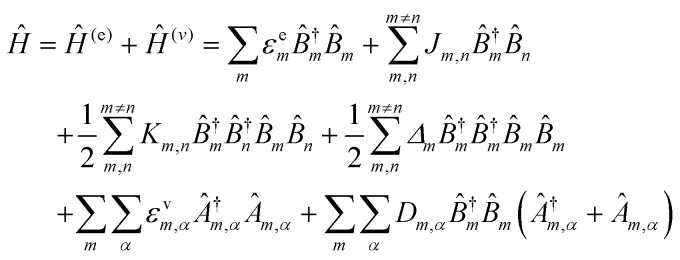
Experimental examples of excitonic interactions in DNA–dye aggregates are described further below in sections after the descriptions of FRET-based studies.

A detailed report by Roy *et al.*^224^ clearly shows the value of including the vibronic part of the Hamiltonian when simultaneously fitting absorbance and CD data to extract useful parameters including *J*_*m*,*n*_, *ε*^v^_*m*,*α*_, and *D*_*m,α*_ (*i.e.*, includes the Huang–Rhys factor, *S*_*m*,*α*_ shown in eqn (18)) and dye orientation. They modeled Cy5 dyes organized into dimers using duplex DNA and BNA and extracted these parameters and dye orientation (explained in detail below). To examine influence of vibronic coupling to the electronic states, they then varied *S*_*m*,*α*_ (from 0 to 1) keeping the extracted orientation constant to show the impact of vibronic coupling on the absorbance and CD data. If only considering the electronic part of the Hamiltonian in eqn (19) for a dimer where *S*_*m*,*α*_ = 0, one would expect to see only two peaks in the absorbance data and the CD data. This electronic coupling case is shown by the black curves in Fig. 14B and C where Davydov splitting is observed in the absorbance and the CD data shows a perfect couplet. In practice, this purely electronic case is rarely observed in dimer absorbance and CD data. Typically, multiple peaks are observed due to vibronic coupling which requires one to vary *S*_*m*,*α*_ between 0 and 1 in order to describe the data. In general, the electronic peaks in the absorbance and CD spectra shift in energy and additional peaks form. The interested reader is directed to Roy *et al.*^224^ for more details.

## 4. Programmable DNA-based optical breadboards

### 4.1. Linear DNA structures and introduction of the DNA photonic wire

An early but important influence on the development of these materials traces to the work of the Mathies’ group at U.C. Berkeley in the 1990s. In their work, a common D dye was coupled to one of 4 potential A-dyes using a pre-optimized spacing created with sugar phosphates on an ET ‘cassette’ and then appended to DNA primers; this allowed for simplified 4-color fluorescent DNA sequencing using a single laser excitation source rather than requiring multiple laser lines to excite spectrally separated dyes.^233,234^ ET cassettes were also incorporated into chain-terminating dideoxynucleotides to act as specific base labels for classical Sanger DNA sequencing.^235^ More pertinently, the ET cassettes confirmed that multiple different dyes could be incorporated into DNA for FRET using common DNA synthesis chemistry. Ju extended this concept in 2001 to combinatorial FRET tags (CFETs) with 3 dyes and 2 ET steps highlighting how more complex spectral signatures could be used for deeper coding to expand the set of multicolor labels available for bioassays; the code consisted of each individual dye's fluorescent contribution within the different emission channels that are monitored during an assay.^236^ Altering the number of dideoxyribose phosphate spacers that separated a given D–A pair in the 3-dye CFET configuration tuned the ET efficiency and changed the fluorescent contributions of a given D–A dye to their assigned optical channels thus yielding a different optical code. Further tuning of ET in this configuration provided for 3 ET steps by allowing the initial D to interact with its proximal A – intermediary relay dye – and also directly with the distally-located terminal A.^237^ Overall, development of organic dyes for incorporation into fluorescence-based DNA assays and their widespread use during the genomic revolution (*e.g.* for DNA sequencing, single nucleotide polymorphism determination, and haplotyping assays) around the turn of the century contributed significantly to the later availability of DNA-incorporating dye chemistries.^235,238^

Table 3 lists some representative FRET configurations prototyped in linear DNA structures. Most of these initial studies utilized ds linear DNA configurations where ss DNAs were concatenated together into a duplex structure due to the inherent design simplicity of this approach. These studies can be considered to be primarily of an exploratory nature as they evaluate what a given DNA structure's D/A dye combination was capable of accessing in terms of FRET. Although not a comprehensive list, these publications reflect some key lessons learned that would later contribute to improving ET in far more complex DNA assemblies. In one initial example, Kawahara *et al.* found that longer range ET on a dsDNA 25 mer (∼8 nm length) could be improved *ca.* 1.5×, from 10 to 16%, by inserting a fluorinated carboxyfluorescein intermediary or relay dye between an initial end-labeled D-fluorescein and an A-rhodamine located at the other end of the DNA duplex.^239^ Ohya achieved ET efficiencies of 20–25% through a 3-dye Eosin_D_ → TAMRA_Relay_ → Texas Red_A_ (→ = ET step) construct where the dyes were placed at 10 bp spacing (equivalent to 1 helical turn of B-DNA) between each other while the DNA sequences themselves incorporated more A and T bases near the dyes to prevent G–C induced quenching.^240^ The 10 bp spacing was intentionally meant to orient the dyes on the same side of the helix at a *ca.* 3.4 nm spacing that corresponded to ≤*R*_0_. Interestingly, FRET theory is agnostic as to what is placed between a D–A dye pair unless it exerts an influence on the ET process by screening the dipoles, *e.g.*, by a nearby metal surface or some type of magnetic material.^4^ More importantly, Ohya *et al.* demonstrated an extended length 4-dye system where 2 TAMRA relays were placed between the D–A, which increased ET efficiency from 8 up to 20% when going from a single to a double relay.^240^ This double relay dye presumably allowed for a homoFRET step between identical D–A dyes to contribute to ET efficiency. The benefits and liabilities of incorporating homoFRET process(es) into these structures would be extensively investigated later within far more complex DNA scaffolds (*vide infra*). It is also worth noting that Ohya's example would become very influential for many of the formats tested later as it highlighted the plasticity of DNA sequences, the ability to intimately control D–A spacing to better match putative *R*_0_ values, and the ease with which dyes could be inserted or removed from a given structure just by changing the sequence used in the assembly reaction.

**Table 3:** Select characteristics of representative multi-FRET linear photonic wires

DNA structure (estimated length)	# of ET steps	Fluorophores/D → A	*r*_DA_ a	*E*_ee_	Notes	Ref.
25 bp ds triplex (∼8 nm)	2	3/6-Carboxy Fl, hexachloro-6-carboxy FL, 6-carboxy-X-rhodamine	—	∼16%	Relay dye increases *E*_ee_ from 10% to 16%	239
30 & 40 bp ds duplex (∼8 nm)	3 (+ 1 homoFRET)	3/Eosin, TAMRA, Texas Red	10 bp < 1 × *R*_0_ for each D–A pair	20%	10 bp (1 helix) separates dyes. *E*_ee_ increase 8% to 20% for 2xTAMRA relay on 40 bp	240
60 bp ds duplex (∼13.6 nm)	4	5/RhG, TAMRA, Atto 590, LCR, Atto 680	10 bp < 1 × *R*_0_ for each D–A pair	15–30%	smFRET shows subpopulations with 90% *E*_ee_	231
ds concatenated duplex (>15 nm) attached to QD	4	5/525 nm QD, Cy3, Cy3.5, Cy5, Cy5.5,	0.5, 1.0, 1.5 × *R*_0_ for each D–A pair	10%	Multiple DNA wires arrayed around QD increases *E*_FRET_ QD:(DNA)_8_	241
86 bp ds concatenated duplex (∼29 nm)	6	7/Fl, Cy3, Cy3.5, AF610, Cy5, Cy5.5, AF700	0.3–0.5 × *R*_0_ for each D–A pair	∼5% for 1^st^ 5 ET steps	AF610 acts as energy sink AF700 acts as dark quencher	242
ds concatenated duplex (>14 nm) attached to QD	5	6/Luc, 540 nm QD, Cy3, Cy3.5, AF647, Cy5.5	0.5–0.7 × *R*_0_ for each D–A pair	10%	Luc → QD BRET. Construct = (Luc)_6_:QD:(DNA)_8_	243

a *r*_DA_ D–A separation distance given as a function of *R*_0_ for the D–A pairs, → = ET step, – not given, AF-AlexaFluor, BRET-bioluminescence resonance energy transfer, Fl-fluorescein, LCR-LightCycler Red, Luc-luciferase enzyme, RhG-rhodamine green, TAMRA-tetramethylrhodamine, smFRET-single molecule FRET, 525 nm QD/540 nm QD-quantum dot with 525 nm/540 nm emission maxima.

**Fig. 15:**
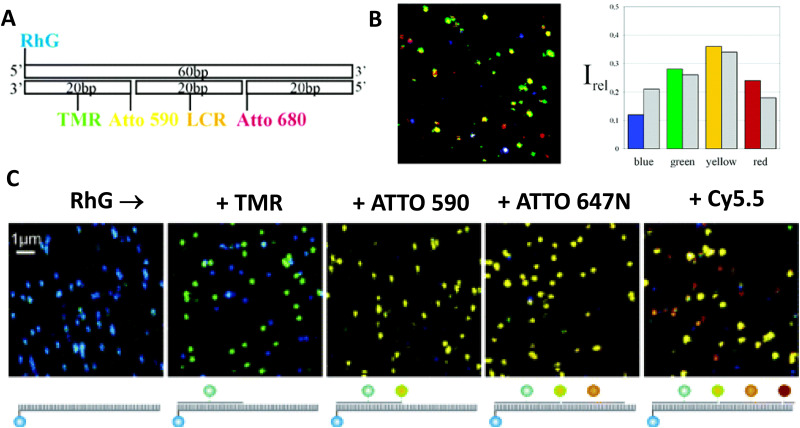
smFRET analysis of a 5-dye wire. (A) Synthesis strategy used to incorporate five different chromophores into dsDNA at well-defined positions. TMR was introduced *via* phosphoramidite chemistry. All other chromophores were covalently attached to amino-modified oligonucleotides using standard NHS chemistry. RhG-Rhodamine Green, TMR - tetramethylrhodamine, LCR-LightCycler Red. (B) False color fluorescence intensity image (10 × 10 μm) of photonic wires adsorbed on dry glass surface (488 nm excitation, 50 nm per pixel, 2 ms integration time, 10–100 counts/2 ms). The different colors demonstrate strongly different multistep FRET efficiencies. Histogram of spectral characteristics of 200 photonic wires (color columns) compared to the distribution as expected (calculated) from the ensemble emission spectrum (gray columns). Reproduced with permission from ref. 231 Copyright 2004 American Chemical Society. (C) Confocal spectrally resolved fluorescence intensity images of hybridized photonic wires on a cover slide as each subsequent TMR, ATTO590, ATTO647N, and Cy5.5 labeled strand is added to the immobilized ss 60 mer carrying biotin on the 3‘-end and RhG on the 5′-end. (detection channels 1–4 are encoded in blue, green, yellow, and red). Reproduced with permission from ref. 232 Copyright 2006 American Chemical Society.

In 2004, the Sauer Lab reported on a 13.6 nm length 60 bp dsDNA construct displaying 5 chromophores at 10 bp placements that was active across the ∼488–700 nm portion of the visible spectrum.^231^ Each chromophore was attached to its own DNA strand with one strand having 2 dyes, these were then hybridized to a 60 mer scaffold which was terminally labeled with the first D dye, see Fig. 15A. Initial fluorescent interrogation of the ensemble samples showed *E*_ee_ between 15 and 30%, however, smFRET spectroscopy performed with four spectrally-separated detectors uncovered the presence of subpopulations exhibiting overall *E*_FRET_ of up to ∼90%! This suggested that several populations were present including ones that were structurally malformed, had unfavorable dipole orientations, suffered from localized energy sinks, or that lacked the presence of a given dye. In subsequent work using essentially the same configuration but with two different terminal A-dyes, the Sauer Lab applied multiparameter single-molecule fluorescence spectroscopy (SMFS) to dissect the different sources of heterogeneity in these type of constructs.^232^ They showed that the homogeneity of ET in these structures could be dramatically improved by immobilizing the structures in aqueous solution to a slide without suffering from perturbation by the surface. Further, *in situ* assembly of the wire molecules (stepwise hybridization of the labeled oligonucleotides) on glass cover slides further decreased structural heterogeneity in overall ET efficiency (Fig. 15C). The latter suggested that the assemblies assumed a more optimized orientation as they assembled stepwise on the surface-attached scaffold in contrast to forcing an already assembled structure to attach to the surface which may induce allosteric heterogeneity.^232^ More importantly, these studies confirmed the diagnostic power of smFRET studies and how they could provide insight into the presence of the different structural populations that constituted such ensemble assemblies where each subpopulation may display their own *E*_FRET_. This lesson would be carried forward in many later studies where smFRET and/or simulations try to account for the presence of such differential populations (*vide infra*). Interestingly, although the term ‘photonic wire’ originated in the mid 1990's from Wagner and Lindsey's description of a porphyrin array,^244^ it was around the time of these two studies from the Sauer group that such dye-labeled DNA structures began to be collectively referred to as ‘molecular photonic wires’ (MPW), a term which is now used interchangeably with ‘DNA photonic wires’.^47,146,147,151,173,231,241,242,245^

**Fig. 16:**
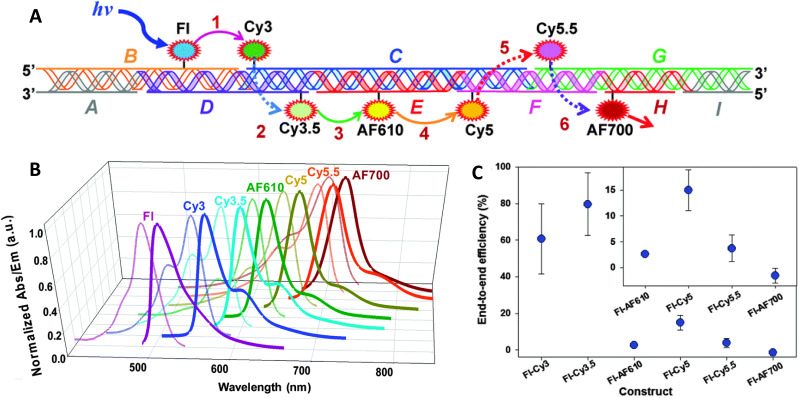
Seven dye FRET cascade. (A) Schematic of the modular ds concatenated DNA scaffold highlighting the 9 different sequences used (A–I), how they assemble together, the position of each of the dyes and the six putative FRET steps. Fl – fluorescein, AF610 – Alexa Fluor 610, AF700 – Alexa Fluor 700. Discrete dye/FRET steps within a given construct are abbreviated as Fl → Cy5.5, for example. Note the schematic does not account for the DNA helicity in dye placement. (B) Normalized absorption/emission spectra of the 7 dyes used. (C) Averaged *E*_ee_ values for each consecutive FRET step within the 7 dye construct. Inset magnifies values for the last 4 steps. Reproduced with permission from ref. 242 Copyright 2014 The Royal Society of Chemistry.

Spillmann and colleagues later tested the boundaries of the linear design with a concatenated dsDNA construct hosting 7 dyes placed at <0.5 *R*_0_ relative spacing for each D–A pair and meant to engage in 6 ET steps that spanned across >300 nm of the visible spectrum (Fig. 16A and B).^242^ As the evolution of ET across this configuration was monitored in a stepwise manner with addition of each increasing A on structures assembled in parallel, it was observed that ET efficiency dropped from ∼80% to <5% when the assembly increased from a 3- to a 4-dye system with AlexaFluor 610 (AF610) as the terminal A. Increasing from that to a 5-dye system with Cy5 now acting as the terminal A for the previous 4 dye system actually increased ET to ∼15% (Fig. 16C) revealing that the AlexaFluor 610 dye acted as a local energy sink despite having excellent putative FRET characteristics on paper and, more importantly, that longer-range transfers could functionally compensate for and bypass a local sink. Interestingly, further additions of Pacific Blue/Alexa Fluor 350 as initial Ds and Alexa Fluor 750 as an even further spectrally red-shifted terminal A to the 7-dye construct were unable to extend the FRET cascade. Similarly functional 4-dye, 3-FRET step dsDNA constructs referred to as ‘linear DNA arrays’ with defined overhangs placed at key points in the assembly have also been used as sensors for other DNA sequences.^246^ Here, the addition of one to three other target DNA sequences would disrupt a specific step in the FRET cascade allowing for identification in a combinatorial or multiplexed manner.

**Fig. 17:**
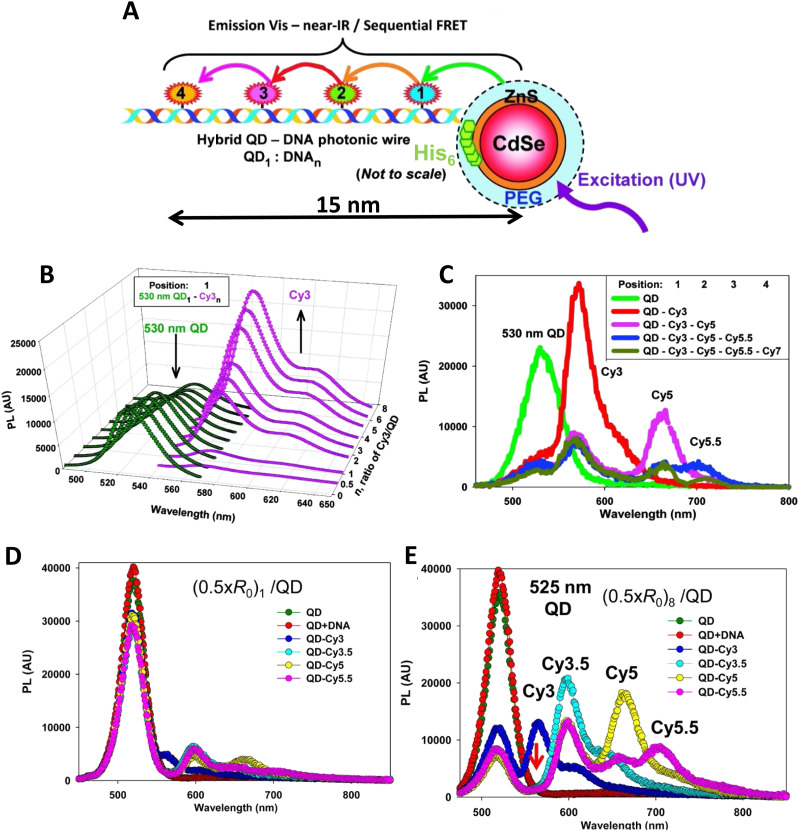
Hybrid QD-DNA linear wires. (A) (His)_6_-modified DNA backbone self-assembled to the QDs *via* metal-affinity coordination. The resulting structure consists of a central QD with multiple, rigid dye-labeled DNA centro-symmetrically arrayed on its surface as designated by the QD_1_ : DNA_*n*_ ratio. UV excitation of the system results in an energy transfer cascade from the central QD through the sequential aligned dye-acceptors. 1–4 indicate sequential dyes and are used to indicate dye position relative to the QD. (B) Deconvoluted PL spectra from 530 nm QD donors self-assembled with an increasing ratio of (His)_6_-peptide-DNA with Cy3 acceptor located in position-1. (C) Composite PL spectra from 530 nm QD donors self-assembled with unlabeled DNA, DNA with Cy3 in position-1, Cy3 in 1/Cy5 in 2, Cy3 in 1/Cy5 in 2/Cy5.5 in 3, Cy3 in 1/Cy5 in 2/Cy5.5 in 3/Cy7 in 4. Panels A-C reproduced with permission from ref. 126 Copyright 2010 American Chemical Society. Representative FRET progression in second generation QD-DNA constructs where the QD displays 1 fully dye-loaded wire (D) or 8 full wires (E). FRET acceptors are placed on the wires with 0.5 × *R*_0_ inter-dye spacing. Green and red curves correspond to the emission spectrum of DHLA-PEG coated QDs alone and then in the presence of just 1 non-labeled DNA wire. Panels D, E reproduced with permission from ref. 241 Copyright 2013 American Chemical Society.

Boeneman used (His)_6_-metal affinity driven coordination to array multiple ds linear DNAs displaying 4 sequential D–A dyes around a central 530 nm CdSe/ZnS core/shell QD (diameter ∼5.8 nm), see Fig. 17A.^126^ In this configuration, the centrally-located QD was meant to act as both an energy harvester and an initial D to sensitize the surrounding photonic wires. When displaying more than one of these DNA wires, the construct can have a radial appearance, however, since the QD can only couple to and sensitize one of the DNA wires around it per excitation event, the photophysical function is essentially that of a linear photonic wire of *ca.* 15 nm. To create this structure, an amine-terminated DNA backbone was coupled *via* chemoselective ligation to a (His)_6_-peptide and this was utilized as a central assembly scaffold to which other dye-labeled D–A DNAs were sequentially placed by hybridization.^125^ The (His)_6_-peptidyl portion facilitated metal affinity coordination to the QD surface with average per QD assembly ratios that follow a Poisson-like distribution.^127^ Each of the dyes utilized were terminally labeled on 10-bp sequences to orient them roughly on the same side of the dsDNA at one helical turn and to also place them at <*R*_0_ for each putative D–A interaction. However, the initial QD D to first A dye (Cy3) center-to-center separation of 7.3 nm was more than 20% greater than the 6.0 nm *R*_0_ for that D–A pair. To help mitigate this, an average of 4 wires were always displayed around the central QD D. Arraying multiple As in a centrosymmetric manner around a central QD D functionally increases the A's absorption cross-section, which acts to improve the effective *R*_0_ and hence, the achievable *E*_FRET_.^135^ This approach increased *E*_FRET_ from ∼10% for a 1 : 1 D : A ratio to ∼30% for a 1 : 4 D : A configuration (Fig. 17B). The functionality of multiple different configurations was investigated including assemblies with repeated copies of Cy5 As in a terminal homoFRET configuration which increased the latter's sensitization. Adding the intercalating dye BOBO-3 to the wires did not help increase FRET for each intra-dye step but rather served to quench the entire system by acting as localized energy traps. Lastly, testing of a QD_1_ → [Cy3 → Cy5 → Cy5.5 → Cy7]_4_ construct showed ∼0.1% ET reaching the Cy7 dye but no subsequent sensitization, see Fig. 17C. The Cy7 appeared to act as a dark quencher in this scenario, similar to the AF610 performance seen in the structure described above.

A second generation of QD-DNA photonic wire constructs were subsequently constructed and tested with a focus on improving the *E*_ee_.^241^ Four main changes were instituted with this latter version including: (i) making the initial DNA-peptide modification chemistry smaller and easier to implement by switching to a disulfide exchange; (ii) optimizing D–A dye pairings and consolidating to only display cyanine dyes on the DNA wire itself; (iii) improving D–A spacing as a function of *R*_0_; and (iv) increasing the number of DNA wires displayed around the central QD from 4 to 8; the latter further improved the *E*_FRET_ of the first step. Overall, *E*_ee_ in this improved construct approached ∼10% representing a nearly 2 orders of magnitude increase in performance over the first-generation assembly, see Fig. 17D and E. Analysis of the results within the context of Förster theory suggested near ideal performance from the initial QD D through the first two sequential Cy3 and Cy3.5 A-relay dyes. Loss in performance resulted primarily from non-idealities (*e.g.*, energy sinks, non-productive orientation) introduced by the last two Cy5 and Cy5.5 A dyes.

In a further iteration of this design, Dwyer demonstrated that such QD-DNA constructs could be engineered to provide their own illumination *via* a biochemically-based self-excitation component thus decoupling it from an external light source.^243^ To accomplish this, the QD-DNA portion was kept essentially the same as described above except that multiple copies of a ∼36 kDA mutated *Renilla reniformis* luciferase enzyme (abbreviated as Luc, the enzyme responsible for bioluminescence in fireflies) were directly assembled to the QD surface in addition to the DNA wires.^247^ Luc was expressed with a C-terminal (His)_6_-motif for conventional purification over Ni-chelate media and this also allowed it to self-assemble to the QDs by metal affinity coordination. Although the 540 nm emitting CdSe/CdZnS/ZnS core/shell/shell QDs (diameter 4.3 nm) utilized could accommodate an estimated maximum of 15 Luc on its surface, in experiments only 6 were used. Luc catalysis of Coelenterazine H (Coel), the enzyme's preferred substrate, produces an electronically excited state with a broad blue emission centered at 475 nm and which displays a QY of ∼0.40. Here, the central QD again acts as both an A, harvesting the initial energy from the bioluminescence, and then as a relay when it sensitizes the proximal first Cy3 relay on the appended DNA wires. Displaying multiple D Luc copies on the QD surface did not increase FRET, rather it increased the probability that a FRET coupling does indeed occur with the QD. An optimized ratio of eight DNA wires per QD was again used in this construct to maintain high *E*_FRET_ from the QD to the first Cy3 A. Remarkably, an *E*_ee_ of ∼10% was estimated for ET through this bioluminescence driven cascade, analogous to that seen for the directly excited QD system described above. The ability to decouple the function of these materials from a light generating device (*i.e.*, fluorometer or laser) and excite them with just the addition of a chemical substrate bodes well for potential application within some type of portable or microfabricated sensor device designed to function at the point of need.

Measuring FRET distances in this same type of QD-DNA wire composite format and then inferring structural assembly from those distances proved to be quite useful in discriminating how different QD-DNA attachment chemistries can influence the structure of the resulting assembly. Deriving the separation distance from the QD D to individual As discretely placed in each of the wire's four increasingly distal positions confirmed that the DNA wires extend laterally from the QD surface with only a small amount of radial movement when assembled by (His)_6_ coordination of a modified DNA.^248^ In direct contrast to this configuration, when the QD is first functionalized with streptavidin with its 4 biotin binding sites, the resulting FRET distances derived from 4-dye labeled/biotinylated DNA wires bound to the streptavidin always place the As close to the QD surface. Although some wires may be aligned correctly and extending away from the QD surface, streptavidin's inherent tetravalency yielded a heterogeneous display of wires in all directions relative to the QD surface across the ensemble sample; some of the As on the wire were always very close to the QD surface and thus dominated the FRET signal.^248^

Although the QD is far larger than the A dyes found on the DNA wires, which in practice are actually displayed on the QD, use of QDs in such configurations brings with it many unique properties as a D for single or multistep FRET configurations including the ability to: (i) tune *E*_FRET_ to the first A by changing the number of wires displayed; (ii) diminish direct A excitation by using a wavelength far removed from its absorption max but which still readily excites the QD due to its absorption which steadily increases towards the blue as almost a continuum starting from just short of its emission; and (iii) easily optimize QD emission wavelength as a function of its size or composition to better match an A absorption profile. For a further description of these and the many other benefits of using QDs in the role of a D, the interested reader is referred to several excellent review articles.^135,249,250^

Cumulatively, we see many useful ‘rules-of-thumb’ and other lessons being learned from studying these first-generation linear photonic wires. The unexpected dark quenching role played by some dyes, *e.g.*, AF610 and Cy7, in configurations where this was not desired suggests that the design of more complex systems incorporating previously untested dyes would benefit from a full pre-evaluation of their FRET and photophysical properties on DNA scaffolds prior to use. Since these structures are all self-assembled and highly modular, almost all controls and derivative structures of interest can be set up at the same time in parallel and with as many replicates as desired; the large amount of experimental data that is efficiently garnered from such approaches in a short time certainly contributes to providing insight in the underlying mechanisms. DNA photonic wires can also be functionally integrated with other FRET participating materials that may be far larger than themselves, such as QDs, for example, or with bioluminescent enzymes where the QDs are now the ET intermediaries. This suggests the possibility of creating such wires that functionally integrate even other types of fluorescent materials. Linear wires where single copies of each D–A dye are paired up in a concatenated manner seem to be limited beyond 4–6 FRET steps due to cumulative energy loss, exciton lifetime, and other non-idealities. SmFRET studies also revealed the presence of different functional conformers displaying drastically different *E*_FRET_ within an ensemble sample. It still remains unclear whether such a mix of high and low efficiency structures is common throughout many of these assemblies or whether this finding was just unique to that experiment.^231^ Additionally, beyond the photophysical characterization, little if any information has been provided on DNA assembly efficiency or how accurately the structure resembles the intended design. Many of these issues would need to be both understood and then potentially addressed in order to create far larger, more complex DNA photonic wires that function with significantly higher efficiency.

### 4.2. Complex DNA structures manifesting complex energy transfer processes

We differentiate from the previous section by focusing on ET processes that are now far more complex in nature. Complexity here arises from two different types of contributions and are implemented either individually or jointly in a given configuration, these include: (i) functional complexity – where the ET processes that manifest on the DNA scaffolds are somewhat unconventional or distinct from what was described above in that they do not represent a linear cascade of dyes with similar photophysical properties and overlapping absorption/emission profiles; and (ii) structural complexity – where the arrangement of dyes on the DNA or the scaffold itself becomes far more complex so as to move beyond just a single, essentially linear cascade of sequential ET steps. For the latter, several themes are repeatedly accessed such as multiple Ds arranged around a proximal A (or the converse), multiple ET pathways converging together, and, of course, involvement of multiple fluorescent materials beyond just arrangements of organic fluorophores. Table 4 presents a tabulated overview of data from some of these examples allowing for comparison of how selected structural and photophysical properties influenced the *E*_ee_ ultimately achieved. In some cases, the following presentation attempts to bring together the cumulative lessons learned across a series of publications. This sometimes means that the order of the reports discussed does not follow a linear evolution in time, but rather builds towards some common understanding where later studies enforce or confirm concepts introduced earlier.

**Table 4:** Select characteristics of representative DNA structures manifesting complex structural and multi-FRET process

DNA structure (estimated length)	# of ET steps	Fluorophores/D → A	*r*_DA_ a	*E*_ee_	Notes	Ref.
7 helix bundle	2	Py, Cy3, AF647	<0.5 × *R*_0_	30–90%	Stepwise *E*_FRET_ depended on ratio of dyes to each other at each step	251
Origami triangle (120 nm per side)	2	Fam, Cy3, Cy5	0.5 to 1 × *R*_0_	70% for 8 : 4 : 1 ratio	Concentric rings of D → relay → A highest AE ∼8 for 8 : 4 : 1 ratio	150
7–10 oligo dual/split rail (∼10 nm for dye containing portion)	3	Cy3, Cy3.5, Cy5/A647, Cy5.5	<0.5 × *R*_0_	∼50%	Replacing 2 Cy5 with a Cy5/A647 hybrid significantly improved *E*_ee_	173
Linear-8 arm; 2 : 1, 3 : 1, 4 : 1 DNA dendrimers^b^	3,4	A488, Cy3, Cy3.5, Cy5, Cy5.5	0.5, 1.0, 1.5 × *R*_0_	28%	Dendrimer yields > 500 increase in terminal A sensitization	252
45 bp ds dimer (∼15 nm)	4	Tb-chelate, A546, A594, A647, Cy5.5	9 bp < 1 × *R*_0_ for each D–A pair	∼8%	Decreased *E*_FRET_ needed for Tb D to 1st A to meet time-gated requirements	253
DNA block (40 × 10 × 15 nm)	4	A488, Cy3, Cy3.5, A647, Cy5.5	<0.5 × *R*_0_	22%	Improved 1/*r*^4^ ET distance dependency due to sheet regime effects	103
DNA dendrimer	5	Luc, A488, Cy3, Cy3.5, Cy5, Cy5.5	0.5 × *R*_0_	16–25%	1st step Luc catalyzed BRET from Coel. Inward and outward directed cascades	254

a *r*_DA_ D–A separation distance given as a function of *R*_0_ for the D–A pairs.

b linear, bifurcated, Holliday junction, and 8 arm star. A in A546-Alexa Fluor and same for other A – designated dyes. AE – antenna effect; Coel – coelenterazine substrate; Luc – luciferase enzyme; Py – pyrene.

In terms of complex ET functionality, the first example actually uses a relatively simple linear DNA scaffold, but delves into the complexity of correctly pairing fluorophore lifetimes in FRET and overcoming D–A lifetime mismatches. Förster's seminal theory makes no explicit mention of analyzing excited state fluorophore lifetime (*τ*) and utilized two identical dyes engaged in homoFRET as its *gedanken* or descriptive example.^255^ When pairing a shorter lifetime D with a longer lifetime A, preferential spectral excitement of the D means that many of the As will typically not be excited and so Ds can engage their paired As and demonstrate FRET. This same situation becomes almost untenable when pairing an organic dye D (typical *τ* ≤ 3 n s) to a semiconductor QD A as the latter's unique blue-shifted absorption profile, its larger finite size/cross section, and especially its far longer lifetimes (*τ* = 20–50 ns or longer when considering multi-exponential decays) all conspire to make finding actual evidence of FRET in this configuration quite challenging.^135,256^ In such a configuration, the QD As are almost always more efficiently excited than the dye Ds and their excited state outlives that of the shorter lifetime D dye. Hildebrandt ingeniously showed that utilizing long-lifetime rare-earth Tb chelates (*τ* = millisec) as the D could functionally overcome this mismatch since any excited QD A will quickly relax back to the ground state in less than a microsecond and become an available A for the still excited Tb chelate Ds they are proximal to.^257^ There is, however, no physical reason a short lifetime organic dye could not donate energy to a long lifetime QD if a way could be found to selectively excite it as the D but not the proximal QD A. Díaz was able to prove this conjecture by placing an organic dye FRET relay between an initial Tb D and a terminal QD A using a DNA scaffold.^258^ By labeling modified oligos with the Tb D chelate and a Cy3 dye relay, and then using a chimeric (His)_6_-DNA as a QD-attachment point, concatenated Tb-Cy3 oligo structures could be assembled onto the QD A where the number of attached structures and the relative spacing between the D-relay-A moieties could all be controlled. The structure of these QD-scaffolded photonic wires resembles those described in the section above, but the ET pathway is now in a reversed configuration. Surrounding the central terminal QD A with multiple wires now increases the probability that a FRET coupling to it does indeed occur. Using a time-gated fluorimetric setup, definitive evidence of FRET from the Tb to the Cy3 intermediary and then the QD was observed. Moreover, using the intermediary dye as an ET relay between the two extended lifetime Tb and QD components actually increased the distance of viable Tb–QD FRET to ∼14 nm, which is almost 2× that of their estimated 7.6 nm *R*_0_ value.

**Fig. 18:**
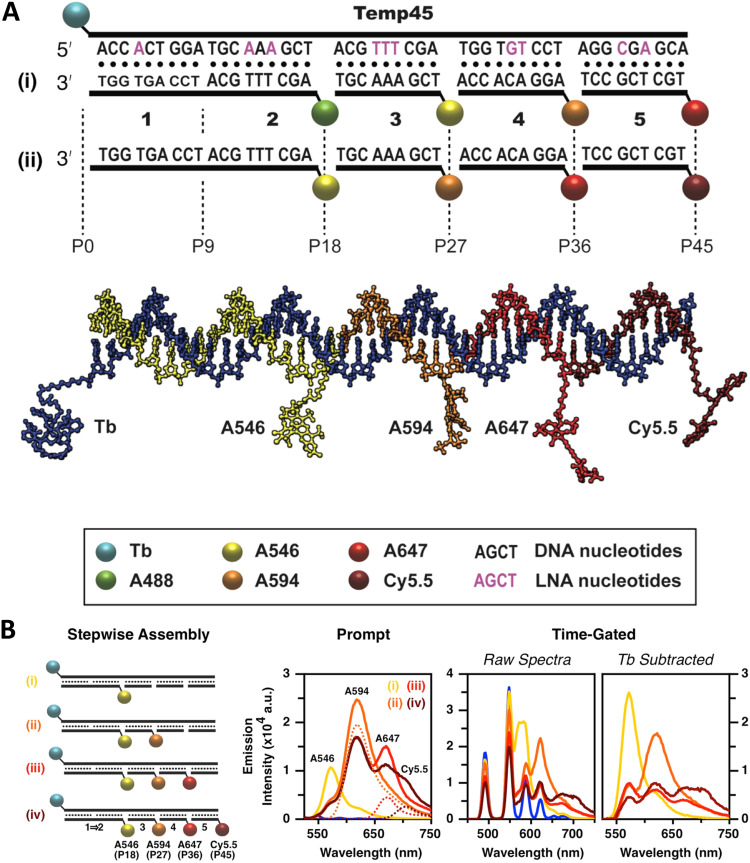
Design of the Temp45 photonic wire and time-gated FRET. (A) Nucleotide sequences of the template and complementary segment strands; two arrangements of fluorescent dyes, (i) and (ii), and a model of the photonic wire in (ii). Boldface Arabic numbers are used to denote segment strands and P*n* labels denote dye positions. (B) Evolution of the FRET cascade in the Tb/A546-initiated photonic wire. From left to right: stepwise assembly of the full photonic wire; representative prompt emission spectra (estimated direct excitation components are shown as dashed lines and arise from the UV absorption of the dyes); representative raw time-gated emission spectra; and corresponding Tb-subtracted time-gated emission spectra. The colors of the spectra correspond to the colors of the lowercase Roman numeral labels. Lag time and integration time of 32 and 350 μs, respectively, for time-gated measurements. 20 μs integration time used for prompt measurements. Reproduced with permission from ref. 253 Copyright 2015 American Chemical Society.

Using a functionally analogous approach, Algar exploited the same type of Tb-chelate as an initial D to drive a time-gated FRET cascade consisting of 4 sequential organic fluorophores assembled on a concatenated ∼15 nm long dsDNA scaffold, see Fig. 18A.^253^ Time-gating in this case was meant to minimize background emission improving signal to noise. Sources of background emission include: direct excitation of fluorescent dyes along the length of the photonic wire; unbound dye-labeled DNA strands in the sample; and potential biological sample matrix should this structure eventually be utilized there. The DNA sequences also included locked nucleic acid (LNA) portions, which served to increase the thermal stability of the photonic wire. Along with showing that the Tb chelate could act as an initial FRET D to a variety of next in line As at different spectral positions along the wire, Algar showed that the key to using the Tb as an effective initial D was to optimally position the next-in-line acceptor dye in a so-called “sweet spot.” With this positioning, the FRET efficiency was sufficiently high for practicality, but not so high as to suppress time-gated emission by shortening the Tb D emission lifetime to within the instrument lag or delay time required for measurements. For the Temp45 structure shown in Fig. 18, the *E*_ee_ from the A546 dye at P18 to the terminal Cy5.5 dye at P45 (∼9 nm) was estimated at 16 ± 5%, which decreased to 8 ± 3% when considering ET from the Tb at P0 to the dye at P45 (∼15 nm). The underlying DNA scaffold in this structure is again quite simple allowing for modular inclusion and exclusion of dyes; the latter in the form of all the various control samples was again key to revealing the complex interactions between the initial long-lifetime Tb chelate D and the sequentially staggered downstream As. Somewhat counterintuitively, when considering how to optimally sensitize a downstream chain of sequential ET events, *E*_FRET_ from the Tb to the first dye had to be decreased to extend the quenched Tb D lifetime sufficiently to meet the functional constraints of optimized measurements on a time-gated fluorimeter.

**Fig. 19:**
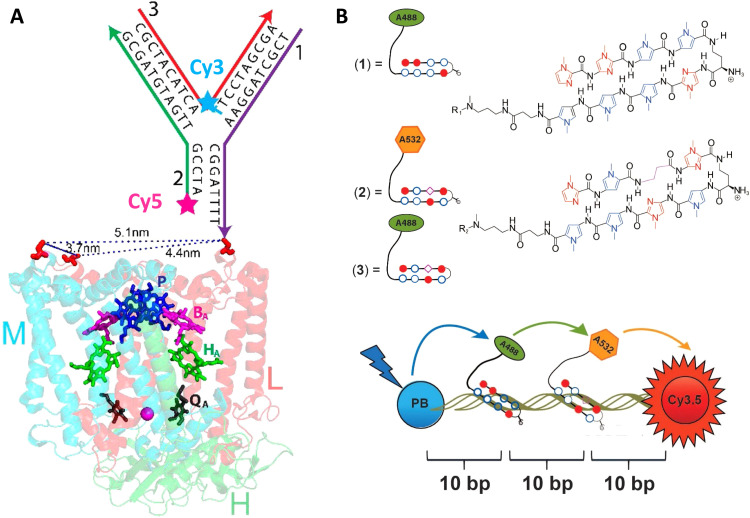
DNA-sensitized reaction centers and polyamide driven assembly of dyes to DNA duplexes. (A) Modified structure of the reaction center (RC) from *Rhodobacter sphaeroides* 2.4.1 (PDB 2J8C10) with sequences of the three-arm-DNA construct shown. Cofactors of the RC are colored, those active in electron transfer reactions are designated by letters: P, bacteriochlorophyll pair; BA, bacteriochlorophyll monomer; HA, bacteriopheophytin; QA, ubiquinone. The arrows in the DNA structure point in the direction of the 3′-end of DNA strands. The 3′-amine-modified strand-1 (purple) of the three-arm DNA is conjugated to one of the Cys (shown in red) on the surface of the RC. Other two strands (strand-2 and -3, in green and red, respectively) hybridize to strand-1 to form the three-arm-DNA junction. Inter-Cys distances on the RC marked as dotted lines. Reproduced with permission from ref. 259 Copyright 2014 American Chemical Society. (B) Top: Structure of the fluorophore-tethered pyrrole-imidazole polyamides where 1 binds the sequence 5′-ATGGACA-3′ while 2,3 bind to 5′-TACTGCT-3′. Bottom: Schematic of the ds 30 bp Pacific Blue (PB), Alexa 488 (A488), Alexa 532 (A532), Cy3.5 multiFRET construct. Reproduced from ref. 260 with permission from Springer Nature, copyright 2013.

As described initially, a principal benefit of DNA nanostructures is their biocompatibility and ease of functionalization through myriad available chemistries. Dutta and colleagues at the Biodesign Institute of Arizona State University, took direct advantage of this to couple three-arm-DNA nanostructures containing Cy3 and Cy5 dyes to isolated photosynthetic reaction centers (RCs) from the purple bacterium *Rhodobacter sphaeroides* as schematically shown in Fig. 19A.^259^ For bioconjugation, an aminolated DNA terminus was attached to one of three surface-exposed cysteine-thiols on the RC using a commercial heterobifunctional cross-linker allowing for up to 3 DNA structures to be conjugated to the RC protein with measured DNA:RC ratios ranging from 0.8 to a max of 2.4. Using the DNA arm as an external antenna, the efficiency of charge separation in the conjugated RCs approached 100% reflecting that the increase in absorption cross-section provided for a 2-fold increase in charge-separated states. Complexity here comes from taking natural biomaterials that are functionally orthogonal to each other in nature in the form of taking a DNA-scaffolded dye antenna and coupling it to RCs in pursuit of augmented charge separation. Such composites suggest themselves for developing bionanomaterials that are capable of either powering themselves or driving downstream coupled catalytic process by harvesting light.

The assembly of complex photonic DNA nanostructures can also incorporate novel chemistries beyond covalent dye linkages and the like. For example, Burley and colleagues demonstrated that pyrrole-imidazole polyamides, which are a family of minor groove DNA binding ligands, could display sequence specificity and bind to targeted portions of dsDNA to yield functional photonic wires. Here, the polyamides helped align covalently attached Alexa Fluor 488 (A488) and Alexa Fluor 532 (A532) relay dyes to targeted sequences to yield 3-dye ET wires hosted on a duplex and also a 3-way arm, as depicted in Fig. 19B.^260^ For the duplex structure, the dyes were programmed to be at 10 bp separation and stepwise addition of the Alexa dyes to a dsDNA scaffold displaying the initial Pacific Blue D and a terminal Cy3.5 A increased *E*_ee_ more than 3-fold from 4% to 14%. The beauty of this approach lies in the ability to add more dyes site-specifically as desired to already formed dsDNA structures without requiring synthetic modification of a constituent oligo. Such added dyes could help augment FRET directionality along with *E*_FRET_ in a given assembly.

There are many other interesting examples where alternative chemistries and especially different fluorophore materials have been utilized to create various complex DNA incorporating photonic wire construct. In many cases, this is done for a variety of reasons including to make up for some deficiency, augment some capability, create a viable heterogeneous material, incorporate a non-standard fluorophore or fluorophore environment, and, of course, to provide properties that are not inherently available to the DNA or other dye material(s) alone. For example, to address self-quenching issues from excessive proximity and perhaps aggregation, cyclodextrin caps were integrated into porphyrin G-quadruplex DNA nanostructures, allowing very densely packed dye nanostructures that improved their fluorescence by 180-fold as compared to the non-cyclodextrin designs.^261^ Still another approach mimicked nature in using a lipid bilayer to provide a protective biocompatible environment for porphyrin As coupled to DNA and paired with intercalated YO-PRO-1 as an antenna D; this design improved the AG by 12-fold.^262^ Porphyrins were also used in the work of Anderson *et al.* where 5' azide-terminated ssDNA was covalently attached to a Zn-tetra-(phenylethynyl) porphyrin (ZnTPEP) meant to be an A utilizing copper(i) catalyzed azide–alkyne cycloaddition to form DNA-porphyrin adducts containing from 1 to 4 ssDNAs attached around the porphyrin core.^263^ Quasi-cruciform shaped ET arrays formed using this with the porphyrin A at the center were then assembled by hybridizing Alexa Fluor 546 D to the DNA and these demonstrated almost 90% *E*_FRET_ in the full four D-labeled 4-arm configuration. The work of Oh *et al.* further compared dendrimers containing porphyrin dyes (zinc as D and free-base as A) but with a focus on the symmetric components of the dendrimers.^264^ Here, they investigated the added benefit of homoFRET and found that light-harvesting increased with homoFRET by lessening the impact of defects in the structures. HomoFRET can also be exploited to increase the overall absorption cross-section of DNA photonic systems, as discussed below where the inclusion of repeating linear Cy3 sections within a five dye FRET system resulted in ∼50% increase in AG (*vide infra*).^151^

Instead of using the DNA to host the LH system as in the previous examples, light-harvesting supramolecular phenanthrene polymers were end-labeled onto DNA by the Häner group to activate a 3-dye FRET Cy3, Cy5 cascade feeding into a final Cy5.5 A that improved AG by up to 23-fold.^265^ Facilitating this, phenanthrene monomers were terminally attached to DNA oligos during synthesis and these then self-assembled into the final experimental polymer structure. The same group later also integrated spermine into dye labeled DNA-phenanthrene structures that formed vesicles with 50–200 nm diameters and functioned as light harvesters.^266^ The interesting part of these vesicles is that they could be reversibly controlled by modifying the salt content of the solution, for example, 200 mM NaCl reduced the FRET and therefore LH to zero. In a more recent example, the Gothelf group utilized a conjugated phenylene-vinylene-based polymer capable of transferring photonic energy.^267^ Relevant to our interests, they functionalized the polymer with multiple DNA strands with Atto425 dyes as light harvesting Ds and immobilized it on DNA nanostructure that had an Atto594 dye as the final A to yield a single molecular wire system. This unique example exploits DNA's capability as a versatile chemical conjugation handle in the ssDNA form and then as templates for other nanostructures in its DNA origami form. Using this strategy, ET transfer of up to 24 nm with ∼4% *E*_ee_ was achieved,^267^ which was in line with other DNA MPWs studied at room temperature.^149,268^

**Fig. 20:**
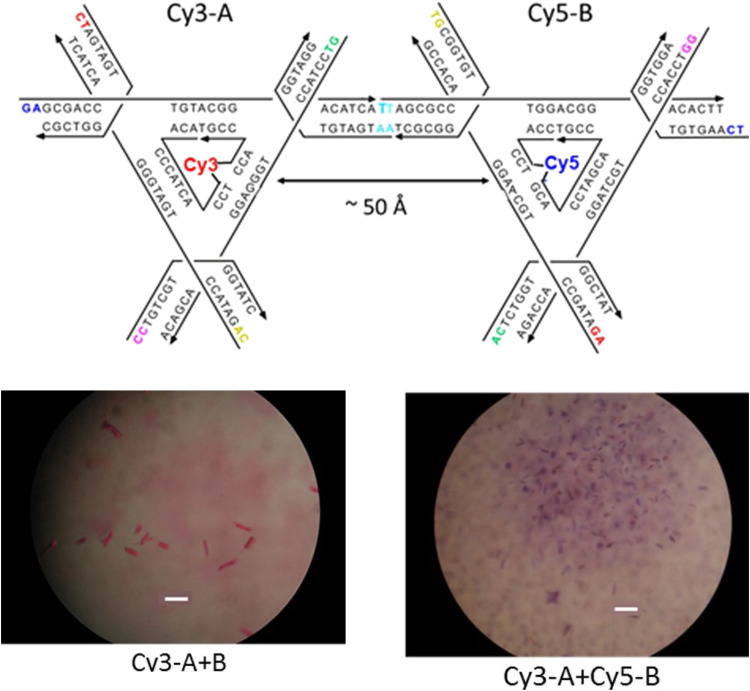
Dye incorporating DNA crystals. Top: Tensegrity triangle sequences that form the 3-D DNA crystals. Bottom: Micrograph image of DNA crystals with a Cy3 labeled A triangle and unlabeled B triangle. Micrograph image of DNA crystals with Cy3 labeled A triangle and Cy5-labeled B triangle. Scale bar in both images is 100 μm. Reproduced with permission from ref. 270 Copyright 2016 American Chemical Society.

A far different yet still fascinating form of bp driven nucleotidyl structures are those of DNA crystals, which can be based on building block structures, such as tensegrity triangles or even small DNA sequences, and then extended and repeated until a DNA-based 3D crystal is obtained.^269^ Such crystals can extend beyond the nanoscale size regime common to the previous examples and reach micron scales while their rigid internal structures can be quite beneficial for improving the photonic properties of any integrated chromophores. A DNA crystal that integrated a Cy3–Cy5 D–A FRET pair revealed that the Cy3 fluorescence was 2-fold higher in the crystal as the structure's rigidity suppressed excited-state dye isomerization which often occurs due to the presence of a methine bridge in the cyanine backbone, and this then correlated with an enhanced *E*_FRET_, see Fig. 20.^270^ Paukstelis and coworkers developed DNA crystals based on a single DNA 13-mer that could form large 3D crystals and by using controlled addition of small proportions of secondary interrupting DNA strands, termed poison strands, the shape of the crystal could be dramatically modified from pyramidal to columnar.^271^ In subsequent work, they demonstrated the capability to integrate fluorescein and Cy5 dyes in a layer specific manner, obtaining both core–shell and layer-by-layer formations that presumably had some ET interactions.^272^ Though the ET examples in DNA crystals at this stage are merely proof-of-concept, the enhanced photonic properties of the integrated chromophores and the unique capability to expand to the macroscale make them potentially relevant to light harvesters or even light emitter diodes.^273^

DNA based photonic systems can also encompass actual nanofibers where the DNA is not exploited for its base pairing properties but rather as a dye-conjugated or dye-displaying material within DNA-surfactant nanofibers. Work in this area is epitomized by the efforts of the Sotzing lab,^274,275^ who created salmon DNA- (an economic source of long length dsDNA) CTMA (cetyltrimethylammonium chloride surfactant) complexes that were then spun into nanofibers with diameters averaging 300 nm. The periodic arrangement assumed by the DNA and CTMA (lamellar structures of aligned, parallel DNA, sandwiched by CTMA layers) allowed for association of the dye chromophores in individual sites, including intercalation and/or groove binding and this helped avoid fluorescence quenching.^274^ The FRET systems were based on Cm102 and Hemi22 as a D–A pair with the A-to-D ratio varied from 1 : 200 down to 1 : 5 and this shifted the emission from blue to orange. The 1 : 20 ratio resulted in color coordinates (0.35, 0.34) corresponding to almost pure white-light emission.^274^ Long *et al.* went a step further and coordinated three dyes into their DNA-CTMA nanofibers, specifically Hoechst 33258 (D), acriflavine (relay) and Rhodamine 6G (A).^276^ The FRET relay within this DNA-CTMA fiber allowed it to function as a low loss molecular waveguide with a transmission of up to ∼40 μm for the red light emitted by the Rhodamine 6G.

As already witnessed in several examples above, conjugation of DNA to inorganic NPs is a rapidly growing field with important application space in nanoplasmonics, sensors, and nanophotonics amongst others.^20,21,41,277^ NP conjugation to complex DNA structures have also been studied in the context of light-harvesting and ET.^278^ Plasmonic NPs, such as Au or Ag, are known to modify light-matter interactions, yet these effects can have positive or negative effects by enhancing or reducing dye emission and ET.^279^ To properly understand the effect, Anderson *et al.* studied a DNA origami breadboard with Alexa Fluor 488, Alexa Fluor 546, and zinc(II) tetraphenylporphyrin dyes in conjugation with 5 and 10 nm gold NPs, and found enhancement in the FRET within the DNA–dye arrays.^280^ The Acuna group exploited a similar approach with single gold NPs, with varying diameters of 5, 10, 15, and 20 nm, were placed at fixed distances from an Atto532-Atto647N D–A FRET pair.^281^ Ingeniously, they subsequently photobleached the dyes to determine the plasmon assisted FRET rates and observed that the gold NPs have minimal effect on the FRET rate, which contradicts previous reports.^34^ A core–shell approach has also been described where an interior AuNP has satellite semiconductor QDs displayed around it through DNA conjugation. Here, the DNA was used to vary the distance between the NP and the QD.^282^ As noted above, gold NPs can modify the emission properties of fluorescent moieties such as QDs through their plasmonic interactions. The authors found that for a 50 nm diameter AuNP, if the QDs were closer than 15 nm from the surface the emission was quenched while the emission was maximized (∼1.6-fold) at 20 nm from the surface.

**Fig. 21:**
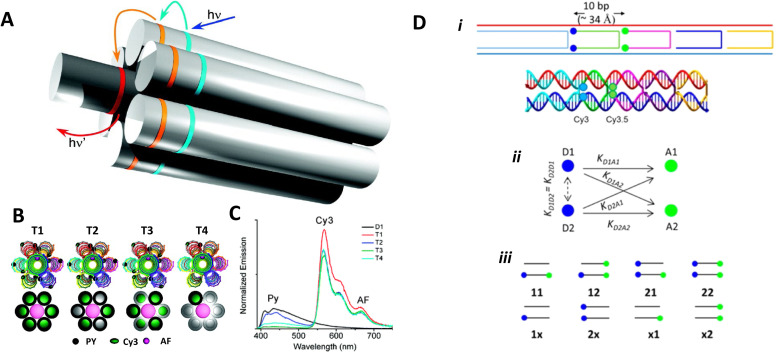
Seven-helix DNA bundle hosting cyclic arrays of three chromophores and dual rail structure. (A) Schematic of the self-assembled 7-helix bundle (7HB) nanoscaffold containing three distinct arrays of chromophores: the primary D, the intermediate relay R, and the A, represented by the cyan, orange, and red rings, respectively. Upon excitation of the primary D array, a stepwise energy transfer cascade occurs. The distance (along the helical axes) between the dyes in adjacent arrays is 3.5 bps. (B) Schematic representation of each triad. The black spheres, dark green ovals, and pink spheres represent Py, Cy3, and AF, respectively. A simplified representation of each triad is also shown below the corresponding helical schematic, where the colored circles represent the presence of the dye molecules on the DNA helices. Triads 1–4 contain ratios of 6 : 6 : 1, 6 : 3 : 1, 3 : 6 : 1, and 1 : 1 : 1 of the Py, Cy3, and AF dyes, respectively. (C) Normalized emission spectra of D1 and T1–T4, all with excitation at 380 nm. Reproduced with permission from ref. 251 Copyright 2011 American Chemical Society. (D) i – Upper: Dual rail structure schematic self-assembled from seven DNA oligos. Cy3 and Cy3.5 positions at the ends of hairpin oligos indicated by solid blue circles and solid green circles, respectively. One Cy3 attached using a 3-carbon linker, while the other Cy3 uses a 6-carbon linker. Cy3.5s are each attached using a 3-carbon linker. Lower: Schematic of the dual rail DNA structure showing the double crossover motif and dye positioning. ii – Putative ET pathways from Cy3 (solid blue circles) and Cy3.5 (solid green circles). The solid black arrows indicate D–A hetero-FRET and dashed black double arrow is a homo-FRET coupling between the two Ds. iii – Schematics of the FRET structures and control structures. Reproduced with permission from ref. 160 Copyright 2016 American Chemical Society.

In terms of how underlying structural complexity has influenced DNA-based photonic wires and relays, one of the early and significant examples that highlighted what DNA could potentially offer as a scaffolding architecture was based on exploiting a 7-helix bundle (7HB) assembly. The Liu lab utilized a design originating from Seeman consisting of a cyclic arrangement of six helices with a honeycomb cross-section assembled around a protruding central helix, see Fig. 21A.^251^ By incorporating dye-modified oligonucleotides consisting of an initial pyrene D, a Cy3 relay, and a terminal AF647 A at selected positions, this architecture provided for a series of ring-shaped 2-step ET networks or triads (Fig. 21B). Both time-resolved and steady-state measurements, see Fig. 21C, revealed *E*_FRET_ ranging from 30 to 90% with the higher values obtained when the first pyrene → Cy3 ET step had ratios of 6 : 6 and 3 : 6 and the lower *E*_FRET_ associated with a ratio of 6 : 3. Estimated AE values ranged from 16% to 85% with the lowest value observed, as expected, in a structure having only a 1 : 1 : 1 ratio of each of the 3 dyes. Despite the originality of this design, which enabled the key cyclic arrangement, the vast majority of space on the DNA scaffold did not host any dyes, suggesting that such structures could explore significantly more complex arrangements of dyes. In line with this, the authors noted that although multiple possible ET pathways were present in each multidye configuration, actual ET to the final A appeared to be always unidirectional.^251^

Natural light harvesting (LH) complexes are thought to utilize multiple ET pathways as a design principle for achieving high-energy transfer quantum efficiencies. In the context of DNA-templated FRET networks designed to mimic this aspect of LH antennae, Buckhout-White *et al.* used simulations to show that multiple redundant pathways could compensate for inhomogeneities and non-ideal ensembles and help improve overall FRET quantum efficiency.^252^ Melinger *et al.* demonstrated the potential benefits that multiple interacting ET pathways could make to the overall achievable *E*_FRET_ in DNA structures.^160^ Considering the potential complexity that large dye networks may eventually assume, they targeted a simplified structure that could host multiple FRET pathways depending upon the configuration assembled. This consisted of a so-called dual rail (DR) platform assembled from 7 oligos where select crossovers were labeled with either one or two copies of a Cy3 D and a Cy3.5 A. D–A dyes were separated by 10 bp or 3.4 nm which placed them at a distance between their corresponding 0.5 × − 1.0 × *R*_0_ values. They examined FRET in a series of assemblies displaying either single or double dye configurations or both, see Fig. 21D.^160^ Application of steady-state, time resolved lifetime/anisotropy, and ultrafast pump–probe measurements allowed them to isolate and extract the contributions that the underlying single homo- and four heteroFRET pathways make to stepwise and overall *E*_FRET_. Their results confirmed that multiple FRET pathways lead to an increase in *E*_FRET_ and that this arose in part because of the presence of homoFRET between the Ds, thereby providing access to multiple parallel pathways to the A; these could cumulatively compensate for low *E*_FRET_ channels presumably arising from a static transition dipole distribution. This was one of the first reports to advocate for implementing these compensatory processes during the initial design of the FRET network.

**Fig. 22:**
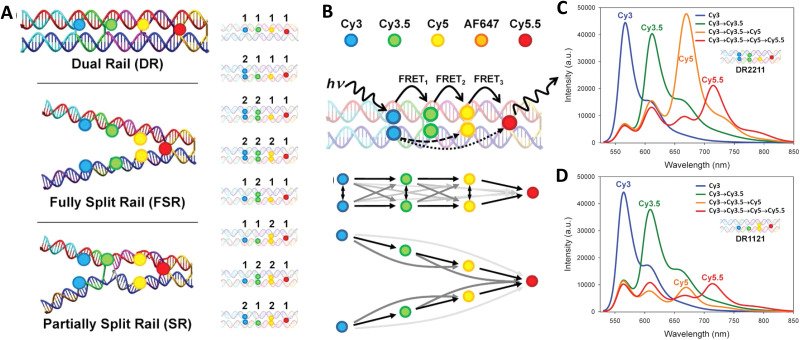
Comparison of FRET pathway redundancy in dual *versus* split rail DNA constructs. (A) Schematic of DNA rail structures including dual rail (DR), fully split (FSR), and partially split rails (SR). Each is assembled from several constituent dsDNA oligonucleotides highlighted by colored strands. Full array of DR constructs each dye shown on right. (B) Cy3, Cy3.5, Cy5, AF647, and Cy5.5 dyes used as energy collection, transfer, and reporter elements. Potential ET pathways, including the three primary FRET channels (FRET_1–3_) and longer distance interactions, from the primary Cy3 donor. ET pathways in the DR construct highlighting multiple interacting pathways and split rail SR construct where the two branches are depicted acting primarily as two independent wires. Spectral evolution of the DR2211 (C) and DR1121 (D), representing the best and worst performing structures, respectively, with the addition of fluorophores Cy3.5, Cy5, and Cy5.5. Reproduced with permission from ref. 173 Copyright, 2021, John Wiley & Sons.

In a more comprehensive follow-up study, Cunningham *et al.* put these suggestions to the test with more in-depth studies on derivatives of the aforementioned DR platform in pursuit of a functional multi-dye/multistep FRET DNA system of minimal complexity that would also maximize *E*_FRET_, see Fig. 22A.^173^ Using a model system consisting of three FRET steps in a 4-dye Cy3 → Cy3.5 → Cy5 → Cy5.5 FRET cascade, they evaluated how the DR platform employed multiple interacting *versus* redundant FRET pathways *via* steady-state, time-resolved, and single molecular fluorescence spectroscopy (SMFS). Variants of the DR design, where one or two copies of each dye are aligned in rigid linear parallel rows, were compared to a split rail (SR) format, where varying degrees of spacing were introduced between the ‘independent’ rails or duplexes (Fig. 22B bottom structure). Experiments in combination with detailed numerical simulation of the ET and MD modeling of the structures revealed the DR design as more efficient than the SR along with the critical role-played by functional and detrimental dye redundancy, see the difference in terminal Cy5.5 emission in Fig. 22C and D. These results suggested the design principle that efficient FRET networks must balance the increase in FRET rate from multiple interacting pathways with undesirable fluorescence quenching between dyes in close proximity. Moreover, hybrid fluorophore combinations where duplicate copies of Cy5 at the 3rd dye position were replaced with a Cy5/AF647 hybrid mix were identified as a strategy to mitigate this quenching. Overall, optimized DR designs displaying a three-step energy cascade with only 7 dyes were capable of achieving an *E*_ee_ of ∼50%, representing a marked improvement over many previous DNA networks with more dyes, see Tables 3 and 4. It is important to note that for each primary experimental structure assembled in Fig. 22A, every single dye permutation was also assembled and evaluated as a control to extract estimates for the direct excitation components of downstream dyes along with evaluating the potential contributions from longer range ET interactions when intermediary dyes were missing; this again attests to the power of DNA assembly as an easily accessible, modular platform capable of being implemented in a massively parallel format. Achieving even more improvements in *E*_ee_ beyond this would, of course, be predicated on including the right design considerations and especially those that relate to D–A ratios and spacings that allow for the requisite interactions.

**Fig. 23:**
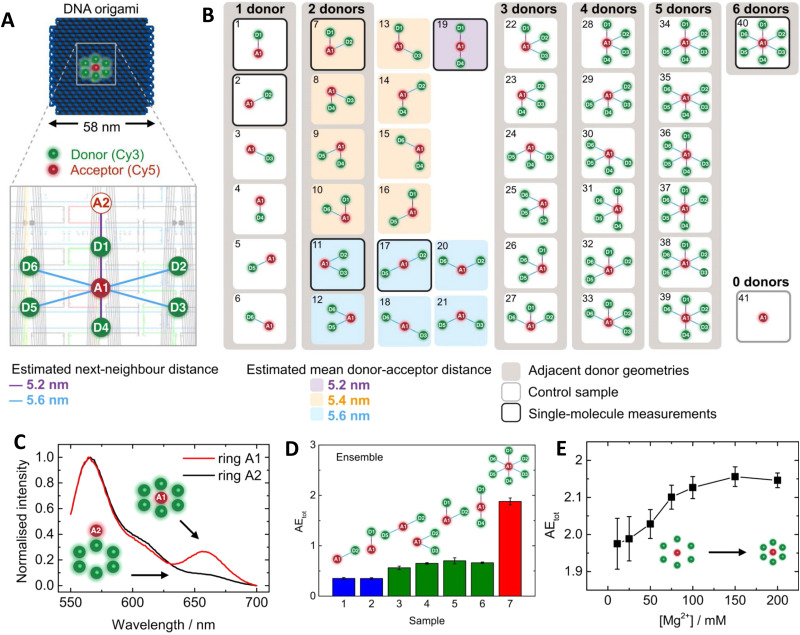
Systematic evaluation of LH in a circular ring configuration. (A) Ring of Cy3 D dyes (green) surrounds a Cy5 A dye (red) on a flat, square-shaped DNA origami platform. The zoomed-in window shows the precise fluorophore attachment sites using a caDNAno scheme. (B) For each number of Ds N, all six permutations of adjacent D positions (gray boxes) were prepared and compared to an acceptor only control sample (gray frame). For *N* = 2, all possible dye geometries were assembled resulting in three groups of different mean D–A distances (colored boxes). The seven structures highlighted with black frames were analyzed both in ensemble and single-molecule measurements. (C) Normalized fluorescence emission spectra with A inside and outside the D ring. (D) AE_tot_ obtained from ensemble measurements. Samples analyzed include one-D (blue), two-D (green), and six-D (red) assemblies. (E) Influence of Mg^2+^ concentration on antenna effect (AE_tot_) for the six-D ring with an A in the center. Increases in Mg^2+^ concentration to shrink the interhelix distances in DNA origami structures. Reproduced with permission from ref. 283 Copyright 2016 American Chemical Society.

Chin and coworkers from the Cavendish Laboratory at Cambridge were able to provide strong support for the role of D–A ratios by systematically analyzing light-harvesting efficiency as a function of D number and interdye distances; these experimental formats exploited ensemble and single-molecule fluorescence spectroscopy based on alternating laser excitation in conjunction with detailed Förster modeling.^283^ The ring-like DNA antenna design they utilized consisted of a flat square origami scaffold where a central Cy5 A could be controllably programed to have from 1 to 6 Cy3 surrounding Ds located at slightly different average distances, see Fig. 23A. As reflected in Fig. 23B, C, and D, they were able to confirm that LH efficiency, as measured by the AE, depended upon both correct geometrical placement and the D : A ratio. Along with showing a linear increase in AE as function of D number present, they were able to increase AE even further by bringing the surrounding Ds closer to the central A. The latter was achieved by increasing the Mg^2+^ concentration present which served to reduce the origami structure's inter-helical distances through electrostatic screening of the negatively charged DNA backbone (Fig. 23E).^283^ Chin's fascinating study also served as another prime example of the power available in accessing parallel DNA assemblies to systematically investigate multiple variables.

In a mechanistically related example, Olejko and Bald brought to bear a 3-dye system comprising of fluorescein D, Cy3 relay or transmitter, and terminal Cy5 A assembled in analogous concentric rings on a origami-based pyramid to probe many of the same variables.^150^ From their results, they suggested several guiding design principles in the context of their D-relay-A system, including: (1) to increase AE, the number of energy-delivering pathways must be increased by increasing the number of D and relay molecules; a rosette-like design was put forth as being best suited. (2) *E*_FRET_ can only be increased by optimizing spectral properties and making intermolecular distances small. The number of D molecules barely changes the FRET efficiency but does increase the probability that it occurs. (3) The final ET step is also influenced by the number of dyes, which are further blue shifted in the electromagnetic spectrum (addition of extra upstream Ds acting as antenna). (4) Including a variety of fluorophores resulting in multiple FRET steps leads to a broader range of possible excitation wavelength.^150^ Confirmation of these generalized suppositions, but in the context of an increasing numbers of separate ‘feeder’ arms containing multi D–A hetero/homo-FRET pathways leading to some downstream apex A are already seen in some of the above examples and will also be seen repeatedly in other examples to come (*vide infra*).

**Fig. 24:**
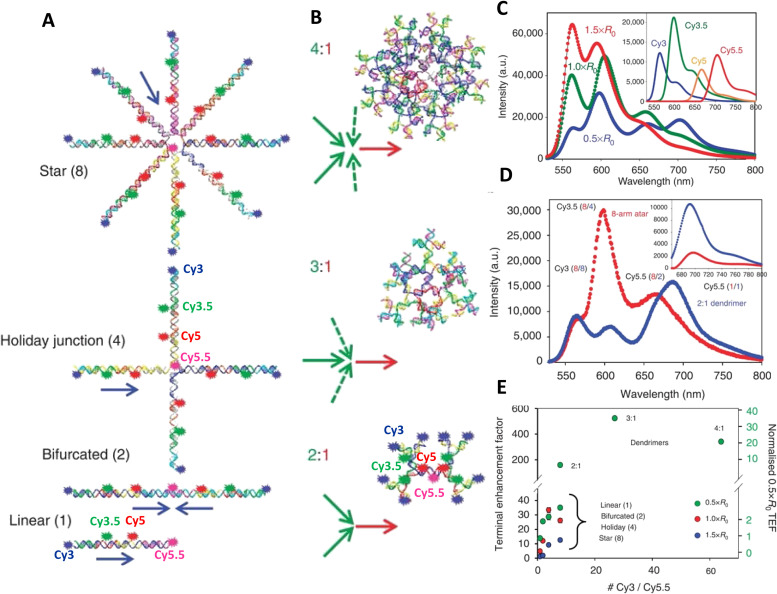
Comparison of linear *versus* dendrimeric photonic wires. (A) Linear [Cy3 → Cy3.5 → Cy5]_*n*_ → Cy5.5 four-dye, three FRET step systems with sequential donor–acceptor arrangements of Cy3 (blue), Cy3.5 (green), Cy5 (red) and Cy5.5 (pink) in photonic wire configurations. The number of [Cy3 → Cy3.5 → Cy5]_*n*_ wires leading into each terminal Cy5.5 dye increases from one to eight using linear, bifurcated, HJ and eight-arm star constructs. Blue arrows indicate directionality of the FRET cascade(s) along each wire in each structure as they converge on the terminal Cy5.5 A. (B) Dendrimer-based FRET systems in configurations where each dye preceding the central-terminal Cy5.5 A has two, three, or four Ds. D–A spacing for the dendrimers fixed at 0.5 × *R*_0_. The linear, HJ, and 2 : 1 dendrimer structure show approximate dye locations. (C) Representative comparative spectral data for the 0.5 ×, 1.0 ×, and 1.5 × *R*_0_ linear systems. Data were normalized to the direct Cy5.5 emission at the same excitation. Inset: Decomposed individual component spectra for the 0.5 × *R*_0_ linear system. (D) Comparison of normalized emission for the 0.5 × *R*_0_ 2 : 1 dendrimer and eight-arm photonic wire star structures. Dye ratios corresponding to each position in each structure are indicated with red or blue numbers in parenthesis. Inset: Decomposed Cy5.5 sensitization for the 2 : 1 dendrimer (blue) is much larger than for the eight-arm star (red). (E) Plot of Cy5.5 terminal enhancement factor (TEF) for the [Cy3 → Cy3.5 → Cy5]_*n*_ → Cy5.5 photonic wire and the 2 : 1, 3 : 1 and 4 : 1 0.5 × *R*_0_ dendrimer structures as compared with the initial 1.5 × *R*_0_ linear system (left axis). Right scale plots TEF for only the 0.5 × *R*_0_ structures as compared with the 0.5 × *R*_0_ linear four-dye system. Reproduced from ref. 252 with permission from Springer Nature, copyright 2014.

The complex interplay of FRET pathway redundancy, D–A ratio, and linear *versus* concentric or dendrimeric D–A configurations were definitively investigated by Buckhout-White and colleagues.^252^ Here, FRET cascades displaying an increasing numbers of linear multi-dye arms that converge to a terminal A-labeled apex were directly compared to dendrimeric arrangements where D ratio to each subsequent relay/A dye increased linearly from 2 to 4 across 36 different types of structures and with over 500 control structures assembled in parallel, see Fig. 24. For the linear, unidirectional, bifurcated, HJ, and 8-arm star configurations, [Cy3 → Cy3.5 → Cy5]_*n*_ → Cy5.5 photonic wires were assembled with dyes spaced at 1.5, 1.0, and 0.5 × *R*_0_ relative to each other. Within this structural subset, the 0.5 × *R*_0_ bifurcated structure demonstrated the best *E*_ee_ and AE at 14% and 2.9, respectively, see Fig. 24C. For the 0.5 × *R*_0_ dendrimer subset, the 2 : 1 and 3 : 1 structures demonstrated the best *E*_ee_ and AE at 28% and 3.5 along with 23% and 3.9, respectively. More pertinently, the 2 : 1 dendrimer showed far more energy delivery and sensitization than the 8-arm star, see Fig. 24D, despite the latter having 10 more Cy3.5/Cy5 intermediary dyes than the dendrimer. A terminal enhancement factor (TEF) metric was developed to compare PL intensities of the terminal Cy5.5 A across all FRET constructs regardless of geometry (*e.g.* across linear, bifurcated, HJ, eight-arm star and dendrimers) or D number. This normalizes all the spectral data and then compares the terminal A enhancement to the poorest performing structure, namely, the unidirectional, 1.5 × *R*_0_ construct hosting 1 of each dye at the largest *r*_DA_. This showed that the 3 : 1 dendrimer stood out with a relative TEF of >500 achieved. Data also showed that TEF could still be enhanced by ∼12× even in the linear 1.5 × *R*_0_ constructs despite three intervening FRET steps if D number and geometry were optimized. TEF increased and approached 40 × as the dyes were brought closer to each other within the 0.5 × *R*_0_ dye spacing structure (Fig. 24E). Overall conclusions drawn from this comparative study again confirm that decreasing *R*_0_ while augmenting cross-sectional collection area with multiple Ds significantly increases terminal TEF within dendrimers compared with the linear constructs. Moreover, Förster modelling confirmed that the best results were consistently obtained when multiple interacting FRET pathways (*i.e.*, the dendrimers) were present rather than independent channels (*e.g.* 8 arm star) by which excitons travel from initial D(s) to final A.^252^ From these results, the authors suggested the 2 : 1 dendrimer structure was the most practical since it combined simplicity of design and high assembly efficiency with good *E*_ee_ properties. Pertinently, the Asanuma group recently published a study using DNA junctions that confirmed many of these same findings.^284^ Their DNA junctions displayed pendant arms that incrementally increased in number from 3 to 8 and, using synthetic insertion of d-threoninol, incorporated 6 pyrene donors per arm without visible self-quenching. These arms focused energy to a perylene A located at the center of the junction. AE in a duplex control and the 3- up to 8-way junctions were systematically compared with the 6 and 8 arm systems demonstrating the highest effect; their effective absorption coefficients were also 8.5 times higher than that of perylene. The authors noted that constructs with even-numbered junctions had higher efficiencies than odd-numbered junctions and were able to attribute this to structural instability in the latter assemblies.

**Fig. 25:**
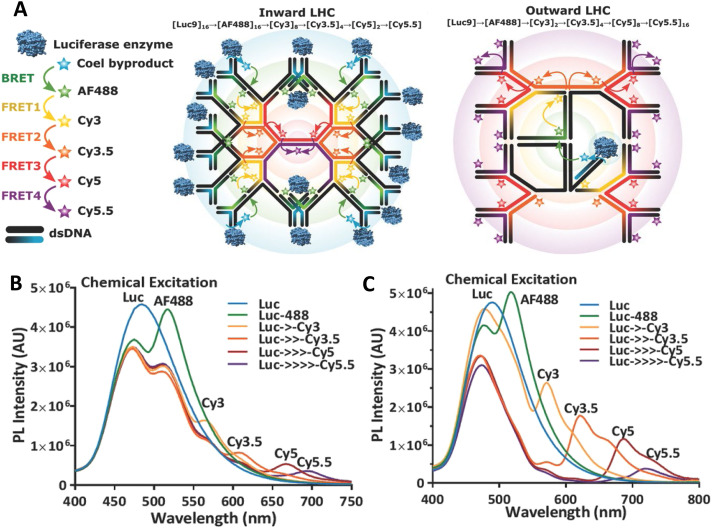
MultiFRET BRET-FRET dendrimers. (A) Left - Schematic of the inward LHC, in which 16 Luc9 are displayed on the outside of the dendrimer and energy is transferred toward the terminal Cy5.5 at the center of the dendrimer through the multistep FRET relay with two Ds per A. Right-Schematic of the outward LHC, in which a single Luc9 is attached in the center and excitons travel from this central Luc9 to the peripheral Cy5.5 dyes *via* the same FRET cascade but with two acceptors per donor. (B) Emission profiles with sequential addition of fluorophores. Representative BRET/FRET progression for the inward LHC as the dyes are consecutively incorporated in the dendrimer structure. Emission from the Luc-only construct (blue) gets quenched as AF488 is introduced with a concomitant BRET sensitized emission from it (green). Effects of subsequent dye additions of Cy3 (yellow), Cy3.5 (orange), Cy5 (red), out to the final acceptor Cy5.5 (purple) *via* multiple dyes, are seen in the respective traces. Arrows in the legends indicate the presence of intermediate fluorophores. (C) Representative BRET/FRET progression of outward LHC as the dyes are consecutively incorporated in the dendrimer structure. Reproduced with permission from ref. 254 Copyright, 2017, John Wiley & Sons.

Dendrimer scaffolding benefits were also applied to composite assemblies where an initial enzymatically-catalyzed BRET step was used to sensitize a subsequent 4-step FRET cascade.^254^ As shown in Fig. 25A, two versions of this composite assembly were tested. The first inwardly-directed version focuses energy from multiple luciferase enzymes (Luc or Luc9 – reflecting the 9 mutations incorporated to improve its function)^285^ arrayed around the dendrimer's periphery. Luc9 catalyzes the initial BRET step that then drives a centrally focused [Luc9]_16_ → [AF488]_16_ → [Cy3]_8_ → [Cy3.5]_4_ → [Cy5]_2_ → [Cy5.5]_1_ LH cascade (LHC). Aside from the initial Luc9 placed at the dendrimer's periphery, there were 2 Ds inserted as sensitizers for each downstream A dye. In contrast, the second LHC had an outward direction of ET and used two As for each D dye with an arrangement consisting of [Luc9]_1_ → [AF488]_1_ → [Cy3]_2_ → [Cy3.5]_4_ → [Cy5]_8_ → [Cy5.5]_16_. From a purely theoretical perspective, these structures should have different factors contributing towards improvement in the *E*_FRET_ that each manifest. In the outward LHC, the D:2A ratio would increase *E*_FRET_ as a result of the proportional increase in A absorption cross-section, which, in turn, would increase spectral overlap. For the inward LHC's 2D:A ratio, basic FRET assumptions might predict less efficient transfer in comparison, but here efficiency would arise from an increase in the probability that any possible FRET step actually happened although it, in turn, minimizes the possibility for each particular pathway. To assemble Luc9 to the DNA dendrimers, key oligos where chemically modified with nickel nitrilotriacetic acid (Ni^2+^-NTA) moieties to facilitate coordination to the enzyme's terminal (His)_6_. The other dyes were incorporated into the DNA using standard synthesis approaches. D–A fluorophores were all placed at <0.5 *R*_0_ spacing, which predicted ∼90% *E*_FRET_ for each step and an overall *E*_ee_ of 60% for 5 sequential ET steps assuming perfect assembly and no energetic losses. The inward 5-step LHCs demonstrated *ca.* 16% *E*_ee_ and 25% *E*_ae_, while the outward versions showed 25% *E*_ee_ and 50% *E*_ae_, see Fig. 25B and C. In the outward LHC, the intermediate dyes were found to contribute very little to the full cascade spectrum due to strong direct excitation of the terminal Cy5.5 A by the initial BRET step. In contrast, the inward LHC relied on substantial contributions from several of the intermediate dyes. A modified derivative of this design placed a dendrimer hosting a Cy3 → Cy3.5 → Cy5 → Cy5.5 FRET cascade extending from the surface of green QDs, which also hosted Luc9 on its surface; the QD acted as both a central scaffold and initial harvester of energy from the Luc9 that subsequently donated excitonic energy to the DNA wire.^286^ By again exploiting a strategy of having multiple A dyes arranged near each relay/D and then also attaching multiple copies of the full dendrimer around each QD, *E*_ee_ of ∼25% was achieved. Overall, these examples serve to demonstrate that biocompatible, chemically-driven designer LH systems can be designed and prototyped while relying only on self-assembly - the most facile and accessible form of nanoscale fabrication.

**Fig. 26:**
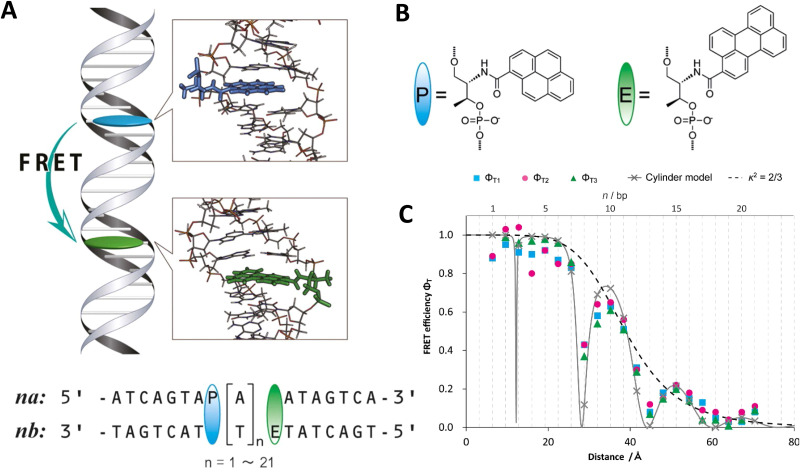
Confirmation of dye position and orientation during FRET within DNA duplexes. (A) Top: Schematic of FRET system used in this study. Bottom: Sequence of FRET dsDNA pair. AT pairs were inserted between P (pyrene as D) and E (perylene as A). P and E moieties were introduced into strands na and nb, respectively, where n indicates the number of inserted A or T residues. (B) Structure of P and E fluorophores were directly conjugated *via* d-threoninol to the DNA strand. (C) Comparison of FRET efficiency obtained from static fluorescence measurement (ΦT1, cyan squares, ΦT2, magenta circles) and time-resolved fluorescence spectroscopy (ΦT3, green triangles) with theoretical values of cylinder model (gray line with cross symbols). The value of averaged orientation shown by dashed line. Reproduced with permission from ref. 111 Copyright 2013 American Chemical Society.

The DNA structures described here not only exploit FRET in various designs, they themselves can also be used as an important tool for elucidating key aspects of the FRET processes, including especially those that require exquisite control over dye positioning. In a brilliant example of this, Asanuma utilized a series of extended separation lengths in DNA duplexes incorporating custom pyrene D and perylene A base surrogates to confirm not only the effect of spacing but, more importantly, dye orientation on ET coupling, see Fig. 26.^111^ Dyes were attached to the oligos *via* a D-threoninol (Fig. 26B) introduced as a custom phosphoramidite and then their *r*_DA_ systematically extended in self-assembled duplexes. Critically, these dyes and this linker scaffolding were chosen as they allow the dyes to become strongly fixed within the DNA duplex and their position relative to each other will further rotate as a function of the DNA helicity in a predictable manner that matches *r*_DA_ separation. As shown in Fig. 26C, *E*_FRET_ dropped as a function of FRET theory as expected when *r*_DA_ increases but then showed significant drops at approximately every 5 bps, which corresponds to a half-turn of the B-type helix. In conjunction with theoretical calculations that treat the DNA duplex as a rigid cylinder with B-type geometry, Asanuma showed that these significant drops coincided to *r*_DA_ values where dipole orientation becomes unfavorable for exciton coupling.^111^ In essence, dye rigidity and inherent DNA twisting allowed the dyes to be repeatedly placed at orientations where their dipoles were unfavorably aligned and this could be seen experimentally. These results again confirm those discussed above with the characteristic ‘bouncing ball’ effect clearly repeated (*cf.*Fig. 8). Confirming dipole orientational effects on FRET was not a trivial thing to show unambiguously and was in a constant state of debate before the advent of DNA technology along with custom DNA synthesis, which allowed for these types of architectures to be achieved.

In experimental work that also used extended separation lengths along a DNA duplex, Cunningham *et al.* systematically studied RET between Cy3 D and Cy5 A, where the D–A separation was incrementally changed from 0 to 16 bp.^169^ The dyes were localized on the duplex using double phosphodiester attachment chemistry. Using steady-state and time-resolved spectroscopy, they found that ET rates were much more sensitive to the distance between D and A than the ET efficiency. For D–A separations less than 10 bp, they also found significant deviations from the predictions of Förster theory, which was attributed to a failure of the point dipole approximation. In contrast to the experiments of the Asanuma group described above, clear oscillation in the ET efficiency with changing dye separation was not observed. An analysis based on structural modeling suggested that the double phosphodiester attachment, while localizing the dyes, leads to a distribution of intercalated and non-intercalated dye orientations. This conclusion is consistent with MD simulations carried out by Stennett *et al.* that found small energy barriers between intercalated and non-intercalated states of Cy3 doubly attached to a DNA duplex.^287^

**Fig. 27:**
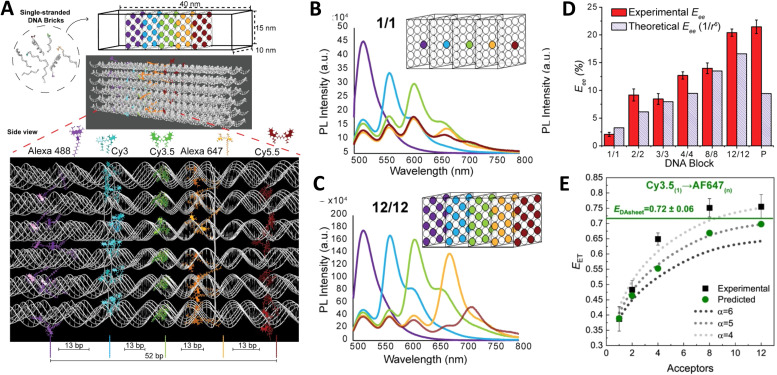
Accessing sheet FRET in a DNA block assembly. (A) Schematic of the rectangular dye-labeled DNA block. Top: The DNA block self-assembles from ssDNA bricks into a prescribed 10 × 15 × 40 nm^3^ cuboidal structure shown schematically and as rendered in UCSF Chimera. Below: An enlarged side view highlighting the positioning of the five 2D planes arranged with an inter dye-plane separation of 13 bp. Each plane can display from 1 up to 12 dye copies with each dye copy positioned on alternating helices. The planes display Alexa 488, Cy3, Cy3.5, Alexa 647, and Cy5.5, respectively, to yield the full sequential initial D → relay → terminal A FRET cascade. (B) Steady-state fluorescence characterization of DNA blocks incorporating 1 (B) and 12 (C) ratios of dyes per plane. Each panel shows the evolution of spectra collected as downstream A dye(s) were added to the next plane in parallel assemblies. Nomenclature indicates the number of copies of each dye type incorporated in the structure, while the box structure schematically depicts this for the final plane-to-plane configuration. (D) Experimentally determined *E*_ee_ estimated from steady-state fluorescence collected from fully labeled block assemblies (AF488 →→ Cy5.5) as the number of dye copies per plane is increased. Theoretical *E*_ee_ assumes each D–A pair has a 1/*r*^6^*E*_FRET_ distance dependency. (E) Experimental and predicted *E*_ee_ values for a single Cy3.5 D to multiple A647 As distributed in a plane. The dashed curves are guides for the1/*r*^α^*E*_FRET_ distance dependency exponent values of *α* = 4, 5, or 6. Predicted *E*_DAsheet_ values show the predicted FRET in the sheet regime shown as the horizontal lines. Reproduced with permission from ref. 103 Copyright 2021 American Chemical Society.

In another example of how DNA-based scaffolding and the positional control it affords can be exploited, Mathur and colleagues utilized a DNA brick-based block assembly to investigate FRET in the sheet regime, see Fig. 27^103^ wherein the ET from a D dye to multiple As arrayed in a 2-dimensional sheet is predicted to show a characteristic 1/*r*^4^ distance dependency rather than the 1/*r*^6^ dependency for a single D–A interaction. The improvement arises from a summation over all the pairwise ET processes. The DNA scaffold was a ‘rigid’ DNA block designed and assembled using Yin Group's brick-based approach,^18^ and it could incorporate up to five 2-dimensional planes with each displaying from 1 to 12 copies of AF488, Cy3, Cy3.5, AF647, and Cy5.5 D, A, or intermediary relay dyes with nearest neighbor dyes on the same helix attached 13 bp apart (Fig. 27A). For the nearest neighbor dyes the predicted *E*_FRET_ was >85%, while next-nearest dyes at ∼26 bp (in alternate planes) had a predicted *E*_FRET_ of <10%. DNA blocks assembled with 1, 2, 4, 8, and 12 copies of each dye per plane were assembled and interrogated with both steady-state and time-resolved spectroscopy, see Fig. 27B and C. Interpreting the observed efficiencies with MD-based numerical simulations it was concluded that the sheet regime was indeed being accessed when the appropriate structural considerations were met, see Fig. 27D and E.^103^ The latter include having an A dye density greater than the distance from the D to the closest A so that it could approximate a continuous 2-dimensional A plane. A pyramidal version of the 5 plane block structure assembled with 12 AF488, 10 Cy3, 8 Cy3.5, 6 AF647, and 4 Cy5.5 dyes, where dyes in each plane were deliberately removed from the block's outer edges inward in each successive plane to yield a configuration with a large AF488 D base plane that constricts into a central terminal Cy5.5 apex plane, was also investigated. Interestingly, this variant demonstrated the same *E*_ee_ values as the densest fully labeled 12-dye block but with one-third fewer dyes present. This suggests that the same sheet benefits could be accessed in other constructs with less dye-labeling present assuming appropriate structural and design criteria are incorporated. Lastly, it is important to note that this demonstration is more than academic as it has ramifications for quantitatively interpreting FRET microscopy results where interactions with densely-labeled cellular membranes are being monitored, designing solar energy harvesters, along with helping improve overall achievable *E*_ee_ in the burgeoning class of related hybrid bioorganic and organic photonic materials.

The plethora of data from the varying examples described above cumulatively confirm the importance of redundant pathways and even redundant copies of each dye in order to maintain a high level of *E*_FRET_ across multiple consecutive ET steps. They also consistently confirm the benefit of concentric LH stages achieved in 1-dimension on flat scaffolds or by using DNA dendrimers to shuttle excitons to a final A. For the dendrimers, these benefits are due to the large absorption cross-section and efficiency of individual ET steps along with the greater dye density that can be achieved in 3-dimensional structures, while still keeping identical D–A dyes separated enough (≥2 nm) so as to not cause quenching. These results also reveal that when expanding beyond organic dyes to other fluorescent materials such as bioluminescent enzymes, rare earth chelates, and QDs, a lot of other factors come into consideration for not only chemically building the DNA based structure but also in how to access FRET within it. These can include accounting for significant differences in relative size (QD *vs* dye, Luc *vs* dye or dsDNA) along with incorporating different bioconjugation chemistries. Additionally, the unique photophysical properties of each material can and will come into play including especially absorption profiles and excited-state lifetimes. DNA structural complexity also needs to be weighed against potential formation issues. Linear ds constructs can assemble with almost unity efficiency while very dense dendrimers can have much lower yields and require complex hybridization protocols that are instituted over a long formation time.

Although not discussed in detail in this section, the above examples also confirm different forms of modeling being frequently applied to aid the interpretation of results and their use is now becoming an accepted part of the FRET analytical toolbox.^118^ MD and other structural or atomistic analyses help with defining the relaxed state of a DNA scaffold and the potential favored position of dye(s) within that configuration; this helps with estimating expected *E*_FRET_ while also providing useful information about dipole considerations.^103,173^ Numerical simulations in this context allow the upper limit of achievable *E*_FRET_ in a given structure to be estimated and to assess various non-idealities (*e.g.* missing dye, unfavorable dipole orientation, *etc.*) that may be contributing to the lower efficiencies often observed experimentally.

### 4.3. Dynamic FRET structures

In direct contrast to the functionality seen with many of the previous structures, where the DNA acted, for all intents and purposes, as a static optical breadboard to assemble and then host the FRET participants and allow in-depth analysis of ET, changes in FRET are also often used in the converse format to report on rearrangements in DNA structures. This forms the impetus behind some of the structures described in this section where the goal is very often that of transducing a sensing or signaling event or demonstrating control over some other process. The ability to alter or reconfigure an assembly in a fully preprogrammed manner by strand displacement and other dynamic DNA-driven processes represents perhaps one of the most underutilized aspects of DNA nanotechnology and, beyond sensing, it is commonly applied to creating logic gates for rudimentary information processing^288–293^ Another common application in the current context is that of a dynamic ‘switch’ to control or alter FRET pathways. If designed correctly, such switches can function as toggles for moving back and forth between two or more designated optical states. A very brief cross-section of pertinent examples is provided here in order to highlight what dynamic DNA structures have to offer for controlling or altering the FRET processes they may host.

**Fig. 28:**
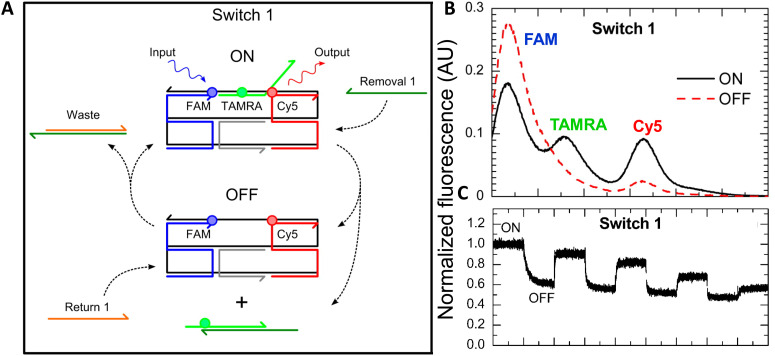
Cyclic switching of dynamic FRET-based transmission lines by DNA strand invasion. (A) When Switch 1 is in its ON state, the TAMRA-functionalized control strand (green) is attached to the scaffold (black), resulting in an intact transmission line. The Removal 1 strand (dark green) hybridizes with the control strand, removing the TAMRA dye from the scaffold and interrupting FRET, which switches the device to its OFF state. To restore FRET and return the device to its ON state, the Return 1 strand (orange) hybridizes with the Removal 1 strand, releasing the control strand and allowing the TAMRA dye to rejoin with the scaffold. (B) Full fluorescence spectra for Switch 1 in its ON and OFF states. (C) Switch reaction kinetics data demonstrating changes in terminal Cy5 A fluorescence intensity due to control strand removal and restoration. Reproduced with permission from ref. 201 Copyright 2012 American Chemical Society.

Graugnard and colleagues sought to exert dynamic control over exciton pathways or FRET-based transmission lines in order to enable information processing using DNA switching devices based on strand invasion.^201^ Each of the switches they implemented consisted of a DNA scaffold strand hybridized into a serpentine architectural array with three staple strands (Fig. 28). These staple strands, in turn, were made of eight orthogonal sequence domains each 14 nucleotides long and separated by crossovers. A 4th DNA strand designated as a “control” strand was then used to regulate the flow of excitonic energy within a given switch. One of the staple strands contained the FAM input D dye while another staple strand contained the Cy5 A output dye and these were separated by an intermediary strand with a TAMRA dye. With all dyes present and 7 bps separating each dye in the FAM → TAMRA → Cy5 relay, the switch is in the ON state, see Fig. 28A. A ‘Removal’ strand is then used to hybridize to and displace the TAMRA containing strand *via* toehold-mediated displacement, significantly decreasing the FAM → Cy5 FRET; this is the OFF state (Fig. 28B). Finally, a ‘Return’ strand binds Removal strand and separates away from the TAMRA strand allowing for the regeneration of ON by the same sequence. Switch 2 consists of a functional homologue of this 3-dye structure with an added dark quencher that disrupts the ET and which is then removed and put back to accomplish the switching of states. Concerted cycling of the switches was demonstrated over 3 hours with a slow loss of gain (Fig. 28C). In terms of operational performance, the first switch showed a 6% loss in gain per cycle yielding an overall *E*_FRET_ of ∼50% while the second switch showed a 2% gain with an overall *E*_FRET_ of ∼20%. Switching to appropriate orthogonal sequences with different dyes and FRET pathways in one of the switches would create the intriguing possibility of operating both together and provide the option of accessing up to 4 states with its inherently higher complexity. Far more complex AND gates have also been prototyped and incorporated components that functioned in a manner similar to these excitonic switches. To yield these excitonic transmission lines, 2-D nanobreadboards (assembled from 6 helices × 94 bp) functioned to position 4 dye chromophores in what was designated a zigzag (corresponding to an AND logic gate 1) or in a different quasi-linear (corresponding to AND logic gate 2) array.^200^ Toehold-mediated strand displacement, ssDNA invasion, and restoration strands were utilized to either remove or restore strands from or back onto the nanobreadboards, respectively, to enable isothermal reversible and repeated switching. The Tinnefeld Group analogously demonstrated that the ET paths on an underlying DNA origami breadboard could be controlled by what they referred to as ‘jumper” dye, which directed the exciton energy between two alternative output dyes that had either red or IR emission.^294^

**Fig. 29:**
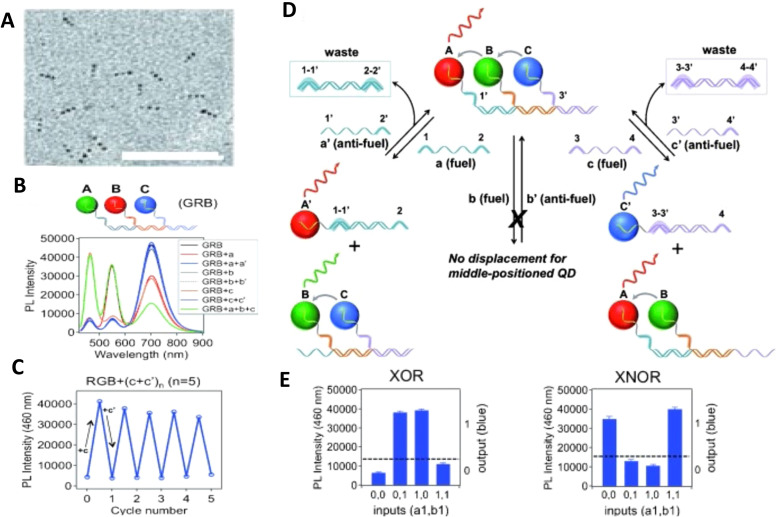
DNA functionalized QDs for molecular logic. (A) TEM of the DNA functionalized ternary complex consisting of ZnHgSe (red), CdTe (green), and ZnCdSe (blue) QDs. (B) Ternary complex that has undergone disassembly and reassembly through strand displacement reactions. (C) Multi-round strand displacement reactions of RGB ternary complex with fuel and anti-fuel DNA. (D) Illustration of disassembly and reassembly of ternary QD complex through strand displacement reactions. (E) QD photonic logic gates operated by strand displacement reactions to yield QD PL output signals corresponding to the definitions of XOR and XNOR logic gates. Reproduced with permission from ref. 295 Copyright, 2014, John Wiley & Sons.

Extending from fluorophores to FRET processes based on QDs, He *et al.* developed DNA assemblies capable of dynamically incorporating multiple different colors of QDs and which functioned as a FRET-based QD computing system, see Fig. 29.^295^ Chimeric DNA sequences were directly coordinated to the QD surfaces *via* a phosphorothioate domain utilized for QD passivation while an adjacent phosphate domain drove complementary interactions. Using both DNA fuel and anti-fuel strands as inputs, different structural rearrangements could be accomplished and then repeatedly toggled as well. Ultimately, this allowed for a set of 7 elementary logic gates (consisting of XNOR, AND, OR, NAND, NOR, INH, XOR) to be realized using a working series of binary/ternary QD-labeled complexes and as operated by multiple programmed strand displacement reactions. A further set of logic gates could also be integrated together to yield a half-adder circuit. Based on Förster theory, *E*_FRET_ of *ca.* 76–82% was predicted between each of the QD pairs while experiments revealed this to be far less with an actual range of ∼13% up to 85%.^295^ It is probable that some of the aforementioned issues with utilizing QDs as As in steady state fluorescent experiments, especially for other QD Ds, affected transfer efficiency due to substantial direct excitation components. One fascinating extension of this approach would be to use DNA sequences in a dual-hatted modality where they program the synthesis of different sizes of QDs directly and then, further drive them to hybridize together into preprogrammed designer lattices.^296^ This would obviate the need for some of the initial QD-DNA linkage chemistry.

**Fig. 30:**
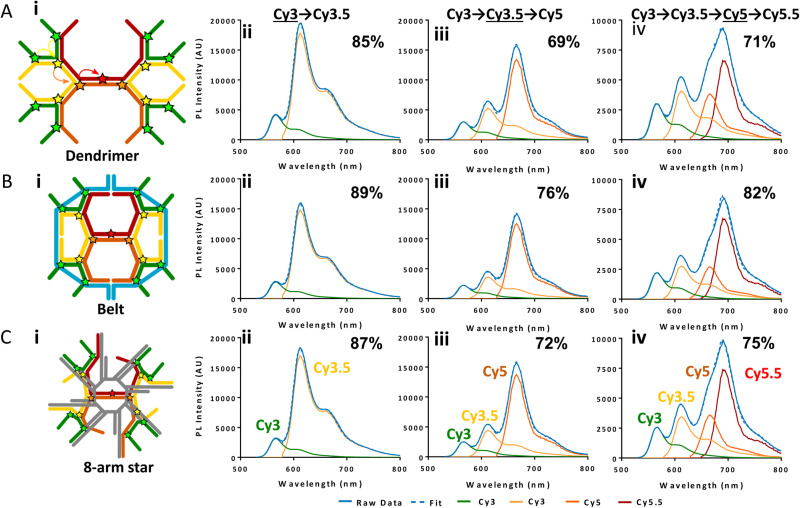
Spectral analysis of representative external modification-based approaches. Each row represents one structural conformation. (A) The native DNA dendrimer was compared to (B) the Belt-6 dendrimer, with the smallest bridge length (6 bp) and the (C) star grabber with the ball conformation. The first column (i) displays a schematic of the dendrimer and what each modification is intended to do. Structures were analyzed with the (ii) two dye (Cy3 → Cy3.5), (iii) three dye (Cy3 → Cy3.5 → Cy5), or (iv) four dye (Cy3 → Cy3.5 → Cy5 → Cy5.5) cascades present. Black arrows indicate FRET steps. FRET efficiencies from penultimate donor (underlined) loss for each subsequent dye addition are shown in the upper right corner in each panel: (ii) Cy3 donor loss; (iii) Cy3.5 donor loss; and (iv) Cy5 donor loss. Raw data is shown as the solid blue line, with the reconstructed spectral fit as the blue dashed line. The contributions of each dye are separated by color; Cy3 (green), Cy3.5 (yellow), Cy5 (orange), and Cy5.5 (red). Reproduced with permission from ref. 299 Copyright 2017 American Chemical Society.

Rather than design a new DNA assembly for each intended logic application, Buckhout-White and coworkers focused on one generalized DNA design to see how far it could be extended towards providing multiple different logic gates or states in a single architecture.^297^ They demonstrated dye-labeled 3-arm triangular DNA scaffold switches, where each of the arms could be assembled individually in different combinations. Here, the linkages between each of the arms could be removed in response to a toehold-mediated strand displacement initiated by adding the correct target complement and then replaced by using a rapid annealing protocol. Rearrangement of the base structure allowed for alterations in *E*_FRET_ between each of the pendant dyes (either 2 or 3) and this provided for a dense library of spectral signatures with unique properties; the latter are what formed the underpinning for subsequent establishment and use of photonic logic gates. The underlying DNA assembly was itself also designed so that different linker lengths could join each arm and function as the inputs; these different sized inputs could also function independently of each another thus allowing for an enhancement in the range of molecular gates. This platform's functionality was demonstrated in configurations that gave rise to a series of 1-, 2-and 3-input Boolean logic photonic gates such as INHIBIT, OR, AND, along with a device that functioned as a photonic keypad lock.^297^ Further work was pursued to optimize and extend sensor function *via* several different approaches: (i) redesign of the dye-choices and connecting linkers *versus* that found in the original structure; (ii) altering of the mechanism that gave rise to distance modulation between each of the arms; and (iii) a rearrangement of the signaling dyes to now be within the ss portion of the gate assembly.^298^ FRET properties were improved by the first approach along with the ability to switch between different Boolean logic gates (such as going from an INHIBIT 1 to an Enabled OR) by changing dyes. The final approach demonstrated the most versatility by allowing for the highest magnitude changes in FRET along with access to the ability to undergo repeated toggling and resets that would be expected with multiple sequential sensing events. The actual switching mechanism itself was implemented in an isothermal manner with nearly equal or stoichiometric amounts of inputs and their complements.^298^ This last attribute could potentially allow for function as a sensor readout without requiring support from a thermal cycling device. With a similar goal of pursuing a more universal DNA platform in mind, Brown *et al.* investigated four different modification strategies to effect dynamic manipulations of an underlying DNA structure.^299^ Here, a design based on a previously developed dendrimer scaffold was exploited as the testbed.^252^ A branching dendrimer based upon an underlying 2 : 1 architecture allowed for the dyes to be organized into a FRET-based antenna structure; here initial excitons generated on the multiple surrounding Cy3 D dyes displayed at the periphery of the assembly could be transferred inward through a sequential series of Cy3.5 and/or Cy5 A/relay dyes to the single terminal Cy5.5 A found at the structure's core. Two structural rearrangement mechanisms were tested with the first being based on toehold-mediated strand displacement or strand replacement, which primarily affected the transfer efficiency of individual steps. The second approach implemented either a belting mechanism or an exogenous 8-arm star nanostructure that directly interacted with the dendrimer to compress the nanostructure and thereby modulate its spectral response through an enhancement in parallel, see Fig. 30. Overall, this second strategy proved to be more efficient in terms of altering FRET as its effects were globally implemented across the entire structure in parallel. Advantages originating from the underlying dendrimeric design and also accessed by utilizing this approach include good *E*_FRET_ at every step, high *E*_ee_, large spectral separation between the initial D and final A emissions for signal transduction, and an inherent tolerance to defects. As mentioned above, derivatives of this dendrimeric structure have also been demonstrated with chemically driven BRET excitation.^254^ If addition of substrate with the concomitant extra BRET step or use of direct FRET excitation are also considered as variables, then the complexity of accessible logic functions could be further extended quite significantly. The time component inherent to FRET cascades incorporating long-lifetime rare earth chelated Ds can be similarly used to extend the number of variables available to such structures.^253,300,301^

**Fig. 31:**
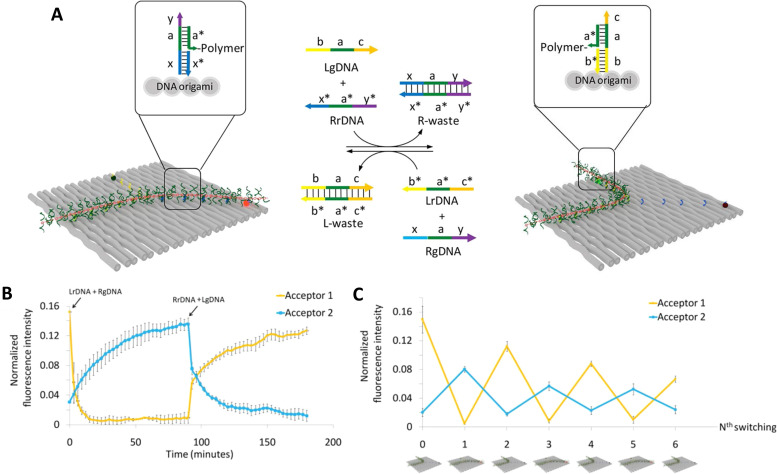
Switching of single polymer conformations on a DNA origami scaffold. (A) Nanomechanical switching of poly(APPV-DNA) on DNA origami based on toehold-mediated strand displacement. Schematic illustration of guiding/removing ssDNA-assisted switching of poly(APPV-DNA) conformation. Complementary sequences are shown in the same color and denoted as X and X*, respectively. The arrowheads represent the 3′ end of DNA. (B) Time-dependent FRET measurement of nanomechanical switching. For FRET experiments, the initial state of poly(APPV-DNA) was the L-turned pattern. Fluorescence intensities of A 1 measured at its maximum emission wavelength, 618 nm (yellow line), and A 2 measured at its maximum emission wavelength, 670 nm (blue line), were used to follow polymer switching through energy transfer from the polymer to the acceptors. (C) FRET measurement of nanomechanical switching showing six successive events of programmed poly(APPV-DNA) switching. Reproduced with permission from ref. 306 Copyright 2016 American Chemical Society.

One application where exploiting dynamic changes in FRET has proven particularly useful is that of indirectly monitoring DNA walkers.^302,303^ These synthetic nanoscale machines are reminiscent of biological protein motors and are meant to move in a directed manner as specified by a track. Both the walker itself and the tracks are typically DNA assemblies and the motion and directionality are driven by addition of fuel and anti-fuel DNA or RNA strands, which induce toehold-mediated strand displacement that displaces a given DNA strand on the walker allowing it to find and bind to another site on the track in an ordered manner. Both ensemble and smFRET approaches have proved useful to monitoring the stepwise movement of these constructs.^304,305^ Here, the changes in FRET between dyes attached to the walker and the track reflect the step motion while also allowing for some estimates of nanoscale distance traversed in some cases. In a different application that relied on similar monitoring of FRET changes, the Gothelf lab described the development of a nanomechanical device that allowed switching of the position of a single-molecule DNA conjugated polymer.^306^ Poly[5-methoxy-2-(3-sulfopropoxy)-1,4-phenylenevinylene] polymer was first chemically functionalized with short ssDNA strands (APPV-DNA) that extended from the backbone of the polymer using modified solid state oligonucleotide synthesis. These short strands served as handles allowing the DNA polymer conjugate to be aligned on a DNA origami structure in three well-defined geometries (straight line, left-turned, and right-turned pattern) as driven by DNA, see Fig. 31A. Switching the polymer between left- and right-turned conformations was based on toehold-mediated strand displacement and signaled by FRET between the polymer and two different organic dyes (Alexa Fluor 594 and 647) positioned in close proximity to the respective patterns (Fig. 31B). Using this method, the polymer conformation was switched between configurations successively six consecutive times (Fig. 31C).

There are many other examples of dynamic FRET changes being used to report toehold-mediated rearrangements in complex DNA structures.^307–311^ Rearrangement of DNA scaffolds displaying discrete FRET pairs or even multi-dye cascades have also been utilized to monitor other types of quite disparate biological activities. De Ruiter and colleagues utilized a 120 nucleotide DNA structure hosting an Atto488 → Atto566 → Atto647 FRET cascade with each dye placed at significant distances from each other (≫*R*_0_) as a sensor for detecting assembly of cowpea chlorotic mottle virus formation into monodisperse 22 nm icosahedral particles.^312^ Viral encapsulation of the DNA structure not only increased FRET 8-fold, it also induced 2-step FRET across the entire cascade. In an attempt to leverage the power of DNA Boolean computation to discriminate between actual biosensing events, Bui synthesized a two-signal sensor incorporating a chimeric peptide-DNA substrate as a protease-to-DNA signal convertor to transduce protease activity through DNA gates allowing the latter to differentiate between the activity of different initial input hydrolytic enzymes.^313^ In this system, Cy3 A dye-conjugated peptide-DNAs were assembled onto QD Ds as an initial input gate. Trypsin or chymotrypsin, enzymes commonly used for prototyping purposes due to their robustness and differing sequence specificities,^314–316^ cleaved their target peptide sequences on the QD thereby altering *E*_FRET_ with the QD to provide the first signal while also freeing a DNA output to interact with a downstream DNA tetrahedral output gate. Output gate rearrangement resulted in FRET sensitization of a new A dye as the second signal or bit of information. Processing multiple distinct and/or orthogonal bits of information for a sensing outcome will provide more confidence in any nanoscale diagnostic device as opposed to relying on a single change especially for discriminating between different targets. Coupling other substrates to DNA that respond similarly could help target other types of enzymes beyond just these 2 prototypical proteases. Indeed, it is well worth considering that almost every bacterial and viral pathogen incorporates a protease in a key part of its lifecycle or critical infective step;^317,318^ thus monitoring and discriminating between their activity can be quite informative in this new era of pandemic threats. In the context of DNA nanotechnology, peptide substrates for proteases can be obtained fused to the DNA by several different chemistries.^21,319,320^ Not only can FRET reflect the underlying dynamic structural rearrangements in such nanoscale assemblies in almost real-time, it can also uniquely provide concomitant information on separation distances in these structures.

A fascinating approach taken by the Zhen and Huang Labs extended the capability of DNA-only nanostructures to report on enzymes by utilizing aptamers as the recognition element. Using thrombin for the proof of concept, when two aptamers come together on the target enzyme they combine to create a catalyst capable of toehold displacement on a 3-dye MPW, turning off the FRET signal. With the proper design and addition of a fuel strand the catalyst could disrupt multiple MPWs. The use of the MPW improved the sensitivity to as low as 5 nM thrombin detection. Clearly, if the requisite aptamers are obtained, this approach could be extended to other targets relatively easily.^321^ Although the above examples represent only a small cross-section of examples where dynamic DNA rearrangements are used in conjunction with FRET monitoring, it is readily apparent that this potential application space is quite large, vastly underexploited, and will only continue to grow.

### 4.4. Extending energy transfer with homoFRET

**Fig. 32:**
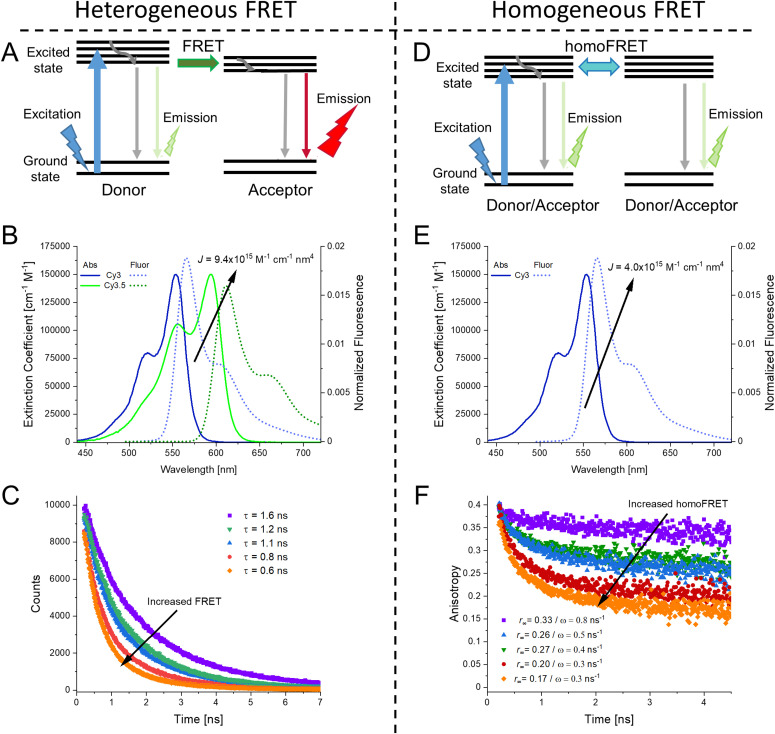
Comparison of heterogeneous FRET and homogeneous FRET. (A) Jablonski diagram of heteroFRET, showing transfer from D to A and its subsequent emission. (B) Spectral overlap of Cy3-Cy3.5 D–A heteroFRET pair resulting in a *J* of 9.4 × 10^15^ M^−1^ cm^−1^ nm^4^. (C) Fluorescence lifetime of a donor molecule. As heteroFRET increases the average fluorescence lifetime, *τ*, decreases. (D) Jablonski diagram of homoFRET, the identical molecules can function as both D and A now. (E) Spectral overlap of Cy3-Cy3 D–A homoFRET pair resulting in a relatively smaller *J* of 4.0 × 10^15^ M^−1^ cm^−1^ nm^4^, though this is still reasonably good value for efficient FRET. (F) Fluorescence lifetime anisotropy of emitting molecules. See eqn (20) and (21) for how fitting values were derived. Fluorescence lifetimes are unchanged in the presence of homoFRET and, as such, the fluorescence lifetime anisotropy is necessary.

Homogenous FRET (homoFRET) encompasses the same formalism that describes heteroFRET, with the distinction being that the D and A molecules are now chemically equivalent (*i.e.*, identical) and as such their photophysical characteristics, including emission wavelength and fluorescence lifetime, cannot be used to distinguish between them in this configuration, see Fig. 32. Furthermore, due to the indistinguishable nature of the D–A, it cannot truly be said that there is a distinct D–A pair as both can function as D and/or A, and, in fact, multiple stochastic transfers can and typically will occur between the two. This lack of ET directionality, meaning there is no downhill D–A energy cascade, along with the more involved nature of homoFRET quantification, which generally requires anisotropy measurements, results in some eschewing the use of homoFRET within their DNA-based photonic systems or simply just ignoring its presence. However, homoFRET can provide considerable advantages for such systems as detailed below. The two principal advantages in using homoFRET within the current context are: (1) conservation of exciton energy; and (2) the ability to access dyes with desirable photophysical properties such as high QYs and long fluorescence lifetimes. We begin by providing a primer on the general considerations that users should take into account when incorporating homoFRET systems or modules within their DNA-templated photonic networks.

#### 4.4.1. Considerations when using homoFRET

As shown in eqn (2), one of the key components that strongly influences the Förster distance (*R*_0_) is the spectral overlap integral (*J*). This holds true for homoFRET as well and to achieve optimal transfer efficiency, the chosen homoFRET molecule's absorbance cross-section must overlap as much as possible with its own emission spectra. In a homoFRET configuration, *R*_0_ can generally be optimized by choosing a fluorescent species with the smallest Stokes shift possible (see Fig. 32E). The Stokes shift is the energetic difference in the peak absorbance and emission and correlates with the vibrational relaxation and solvent reorganization that the molecule undergoes upon excitation; the greater amount of relaxation and reorganization – the greater the Stokes shift.^131^ In contrast, heteroFRET imaging and assays desire spectrally distinct dyes, *i.e.*, the A absorption should be small at the wavelength where the D is excited. This usually means that the peak of the A absorption band is sufficiently red-shifted from the peak of the D absorption band to avoid spectral cross-talk between D excitation and A emission (*i.e.* minimizing the direct A excitation component) (Fig. 32B). Thus finding optimal homoFRET emitters may require a little more research. Interestingly, two of the more popular dyes utilized in both hetero- and homoFRET configuration are Cy3 and Cy3.5, which, even with their own intrinsic ∼20 nm Stokes shift, still have 4.5–5.0 nm homo-*R*_0_ values due to their high QYs and extinction coefficients.

A second consideration is also perhaps the most famous issue associated with implementing homoFRET, namely, that it is best described by using the mathematical random walk model.^322^ This reflects that it is a stochastic process in which an object moves in a series of random steps. Assuming symmetric positioning of the fluorophores, the probability of moving to any contiguous position is therefore equivalent,^323^ and this results in the lack of ET directionality within homoFRET systems. The critical point to be appreciated when designing homoFRET sections within a DNA structure is that the positive attributes, including large absorption cross-sections and high transfer efficiency increase with the number of fluorophores present, which will in turn, minimize the probability of any particular fluorophore being excited while increasing the potential number of ET steps along with possibilities of random directionality. As homoFRET sections are primarily incorporated into MPWs and similar devices as relays or antennas for LH systems, it is generally desired that they have a terminal transfer to a distinct A or even a chemical/reaction center through heteroFRET. In practice, the greater the number of identical (homo)fluorophores – the less efficient this final crucial step will be, yet subtle design choices can optimize funneling to and from the conjoined heteroFRET steps as shown in the examples below.

A third important consideration is in the methodology required to quantify homoFRET. While heteroFRET can be readily distinguished by changes in D/A fluorescence lifetime (see example in Fig. 32C) and intensity as seen in eqn (6), the presence of homoFRET will result in no visible changes in these parameters. To determine homoFRET efficiency fluorescence anisotropy measurements are typically required, see Fig. 32F.^324,325^ Though many modern fluorescence spectroscopy systems have some automated or other type of anisotropy capabilities, the measurements are generally more time-consuming than direct steady-state or lifetime fluorescent measurements.^326^ Also of potential concern is the limited dynamic range and relatively small signal to noise of the homoFRET experiment. Furthermore, for heteroFRET quantification, the D and A should be weakly coupled so that they do not change each other's optical properties. This condition should also hold for homoFRET anisotropy experiments. In homoFRET, it is the ‘base’ anisotropy of the dyes that one assumes is unmodified upon interaction, except, of course, due to the ET in question. As the anisotropy of the dyes depends on the rotational freedom of the entire system, if there is considerable size or rigidity changes in the system upon creation of the homoFRET section this must be taken into account when trying to quantify subsequent ET.^327^ Once the appropriate anisotropy values are obtained, the ET efficiency can be determined by the following equations in a manner akin to heteroFRET.^324^ The homoFRET rate is given by ω (equivalent to the FRET rate in eqn (1)), which in combination with *τ*, the fluorescence lifetime, can, in turn, provide the homoFRET efficiency (*E*_hF_).^324^ The time-resolved anisotropy decay, *r*(*t*), due to homoFRET is given by:

20 *E*_hF_ = *ω*/(*τ*^−1^ + *ω*)

21 *r*(*t*) = (*r*_0_ − *r*_∞_)e^−2*ωt*^ + *r*_∞_

where, *r*_0_ is the maximum initial anisotropy determined by the angle between the excitation and emission dipole and *r*_∞_ is the anisotropy at infinite time, which should go to zero for a fully and freely-rotating emitter.

#### 4.4.2. DNA photonic wires incorporating homoFRET

**Fig. 33:**
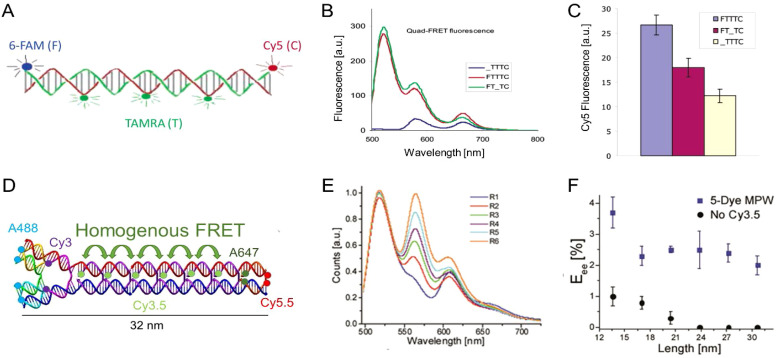
HomoFRET incorporating MPWs. (A) Schematic of dsDNA based MPW with a 3 TAMRA homoFRET relay. (B) Fluorescence spectra of homoFRET MPW and corresponding controls. (C) Cy5 fluorescence of spectra in panel B. Reproduced with permission from ref. 328 Copyright 2004 American Chemical Society. (D) Schematic of DX tile-based DNA MPW with a 6 Cy3.5 homoFRET relay. (E) Fluorescence spectra of MPW as the number of relay steps increased, R1–R6, signifies 1 Cy3.5 to 6 Cy3.5 respectively. (F) *E*_ee_ values as a function of the length of the MPW. Reproduced with permission from ref. 147 Copyright, 2016, John Wiley & Sons.

As highlighted previously, a primary advantage of DNA-templated MPWs is their versatility in integrating various dyes, often times in a modular approach. Given the inherent structure of linear photonic wires, researchers became interested in physically extending MPWs to their maximal length and studying the transfer efficiencies manifested on these structures with the goal of learning how to extend the number of ET steps, the distances over which ET could take place, and improving the overall *E*_ee_. Table 5 highlights a list of several representative DNA MPWs where homoFRET was used as an integral part of the overall ET processes within a given structure. The table demonstrates some recent advancements in homoFRET MPW design and efficiency but leaves apparent that many further improvements are still needed for future applications. Interestingly, in one of the first published works on DNA templated MPWs, Ohya demonstrated the use of a TAMRA homoFRET relay (which they referred to as energy migration) between an eosin D and a Texas Red final A.^240^ By using a two-dye relay instead of a single TAMRA, they could extend their MPWs from 6.6 nm to 10 nm in length with their *E*_ee_ only decreasing marginally from 24% to 22%. Importantly, this study also demonstrated that covalently bound organic dyes could be directed by DNA templates to act as very efficient homoFRET relays, but they did not proceed beyond a 2-dye relay. Subsequent work from other groups focused on formalizing the design rules for 1-D homoFRET relays. From the Quake Lab, MPWs using dsDNA extended the homoFRET relay to 3 dyes, in this case using 6-carboxyfluorescein as the initial D, TAMRA again as the relay section, and Cy5 as the final A, see Fig. 33A–C.^328^ Beyond pushing the homoFRET relay module itself to 3 dyes with an *E*_ee_ of ≈20% for a 12 nm MPW, the more important aspect was the use of a simple rate balanced ET model to properly predict the efficiency in short homoFRET relays. One limit found associated with the design was the need to label a single DNA strand with multiple dyes, which generally leads to a low product yield and can be quite costly.

**Table 5:** Overview of representative homoFRET MPWs

DNA structure/estimated length	# of homoFRET dyes	HeteroFRET dyes	Dye conjugation	*E*_ee_	Notes	Ref.
25 bp ds triplex (∼8 nm)	2 TAMRA	Eosin (D) Texas Red (A)	Covalent	∼22%	Pioneering study in this field	240
dsDNA/12 nm	3 TAMRA	6-Carboxyfluorescein (D) Cy5 (A)	Covalent	20%	Waveguide application	328
DX tile/31 nm	6 Cy3.5	A488 + Cy3 donors	Covalent	2%	Linear adjustable homoFRET module	147
		Alexa647 + Cy5.5 acceptors				
DX tile/24 nm	6 Cy3.5	Cy3 (D) and Alexa647 (A)	Covalent	6%	At 5 K in PVA film	146
Origami breadboard/16 nm	3 Cy3	A488 (D) and Cy5 (A)	Covalent	3%	Triangular homoFRET relay	329
Origami helix bundle/29 nm	10 Cy3.5	A488 (D) and A647 (A)	Covalent	1%	homoFRET antenna	149
Origami helix bundle/29 nm	6 Cy3.5	A488 (D) and A647 (A)	Covalent	59%	At 5 K in PVA film	149
dsDNA/16 nm	YO-PRO-1	Pacific Blue (D) and Cy3 (A)	Intercalation	20%	0.4 YO-PRO-1 dyes per bp	330
dsDNA/26 nm	YO	Pacific Blue (D) and Cy3 (A)	Pyrrole-imidazole polyamide directed site-specific binding	14%	Site-directed binding of YO dyes in the DNA scaffold	331

In 2016, a DNA MPW based on the DX tile design, which permits a modular approach to dye integration, was described. This structure allowed for homoFRET relays of up to six dyes, constituted from either Cy3 or Cy3.5 depending on the design, within MPWs containing either two or four additional heteroFRET steps.^147^ Along with demonstrating that DNA MPWs could be extended to 31 nm with *E*_ee_ values of 2% using four heteroFRET steps within a six Cy3.5 homoFRET relay (see Fig. 33D–F), the authors analyzed the system using both a 1-D symmetric random walk with reflecting and absorbing edges^332^ as well as a kinetic model based on the FRET rate equations. Experimentally, the *E*_ee_ were lower than the predicted values based on the simplest structural and FRET assumptions, yet small adjustments to the parameters obtained from component characterization, such as substituting experimentally determined dye separation distances as compared to that from assumed DNA positions, allowed both models to fit the data reasonably well. This was another crucial result as it confirmed the viability of modeling for extended homoFRET relays.^147,323^ Another important point realized was that initial dye selection was critical; efficient homoFRET relays (*i.e.*, with long excited state lifetimes and large homo-*R*_0_) improve transfer efficiency even over the directionality provided by heteroFRET. The efficiency of the homoFRET relay module could be even further improved by modification of the environment around the DNA nanostructure when depositing it in polyvinyl alcohol (PVA) films as well as cooling to cryogenic temperatures; this resulted in up to a 2.5-fold increase in *E*_FRET_ by removing many of the competing, parasitic, and non-radiative decay pathways.^146^ This experimental evidence also supported the hypothesis that homoFRET sections are particularly sensitive to increases/decreases in dye QY.

**Fig. 34:**
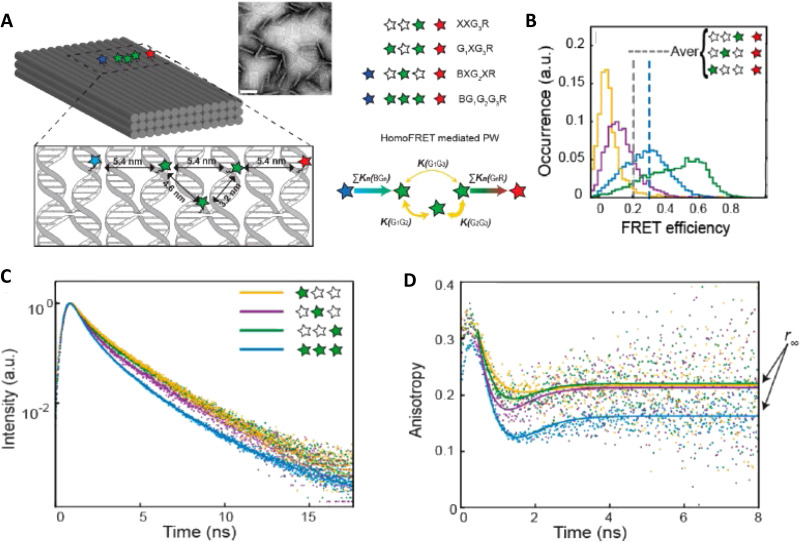
HomoFRET on an origami breadboard. (A) Schematic of DNA origami breadboard containing triangular homoFRET relay. (B) Single particle FRET of the Cy3 relay dye to the final Cy5 accepter. (C) Fluorescence lifetime of Cy3 relay positions, it is notable that the lifetimes are similar but not indistinct as might be expected. (D) Fluorescence lifetime anisotropy of the same Cy3 configurations seen in C where localized influences on individual dyes were noted. Reproduced with permission from ref. 329 Copyright 2017 American Chemical Society.

**Fig. 35:**
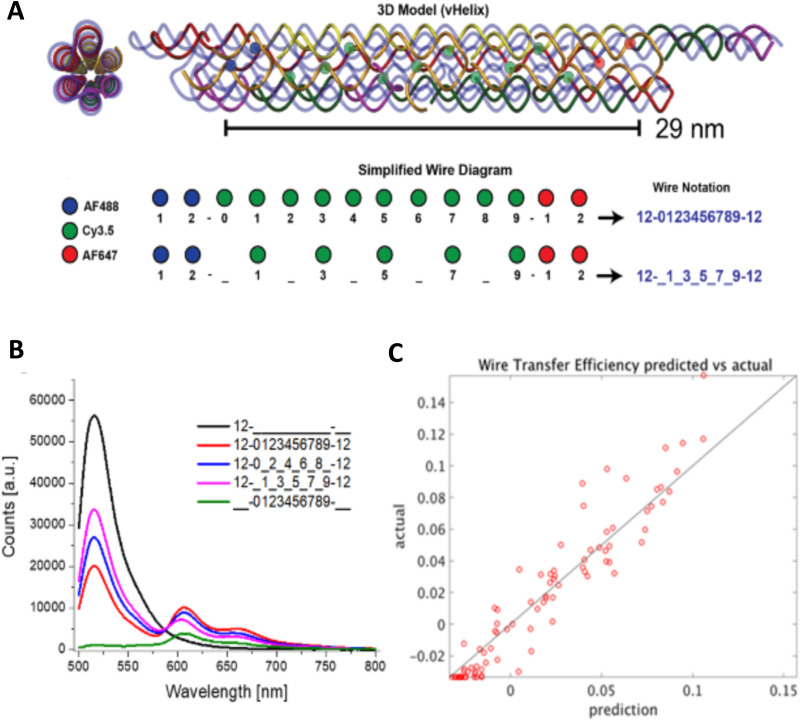
Extending homoFRET in a DNA bundle. (A) Schematic of six helix bundle origami MPW highlighting how different variants with different densities of homoFRET relay dyes can be assembled. (B) Solution based fluorescence spectroscopy of varying dye configurations within the MPW. (C) Random forest machine learning predicted wire transfer efficiency in comparison to experimental results. The trend highlights the general capability of models to describe the systems yet the deviations show that empirical confirmation is still optimal. Reproduced with permission from ref. 149 Copyright 2020 American Chemical Society.

Nicoli *et al.* also utilized a three dye Cy3 relay between an A488 D and Cy5 A within a 2-D DNA origami breadboard system and were able to get 3% *E*_ee_ over 16 nm distances.^329^ They confirmed the utility of homoFRET relays, but more uniquely, using single-particle microscopy, provided some of the first evidence that the assumption of equivalent fluorescent moieties within homoFRET relays may be contradicted by the variable nano-environments that each dye experiences within the DNA nanostructures, see Fig. 34. To this end, they reported changes in the fluorescence lifetime and QY of the Cy3 upon undergoing homoFRET. Klein and colleagues pushed the homoFRET DNA MPW concept even further by exploiting DNA's modularity to study a six-helix DNA origami MPW with up to ten Cy3.5 relay dyes placed between A488 Ds and A647 As, which functioned as a variable density homoFRET module (Fig. 35).^149^ They created a database of over 500 individual steady-state measurements that varied the combinations of different possible Cy3.5 relay positions and correlated that to the observed *E*_ee_ values. Consistent with what Nicoli observed, the Cy3.5 had variable effects on the *E*_ee_, but the subtleties in the differences were such that a human user would be hard-pressed to distinguish them. Monte Carlo and random-forest machine learning methods allowed for an analysis of homoFRET transfer as a function of Cy3.5 density and arrangement. By eliminating inefficient positions, a MPW with six optimally placed Cy3.5 relay dyes was demonstrated to be capable of ET over 29 nm with a remarkable 59% *E*_ee_ achieved when again assembled in a PVA film and placed under cryogenic conditions.^149^ This also confirmed that greater dye density does not always result in better exciton transfer. The latter arises from the random-walk nature of the ET requiring additional FRET steps to cover the same physical distance as compared to heteroFRET. A follow-up study applied machine learning to this complex data set to gain insights about how energy transfers across the D-relay and relay-A interfaces, and to understand the role that each site plays in these type of optoelectronic molecular networks.^333^ This report confirmed the complex interactions that dye networks undergo, noting that the presence or absence of downstream dyes could modify the probability of a given dye position acting as an energetic sink; the latter could arise due to subtle changes in the DNA nanostructures overall shape.^333^

Overall, this growing body of work confirms that harnessing homoFRET can significantly extend viable MPW lengths and improve achievable *E*_ee_ within these systems. More importantly, this work further suggested some seemingly contradictory or counterintuitive ideas: (1) that analytical random walk models as well as numerical FRET rate based models can properly describe the overall system if they are bound by some simple experimental results; and (2) an underlying assumption that the basic homoFRET models and the assumptions they are sometimes based on (*i.e.*, that homoFRET sections are composed of equivalent fluorescent moieties) may not hold within DNA nanostructures as nano-environments can create subtle-localized differences in the dye photophysical properties. As such, although models are good predictors of MPWs containing covalently bound homoFRET relays, detailed characterization may still be required for the best interpretation and understanding of the underlying phenomena.

#### 4.4.3. Adding intercalating dyes into the DNA MPW homoFRET mix

The examples above focused on covalently tethered dyes with their respective linkers conjugating them to the DNA oligos. Yet there are alternative approaches based on self-assembly of the dyes to the DNA to create MPWs. This is very enticing, as self-assembly strategies minimize the cost of including chemically modified nucleotides while exploiting van der Waals and hydrophobic interactions to direct the localization of the fluorophores. The most common of these DNA–dye interactions are based on the intercalation of dyes either between the nucleotides (base stacking) or by binding to the DNA double helix grooves.^334^ While maintaining similar requirements as those mentioned above for homoFRET relays, such as the need for a good homo-*R*_0_, such self-assembling dyes have two particularly important requirements. The first is the self-evident requirement that the binding constant be high and, in conjunction with this, that the photophysical properties (most often a decrease in the non-radiative decay rate) be strongly modified upon binding with DNA.^335^ By exploiting these two properties, such systems can avoid excess unbound dye in solution and, furthermore, any dye that is in solution will lack fluorescence, allowing for proper characterization and quantification of the ET that is now primarily localized to only the DNA structure/MPW and not the surrounding solution. In general, the increase in fluorescence is obtained by the increased rigidity of the dye molecules upon intercalation into the DNA, this nullifies the principal non-radiative decay mechanism improving ET properties.^335,336^

**Fig. 36:**
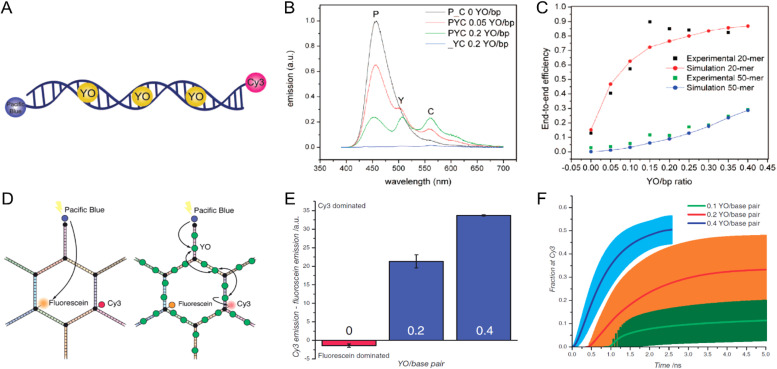
Exploiting intercalating dyes with homoFRET. (A) Schematic of the self-assembled YO relay in a dsDNA MPW. (B) Fluorescence spectra of MPW and controls at varying YO/bp ratios. (C) Simulated and experimental *E*_ee_ values as a function of YO/bp ratio for short and long MPWs. Reproduced with permission from ref. 330 Copyright 2008 American Chemical Society. (D) Schematic of the dual output DNA based photonic network. (E) The emission ratio of the acceptor dyes depends on the amount of YO/bp added to the system. (F) Modeling the fraction of excitation energy arriving at Cy3 as a function of time following the initial D excitation as a function of YO/bp. Reproduced with permission from ref. 337 Copyright, 2011, John Wiley & Sons.

The first example of efficient MPWs created by exploiting such dyes in a hybrid MPW was described by the Albinsson Group using pacific blue (PB) as the D and Cy3 as the final A as assembled on a 20- or 50-mer length dsDNA scaffold (∼6.6 and 16 nm, respectively), see Fig. 36A–C.^330^ Testing of this initial configuration confirmed no viable ET between the two dyes. Subsequent addition of the intercalating dye YO-PRO-1 (4-[(3-methyl-2(3*H*)-benzoxazolylidene)methyl]-1-[3-(trimethylammonio)propyl]) then facilitated ET through the MPW. This work was influential beyond just proof-of-concept, due in large part to its investigation into the benefits and limitations of intercalating dye-density in the MPW. Indeed, many of the authors of this review consider this to be one of the seminal papers in this field because it demonstrated what such structures could offer and the ease with which they could be rapidly reconfigured and tested in different configurations. In line with the results discussed above for covalently bound dyes, initial increases in dye density resulted in better transfer in homoFRET relays, yet a saturation and even inflection point could also be reached at higher density.^149^ In the specific case of intercalating dyes, the plateauing of the *E*_ee_ is caused by the natural limitation of binding site access and independently of the increase in dye concentration. Decreases in transfer efficiency here could in part be ascribed to the intercalating dye destabilizing the dsDNA, resulting in mal-formed MPWs. In this example, YO-PRO-1 was increased up to 0.4 dyes per bp within the 20-mer yielding an *E*_ee_ of up to 90% at 0.15 dyes per bp, and then decreasing down to 80% at 0.35 dyes per bp, see Fig. 36B. For the longer 50-mer scaffold, the peak *E*_ee_ of 20% was obtained at the highest dye ratio as the additional length increased the overall stability of the dsDNA. Finally to cap off this fascinating study, the authors were able to predict the ET within the MPWs quite accurately using a Markov chain FRET rate equation model (Fig. 36C).^337^ Subsequent work testing use of the intercalating dye BOBO-3 in a similar role increased the complexity of these systems by also integrating QDs as the central Ds.^126^ In a follow-on report from the Albinsson Group, YO-PRO-1 was again utilized but this time assumed a role in a dual output photonic network.^337^ In the latter example, PB was once more utilized as the initial D but the final A was alternated between either fluorescein or Cy3. Here, the final proportion of the energy directed to either A was modified by the inclusion of the intercalating YO-PRO-1, see Fig. 36D-F. Without the homoFRET relay, fluorescein was the dominant A emitter but upon inclusion of the YO-PRO-1 dye, the Cy3 now became the principal A. This added a dynamic component to the photonic network, though this particular design was not functionally reversible. Another example of dye intercalation altering the overall FRET dynamics was provided by Yang and co.^338^ They demonstrated that FRET within MPWs with a D–A system of 7-amino-4-methyl-3-coumarinylacetic acid (D) and Texas Red (A) could be regulated by the inclusion of different amounts of acridine orange as a homoFRET relay. The end result was that 36 unique emission spectra could be obtained. The authors then combined these as a conceptual cryptography system by creating 3 × 3 QR codes (multicolor 2 dimensional codes in this context).

A consistent limitation of including intercalating dyes into these MPWs is the lack of site-specificity or targeted placement of the intercalating dyes. Su and colleagues addressed this limitation by using the intercalating relay dye YO (oxazole yellow) as a homoFRET relay between PB and Cy3.^331^ In this configuration, the YO dye was conjugated to pyrrole-imidazole polyamides (PAs), which bind specifically within dsDNA's minor groove. Importantly, PAs have nanomolar affinity and can be designed with binding specificity for sequences ranging from 6 up to 10 bp in length.^339^ This approach allowed them to compare the site-specifically positioned PA-YOs to the same amount of randomly intercalated YO dye and they consistently found that there was increased A output for the PA-YO MPWs with an up to 3-fold increase in Cy3 emission (*E*_ee_ value of 14% for a 26 nm PA-YO MPW).^331^ This suggests that an optimal approach for using different intercalating dyes could integrate high affinity and site-specificity to such self-assembled DNA intercalated dye systems.

There are also derivative examples of integrating intercalator dyes within 1-D DNA templated structures in the literature, with the distinguishing factor in these examples being both their chosen dye and the integration of secondary components to interact with the dye. Ruiz-Carretero and coworkers utilized ssDNA, specifically a 40-mer oligothymine end-labeled with a Cy3 A, as a template for naphthalene guest molecules which had been labeled with diaminopurine to function as a D.^340,341^ The diaminopurine then acted as a homoFRET antenna with optimal transfer to the Cy3 A due to its DNA directed stacking and QY. The stacking of the guest molecules resulted in the straightening and rigidification of the dynamic ssDNA into what became essentially a 1-D wire. The Cotlet group brought in two different guest molecules including a cationic conjugated poly(phenylene vinylene) which wrapped around the DNA along with a malachite green chloride (MGC) for intercalating into the bps.^342^ They showed that both guest molecules had improved photophysical properties from interaction with the DNA bases and that both subsequently functioned as homoFRET antennas, the polymer as a D-antenna, and the MGC as the A-antenna. The end-goal application of this system was to determine DNA bp mismatches based upon the fact that the MGC interacted more weakly with DNA as a function of increasing bp mismatches; this modified the *E*_FRET_ from the polymer to the MGC and altered their ratiometric fluorescent signal. Here, the homoFRET relays were intended to provide exciton delocalization and high brightness to the sensor.

There are other functional alternatives to intercalating dyes such as the nucleotide tags pyrene or phenanthrene that, when integrated into DNA oligomers, will base stack with themselves as well as other nucleotides and increase their rigidity thus optimizing their fluorescent properties. As these dyes can serve as environmental reporters they are often exploited as sensors.^30^ The Häner group has explored their utility for diverse applications such as increasing catalysis^343^ along with using them to create MPWs. In a study reported by Bürki *et al.* homoFRET phenanthrene antennas were utilized as wires for a final pyrene A.^344^ By creating phenanthrene dimers separated by normal DNA bases, the authors created a homoFRET wire composed of coupled dimers that were able to maintain high transfer efficiency for up to four duplexes.

Although not the primary focus of this discussion, it is still important to note that research on DNA templated intercalating dyes is also pushing the limits beyond the weakly coupled homoFRET regime and beginning to exploit ET between strongly coupled dyes with both homoFRET and coherent transfer being jointly incorporated. This nascent field hopes to exploit the self-assembly of DNA nanostructures and dyes with the more complex photonics discussed in Section 5 of this review. The principal work at this time has focused on two cyanine derivatives. The benzothiazole cyanine dye K21 has been shown to form dye aggregates on DNA that couple strongly and has been integrated into complex and μm length scale DNA structures. The collaboration between the Woodbury and Yan groups has led to inventive application of K21 with initial work demonstrating a 32 nm MPW where the K21 functioned as the relay between AF350 and AF555 obtaining up to 60% *E*_ee_.^345^ This was then expanded to show K21 capable of integrating into extended linear origami constructs with reported transfers achieved across up to 500 nm along with the creation of 2-D LH networks that demonstrated a 2-fold enhancement over the discrete components.^346,347^ A key limitation of the K21 dye is that it has no site specificity for DNA sequences. The other cyanine dye derivative, pseudoisocyanine (PIC), has the benefit of preferentially binding and forming the required aggregates within designer AT tracks that extend across 8 bp of dsDNA.^348,349^ This characteristic allows the PIC to self-assemble in unique positions within more complex structures that are no longer limited to linear systems, and for the aggregates to function as unique molecules.^350,351^ Continuing in this vein, the work of Chiriboga and colleagues demonstrated that PIC aggregates could be split into non-contiguous tracks and that they could function as ET As to an AF405 and Ds to an AF647 in this confirmation; frustratingly the authors demonstrated that as homoFRET relays the individual PIC aggregates were not that efficient.^352^ Follow-up studies demonstrated that the spectral properties of the PIC aggregate itself can be tailored by modifying the properties, *e.g.* sequence and rigidity, of the chosen DNA template, providing an additional variable for developing DNA based photonic structures.^353^ Though quite promising, to ultimately meet expectations desired from this field, dyes will have to be found that not only have the desired optical properties upon aggregation, *i.e.* strongly coupled and high fluorescent QY, but that have specificity and perhaps most importantly higher binding efficiency than the currently available dyes. All of the above examples required large excesses of dye to be added to the DNA to achieve steady-state binding in the experiments, and this excess confounded achieving a clean analysis. For those with a greater interest in the area of dye aggregates there are some excellent reviews available.^354,355^

#### 4.4.4. HomoFRET for improving light harvesting

**Fig. 37:**
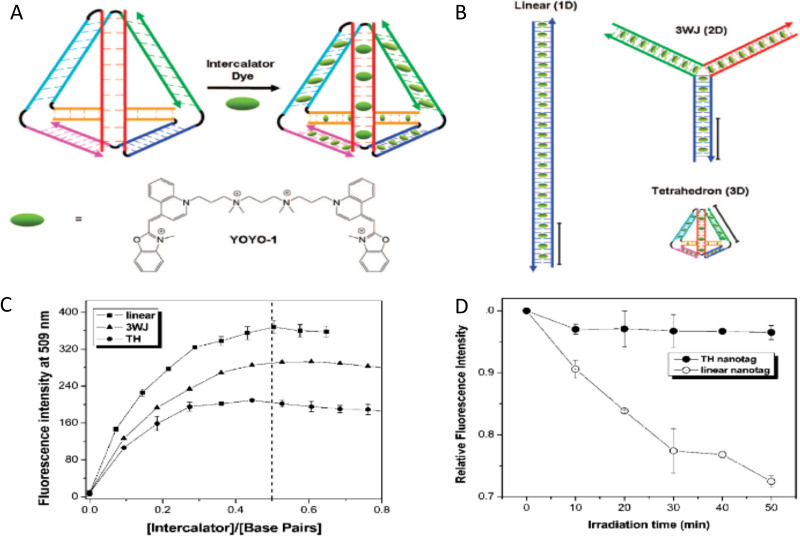
DNA nanotags. (A) Schematic of DNA nanotags and their formation based on intercalation of YOYO-1 dye into dsDNA assemblies. (B) Fluorescent DNA nanotags: linear (1-D), 3-way junction (2-D), and tetrahedron (3-D) Scale bar = 8.5 nm. (C) Fluorescence intensity of YOYO-1 as a function of the nanotag shape and the dye/bp ratio. (D) Photostability of the DNA nanotags. Interesting to note that not only is the tetrahedron structure more stable against enzyme and medium degradation but the YOYO-1 is also more photostable in the tetrahedron. Reproduced with permission from ref. 357 Copyright 2009 American Chemical Society.

As described above, homoFRET relays can function as antennas in 1-D MPWs, but neither FRET nor the random-walk process are limited to just one dimension. The use of DNA dendrimers as light harvesters to transmit excitons to a final A fluorophore often functionally exploits homoFRET in ingenious forms geared towards optimizing the overall ET efficiency. In general, exploiting homoFRET here aims to maximize the initial D absorption cross-section while also providing redundant/alternative FRET pathways to optimize the overall *E*_ee_. Due to the inherent high density of dyes present in dendrimeric DNA structures and other MPWs,^103,298^ it is expected that homoFRET transfer is almost always present, yet in many cases this aspect is often overlooked. One of the first uses of homoFRET LH was not in a traditional DNA dendrimer but for comparison between linear dsDNA, a 3-way junction, and DNA tetrahedron assemblies; the latter two designs can essentially be considered as proto-dendrimers, see Fig. 37.^356,357^ The intercalating YOYO-1 dye (a homodimer of oxazole yellow) was utilized for this purpose as a homoFRET antenna to funnel energy to a terminal Cy3 A.^356^ Here, the homoFRET antenna was meant to maximize the absorption cross-section and increase the overall brightness such that the authors even suggested these ‘nanotags’ could be useful in cellular fluorescence microscopy. Consistent with that observed previously when using YO-PRO-1,^330^ there was a peak ‘labeling density’ of YOYO-1 relative to the number of DNA bps present and additional dye was found to be counterproductive. Interestingly, the authors found that the linear dsDNA actually had the brightest fluorescence, but the tetrahedron yielded the more optimal nanotag. An important point when considering downstream applications for DNA-photonic structures is their stability in the final medium. If the intent is to ultimately use them in cells, resistance to nucleases will be crucial to maintain the dsDNA composition of the DNA. In this case, the authors found that the tetrahedron was far more stable than the linear dsDNA and the 3-way junction. This stability is presumably due to the inherent torsion and rigidity that this structure places on its component strands, which can make it less recognizable by nucleases.^64,358–360^

**Fig. 38:**
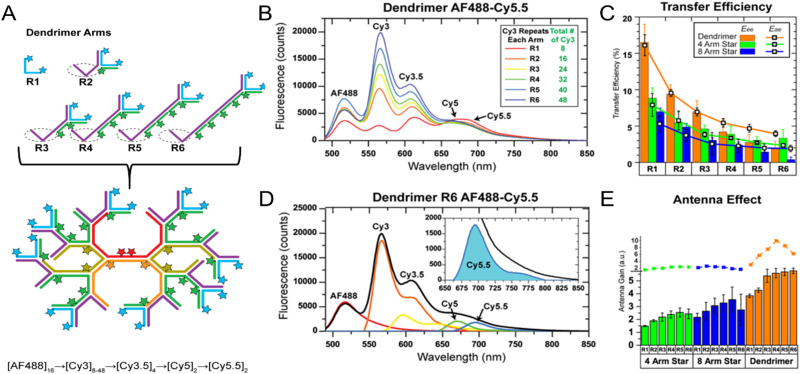
Different homoFRET repeats as antennae in a DNA dendrimer. (A) Schematic of the DNA dendrimer with extendable homoFRET arms to increase LH. R1-R6 correlates with the number of Cy3 homoFRET dyes. (B) Fluorescence spectra of R1–R6. (C) *E*_ee_ and *E*_ea_ of varying LH structures as a function of the number of Cy3 homoFRET steps (*i.e.* R1–R6). As expected, the larger/longer structures have lower values. (D) Fluorescence spectral deconstruction of the R6 dendrimer showing the emission from the final Cy5.5 acceptor. (E) Predicted (line plot) and experimental (bar plot) AG of varying LH structures as a function of the number of Cy3 homoFRET steps (*i.e.*, R1–R6). Here it is demonstrated how initial homoFRET additions increase the AG but subsequent additions are either neutral or impair LH. Reproduced with permission from ref. 151 Copyright, 2018, John Wiley & Sons.

Researchers at the U.S. Naval Research Laboratory came to a related conclusion that structural integrity is a key component for optimizing LH in these type of structures. As exemplified by several of the aforementioned cases, optimal LH and ET in DNA dendrimers counts on more than just increasing dye density, and is a result of a balance between redundant pathways, close dye proximity, and relatively high formation efficiency.^252^ Building from this realization, they studied the use of Cy3 homoFRET antennas attached to the exterior sections of both DNA dendrimers and multi-arm stars, see Fig. 38.^151^ In these systems, the FRET relay was composed of AF488 → Cy3_(*n*)_ → Cy3.5 → Cy5 → Cy5.5, where the Cy3 homoFRET antenna/relay could be from *n* = 1–6 repeats in length. Importantly, they designed the DNA structures to have minimal difference in the formation efficiency between the different lengths (*ca.* >70% for all structures) permitting a more direct comparison of the LH efficiency. The results of AE and AG comparisons between the different length homoFRET DNA nanostructures align well with the general benefit/limitation dichotomy of homoFRET antennae as the number of dyes increase. Focusing on the DNA dendrimer results, as the number of Cy3 homoFRET dyes increased from one to three, there was a marked increase in the AE from ∼4.0 to 5.5, yet subsequent additions showed only small increases up to 5.7 AE with six Cy3 dyes (Fig. 38E).^151^ For context, the *E*_ee_ decreased from 17% to 7% and then to 2% as the Cy3 dye number increased from one to three to six. In-depth characterization again revealed the complex interplay between the positive increases in absorbance cross-section at higher homoFRET dye number with the contrasting decrease in transfer efficiency due to the random-walk nature of homoFRET.

#### 4.4.5. Understanding the nuances of homoFRET in DNA

Determining the mechanisms that underpin homoFRET is not always simple and though some general assumptions can be applied, deviations are often observed since the DNA template itself always presents diverse nano-environments depending on its sequence, structure, and nearby modifications.^113,245,361^ Some interesting work has been done on combining DNA templates with different kinds of chromophores and conjugation strategies towards the goal of a better understanding of the subtle mechanics and nuances that are found with homoFRET. For example, the Hanley group exploited peptide nucleic acids (PNA), a synthetic DNA alternative where the sugar-phosphate backbones are replaced by *N*-(2-aminoethyl)glycine-based polyamide moieties,^362^ to study DNA-templated homoFRET.^363,364^ The PNA maintains bp matching but the chemistry to conjugate the PNA to a peptide is much simpler than that for conjugating DNA to a peptide, thus expanding the potential DNA conjugation toolbox.^21^ Hanley conjugated monomeric teal fluorescent protein (TFP) to a site-specific PNA tag allowing multiple TFP to be bound to a ssDNA template as confirmed by size exclusion chromatography and electrophoresis.^363^ Anisotropy was measured as the DNA-templated TFP increased from a monomer up to a tetramer, and it was found that the results followed the expected homoFRET predictions extremely well. This concurrence occurred since the PNA tag is very site-selective and the interior of the fluorescent proteins where the chromophore is located are shielded by its surrounding beta-barrel structure minimizing any variation caused by the DNA nano-environment. A second example used an A488 dye-labeled SNAP-tag protein conjugated to the PNA tags,^364^ the A488 dyes were not as secluded as the TFP chromophores and the decrease in anisotropy was slightly lower than predicted, perhaps due to the dye's greater exposure to the DNA nano environment. In evaluating these results, the authors highlighted the importance of considering the change in baseline anisotropy as the size of the construct is modified. As noted above, this can result in overestimating homoFRET values if not properly taken into account.^327,363,364^

**Fig. 39:**
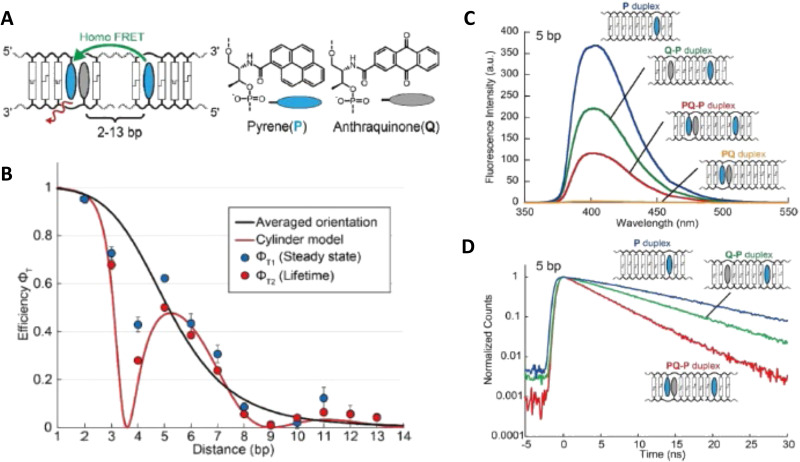
HeteroFRET for homoFRET quantitation. (A) Schematic of dsDNA with pyrene homoFRET dyes separated by 2-13 bp. A single anthraquinone quencher is also integrated and the change in heteroFRET is correlated to the homoFRET of the pyrene dyes. (B) The homoFRET of the pyrene is best fit by the cylindrical dsDNA model, demonstrating that orientation is crucial for homoFRET transfer. (C) Fluorescence spectra of dsDNA containing variations of the pyrene/anthraquinone positions. (D) Fluorescence lifetime of dsDNA containing variations of the pyrene/anthraquinone positions. Reproduced from ref. 365 with permission from Springer Nature, copyright 2021.

Using a different assembly approach, the Asanuma Group focused on closely examining homoFRET with base-stacking dyes such as perylene and pyrene. Exploiting DNA's modularity they conceived an ingenious approach of using heteroFRET as a means of looking at homoFRET efficiency.^365,366^ The homoFRET dye pair, either two perylene or two pyrene dyes, are studied with steady-state and fluorescence lifetime spectroscopy at varying bp separations, subsequently the same structures are interrogated with the introduction of an anthraquinone A placed in an asymmetrical manner that is closer to one dye than the other, see Fig. 39. Changes in heteroFRET could then be correlated with the homoFRET efficiency - the less efficient the homoFRET the less the secondary dye is affected by the anthraquinone A and the greater its fluorescence intensity and lifetime. They further demonstrated that Förster theory holds well for the observed homo- and heteroFRET results by taking into account the rotating cylinder model of dye placement within a dsDNA helix. Critically, they also provided us with a new anisotropy-free alternative methodology for probing homoFRET in a DNA-templated assembly.^365,366^

Overall, it is readily apparent that the use of homoFRET within the two most popular applications currently being explored with DNA templated nanostructures, namely MPWs and LH, comes with both its pros and cons. The benefits include accessing dyes with better QYs and fluorescence lifetimes, maintaining the energetic level of the exciton, maximizing absorption cross-section, and supplying redundant/alternative FRET pathways. How important each of these traits is and how much they will contribute will obviously depend on the underlying MPW design and its specific application. The cons arise from the random-walk nature of exciton transfer and the limited efficiency of structure formation as the designs become more complex, which of course applies equally to heteroFRET as well. For LH in particular, including homoFRET sections or modules can provide considerable benefits when initially introduced at low dye copy number by increasing the absorption cross-section and providing alternative and functionally redundant FRET pathways. However, trying to push the benefits further is often counterproductive due to the decrease in transfer efficiency caused by the random walk nature of the exciton transfer in these systems and the diminished proportion of properly formed DNA nanostructures. It is hoped that by describing these examples, they can cumulatively serve to help demystify the role of homoFRET within such optically active DNA nanostructures. As also described, there are multiple available approaches to quantify homoFRET efficiency and the cognate theoretical framework that exists and is readily available for a user to predict how homoFRET will affect their system and how best to exploit it.

## 5. Exciton delocalization in DNA-templated dye aggregates

The following sections review aggregates formed from dyes covalently linked to ssDNA and organized by DNA scaffolds. The first section examines dye aggregates comprised of specific types of dyes whereby the aggregates are studied predominantly using steady state optical spectroscopy including UV-vis absorbance and circular dichroism (CD). The second section examines various ways that exciton delocalization has been incorporated into DNA for various applications such as hybridization assays and also includes fascinating work from the Asanuma group that tested some of the basic theories in different experimental formats. The third section discusses various mechanisms that have been studied *via* time dependent and steady state optical spectroscopy. In the final section, the dynamics of molecular excitons in aggregates are discussed relative to some of the challenges faced with realizing fluorescent aggregates including excited state quenching and static and dynamic disorder and structural heterogeneity. We point out that this survey is by no means an exhaustive review of exciton delocalization in DNA templated dye aggregates. A considerable amount of work has been done by others, which is highlighted in reviews and reports by Asanuma and coworkers.^367–369^

### 5.1. Experimental and theoretical studies of delocalized excitons in DNA dye aggregates

#### 5.1.1. DNA-templated cyanine dye aggregates

**Fig. 40:**
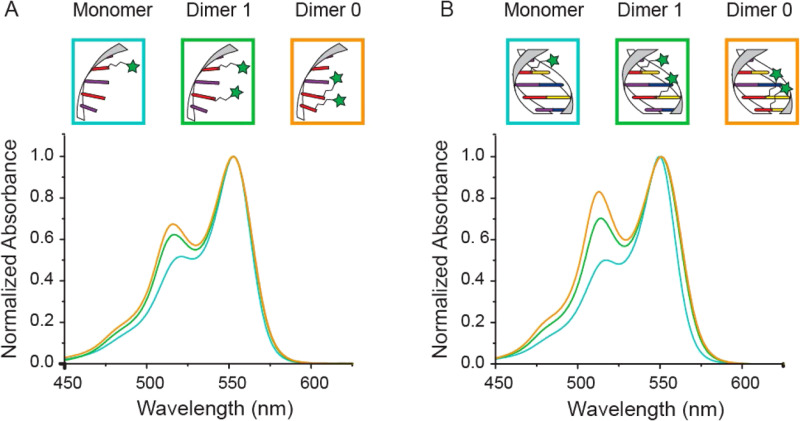
Proximity-induced h-aggregation of cyanine dyes on DNA-duplexes. Absorption spectra of the dye-DNA constructs. (A) Normalized absorption of the ssDNA and (B) dsDNA constructs. The light blue curves correspond to the Cy3 monomer, while the green and orange curves correspond to the Cy3 dimer with 1 base and 0 base distance, respectively. Reproduced with permission from ref. 370 Copyright 2016 American Chemical Society.

Because of their large molar absorptivity and compatibility with DNA, the cyanine dyes Cy3 (*ε*_max_ ∼ 150 000 M^−1^ cm^−1^) and Cy5 (*ε*_max_ ∼ 250 000 M^−1^ cm^−1^) have been used in multiple conceptual experiments to explore exciton delocalization in small dye aggregates templated on DNA scaffolds. One of the first DNA-templated cyanine dye aggregates to be investigated for exciton delocalization were dimeric sequences comprised of Cy3 dyes.^370^ Nicoli *et al.* used DNA duplexes to form several types of Cy3 dimers including two Cy3 dyes on adjacent bases of a ssDNA strand attached using a single NHS ester linker and two Cy3 dyes each on a complementary DNA strand attached with possibly a double phosphodiester linker such that the dyes were across from one another. The type of linker was not specified even though it could be potentially pivotal in these contexts since whether the linker is a double tether or single tether as well as the length of linker determines the extent that the dye is allowed to move about. As compared to a monomer that does not exhibit exciton delocalization, all the dimers showed evidence of exciton delocalization in both absorbance and CD data. All dimers also manifest H-aggregate like stacking or H-like behavior (*i.e.* dye molecules stacked predominantly with a face-to-face orientation).^220^ The absorbance spectra of the dimers in general showed a relative increase in the vibronic peak (0–1 band) intensity, however there was little evidence of Davydov splitting in the lowest energy absorption band (Fig. 40). For dimers assembled with a single linker dye attachment, a CD couplet was not present and only a negative signal was observed. These observations suggested that the exciton coupling strength was relatively small compared to the linewidth of the 0–0 monomer absorption band, and/or that the sample was a heterogeneous mixture of coupled dimers and monomer species.

The strength of exciton coupling, *J*_*m*,*n*_, was not stated, rather, to gain additional insight, Nicoli *et al.* performed MD simulations and time-dependent density functional theory (DFT) and the results showed that the Cy3 dimer solution contained a combination of dimer (1/3) and monomer (2/3) populations; hence, the authors concluded that the sample was heterogeneous. The time dependent-DFT (TD-DFT) calculations of absorbance were quite similar to the absorbance data suggesting H-like stacking of the dimers. MD simulations supported the notion that chiral stacking of the dyes occured in the dimer as opposed to DNA intercalation of the dyes and that the dyes stayed near the negatively charged DNA backbone. Furthermore, an average orientation of the dyes in the dimers was extracted *via* MD simulations showing a small amount of twisting occurring. Finally, they also examined the distance dependence between Cy3 dyes in the dimer by varying the number of bases between the two dyes by either 1 or 6 bp separation. While somewhat diminished, signatures of exciton delocalization continued to be present in the dimers with the 1 base pair separation but signatures of exciton delocalization were not visible with the dyes in the dimer separated by 6 bases. For Cy3 dimers, much stronger signatures of exciton coupling have been observed when the dyes are doubly-attached to DNA (*via* phosphodiester attachment chemistry) and situated at the same position along the DNA duplex, at so-called 0 bp separation directly opposite each other in the ds DNA.^110,370–372^ This dye attachment strategy produced clear signatures of Davydov splitting in the absorption spectrum and distinct couplets in the CD spectrum. The optical spectra exhibited both J-like and H-like signatures depending on the local DNA sequence surrounding the dyes and the value of *J*_*m*,*n*_ was determined to be ∼ 250 cm^−1^.^370^ Nevertheless, the Nicoli study epitomizes the utility of combining steady-state optical absorbance and CD data with TD-DFT and MD simulation. Using Frenkel molecular exciton theory to extract key parameters from the dimer data such as dye orientation and *J*_*m*,*n*_ could have further provided insight to the strength of delocalization and the orientation of the dimer as compared to their MD results. Interestingly, their MD simulations of the monomer showed that Cy3 preferred to lie along the major groove suggesting a low energy barrier for monomer orientation along the major groove. In contrast to this, the MD results of Stennett *et al.* suggested a low barrier for partial intercalation of the Cy3 monomer.^373^ Similarly, MD results by Cervantes-Salguero *et al.* showed partial intercalation of the Cy5 monomer. For the latter, the authors indicate that the base sequences most likely play a role in dye stacking behavior.^106^

**Fig. 41:**
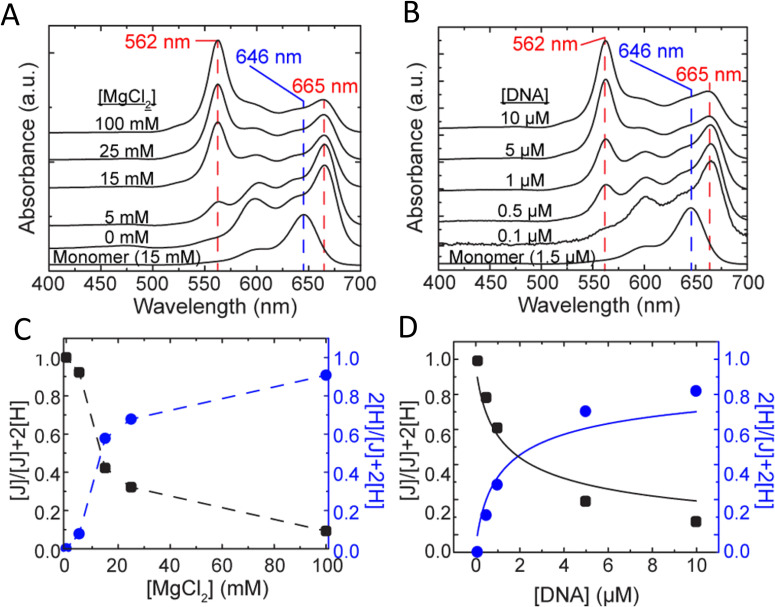
Varying H- and J-aggregate structure with salt in DNA Holliday junctions (see Fig. 56 for HJ structure). Observed UV-vis absorbance spectra with varied (A) salt (DNA concentration held constant at 1.5 μM) and (B) DNA (added MgCl_2_ concentration held constant at 15 mM) concentrations. Changes in J-dimer (black squares and ordinate axes) and H-tetramer (blue circles and ordinate axes) fractions (based on their respective absorbance peaks) as a function of (C) salt (*i.e.*, MgCl_2_) and (D) DNA concentrations. The dashed lines connecting the points in panel C are for visual aid only, while the curves in panel D were obtained by fitting the data. Each spectrum was acquired at room temperature. Reproduced with permission from ref. 374 Copyright 2017 American Chemical Society.

In 2017, Cannon *et al.* published one of the more definitive works demonstrating exciton delocalization in Cy5 dye aggregates assembled by both a DNA duplex and a DNA mobile HJ.^374^ The commercially obtained Cy5 dyes were covalently attached to DNA using the dual phosphodiester linker attachment. They used steady-state optical techniques including absorbance, CD, and fluorescence to establish signatures of exciton delocalization and, in particular, look for spectral shifts as compared to the Cy5 monomer. Most of their DNA templated aggregates were assembled in solutions using MgCl_2_ to stabilize the resulting structures that formed. This was undertaken because the authors were aware that Markova *et al.* had demonstrated exciton delocalization in Cy5 dimers using duplex DNA templates in NaCl buffer which differed in ionic strength to that of the MgCl_2_ solution that Cannon used.^375^ This difference led Cannon to examine the impact of a variety of salt concentrations on exciton delocalization in Cy5 dimers. Cannon *et al.*, observed was that salt concentration affected the binding of the DNA and dye–dye interaction in the aggregates such that duplexes were prevalent at low MgCl_2_ concentrations while mobile HJs were formed at higher MgCl_2_ concentrations, see Fig. 41. The dye stacking in the DNA aggregate changed from J-like dimers templated by DNA duplexes to H-like tetramers templated by mobile HJs as the MgCl_2_ concentration increased. In contrast to H-like stacking, J-aggregate stacking or J-like behavior is where dye molecules stacked predominantly with a head-to-tail arrangement.^208,209,211,219,220^ Interestingly, fluorescence was strongly quenched, relative to the monomer emission, in both the J-like (unexpected) and H-like aggregates (expected). Cannon also investigated the impact of DNA-template concentration on exciton delocalization.^374^ As the DNA-template concentration increased, the DNA-templates changed from duplexes to mobile HJs. The duplexes induced dimers, which stacked in a J-like configuration, while the mobile HJs formed tetramers, which stacked in an H-like manner. As before, the J-like and H-like aggregate fluorescence emission was strongly quenched, relative to the monomer emission. Based on these changes in aggregate stacking and DNA templates, a thermodynamic and kinetics model was used to describe the results. Although both absorbance and CD data can provide a qualitative description of stacking behavior (*e.g.*, J-like *versus* H-like), a quantitative description of relative position and orientation of the dyes within the aggregate cannot be determined by visual inspection of the data alone. Yurke and coworkers developed an innovative simulation tool based on the vibronic exciton theory of Kühn, Renger, and May^376^ and which uses the Frenkel-Holstein Hamiltonian (eqn (19)) to extract the relative positions and orientations of the dyes within the aggregate by simultaneously fitting the absorbance and CD data. The Frenkel–Holstein Hamiltonian extends Frenkel molecular exciton theory (see theory section earlier titled “Frenkel molecular exciton theory and key parameters”) by including vibronic contributions to the Hamiltonian. Their in-house software program called the KRM Model Simulation Tool is referred to as the KRM tool henceforth for brevity.^374^ While most published research on DNA-templated dye aggregates infer qualitative aggregate stacking behavior by only visually examining absorbance, CD, and/or fluorescence data, Cannon *et al.*'s work was one of the first to combine both optical data and a Frenkel–Holstein Hamiltonian approach to quantitatively extract relative dye position and orientation within a DNA-templated dye aggregate.^374^ This development provided a powerful foundation to understand dye aggregate configuration in much of this group's subsequent research. As an interesting aside, this Cannon *et al.*'s article would have been published over a year earlier had the reviewers not insisted that quantitative relative dye position and orientation be provided. In contrast, many publications prior to this point (*ca.* 2016) did not provide such information. While it can be argued from one respect that the reviewers were being biased, over time, Cannon *et al.* and others have benefited from what at first might have seemed like an unreasonable request. Finally, this work demonstrated that the transition of exciton delocalization from J-like to H-like dye aggregate stacking could be controlled by varying salt and/or DNA concentrations.

**Fig. 42:**
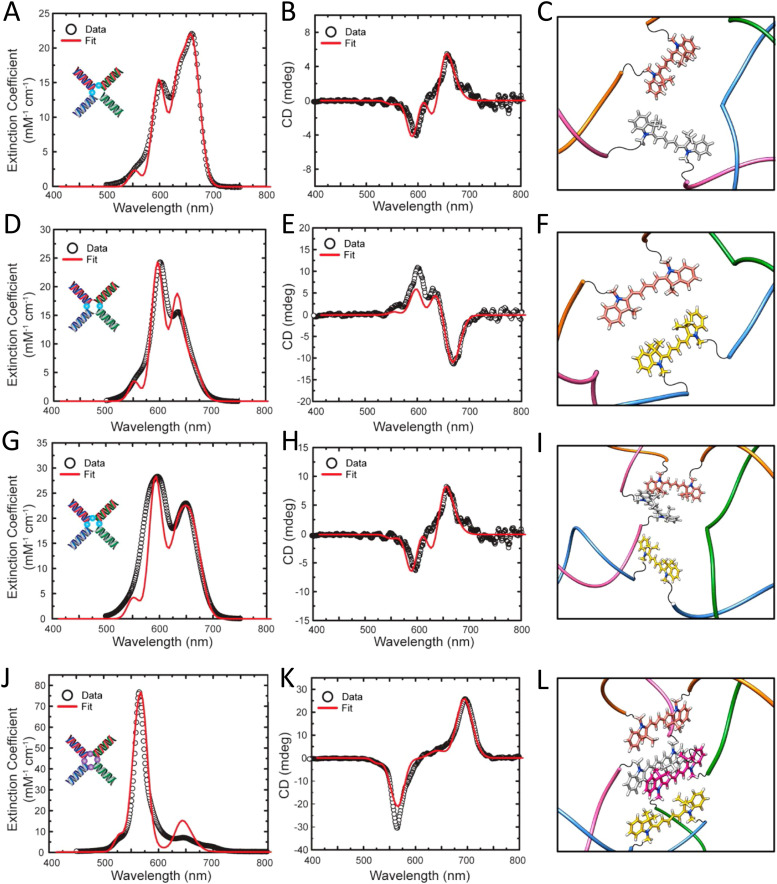
KRM (KRM) Frenkel-Holstein Hamiltonian Model Simulation Tool fits and data. Absorbance (A), (D), (G), and (J) and CD (B, E, H, and K) spectral fits with corresponding structures (C), (F), (I) and (L) of the adjacent dimer, transverse dimer, trimer, and tetramer aggregates, respectively. Experimental data sets are shown as black open circles, while KRM theoretical fits are given as red curves. Fluorescence measurements were obtained by exciting the samples at their respective absorption maxima. Reproduced with permission from ref. 377 Copyright 2018 American Chemical Society.

**Fig. 43:**
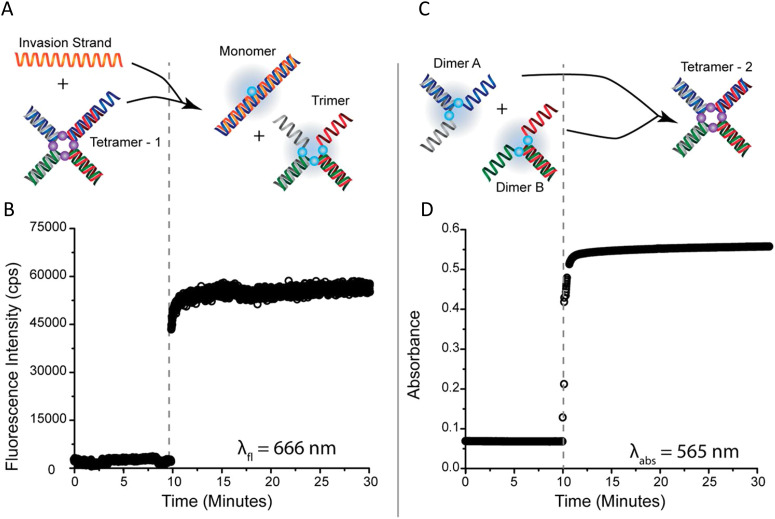
Optical Reporter Functionality (A) Schematic illustration and (B) experimental demonstration of the 4AJ tetramer undergoing strand-invasion using fluorescence detection (*λ*_fl_ = 666 nm). (C) Schematic illustration and (D) experimental demonstration of the 4AJ tetramer formation *via* DNA hybridization using UV-vis−NIR absorbance, or colorimetric, detection (*λ*_abs_ = 666 nm). Reproduced with permission from ref. 377 Copyright 2018 American Chemical Society.

Based on this 2017 publication,^374^ the same authors hypothesized that immobile DNA HJs would template more complex aggregates beyond dimers that included both trimer and tetramer aggregates that, because of the greater number of dyes in the aggregate, would exhibit more extensive excitonic delocalization than dimers.^377^ At that time, neither immobile DNA HJs nor other larger DNA templates had yet been used to organize dye aggregates. Using commercially obtained Cy5 dyes, dye aggregates were organized within the central portion of an immobile HJ *via* covalent dual phosphodiester linker attachment; this allowed for two types of dimers (adjacent dimer and transverse dimer), a trimer, and a tetramer to be experimentally examined. The steady-state optical data, absorbance and CD, of all the dye aggregates showed signatures of exciton delocalization. Specifically, the absorbance data showed blue-shifted spectra as compared to the monomer dyes, which is indicative of H-like aggregates. Simultaneous fitting of the absorbance and CD data using the KRM tool developed for Cannon *et al.*'s 2017 publication^374^ confirmed an H-like stacking orientation occurred for both the transverse dimer, trimer and tetramer while a J-like stacking orientation resulted for the adjacent dimer, see Fig. 42. The tetramer produced the largest Davydov splitting (397.5 meV) observed to date for a DNA-templated dye aggregate, which was significant enough to induce a color change in the solution due to the large shift in absorbance and confirmed the hypothesis that aggregates with a greater number of dyes can induce more extensive exciton delocalization. Nearly 80% fluorescence emission suppression, or quenching, was also observed for the tetramer. Leveraging the large emission suppression and Davydov splitting, two excitonic switches (dynamic) based on aggregates (*i.e.*, exciton delocalization) were fabricated that functioned either *via* absorbance or fluorescence by exploiting DNA strand invasion.^288,293^ The absorbance-based excitonic switch yielded a 10-fold increase in absorbance while the fluorescence-based excitonic switch yielded a 25-fold increase in fluorescence (Fig. 43).^377^ While these dual absorbance and fluorescence-based exciton delocalization switches are the first of their kind and thus quite unique, their minutes-long switching times limit their use to medical and imaging applications and are far from meeting the switching times necessary for microelectronic switches for computing and memory applications. Using photochromic molecules incorporated into the DNA that proximate to aggregates has the potential to shorten switching times to the picosecond range necessary to compete with microelectronic switches.^197^

**Fig. 44:**
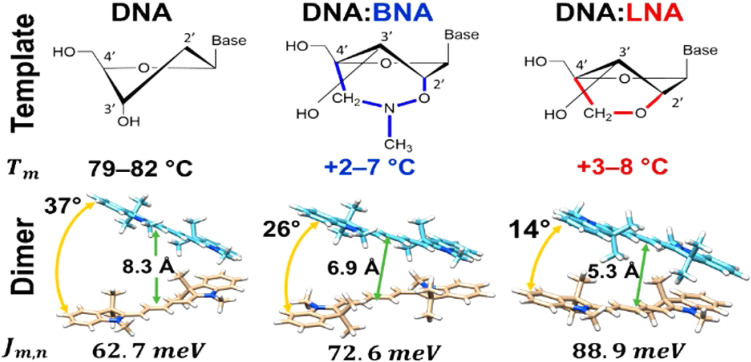
Bridged nucleotide-substituted, DNA duplex-templated cyanine aggregate structures. Examples of dimer configurations determined from KRM Model Simulation Tool outputs along with predicted *J*_*m*,*n*_ values. Reproduced with permission from ref. 224 Copyright 2018 American Chemical Society.

The tendency of DNA duplexes to transiently but naturally destabilize and unwind from the ends, often referred to as DNA “breathing,”^114^ presents a unique challenge for exciton delocalization. The degree of delocalization, as measured by *J*_*m*,*n*_, is a function of both the distance between dyes in an aggregate (*i.e.*, inter-dye distance) and the dye-to-dye orientations. Because DNA breathing causes dye movement and associated changes in inter-dye distances and orientations, it influences both *J*_*m*,*n*_ and delocalization. Roy *et al.* hypothesized that incorporating structural modifications to the nucleic acid bases nearest and next-nearest to the dyes, with replacement *via* bridged nucleotides, might stabilize the DNA and inter-dye orientations and distances and thus lead to larger realized *J*_*m*,*n*_ values.^224^ Their study examined this effect by comparing *J*_*m*,*n*_ values of Cy5 dye aggregates for which (at least) the dyes nearest and next-nearest neighboring bases were hybridized with one of two bridged nucleotides, locked nucleic acid (LNA) bases, bridged nucleic acids (BNA), or with natural DNA bases in the duplex DNA scaffold, see Fig. 44. The aggregates used in the study included a dimer, trimer, and tetramer, each comprised of commercially available Cy5 dyes that were covalently attached to ssDNA *via* a dual phosphodiester linker with the dye-labeled strand subsequently hybridized to its complement. In general, exciton delocalization was observed in all aggregates and the presence of BNA in the DNA increased both the stability of the scaffold and *J*_*m*,*n*_. To demonstrate these results, Roy *et al.* performed absorbance, CD, gel electrophoresis, and melting curve analysis. The KRM tool that was used in previous publications was similarly applied here to extract *J*_*m*,*n*_, as well as inter-dye orientation and distance for each aggregate.^222,374,376,377^ Uniquely, Roy *et al.* also extracted the absorbance transitions separated into proportions of purely electronic transitions and transitions involving at least one quanta of vibrations. The amount of electronic *versus* vibronic contributions revealed that the higher energy peak was significantly vibronic, whereas the lower energy peak was predominately electronic in nature. Extracted orientation results confirmed that H-like stacking in all aggregates was prevalent. The extracted parameter *J*_*m*,*n*_ showed that replacing natural nucleotides with bridged nucleotides yielded a discernible increase in *J*_*m*,*n*_ (≥ 10 meV) with *J*_*m*,*n*_ across all aggregates ranging in value from ∼63 meV to ∼89 meV. The DNA melting curve (*i.e.*, DNA denaturing) data showed, in comparison with natural nucleotides in analogous scaffolds, that the presence of bridged nucleotides (LNA or BNA) did increase the stability of the DNA; however, this stability was partly offset by the inclusion of dyes. The authors pointed out that multiple configurations of aggregate orientations might occur within the samples (aggregate heterogeneity). The heterogeneity may be due in part to the manner in which the dyes are inserted in the DNA backbone, but further investigations will be required to better understand and limit this heterogeneity. It is also worth noting that the authors provide some valuable insight specific to the impact of vibrational effects on the absorbance and CD data using the KRM tool. They demonstrate that the vibrational impact *via* use of the Huang–Rhys factor, *S*_*m,α*_, on the absorbance and CD is required to simultaneously fit the absorbance and CD data appropriately.^229^

#### 5.1.2. DNA-templated squaraine dye aggregates

Squaraine (SQ) dyes offer increased rigidity and photostability as compared to cyanine dyes. In 2012, Markova *et al.* first demonstrated exciton delocalization in a SQ dimer templated by dsDNA.^375^ Double phosphodiester linkers were used to attach the custom-synthesized SQ dye to the ssDNA template strands. Absorbance data showed that the dimer spectrum was blue-shifted with respect to the monomer spectrum indicating H-like stacking of the SQ dyes in the dimer. Intense couplets were also observed in the CD data of the dimer and were indicative of strong exciton delocalization. Fluorescence of the monomer was also quite intense while the fluorescence of the dimer was significantly subdued, or quenched, substantiating the H-like stacking of the dimer that the absorbance data indicated. DNA melting analysis was performed, showing little hysteresis for which the authors suggested that the SQ–SQ dye interaction was strong enough to have a stabilizing effect on the underlying dsDNA scaffold. Frenkel exciton modeling to extract *J*_*m*,*n*_ and dye orientation of the dimer was not performed so the strength of exciton delocalization and the specifics of H-like stacking are unknown.

**Fig. 45:**
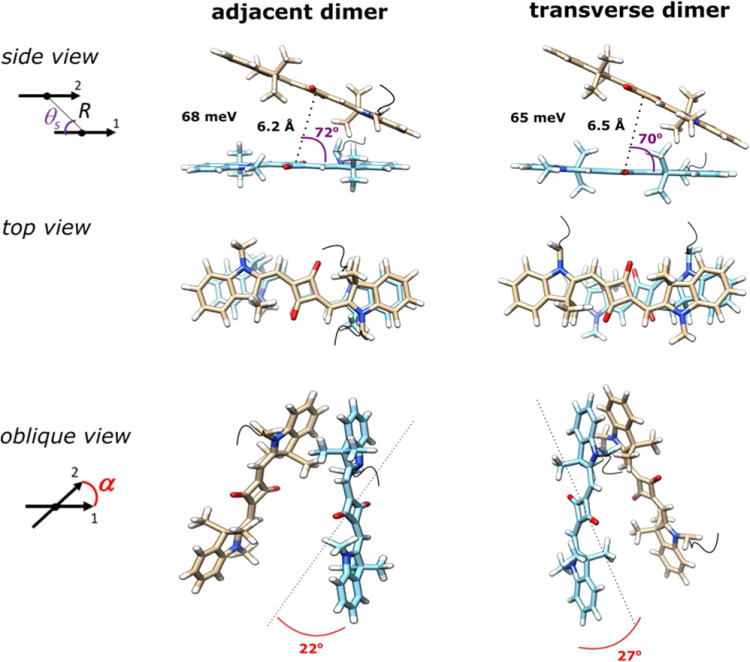
Indolenine SQ aggregates templated by DNA HJ scaffolds. Molecular models of the Square 660 core region adjacent dimer SQ-BC and transverse dimer SQ-AC (see Fig. 46B). The side view shows a J1,2 parameter, in meV, a center-to-center distance *r*, in Å, and a slip angle θs, in degree. The oblique view shows oblique angle α, in degree, as an angle between vectors 1 and 2 if their centers are superimposed. Note that the fitting procedure determines the position and orientation of the long axes of the Square 660 dyes but not the rotation of the dye core around its long axis. As such, the dye core rotations were arbitrarily chosen. Reproduced with permission from ref. 222 Copyright 2020 American Chemical Society.

Mass *et al.* followed Cannon *et al.*'s 2018 work^377^ by examining commercially obtained indolenine SQ dye aggregates templated *via* four-arm DNA HJs where the SQ dyes were covalently attached *via* single flexible linkers to ssDNA.^222^ Aggregates examined included an adjacent dimer, a transverse dimer and a tetramer with all showing evidence of exciton delocalization *via* steady-state absorbance, CD, and fluorescence spectroscopy. CD spectra also exhibited strong couplet behavior and absorbance spectra were blue-shifted or showed Davydov splitting with greater blue-shifted peak intensities observed indicative of H-like stacking of the dyes within the aggregates. Strong suppression of fluorescence emission was again observed as a signature of H-like stacking. The latter orientation of the dyes in the aggregate was confirmed by simultaneous fitting of the absorbance and CD data using the KRM theory.^376^ The KRM simulation tool was also used to extract the Coulombic coupling strength between dyes in the aggregate – this coupling is what induces the exciton delocalization. At the time of Mass *et al.*'s publication, the KRM tool was augmented to extract the transition dipole from the monomer absorbance spectrum and thereby eliminate a fitting parameter. This approach more readily enabled the extraction of the Coulombic coupling strengths between dyes in the aggregate and did so in a more constrained manner. This augmented KRM tool allowed Mass *et al.* to expand the discussion of exciton delocalization to include the Coulombic coupling strength that results due to a single exciton being shared between dyes in the aggregate – in essence, it now provides a quantitative metric for the coupling that manifests exciton delocalization. Based on their exhaustive review of the literature, the authors argue that the Coulombic coupling strength between two dyes, *m* and *n*, in an aggregate is most generally referred to as the excitonic hopping parameter, *J*_*m*,*n*_. *J*_*m*,*n*_ is typically expressed in units of energy and specifically electron volts. A desirable value for *J*_*m*,*n*_, or the Coulombic coupling strength, is to be greater than the thermal energy, *k*_B_*T*, at room temperature (0.026 eV) since thermal energy-induced vibrational modes and/or the surrounding thermal bath would significantly dissipate the effects enabled by *J*_*m*,*n*_ including exciton delocalization, exciton transport, and exciton lifetime (see above Exciton Delocalization theory section). Mass *et al.* reported *J*_*m*,*n*_ values between dyes in the SQ DNA aggregates ranging from 65 to 68 meV, see Fig. 45. By replacing the SQ dyes with commercially available Cy5 dyes covalently attached *via* dual phosphodiester linkers to the HJ and simultaneously fitting the absorbance and CD data obtained from a Cy5 dimer, they extracted a *J*_*m*,*n*_ of 70 meV for the Cy5 dimer and the extracted orientation was also consistent with H-like stacking. Hence, the *J*_*m*,*n*_ values for SQ and Cy5 are quite similar. This is not surprising since the molar absorption coefficients of both SQ and Cy5 are very similar and *J*_*m*,*n*_ is proportional to the square of the transition dipole moment of the dyes, which is related to the square of the molar extinction coefficient. Additionally, since SQ dyes yield superior photostability and structural diversity over that of Cy5 dyes, and with their promising aggregate optical properties, SQ dyes may be another viable candidate for exploring applications requiring exciton delocalization in DNA structures.

**Fig. 46:**
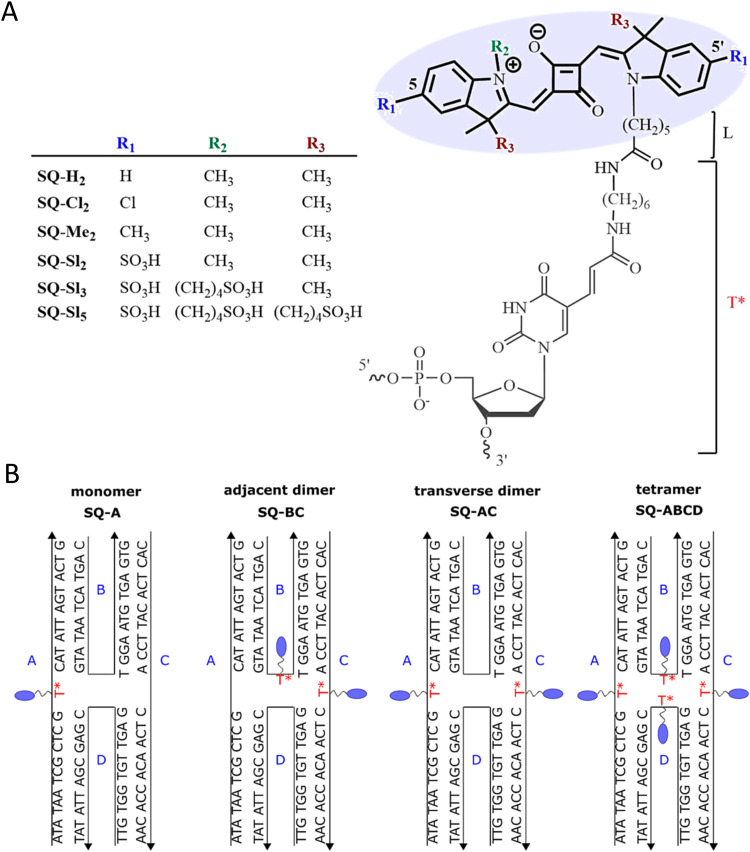
Hydrophobicity and excitonic coupling in DNA-templated indolenine SQ dye aggregates (A) Chemical structures of indolenine SQ (bold) attached to C6 thymine sequence modifier T*(gray). (B) Schematic representation of immobile DNA HJs templating DQ monomer SQ-A, adjacent dimer SQ-BC, transverse dimer SQ-AC, and a tetramer SQ-ABCD. The strands comprising DNA HJ are labeled A, B, C and D. This publication represents a partnership between Boise State University and SETA BioMedicals to address this topic area by designing custom synthesized SQ dyes to permit the necessary tuning of the side substituents. Reproduced with permission from ref. 378 Copyright 2022 American Chemical Society.

While covalent attachment of dyes to DNA for DNA templating of dye aggregates offers considerable control over that of DNA intercalation or groove templating, fine tuning of dye packing by varying side substituents of dyes to enhance excitonic coupling has been insufficiently explored. This is mainly because requisite dyes are not easily commercially available that have the broad range of substituents required for this type of study. Thus, Mass *et al.* undertook this topic area by designing custom-synthesized SQ dyes to permit the necessary tuning of the side substituents, see Fig. 46.^378^ SQ aggregation was driven by DNA HJ self-assembly as the SQ dyes were covalently linked to the constituent DNA strands forming DNA HJs with three different aggregate conformations: two dimers (adjacent and transverse) and a tetramer. Side substituents in five different positions on six SQ dyes were chosen such that dye hydrophobicity varied and the impact on excitonic coupling was assessed quantitatively in the form of *J*_*m*,*n*_. Exciton delocalization was again observed in each of the aggregates. Strong excitonic coupling in the form of blue-shifted peak absorbance intensity and CD couplet signatures indicating H-like behavior was observed in all cases. Large *J*_*m*,*n*_ and dye orientation and distance for each aggregate was again extracted using the KRM tool with an observed general trend revealing that *J*_*m*,*n*_ increased with increasing dye hydrophobicity. The largest *J*_*m*,*n*_ value, 132.2 meV (1065 cm^−1^), was reported for the dichloroindolenine squaraine SQ-Cl_2_ dye in an adjacent dimer configuration. The tetramer of the same dye supported a *J*_*m*,*n*_ value of 103.5 meV. Hydrophobicity was correlated relative to a partitioning coefficient calculated using DFT. Dye alignment was also found to be affected by other factors including dye sterics and electrostatics. This demonstrates that changing the side substituents of the SQ dyes can control the dye hydrophobicity and thus the stacking behavior of the dyes in an aggregate, which, in turn, provides a means to tune *J*_*m*,*n*_. Additionally, the achieved *J*_*m*,*n*_ values for dimers were more than twice that of dimers comprised of commercially available unsubstituted SQ dyes.^222^

**Fig. 47:**
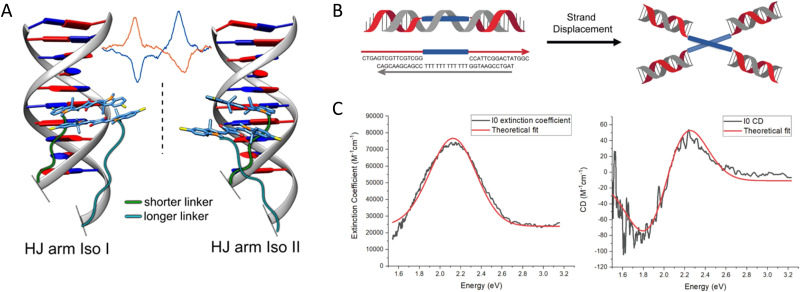
Exciton chirality inversion and delocalization in DNA-templated organic semiconductor dimer assemblies. (A) Structures of the two pairs of dimers of bis(chloroindolenine) SQ dye that enabled strongly coupled molecular excitons of opposite chirality in solution as seen in the CD spectra. Reproduced with permission from ref. 379 Copyright 2022 American Chemical Society. (B) Octaniline-DNA compositional monomer spontaneously assembled into the chiral dimer by adding triggering DNA strand. (C) Absorbance and CD spectral theoretical fits. Experimental data sets are shown as black lines, while theoretical fits are given as red curves. Reproduced with permission from ref. 380 Copyright 2022 American Chemical Society.

Another approach to fine-tune dye packing is by varying the length of the linker used to covalently attach the dye to the DNA scaffold. Mass *et al.* describes the effect of dye-DNA linker length to tune the packing behavior of dyes, in this case bis(chloroindolenine)squaraine, to enable desired optical properties.^379^ This approach was utilized to create two different dimers of opposite exciton chirality, both of which exhibited optical behavior consistent with strong exciton delocalization. Indeed, this approach of tuning dye packing *via* dye-DNA linker length appears to be a first of its kind. Specifically, one of these dimers was formed using unequal covalent linker lengths whereby one dye was tethered *via* a shorter linker to one of the four constituent DNA strands in a DNA HJ and another monomer dye was attached using a longer linker in the same DNA HJ using another one of the four specific strands. The second dimer was obtained by interchanging the two covalent linkers relative to the DNA strands. The CD results of the dimers showed couplets of nearly equal but opposite signs indicating that these dimers exist as virtually equimolar mixtures of enantiomorphs, see Fig. 47A. That is, exciton chirality inversion was achieved. Several control experiments were performed whereby three different linker lengths were used but for each control the same linker length was used for each dimer – chirality inversion was not observed for any of these controls. The orientation of each dimer as well as the *J*_*m*,*n*_ value between dyes in each of the aggregates was extracted using the KRM tool by simultaneously fitting absorbance and CD data. The results demonstrated that interchanging linker length forms dimers that relate as non-superimposable and nearly exact mirror images; the *J*_*m*,*n*_ value for dimer pairs ranged between 121 to 97 meV. The chirality inversion was attributed to the stacked HJ isomer bias (*i.e.*, a strong preference for one HJ conformational isomer templating the dimer over another) due to linker length interchanging. This isomer bias leads to a more homogeneous solution of HJs as compared to the control HJ solutions with equal length linkers without isomer bias, hence, tending to show greater heterogeneity. Mass *et al.* also point out an important challenge with using HJs as a DNA template in this study. HJs are prone to exhibiting two conformational isomers that, with templating dyes to form aggregates, potentially force dyes within the aggregate to orient differently depending on the HJ conformational isomer. If both conformational isomers have a similar probability to form, then at least two different aggregates may form leading to static heterogeneity. Any experimental and computational data would then be a mixture of the two conformational isomers, which could be problematic for excitonic device applications.

#### 5.1.3. Other DNA-templated dye aggregates

Wang *et al.* demonstrated exciton delocalization in an organic semiconductor aggregate comprised of two octaniline monomers forming a dimer using a dsDNA scaffold.^380^ Two excitonic switches were established with the capability of switching between the monomer and two different dimers. Specifically, switching between the octaniline monomer exciton state and the dimer exciton delocalization state was done using DNA strand invasion (see Fig. 47B and C). Another excitonic switch was actuated between one exciton delocalization state to another exciton delocalization state by changing the pH of the solution, which varied the protonation of the octaniline leading to changes in excited states. The absorbance peaks collected from these samples are quite broad and featureless, so CD was readily used in distinguishing exciton delocalization and the amount of Davydov splitting. Because the featureless absorbance peaks did not appear to show vibronic-induced peaks, a simple Frenkel Hamiltonian, neglecting vibronic effects, was used to model the data by simultaneously fitting the absorbance and CD spectra and to extract *J*_*m*,*n*_, dye orientation, and dye distance. The orientation of the dimers was predominantly H-like and the largest *J*_*m*,*n*_ extracted was 110 meV, which was for the protonated octaniline dimer at a pH of 4.5, revealing that it had the greatest extent of exciton delocalization in this system. However, the theoretical model used to extract *J*_*m*,*n*_ ignored vibrational effects, which can impact its value. As the authors point out, the absorbance peaks are quite broad which they attribute to an inherent feature of bandgap-forming organic semiconductors. Thus, the vibronic contribution to the absorbance of the dimers was obscured forcing the authors to simplify their theoretical model. Again, the switching times of these excitonic switches in solutions are in the minutes which is far from the picosecond switching timescale of microelectronic based switches for computing and memory applications.

Other dye types that have been used in a similar context include π-conjugated perylene diimides (PDIs) and porphyrins.^381,382^ In terms of PDIs, the Stulz group recently reported on the DNA-encoded assembly of these molecules with deterministic control over the number of electronically coupled molecules using phosphoramidite coupling chemistry. They discovered that the DNA guided these materials into specified close proximity where hydrophobic–hydrophilic differentiation drove aggregation and local geometry and electrostatic interactions defined intermolecular positioning. This resulted in the PDIs packing to give substantial intermolecular π wave function overlap and led to an evolution of singlet excited states from localized excitons in the PDI monomer to excimers with wave functions becoming delocalized over five PDIs in their pentamer structure.^381^

### 5.2. Towards coherent exciton transport in DNA-templated dye aggregates

The ability to control dye position on DNA scaffolds with a precision of about 3.6 Å (and in some cases also the dye orientation) provides a way to tune excitonic coupling between dye molecules.^111,169,367^ This aspect of DNA nanotechnology presents a unique opportunity to explore coherent ET in designer dye aggregates that display sufficiently strong excitonic coupling. The type of ET, coherent transport *vs* incoherent hopping, is determined by the size of the excitonic coupling between dyes (*J*_*m*,*n*_) with respect to the decoherence rate (*τ*^−1^_decoh_) of the dye aggregate excited state.^383^ In the weak coupling limit (FRET regime), 
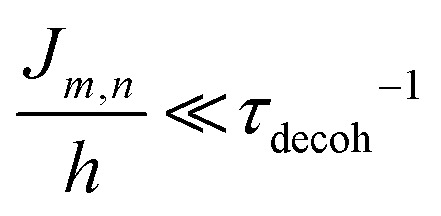
, where *h* is Planck's constant and ET occurs by incoherent hopping. In the strong coupling limit, 
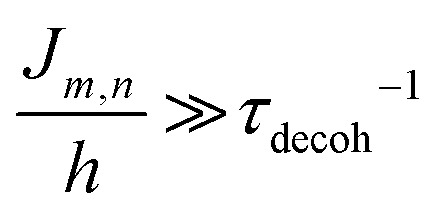
. Here, the coherently delocalized exciton can extend over many dyes and energy transport occurs in a wavelike manner. There is also an interesting intermediate regime which is thought to be used in photosynthetic systems where 
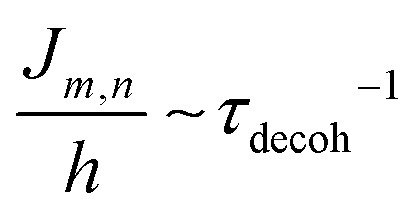
, and where coherent ET can also occur.^384,385^ The experimental signatures of coherent ET include the observation of oscillatory signals from excited state coherences.^386^ Currently, the most direct way to observe such coherences is through the application of ultrafast two-dimensional electronic spectroscopy.^387^ Several of the strongly-coupled dye aggregates on DNA discussed above are candidates for coherent ET, however, it remains for future experiments to elucidate definitive signatures. Below, we discuss a few representative examples of larger dye aggregates on DNA that display strong excitonic coupling and have the potential to exhibit coherent ET.

**Fig. 48:**
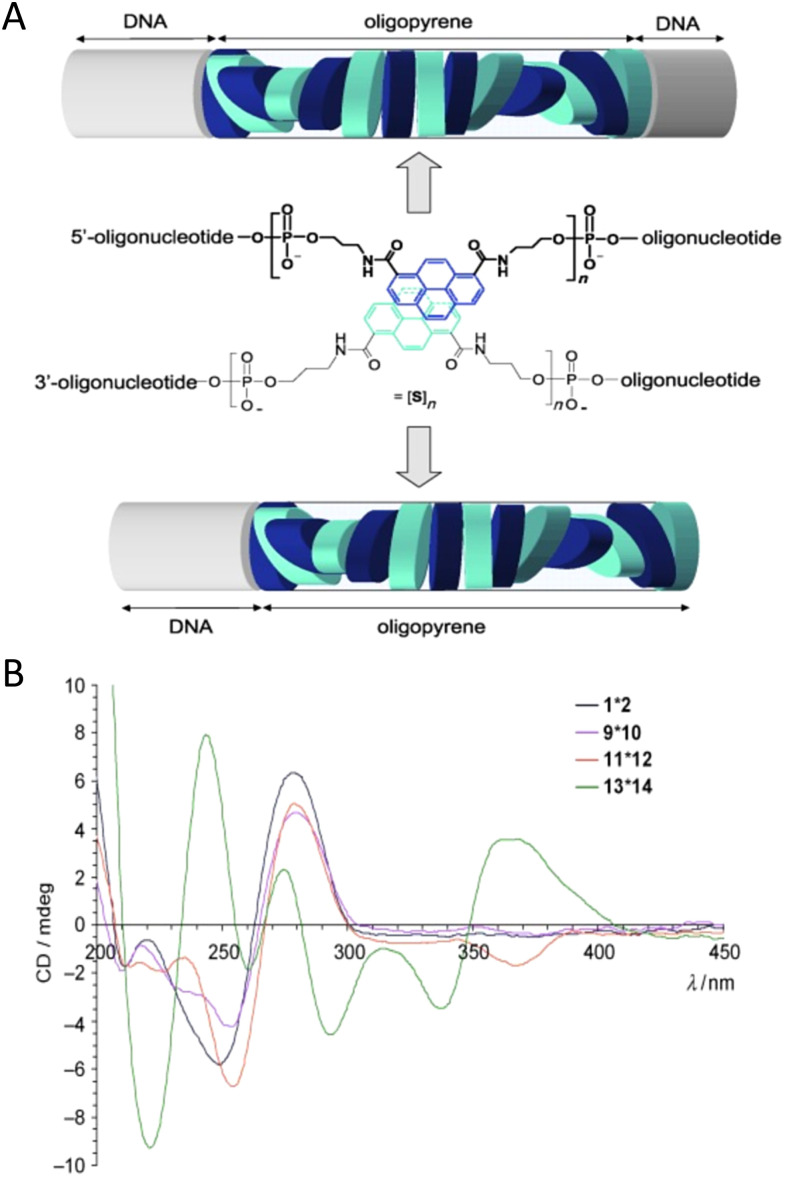
Exciton delocalization in DNA-templated pyrene stacks. (A) Schematics of DNA organized pyrene stacks. The central image presents the chemical structure for the constructs which consist of two complementary DNA strands into each of which has been inserted a pyrene oligomer consisting of *n* pyrene units. The upper image depicts the case of a pyrene aggregate, consisting of a stack of 14 pyrene units exhibiting a right-handed helical pitch, sandwiched between two duplex-DNA strands. The lower image depicts the case when dsDNA is absent at one end of the pyrene stack. (B) CD spectrum for various constructs consisting of pyrene stacks imbedded in a DNA duplex. A pyrene stack is absent in structure 1*2 hence, the CD spectrum exhibits the spectral features of duplex DNA alone. For structures 9*10, 11*12, and 13*14, the number of pyrenes in the stack are 8, 10, and 12, respectively. The CD spectrum of structure 9*10 exhibits no CD features at wavelengths longer than 300 nm, whereas features in this region appear for structures 11*12 and 12*14, indicating that the transition to helical ordering occurs when the number of pyrene units becomes greater than 10. Reproduced with permission from ref. 389 Copyright, 2009, John Wiley & Sons.

As mentioned previously, arrays of chromophores that can serve as wires to transport excitons coherently are of direct interest for optoelectronic device development and concerted efforts towards this have been undertaken by several researchers including that of the Häner Group. Along with describing in detail how such wires may be fabricated,^388,389^ the interested reader is also referred to an excellent review from Häner on how nucleic acids can be used to assemble aromatic chromophores.^390^ The excitonic wires studied by Häner consisted of stacks of pyrene molecules and were prepared from constructs consisting of an oligomer of covalently linked pyrene units sandwiched between two DNA oligomers. Two such constructs with an equal number of *n* pyrene molecules and complementary DNA segments form the structure, shown in the middle and upper image of Fig. 48A, which has a pyrene aggregate consisting of 2*n* pyrene units at the center.^389^ By characterizing how the optical properties of these aggregates change as the number of pyrene units was changed, it was inferred that when 2*n* is greater than 10, the aggregate adopts a configuration consisting of an ordered right-handed helical array as shown in the upper image of Fig. 48A. The stacking is induced by hydrophobic interactions between the pyrene units. The chirality of this aggregate of achiral pyrene units is induced by the duplex-DNA segments, which have a right-handed chirality, at the interfaces where the DNA contacts the pyrene aggregate. In contrast, when 2*n* is less than 10, the aggregate adopts a disordered configuration. Hypochromicity seen in UV-vis absorbance spectroscopy at low temperatures both in ss and dsDNA constructs indicates that the pyrene units engage in face-to-face (H) stacking. Experimental data providing support for an ordered helical array (when 2*n* is greater than 10) included fluorescence and CD spectra. The fluorescence spectra of the aggregates, regardless of whether ss or ds assemblies were used, exhibit a broad excimer peak located at approximately 500 nm. For 2*n* < 10, a red shift of the excimer peak was observed upon duplex formation. In contrast, for 2*n* > 10 a blue shift in the excimer peak was observed upon duplex formation. Since such blue-shifted fluorescence is generally observed in systems exhibiting only partially overlapping excimer geometries such as crystalline pyrene derivatives, pyrenophanes, bispyrenyl systems, and polymers with twisted or strained pyrene conformations this blue-shifted fluorescence served as evidence for a twisted stacking configuration when 2*n* > 10. Further support for this twisted stacking configuration comes from the CD spectra shown in Fig. 48B. An intense bisignate signal in the CD spectra from the pyrene band centered at 348 nm, with a positive Cotton effect at 365 nm followed by a minimum at 332 nm, indicates that the pyrene units are stacked in a right-handed helical array. These spectral features occur at longer wavelengths than the CD features produced by the duplex DNA. Surprisingly, it was found that duplex DNA was only needed on one end of the pyrene oligomers to produce a helical ordered array (Fig. 48A lower image), this was for the case when the aggregate contained 14 pyrene units. This structure exhibited properties similar, but more pronounced, to those of the structures with 12 or 14 pyrene units in the middle of a duplex DNA strand, thereby indicating that higher stacking ordering is achieved when one end of the pyrene stack is left unconstrained by a duplex-DNA strand.

**Fig. 49:**
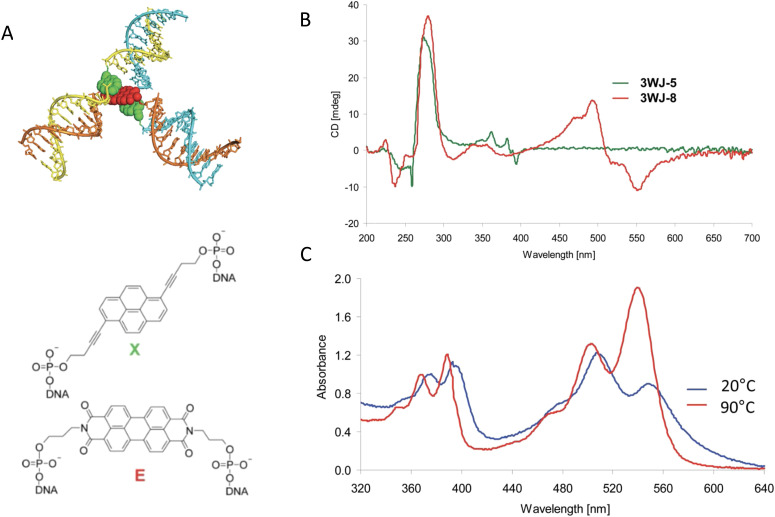
Exciton delocalization in a DNA three way junction. (A) Energy minimized modeling of the 3WJ-7 aggregate from the structure judged most consistent with observation. The three way junction is assembled from oligomers possessing chromophores or other modifications at their centers in order to bring these modifications in close proximity, as indicated by the colored chevrons. The structure formula for the alkynylpyrene modification is labeled X (green). The structure formula for the perylenediimide modification is labeled E (red). (B) CD spectra of the two homo-chromophoric 3WJs. 3WJ-5 (three alkynylpyrenes) and 3WJ-8 (three perylenediimides). (C) Normalized temperature-dependent UV-vis spectra of the 3WJ-7 structure. Reproduced with permission from ref. 392 Copyright 2012, the Royal Society of Chemistry.

The use of DNA assembled pyrene stacks as LH antenna were also investigated in several studies by Häner's group. In one study, the cyanine dye Cy5 was appended to the DNA free end of pyrene stacks having the same structural form as the lower image in Fig. 48A.^391^ The Cy5's absorbance peak, covering 500–700 nm, overlaps with that of the pyrene excimer fluorescence, 410–600 nm. The Cy5 thus serves as the A for energy deposited into the pyrene stack near the pyrene absorbance peak of 350 nm. The behavior of the ET efficiency as a function of the number of pyrene units in the pyrene stack was found to be consistent with transfer of excimer energy to the Cy5 by FRET. The flexibility of DNA nanostructures in the assembly of aggregates was also illustrated using a DNA three-way junction to organize a variety of chromophore aggregates.^392^ A rendering of the DNA construct along with structures of the chromophores employed is shown in Fig. 49A. DNA oligomers were designed, which upon hybridization, would form an immobile three way junction. As indicated by the colored segments at the center of the three-armed junction of a given oligomer the chromophore, alkynylpyrene (X) or the chromophore perylenediimide (E) was inserted. Alternatively a thymine base (T) was inserted at the center of the oligomer or nothing (−) was inserted. In this manner, 14 different three-way junctions were constructed employing the same set of three base sequences. Base sequences were also chosen so as not to have direct neighboring guanines next to the modifications in order to minimize the known fluorescence quenching of this base. The aggregate denoted as 3WJ-5, which we refer to as the XXX aggregate, contains the X modification in all three of the oligomers, thereby forming an alkynylpyrene trimer. The native absorbance spectrum exhibited multiple peaks with the 0–0 peak intensity greater than that of the 0–1 vibronic peak. In contrast, in the trimer the 0–0 peak intensity being less than that of the 0–1 vibronic peak indicating exciton delocalization within the XXX aggregate in the weak coupling regime for which the energy of the quantum of vibration dominates the exciton exchange energies or *J*_*m*,*n*_'s. Further evidence of exciton delocalization came from the CD spectrum of the XXX aggregate, which also indicated that the dyes in the aggregate adopt a chiral arrangement, see Fig. 49B. The fluorescence spectrum exhibited suppressed monomer emission and consisted primarily of excimer fluorescence. Aggregate 3WJ-6, an XEX aggregate, exhibited reduced excimer fluorescence relative to the monomer emission in comparison with the XXX aggregate. The absorption spectrum corresponding to the 440–600 nm range of the perylenediimide absorbance maximum exhibited a bathochromic shift and a hypochromic change relative to the absorbance spectrum of the single oligomers produced by dissociation upon heating to above the melting temperature of the three-way junction. Such a shift and change in the absorbance peak of the longest wavelength chromophore are expected when this chromophore participates in coherent exciton exchange with the other dyes of the aggregate.

**Fig. 50:**
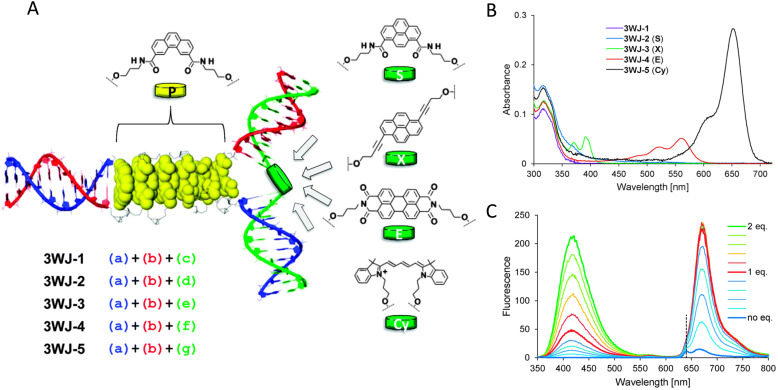
Modular LH complex built on the DNA three-way junction. (A) Schematic illustration of the LH 3WJ with the chromophores used and highlighting how the modularity of the structure provides for wide access to different configurations. Phenanthrene (P), pyrene (S and X), perylenediimide (E), and the commercially available cyanine (Cy5). (B) UV-vis spectra of the investigated three-way junctions; acceptors are given in brackets. (C) Build-up of 3WJ-5: fluorescence spectra after stepwise addition of strands. Reproduced with permission from ref. 394 Copyright 2014, the Royal Society of Chemistry.

In contrast to the above, the absorbance of aggregate 3WJ-7, an EXE aggregate, exhibited a bathochromic shift and a hypsochromic change in the 440–600 nm absorbance of the perylenediimide absorption maximum relative to the thermally dissociated oligomers, see Fig. 49C. The intensity of the 0–0 and 0–1 absorption bands of the perylenediimide peak underwent a reversal upon thermal dissociation much like that exhibited by the XXX aggregate in the 330–410 nm region. These observations along with the CD spectrum indicated that there is strong exciton exchange, or delocalization, between the perylenediimide chromophores of the EXE aggregate and that the dyes also adopt a chiral packing configuration. The absorbance spectrum of aggregate 3WJ-8, an EEE aggregate, exhibited behavior very similar to that of the XXX aggregate. The CD spectrum of the EEE aggregate showed a strong CD signal in the 440–600 nm range, indicating that the achiral perylenediimide chromophores adopted a configuration with substantial chirality. The versatility of using appropriate chromophore-modified DNA to assemble dye aggregates is well demonstrated with this work employing three-way junctions. A relatively small number of dye-modified DNA oligomers enables the combinatorial construction of a large number of aggregates whose optical properties can be compared to sort out photophysical processes. In further application of the combinatorial diversity that these same three way junctions constructed with chromophore-modified DNA could afford and building off previous related work,^393^ the same group investigated LH antennas.^394^ These antennas consisted of phenanthrene stacks constructed in much the same way as the pyrene stacks of the upper image of Fig. 48A, except that the DNA strands on one side of the stack are no longer complementary and, instead form into duplex arms with a third DNA oligomer also modified with a dye at its center that can serve as an A of the energy absorbed by the phenanthrene stack. A schematic of these antennas along with some representative photophysical characterization collected from different variants is shown in Fig. 50. Although not focused on delocalization *per se*, this versatile configuration enabled the testing of antenna performance with a variety of A chromophores that included two pyrene isomers, perylenediimide, and the Cy5 dye.

### 5.3. Comparison of molecular exciton theory with experiment

**Fig. 51:**
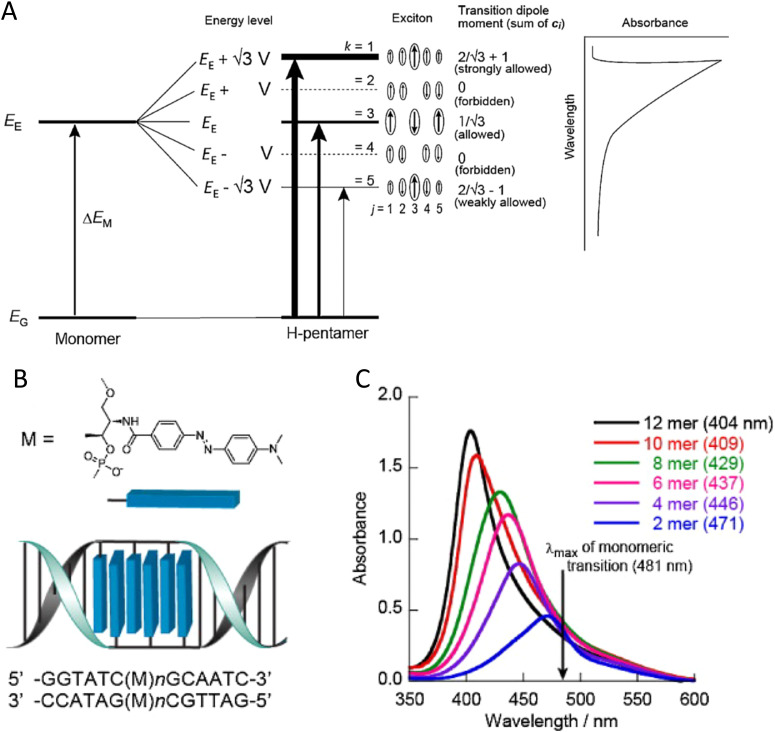
Exciton delocalization and comparison of theory to experiment. (A) A diagram showing the energy levels of an H-pentamer (middle) arising as a result of exciton delocalization over an array of five dyes. The magnitude of the total transition dipole for each state is given along with a schematic of the expected asymmetric absorbance spectrum. (B) Schematic illustration of the design of an H-aggregate (ladder-like assembly) prepared from methyl red on d-threoninol. (C) The UV–vis spectra as a function of the number of the dyes in the aggregate. Reproduced with permission from ref. 367 Copyright 2012, Elsevier.

In a review article,^367^ Asanuma and coworkers surveyed work on the construction of dye aggregates through the self-assembly of covalent dye-modified DNA and also described their own work on the construction of dye aggregates by this method in order to explore the photophysics of systems in which exciton delocalization occurs. In order to provide a comparison between theory and experiment for the case of linear homodimer aggregates, the theory of exciton delocalization in dimer aggregates was developed within the molecular exciton theory (Frenkel exciton theory) for which vibronic effects are neglected. This was extended to the case of exciton delocalization in aggregates consisting of *n* chromophores stacked in a row with either H or J stacking. The selection rules for transitions from the ground electronic state to the *n* molecular exciton states were determined along with the shape of the aggregate absorbance spectrum. The specific case when *n* = 5 is shown in Fig. 51. More pertinently, comparison of the theoretical expectations to experiment was undertaken by assembling H aggregates with varying number of methyl red chromophores. These dyes were inserted between two single-stranded DNA segments using D-threoninol as the scaffold as shown in the top structure of Fig. 51B. Two such constructs with complementary DNA and the same number of dyes results in an H-stacked dye array held together by the duplex-DNA strands formed upon hybridization, as shown in Fig. 51B lower image. As shown in Fig. 51C, the absorbance spectra are in qualitative accord with theoretical expectations. As the number of dyes increases, the shape of the spectrum becomes more asymmetrical and the absorbance peak exhibits a greater hypsochromic shift (shift to shorter wavelength) indicative of H-aggregate behavior. The authors, however, note that quantitative agreement with theory is not observed: “For example, theory predicts that the degree of hypsochromic shift of the dimer with respect to the monomeric transition should be half of the H-aggregate of infinite size. Experimental results show that the degree of hypsochromic shift of the dimer is only 441 cm^−1^ (481 nm → 471 nm or 54.7 meV), whereas that of the 12-mer is 3960 cm^−1^ (481 nm → 404 nm, or 491 meV).” The authors suggest that this discrepancy may be attributable to the asymmetric structure of the dye or to the use of the simple point-dipole approximation.

**Fig. 52:**
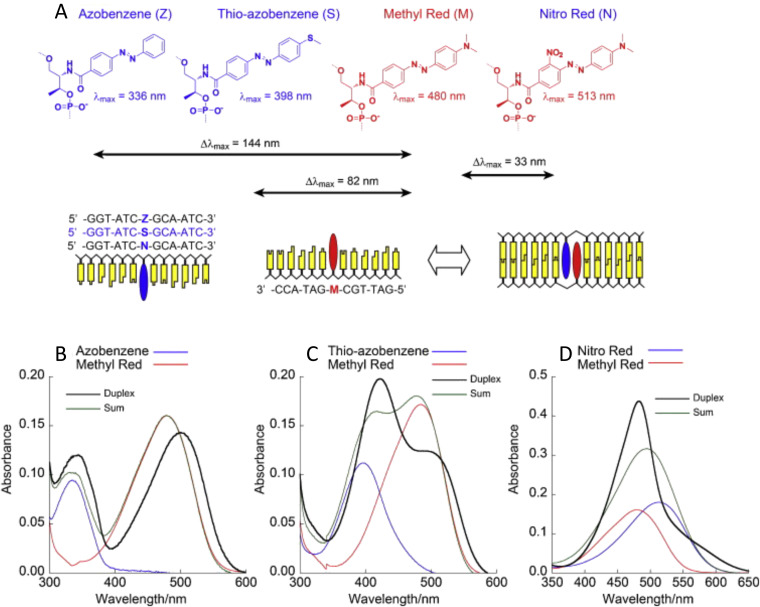
Heterodimer aggregates and comparison of theory to experiment. (A) Structure diagrams for the dyes and their linkage to the D-threoninol scaffold. The DNA constructs incorporating the dyes are also shown. (B)–(D) Absorbance spectra of the dye pair monomers (red) and (blue), the sum spectrum (green), and the dimer spectrum (black) for the dimers containing azobenzene, thio-azobenzene and nitro red, respectively. The methyl red monomer spectra are red. Reproduced with permission from ref. 367 Copyright 2012, Elsevier.

For a further comparison of molecular exciton theory with experiment, the authors also considered the case of heterodimer aggregates. Since by definition the two dyes are different in an H-stacked heterodimer, their dipole moments will differ. Thus, in general, the perfect cancellation of dipole moments in an H-stacked homodimer that results in an optically forbidden transition from the ground state to the lowest energy excited state is relaxed in an H-stacked heterodimer. As a result, the absorbance spectrum will generally exhibit two peaks, Δ*E*_D_^+^ for the higher energy peak and Δ*E*_D_^−^ for the lower energy peak. In terms of the optical transition energies Δ*E*_M1_ and Δ*E*_M2_ for dyes 1 and 2, respectively, of the heterodimer, the location of the dimer absorption peaks is given by:22
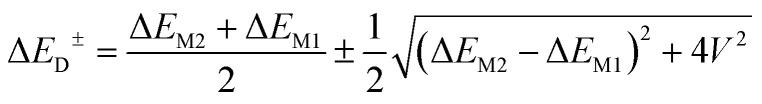
Here *V* is the exciton exchange energy, which in the point dipole approximation is identical to *J*_*m*,*n*_ given by eqn (15). One sees from this that relative to Δ*E*_M2_ the peak Δ*E*_D_^+^ is shifted to a higher energy (shorter wavelength) by the same amount that relative to Δ*E*_M1_ the peak Δ*E*_D_^−^ is shifted to a lower energy (longer wavelength). Similarly, one expects the oscillator strength of the Δ*E*_D_^+^ to increase with respect to that of Δ*E*_M2_ while the oscillator strength of the Δ*E*_D_^−^ to decrease with respect to Δ*E*_M1_ as the difference between Δ*E*_M2_ and Δ*E*_M1_ becomes small since in the limit when they are equal one should recover the selection rules of the homodimer. These predictions were subsequently checked against the experimental results of Fujii *et al.*^395^ Heterodimers were constructed in which the methlyl red (M) dye was paired with one of the three dyes azobenzene (Z), thio-azobenzene (S), and nitro red (N), as shown in Fig. 52. The experimental absorbance spectra of the monomers, the sum spectrum of the monomers, and the dimers are shown in Fig. 52B–D for the dye pairs containing Z, S, and N, respectively. In each case, the long wavelength peak of the heterodimer exhibits bathochromicity (*i.e.*, red-shift) and hypochromicity (*i.e.*, absorbance decreases), in accord with theory while the short wavelength peak exhibits hyperchromicity (*i.e.*, absorbance increase) which was also in accord with theory. However, the short wavelength peak exhibits bathochromicity instead of the theoretically expected hypsochromaticity (*i.e.*, red-shift). Apart from this discrepancy, good qualitative agreement between theory and experiment was again observed in these experiments. It was suggested that the discrepancy was due in part to the point dipole approximation and that the orbital overlap between the two azo-dyes may raise the ground state level.^367^

**Fig. 53:**
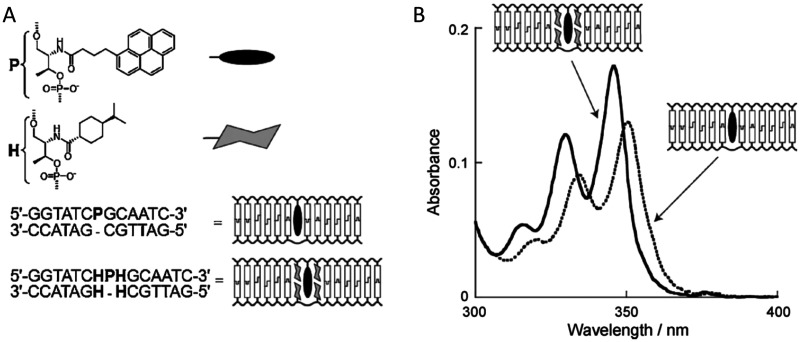
Use of insulator pairs. The use of an artificial base that is optically neutral to investigate exciton delocalization between a dye and natural bases in a DNA duplex. (A) The structure P of the dye (pyrene), the artificial base H (*trans*-isopropylcyclohexane), and schematics of the DNA constructs. (B) The UV-vis absorbance spectrum of the DNA constructs with and without the neutral base pairs. Note the shift to longer wavelength and lower absorbance of the construct without the neutral base pairs. This shift indicates delocalization into the natural DNA bases of the exciton associated with the dye. Reproduced with permission from ref. 367 Copyright 2012, Elsevier.

In many previous examples, the unique addressable properties of the DNA scaffold have been employed to help verify particular aspects of FRET processes such as that of the ‘sheet regime’ effects on distance dependency.^103^ The ease with which dye aggregates, having contrasting aggregate structures, can be constructed through DNA-hybridization guided self-assembly has similarly aided in the identification of the physical mechanisms responsible for aggregate spectral features. Asanuma and colleagues had previously pioneered the use of spacer molecules for this purpose.^369^ This was nicely illustrated with the use of optically inert spacer molecules, that served as “insulator base pairs,” to determine the origin of spectral shifts observed when dyes intercalate into DNA. Specifically, the authors sought to determine the mechanism responsible for the bathochromicity (shift to longer wavelength) and hypsochromicity (reduced absorbance) of the lowest absorbance peaks of pyrene. Possible explanations for the bathochromicity include decrease in the local polarity in the DNA duplex compared to buffer solution, stabilization of the first excited state to reduce the HOMO–LUMO gap, or, in the case of the intercalating dye ethidium bromide, the acquisition of an electron from the negatively charged phosphate ion of the DNA backbone to raise the HOMO level. None of these explanations, however, account for the hypsochromicity that is commonly observed when dyes intercalate into DNA. In accord with the discussion of heterodimers, an alternative explanation for bathochromicity that accounts for hypsochromicity is exciton delocalization between the dye and neighboring bases of the DNA.^396^ To test this last hypothesis, two DNA–dye constructs were created. In one, the pyrene was sandwiched between natural DNA bases. In the other, optically neutral artificial bases were inserted between the pyrene and the natural DNA bases. The artificial bases consist of the molecule *trans*-isopropylcyclohexane whose structure is given as H in Fig. 53A. Also, shown is the pyrene structure P along with schematics of the DNA constructs. The experimental absorbance spectra for the two constructs are shown in Fig. 53B. Insertion of the neutral bps resulted in a blue shift and increased absorbance of the low-lying absorbance peaks. On this basis, the authors attribute bathochromaticity and hypsochromaticity commonly observed with intercalating dyes as mainly due to the coherent interaction of the dye with the natural base pairs. This insulator technique also provides a way to distinguish between intercalation and major or minor groove binding.

### 5.4. Exploiting excitonic phenomena in the design of sensitive hybridization probes

**Fig. 54:**
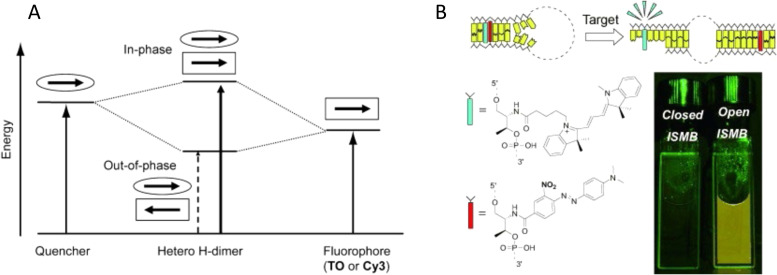
Exciton delocalization in a highly sensitive molecular beacon. (A) Energy diagram of the heterodimer of fluorophore and quencher depicted on the basis of the exciton model. Each arrow, outlined by an ellipse or an oblong, designates the transition dipole moment. (B) Depiction of how excitonic interaction can be utilized to design a highly sensitive in-stem molecular beacon (ISMB) in which both a fluorophore and a quencher on D-threoninols are incorporated as a pseudo base pair (as shown with the optimized combination with Cy3-green and modified Methyl Red-red). Minimization of the difference between *λ*max of the fluorophore and quencher maximized quenching efficiency. Reproduced with permission from ref. 397 Copyright, 2010, John Wiley & Sons.

Building from the previous examples, it has also been demonstrated that exciton delocalization can be used as a basis to enhance the sensitivity of fluorescent DNA probes.^397^ The sensing mechanism behind these probes are based on the principle that quenching of dye fluorescence often occurs when two dye molecules are brought close to each other. Borrowing from common FRET parlance, the dye whose fluorescence is quenched is said to be the D and the dye that causes the quenching, which need not itself be fluorescent, is said to be the A. In contrast to FRET, the much shorter-range mechanism by which fluorescence quenching occurs is referred to as contact quenching. This occurs when dye molecules come into physical contact with each other and thereby open rapid non-radiative pathways by which the energy of the excited D can be dissipated. At these contact distances, short-range exciton interactions are operative and may be used to improve the quenching efficiency. To demonstrate such enhanced quenching, Asanuma and coworkers used DNA dye constructs similar to those used for their H dimer studies, see Fig. 52A. The two fluorophores employed included thiazole orange (TO) with an absorbance max at 516 nm and Cy3 with an absorbance max at 550 nm. The quenchers consisted of multiple azobenzene derivatives, which had an absorbance maximum of 513, 480, 394, and 334 nm, respectively. It was found that the fluorescence quenching efficiency strongly correlated with the degree of hypsochromicity (a proxy for the degree of exciton delocalization in the H dimer) and, hence, was greatest for the construct containing D quencher pair Cy3-Z; the pair for which the fluorophore and quencher absorbance maxima are most closely matched, see Fig. 54. In terms of sensitivity, the authors were able to obtain a remarkable ratio of the fluorescence intensities with and without the target present (*I*_open_/*I*_closed_) at 564 nm of as high as 70 under the conditions employed.^397^ It is worth considering the possibility in this example that the quenching from charge transfer is so rapid that the delocalized state did not have a chance to become fully-established.

**Fig. 55:**
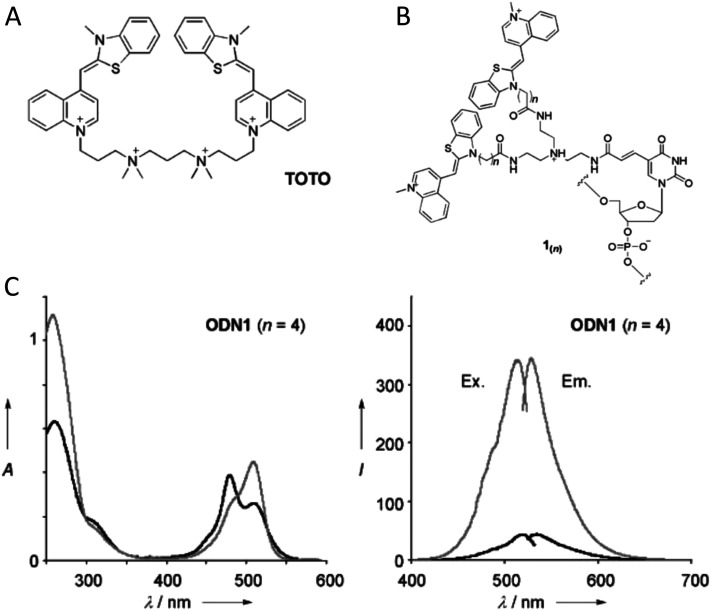
Exciton delocalization in hybridization-sensitive on–off DNA probe. (A) Structure of the TO homodimer TOTO. (B) Structure of the newly synthesized fluorescent nucleotide, 1_(*n*)_ where *n* refers to the length of the carboxyl linker utilized. (C) Absorption, excitation, and emission spectra of *n* = 4 containing oligodeoxynucleotides or ODN. Absorption spectra are shown in the left-hand panel and excitation and emission spectra are shown in the right-hand panel. Black trace: single-stranded ODN (ss); gray trace: ODN hybridized with the corresponding complementary DNA (ds). Reproduced with permission from ref. 398 Copyright, 2008, John Wiley & Sons.

In similarly innovative work, Ikeda and Okamoto had demonstrated the use of excitonic phenomena in the design of sensitive hybridization probes for application in gene analysis.^398^ The tendency of strongly exciton-coupled aggregates to exhibit strong fluorescence quenching was exploited to make a fluorescence probe for which fluorescence was suppressed unless the probe was interacting with a target. For this purpose, a nucleoside was synthesized that was covalently labeled with a homodimeric form of two TO dyes (TOTO) that were covalently linked as shown in Fig. 55A and B. When attached to a ssDNA probe, the fluorescence of the dye construct is quenched. When the probe hybridized with a complementary target the dye construct fluoresced. The quenching was attributed to H aggregate stacking of the two dye molecules. According to Kasha's model, the quenching resulted from rapid vibrational thermalization to the lowest excited state of the aggregate for which an optical transition to the ground state is forbidden. We suggest an alternate potential explanation for this example in that an increase in the rate of non-radiative decay is responsible for the quenching. This explanation would be consistent with the observed presence of two absorbance peaks in the probe spectra suggesting that the aggregates are not perfectly H stacked but instead have more of an oblique stacking for which optical transitions to the ground state from the lowest excited state are allowed. Quenching by this mechanism is commonly observed in dye aggregates where the dyes contain methine bridges. When the probe hybridized with the complementary target, the dyes separated and intercalated within the DNA giving rise to fluorescence. Evidence for the transition from an H aggregate to separated dyes upon the interaction of the probe with the target comes from the shift in the absorption maximum from 480 nm to 510 nm, the absorbance maximum of a single TO dye, upon hybridization of the probe with the target DNA, see Fig. 55C. The enhanced fluorescence is attributed to restriction of rotation about the methine bridge between the two heterocycle systems when the dye is intercalated into the DNA. A TO dimer having the trade name TOTO along with many of its structural analogues are commonly used as fluorescent DNA stains.^336,399^ By covalently linking such a dimer to a ssDNA, a probe is created that is sensitive to the DNA sequence of the target. This probe was demonstrated in a variety of genetic assays including dot blot hybridization.

Further investigations on this same TOTO dimer DNA hybridization probe approach were also undertaken.^400^ In this subsequent work, the dependence of probe sensitivity on base sequence was studied. The fluorescence intensity of 30 different probe sequences was measured and it was found that G/C pairs neighboring the labeled nucleotide resulted in reduced fluorescence quenching of the probe before hybridization. Temperature dependent studies were also performed and indicated that the reduced fluorescence quenching resulted from the pairing of probes to form probe dimers. It was also found that hybridization-enhanced fluorescence occurred even if the labeled nucleotide was at the end of the probe strand. This extensive study enabled the authors to make recommendations on good probe design. Overall, such a probe design has advantages over standard molecular beacons (MBs) in that only one dye type is required, the need for the probe to have intra-strand complementary regions to produce secondary structure in the probe is eliminated, and the dyes need not be attached to the ends of the probe oligomer which opens a greater range possibilities for biological applications such as in PCR-based DNA amplification.

### 5.5. Dynamics of molecular excitons in DNA-templated dye aggregates

#### 5.5.1. Excited-state quenching

**Fig. 56:**
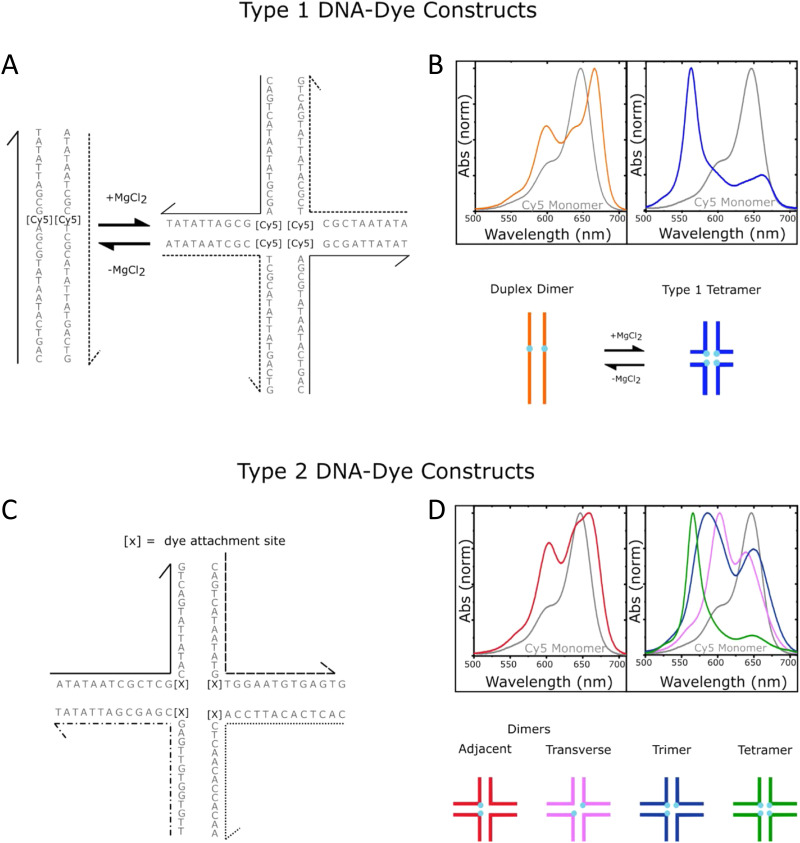
Schematics of DNA-templated cyanine (Cy) aggregates and associated optical spectra. (A) Type 1 DNA–dye constructs formed by mixing two complementary Cy5-labeled oligonucleotide strands in an aqueous buffer solution in the absence (left) and presence (right) of added MgCl_2_. Duplex DNA is the primary nanostructure in the absence of added MgCl_2_, whereas DNA HJs are formed when MgCl_2_ is added. There is one Cy5 dye per strand, and therefore, the duplex and HJ template the aggregation of a Cy5 dimer and tetramer, respectively. (B) Normalized absorption spectra of the duplex dimer and “mobile” HJ tetramer. The lower part of panel (B) depicts schematic illustrations of the duplex dimer and HJ tetramer, where cyan circles indicate the approximate location of Cy5 dyes. (C) Type 2 DNA–dye constructs formed by mixing four mutually complementary oligonucleotide strands in an aqueous buffer solution with MgCl_2_ added. The solutions primarily consist of “immobile” HJs. Cy5 dyes are covalently tethered to two, three, or four positions within the HJ. (D) Normalized absorption spectra of the two dimer species (adjacent and transverse), trimer, and tetramer formed *via* “immobile” Hjs. The lower part of panel (D) depicts schematic illustrations of the dimer, trimer, and tetramer structures, where cyan circles indicate the approximate location of Cy5 dyes. The schematic depictions of the dye-labeled duplex and HJ structures are not meant to imply specific geometric configurations of the dye or DNA backbone. Reproduced with permission from ref. 402 Copyright 2021 American Chemical Society.

A number of studies have shown that nonradiative decay rates and quenching generally increase, sometimes drastically, upon dye aggregation, and that this holds true for many DNA-based structures as well. Clearly, understanding the fundamental mechanisms that govern such phenomena in DNA-based assemblies will help in the design and further examination of their dynamic optical properties. Cunningham *et al.* used steady-state and time-resolved absorption and fluorescence spectroscopy to examine the impact of local bp sequence and increasing bp separation on the dynamics in Cy3 dimer aggregates templated *via* DNA duplexes.^110^ For 0 and 1 bp separation, Cy3 aggregates were observed with intermediate to strong electronic coupling (150–266 cm^−1^; 19–33 meV). Local bp sequence was found to change the packing type, *i.e.*, biasing either an H- or J-like packing arrangement. With decreasing bp separation and increasing coupling strength, the excited-state lifetime decreased. For example, the average lifetime of the 0 bp separation Cy3 dimer was ∼0.4 ns, which was ∼2.8 times shorter than the Cy3 monomer lifetime of ∼1.1 ns. As mentioned above, Cannon *et al.* showed that packing type can be changed by altering the salt content of the buffer solution and the primary DNA nanostructure in the solution.^374^ Specifically, the authors showed that without any salt added DNA duplexes templated Cy5 dimers with largely J-aggregate packing whereas the addition of an appreciable concentration of salt promoted the formation DNA HJ Cy5 tetramer aggregates with H-aggregate packing. The Cy5 dimers and tetramers exhibited drastic changes to their steady-state absorption spectra as a result of strong electronic coupling strengths quantified in the amount of ∼400–1200 cm^−1^ (50–150 meV).^401^ Huff *et al.* investigated the excited-state dynamics of DNA duplex and HJ templated Cy5 dimers and tetramers, respectively. The authors found that both systems exhibited drastically increased nonradiative decay rates compared with the corresponding monomer. While nonradiative decay comprised ∼71% of the overall decay in the monomer (*τ* = 1.5 ns), it comprised >99% of the overall decay in the Cy5 dimer and tetramer (*τ* = 11 and 35 ps, respectively) and thus was the primary contributor to excited-state relaxation. In follow-up work, Huff *et al.* performed a comprehensive analysis of a broad set of DNA duplex and HJ templated Cy5 dimers, trimers, and tetramers,^402^ including the “immobile” HJ Cy5 dimers, trimer, and tetramer originally reported by Cannon *et al.*, see Fig. 56.^377^ Using a combination of transient absorption spectroscopy and global target analysis, the authors visualized the drastically reduced lifetimes in the aggregates (∼10–200 ps) as compared with the monomer (∼2 ns) along with considerable variability of lifetimes, even within individual aggregate solutions. Based on a comparison of the steady-state optical properties of the aggregates, including structural parameters derived *via* optical modeling, it was determined that in relation to excited-state lifetime there was a strong and weak correlation with dye separation and exciton delocalization, respectively. Further, the authors explained the considerable variability of lifetimes measured in individual solutions as arising from various sources of structural heterogeneity, including multiple distinct DNA HJ conformations, *i.e.*, isomers,^222^ and DNA “breathing”.^114^

**Fig. 57:**
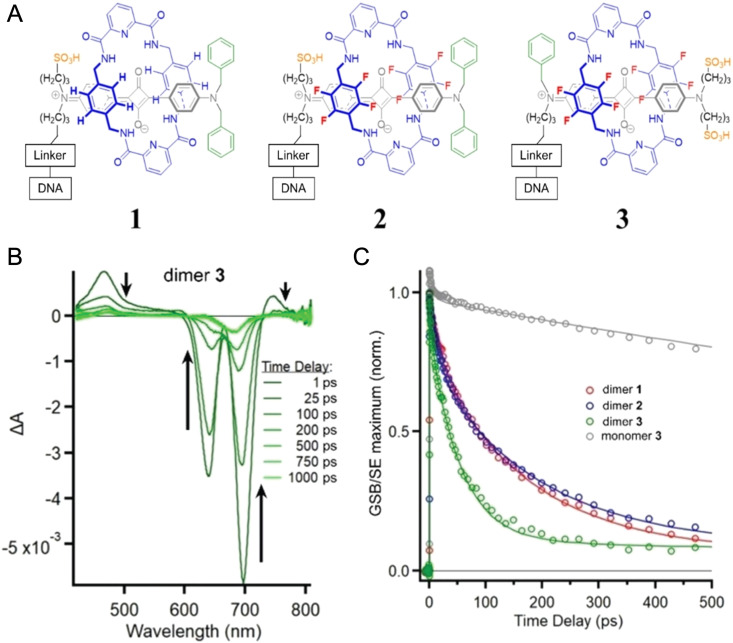
Tunable excitonic coupling in DNA-templated SQ:rotaxane dimer aggregates. (A) SQ:rotaxane dyes 1, 2, and 3 covalently attached to a DNA oligonucleotide using a thymine base modified with a six-carbon linker (C6dT). To help visualize the different parts of the rotaxane, the SQ and macrocycle are drawn in gray and blue, respectively. To facilitate comparison between structures, sulfonic acid, fluorine, and alkyl phenyl substituents are drawn in orange, red, and green, respectively. (B) Spectra at selected time delays for dimer 3 pumped at 695 nm. Black arrows are plotted alongside the transient absorption spectra pointing in the direction of increasing time delay. (C) Kinetic traces for each SQ:rotaxane dimer taken at the maximum amplitude of each respective GSB feature. Additionally, the corresponding kinetics for monomer 3 are shown; only monomer 3 was plotted because all monomers had a similar lifetime of *ca.* 2.4–2.6 ns. Reproduced with permission from ref. 404 Copyright, 2022, John Wiley & Sons.

**Fig. 58:**
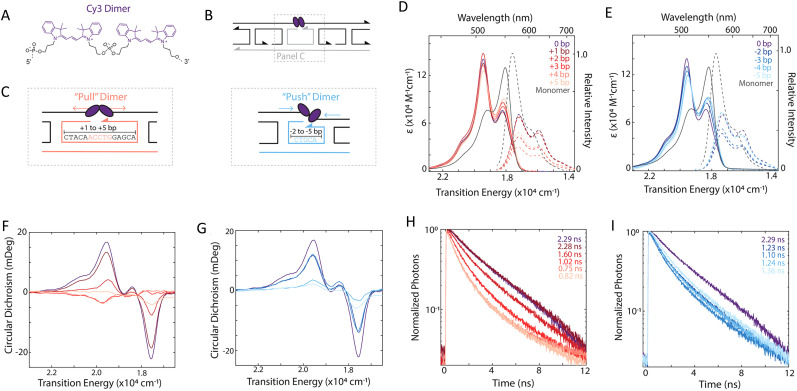
Electronic transitions of Cy3 dimers upon the addition or removal of central crossover strand bases. (A) Chemical structure of the Cy3 dimer. (B) Schematic of the Cy3 dimer that is covalently bridged within a DX tile. The tunable central crossover strand and long strand are shown in gray. (C) Schematic and sequences for Cy3-DX tiles upon the addition (left, “pull”-type) and removal (right, “push”-type) of central crossover and long strand bases. Modified strands are highlighted in light red and light blue for the “pull”-type and “push”-type DX tiles, respectively. Absorption (solid) and emission (dashed) spectra of (D) “pull”-type and (E) “push”-type dimers. Emission spectra are normalized by relative quantum yield for 530 nm excitation. Circular dichroism spectra of (F) “pull”-type and (G) “push”-type dimers. Fluorescence decay traces with 520 nm excitation for dimers formed with the (H) addition, or “push”-type, and (I) removal, or “pull”-type, of central crossover strand bases. Emission was detected at 580 nm. Reproduced with permission from ref. 405 Copyright 2022 American Chemical Society.

More recently, Barclay *et al.* showed that excited-state quenching in DNA-templated dye aggregates is not restricted to the cyanine family of dyes.^403,404^ Specifically, the authors templated the aggregation of SQ and SQ:rotaxane dyes, the latter of which were originally designed to gain finer control of dye aggregation. When attached to transverse sites in a DNA HJ, the authors found that the use of a bulky rotaxane macrocycle surrounding the SQ chromophore promoted an oblique dimer, whereas SQ dimers without the rotaxane ring formed H-like dimers. However, even with the introduction of bulky macrocycles to form SQ:rotaxanes, it was shown that excited-state quenching still persisted, though the excited state lifetime was extended by approximately a factor of 1.7 compared to the SQ dimer without the rotaxane (Fig. 57). The authors observed *τ* ∼ 2.9 ns for monomers of both dyes, and *τ* ∼ 75 and 130 ps for dimers of SQ and SQ:rotaxane dyes, respectively. Because accelerated *τ* due to superradiance can only occur for J-type aggregates and because the SQ and SQ:rotaxane dimers formed H-type and “oblique” aggregates, respectively, it was concluded that the accelerated *τ* was due to enhanced nonradiative decay in the SQ and SQ:rotaxane dimers. In a follow-up study, Hart *et al.* designed a series of DNA crossover (DX) tile templated aggregates in an attempt to understand the influence of structure on the dynamics of Cy3 dimers.^405^ Experimentally, the Cy3 dimers were “pushed” or “pulled” by either removing or adding, respectively, nucleotides in a central crossover strand of the DX tile along with its complementary long strand that completes the DX tile (Fig. 58). Although the dye aggregate packing did not vary considerably, *i.e.*, the Cy3 dimers remained in an H-aggregate packing configuration, when the dimers were either “pushed” and “pulled”, the authors found that in general nonradiative decay rates increased further. While the Cy3 monomer in the study exhibited *τ* ∼1.5 ns, the 0 bp dimer exhibited an average *τ* ∼2.3 ns and the +4 and −3 bp dimers exhibited the shortest average *τ* of 0.8 and 1.1 ns, respectively. Thus, the nonradiative decay rates were generally of the same value or larger compared with the monomer and, interestingly, the radiative decay rates were always smaller, which was consistent with subradiant behavior expected for the Cy3 dimers that exhibited optical properties consistent with H-type packing. The latter behavior may explain, in part, the larger *τ* observed for the 0 bp dimer compared with the Cy3 monomer, although the nonradiative decay rates were similar, the reduced radiative decay rate of the 0 bp Cy3 dimer may be responsible for its larger *τ*.

**Fig. 59:**
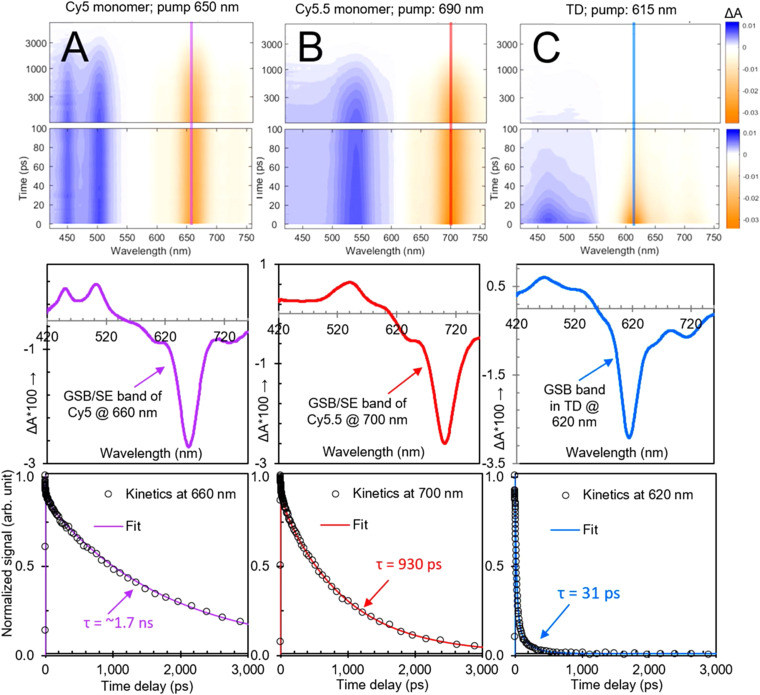
Tuning between quenching and energy transfer in DNA-templated heterodimer aggregates. Transient visible absorption of (A) Cy5 monomer, (B) Cy5.5 monomer, and (C) transverse heterodimer solutions pumped at 650, 690, and 615 nm, respectively, corresponding to each solution's absorption maximum. Surface plots are on the top, selected spectra are (at 5 ps) in the middle, and selected kinetics are plotted at the bottom. In the surface plots, vertical lines are included at the probe wavelength corresponding to the selected kinetics at the bottom. Selected spectra in the middle row show spectrally distinct ground-state bleach, stimulated emission, and excited-state absorption features for all samples. In the selected kinetics plots corresponding to the ground-state bleach maxima, fits from a global target analysis overlay the kinetics traces. The global target analysis derives lifetimes of 1.7 ns, 930 ps, and 31 ps for Cy5 monomer, Cy5.5 monomer, and transverse heterodimer, respectively. Reproduced with permission from ref. 406 Copyright 2022 American Chemical Society.

Recently, Chowdhury *et al.* and Huff *et al.* have explored excited state dynamics of Cy5–Cy5.5 heteroaggregates on DNA HJs.^406,407^ In the case of Cy5–Cy5.5 heterodimers, Chowdhury *et al.* showed that changing the position of the dyes at the core of the DNA HJ tunes the excitonic coupling between the dyes, which in turn dramatically tunes the excited state dynamics. When the dyes occupied adjacent sites on the HJ the coupling was relatively weak, resulting in ultrafast (sub-500 fs) energy transfer from Cy5 to Cy5.5. In contrast, when the dyes occupied transverse sites the excitonic coupling was much stronger, which produced rapid (∼50 ps) quenching of the excited state (Fig. 59). In the case of Cy5-Cy5.5 heterotetramers, Huff *et al.* observed tuning of the main absorption band over 200 meV (1600 cm^−1^), which was accompanied by broadening of the absorption band as the tetramer composition was varied. All of the tetramers showed more than a ten-fold reduction in excited state lifetimes compared to the monomers on the same HJ scaffold. Perhaps surprisingly, given the change in dye composition and absorption band broadening, the authors observed rather small variations in the excited state lifetime, between 40 to 60 ps. This result suggests that each tetramer experienced a similar non-radiative relaxation mechanism. Lastly, Cunningham *et al.* showed in DNA duplex templated Cy3 and Cy5 dimers that nonradiative decay pathways could be mitigated at cryogenic temperatures.^110,408^ The authors showed that whereas Cy5 dimers at room temperature exhibited lifetimes of ∼10 ps, the lifetimes at 100 K increased >2 ns. Thus, progress has been made in identifying a potentially challenging aspect of leveraging DNA-templated dye aggregates for applications, *i.e.*, the prevalence of excited-state quenching, and a number of studies have begun to shed microscopic insight into the nature of the process and how it may ultimately be mitigated. A challenge that remains is to better understand the mechanism of excited state quenching in strongly coupled Cy and SQ dimers on DNA templates.

#### 5.5.2. Static disorder, dynamic disorder, and structural heterogeneity

**Fig. 60:**
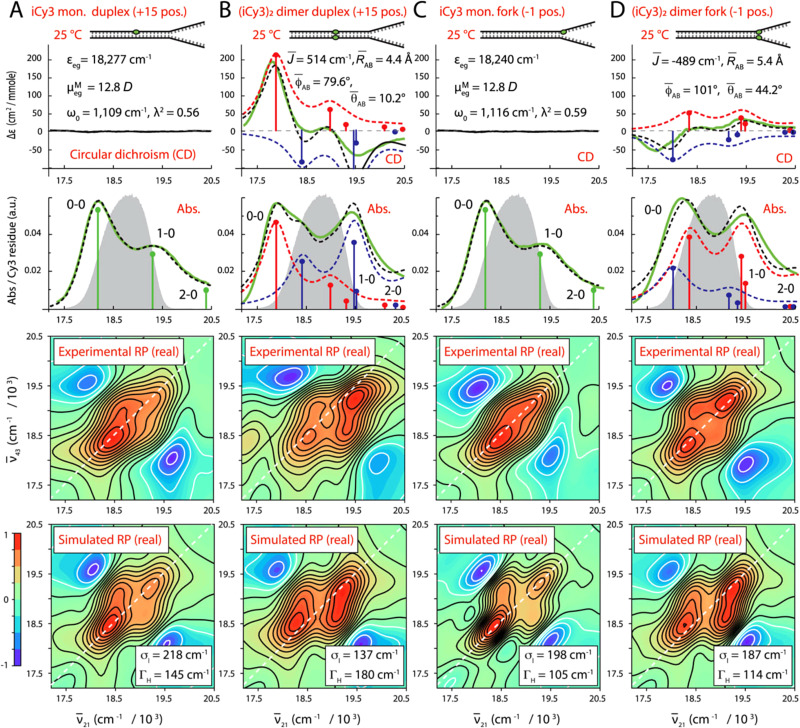
Temperature-dependent conformations of cyanine dimer labeled ss–ds DNA junctions. Experimental and simulated spectroscopic measurements performed at room temperature for (iCy3)2 dimer ss–dsDNA constructs as a function of probe labeling position. (A) iCy3 monomer +15 ss-dsDNA construct, (B) (iCy3)2 dimer +15 ss–dsDNA construct, (C) iCy3 monomer −1 ss–dsDNA construct, and (D) (iCy3)2 dimer −1 ss-dsDNA construct. The experimental CD (top row) and absorbance spectra (second row) are shown (in green) overlaid with vibronic spectral features (black dashed curves) obtained from optimized fits to the Holstein–Frenkel model. The symmetric (+) and anti-symmetric (−) excitons are shown in blue and red, respectively. Values of the optimized parameters are shown in the insets of the corresponding panels. The laser spectrum (in gray) is shown overlaid with the absorbance spectrum. Experimental rephasing spectra (third row) are compared to the optimized simulated rephasing spectra (fourth row). Reproduced with permission from ref. 410 Copyright 2022, the Royal Society of Chemistry.

Heussman *et al.* used absorption, CD, and two-dimensional (2D) fluorescence spectroscopies to study the average local conformations and the degree of conformational disorder of Cy3 dimer aggregates placed in various regions of a DNA fork (Fig. 60).^371,409,410^ Structural parameters, including the coupling strength, intermolecular distance, twist and tilt angles were derived by modeling the absorption and CD spectra using an essential-state Holstein–Frenkel Hamiltonian. The authors found the dyes to pack closely with intermolecular distances of ∼4–5 Å at room temperature and also found evidence for intermediate-to-strong coupling between dyes in the Cy3 dimer as evidenced by coupling strengths of ∼400–500 cm^−1^ (50–62 meV), which were considerably larger than the values derived for inhomogeneous broadening. Thus, the Cy3 dimers support delocalized, collective excitations, *i.e.*, molecular excitons. The authors also showed that at elevated temperatures approaching the ds-ss DNA denaturation transition, the intermolecular distance increased and the electronic coupling strength decreased. Lastly, they showed that the 2D fluorescence spectra of the Cy3 dimers generally exhibited inhomogeneous and homogeneous linewidths of ∼200 and ∼100 cm^−1^ (25 and 12 meV), respectively, and that these values were sensitive to the local environment of the Cy3 dimer aggregate probes when situated within the duplex or in the vicinity of the ss-ds DNA fork. Most recently, groups at MIT and Bern investigated Cy3 *n*-mer aggregates using steady-state absorption, CD, and several fluorescence spectroscopies including time-resolved fluorescence, two-dimensional electronic, and single-molecule spectroscopies.^411^ Two major conclusions of the study were: (i) electronic coupling and system-bath coupling could be tuned separately, with the former tuned by intermolecular distance, and (ii) DNA template rigidity influenced energy transfer. Heterogeneity was also observed and the extent of heterogeneity was influenced by the nature of the DNA template. In a different approach, Rolczynski *et al.* studied disorder in Cy3 and Cy5 homodimers and constituent monomers on DNA duplexes by making temperature-dependent measurements of absorption, fluorescence, and fluorescence excitation spectra from room temperature to 78 K.^372^ These measurements showed continual line narrowing of the fluorescence and fluorescence excitation spectra as the temperature was lowered from room temperature, though the reduction in linewidth flattened out substantially at 78 K, indicating that the 0–0 linewidths at 78 K were dominated by inhomogeneous broadening. For the Cy3 dimer, the fluorescence 0–0 peak linewidth was 162 cm^−1^ (half width at half maximum) at 78 K. The authors observed that the fluorescence spectra of the dimers were consistently narrower than the fluorescence spectra of the monomers at all temperatures, which they attributed to an exchange narrowing effect proposed by Knapp.^412^ Relatively small Stokes shifts between 51–55 cm^−1^ were extracted from the 78 K absorption and fluorescence spectra, which confirmed the formation of J-like dimers. While wavelength-dependent fluorescence and fluorescence excitation spectra indicated the presence of sub-populations in the ensemble of dimers (more so for Cy5 dimers than for Cy3 dimers), due to either different geometrical configurations and/or different local environments, the 0–0 fluorescence linewidths remained independent of excitation wavelength, which indicated that the sub-ensemble have similar degrees of inhomogeneous broadening.

**Fig. 61:**
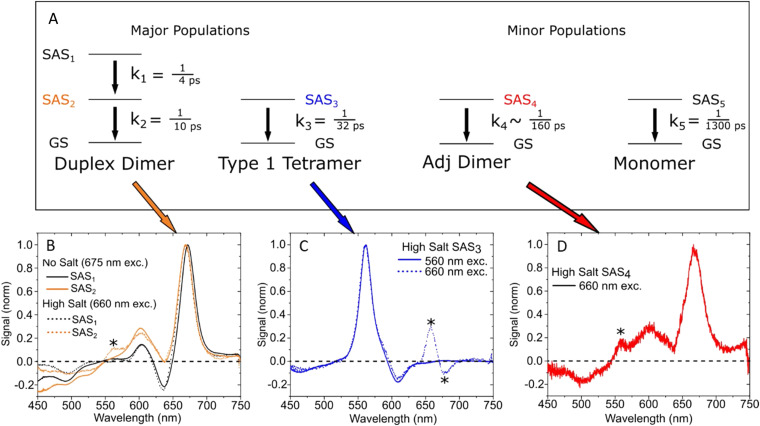
Global target analysis of transient absorption of DNA-templated cyanine dimer, trimer, and tetramer aggregates. (A) Five-component kinetic scheme used to model the high-salt type 1 solution. Four subpopulations—duplex dimers, type 1 tetramers, adjacent dimers, and monomers—decay in parallel in the model. The duplex dimers additionally exhibit a sequential decay in the model. (B) “Pure” species-associated spectra (SAS) derived for the duplex dimer from the no-salt type-1 solution pumped at 675 nm (solid lines) and SAS from the heterogeneous data set*, i.e.*, the high-salt type-1 solution pumped at 660 nm, attributed to the duplex dimer (dotted lines). SAS1 are plotted in black, and SAS2 are plotted in orange. (C) “Pure” SAS derived for the type 1 tetramer from the high-salt type-1 solution pumped at 555 nm (solid line) and the SAS from the heterogeneous data set pumped at 660 nm, attributed to the type 1 tetramer (dotted line). (D) SAS from the heterogeneous data set attributed to the fourth component, which we assign to a small subpopulation of adjacent dimer-like structures present in the high-salt type-1 solution. Reproduced with permission from ref. 402 Copyright 2021 American Chemical Society.

Huff *et al.* used a combination of pump-wavelength dependent femtosecond transient absorption spectroscopy and global target analysis to shed insight on structural heterogeneity in a series of DNA duplex and HJ-templated Cy5 dimer, trimer, and tetramer aggregates.^402^ Specifically, the authors examined solutions consisting of Cy5 aggregates templated *via* duplex DNA, “mobile” DNA HJs (that are known to consist of a mixture of duplex DNA and HJs),^374^ along with “immobile” HJs.^377^ Using pump-wavelength femtosecond transient absorption spectroscopy, the authors showed that the transient absorption spectra of the duplex DNA templated Cy5 dimer aggregates, which exhibit an absorption spectrum (and ground-state bleach features) characteristic of a J-like aggregate, and the transient absorption spectra of the HJ templated Cy5 tetramer aggregates, which exhibit an absorption spectrum (and ground-state bleach features) characteristic of an H-aggregate, could be extracted from an experiment specifically designed to simultaneously excite both of these subpopulations in the heterogeneous solution, see Fig. 61. The results simultaneously confirmed the nature of the heterogeneity in the solution and highlighted the ability of global target analysis to extract the pure transient absorption spectra of the underlying populations. Additionally, the analysis highlighted several limitations of global target analysis, including component mixing, which occurs when the spectra and/or temporal characteristics of the subpopulations are similar. Lastly, the authors examined solutions of Cy5 dimer, trimer, and tetramer aggregates templated *via* immobile DNA HJs. The authors found most of these solutions to exhibit varying degrees of structural heterogeneity, with the exception of the Cy5 tetramer aggregate solution.

## 6. A view from the application space

FRET-based DNA-fluorophore constructs have now been an object of study and/or widely used in various direct applications for almost three decades.^413,414^ While FRET has been applied in different classical and next-generation sequencing approaches,^233,415^ biosensing and molecular diagnostics have been, and continue to be, the most popular use for combining DNA, fluorophores, and FRET.^117^ Nucleic acids labeled with dye FRET-pairs have found frequent use in DNA and RNA target sensing *via* hybridization assays or in protein and small molecule target sensing *via* DNA-aptamers. These applications are discussed in several informative books and review articles.^117,416–421^ The critical role of dye-labeled nucleic acids and FRET in diagnostics cannot be overstated – almost all current molecular tests for corona virus use PCR and dye-labeled probes. As of Jan 1st, 2023, the U.S. Food and Drug Administration (FDA) had provided emergency use authorization (EUA) to more than 275 such tests.^422^ Most of these DNA-based FRET diagnostic tests exploit some form of reverse transcription quantitative PCR (RT-qPCR) methodology targeting the presence of SARS-CoV-2 viral RNA in patient-derived samples. Commercial versions of these tests are commonly implemented on various platforms including LightCycler™ (Roche), TaqMan™ (ThermoFisher Scientific), or Scorpions™ (Qiagen).^423,424^ All of the above-mentioned DNA-FRET technologies can be regarded as somewhat traditional methods that are based on relatively simple DNA structures, *e.g.* single oligonucleotide MBs, and FRET-pair combinations. To exemplify the versatility of combining DNA nanotechnology and FRET in a more sophisticated manner, we focus on some specific applications where larger DNA structures and dye networks were used to place single or multiple FRET pairs at distinct distances in order to design highly specific and, in some cases, even multiplexed FRET signals for biosensing,^425–437^ encoding of information,^438–440^ computing and data storage,^295,426,441^ and even for lasing on the single-molecule or ensemble level.^442,443^

**Fig. 62:**
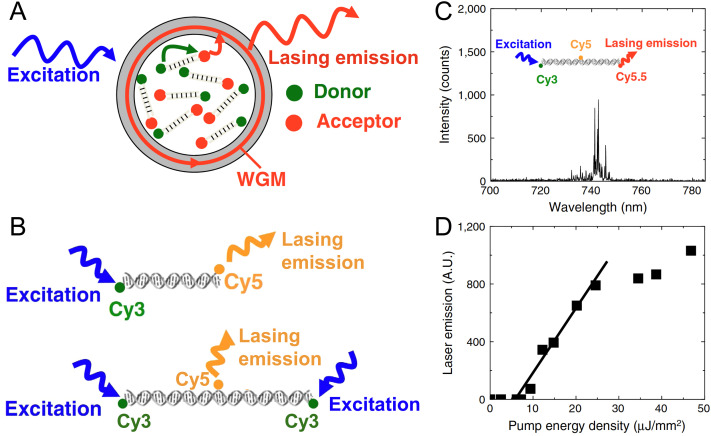
DNA-FRET lasers. (A) Schematic concept of OFRR FRET lasers using DNA scaffolds. (B) Cy5 D lasing could be accomplished *via* 1 : 1 (top) and 2 : 1 (bottom) D:A DNA constructs, which resulted in decreased lasing thresholds (2.3 μJ mm^−2^ for 2 : 1 *vs.* 4.2 μJ mm^−2^ for 1 : 1 for the emission centered at 720 nm). (C) Lasing emission spectrum of a DNA-based cascade-FRET (Cy3–Cy5-Cy5.5) OFRR laser. (D) Lasing emission (integrated between 720 nm and 760 nm) of the cascade-FRET laser was accomplished with a lasing threshold of 6 μJ mm^−2^ pump energy density per pulse. Reproduced with permission from ref. 443 Copyright 2010 National Academy of Sciences.

Optofluidic lasers represent a potentially interesting and useful technology because they combine compactness with ease of microfluidic liquid manipulation. Using FRET-pairs instead of a single dye type can extend the lasing wavelength range without changing the pump wavelength and result in higher pump efficiencies and lower lasing thresholds. However, to accomplish FRET from unconjoined dyes in solution, dye concentrations in the millimolar range are required. To overcome this issue, Sun *et al.* used dsDNA to place D and A dyes at distinct distances in order to overcome the necessity of using such extremely high concentrations.^443^ The dye-DNA constructs were placed in an optofluidic ring resonator (OFRR) and the whispering galley modes (WGMs) in the OFRR were used to initiate lasing at dye concentrations down to ∼2.5 μM which was ∼1000 fold lower compared to single-dye optofluidic lasers, see Fig. 62A. Lasing with the initial 12 bp dsDNA construct where a Cy3 D and Cy5 A were coupled to the 3′ ends of the complementary ssDNAs was further improved by using a 2D-1A system. The DNA scaffold used for this consisted of a 24 bp dsDNA with two Cy3 Ds on the 3′ termini of the ssDNAs and an internally DNA-labeled central Cy5 A (Fig. 62B). The same dsDNA was further used to design a FRET-cascade laser consisting of Cy3 D → Cy5 relay → Cy5.5 A, which further extended the lasing wavelength to the red with a lasing threshold of approximately 6 μJ mm^−2^ (Fig. 62C, D). DNA tetrahedrons using three Cy3 Ds and one Cy5 A (as well as the inverse) were also used for lasing in a similar OFRR-WGM setup,^442^ demonstrating that DNA-based lasing could be extended to more sophisticated DNA-scaffolds. The complex mix of interdisciplinary skill sets required to develop and utilize such fascinating hybrid materials has probably limited the number of researchers who can undertake and further develop this technology.

Another interesting application of FRET-DNA scaffolds is the creation of specific fluorescence signals that can be used for developing physically unclonable functions (PUFs). PUFs are physical security keys for encryption with a large number of input-output combinations that should ideally be impossible to decrypt. A first prototypical demonstrator of DNA–dye PUFs was designed by using DNA grids. Three different dyes could be conjugated at many possible positions on the grid, which resulted in highly specific time-resolved fluorescence signals.^440^ The input of the PUF consisted of the excitation wavelength and the temporal delay between excitation pulses, which led to distinct time-resolved fluorescence histograms. To avoid the rather complicated spectroscopic equipment required for time-resolved fluorescence measurements on the nanosecond timescale, it was demonstrated that the temporal component of a DNA-based FRET PUF can be implemented *via* the bioluminescent Luc enzyme.^439^ Using a DNA dendrimer and a 4-step BRET/FRET cascade consisting of (Luc)_8_ → (AF488)_8_ → (Cy3)_4_ → (Cy3.5)_2_ → (Cy5.5)_1_, the different spectral components of BRET and FRET and the time-dependent emission intensity of the luciferase-catalyzed coelenterazine substrate were used to generate the PUF codes. Here, BRET is initiated by addition of the substrate while FRET is initiated by excitation with an external light source. The input of the PUF consisted of the excitation wavelength and/or addition of substrate and the read time (in minutes); this resulted in a distinct combination of FRET and BRET intensities from the different luminescent components, which were then recorded by a standard fluorescence spectrometer. By considering a matrix of excitation wavelengths, spectral channels, intensity of those channels, and distinct analysis times - 1.4 × 10^12^ potential challenge/key combinations were obtained for the BRET-only PUF. Considering the overlapping characteristics of the FRET/BRET key jointly results in an estimated total of 7 × 10^37^ challenge/key combinations. The Lux lab has also reported the use of dye-labeled DNA for anticounterfeiting, in this case using toehold-mediated strand-displacement reactions to create a pattern that can be validated by smartphone. When the object is tagged with the correct combination of DNA sequences, a pattern appears within minutes, with minimal cross-reactivity, and shelf-stability approaching two years.^444^ Though neither of these technologies are application translation ready at the moment, they offer up alternatives for future consideration and demonstrate how the specificity of DNA in positioning of fluorophores can be exploited for information storage.

**Fig. 63:**
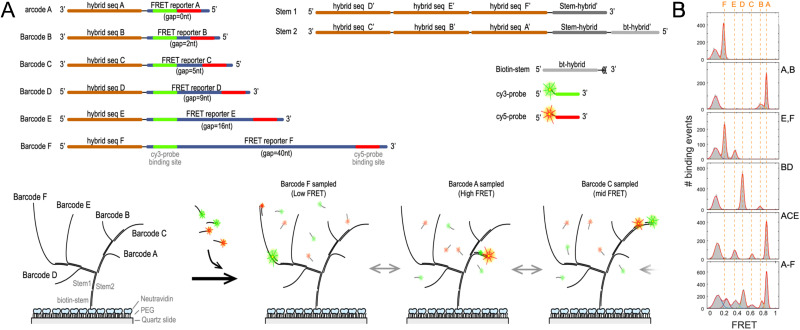
Single-molecule FRET DNA barcoding. (A) Schematic presentation of the different stem, barcode, and probe DNAs (top) and the hybrid DNA complexes with six (Barcodes A, B, C, D, E, and F) FRET barcodes. (B) FRET efficiency distribution peaks (using one, two, three, or six barcode DNAs) collected from a single field of view. Solid red lines are Gaussian fits to each subpopulation. Dashed red lines present the position of each subpopulation. The peaks around 0.1 FRET efficiency correspond to the D only (no FRET) subpopulation. Reproduced with permission from ref. 438 Copyright 2021 American Chemical Society.

Optical DNA barcoding exploits the programmability of DNA to label different biomolecules with distinct codes for multiplexed detection. Fluorescence imaging is one of the major techniques for visualizing DNA barcoding and also performing subsequent sensing with them. Instead of relying on fluorescence colors or lifetimes for barcoding, Kim *et al.* used *E*_FRET_ and single-molecule detection on a total internal reflection fluorescence (TIRF) microscope to distinguish six different FRET codes on a DNA scaffold (Fig. 63).^438^ The DNA constructs were attached *via* two partially hybridized ssDNA stem sequences to Neutravidin-coated quartz slides. Each of the two ssDNA branches provided three sequences for the hybridization of six additional ssDNAs. These six barcoded ssDNAs included specific sequence regions to which Cy3 D probes and Cy5 A probes could bind at distances of 0, 2, 5, 9, 16, and 40 bps (Fig. 63A) resulting in six distinct Cy3–Cy5 D–A distances. By optimizing the single-molecule imaging conditions and probe sequence length along with concentration, transient hybridization of the probes to all six different barcode DNA branches could be distinguished over time *via* distinct *E*_FRET_ distributions (Fig. 63B). This ingenious approach allowed the authors to create six virtual FRET channels *via* conventional two-color (Cy3 and Cy5) TIRF microscopy, which can potentially be extended to additional color channels for even higher multiplexing capability. An additional advantage of FRET compared to single-fluorophore detection is the efficient distinction between unbound (no FRET) and bound (FRET) probes which makes assays using this approach potentially more reliable.

**Fig. 64:**
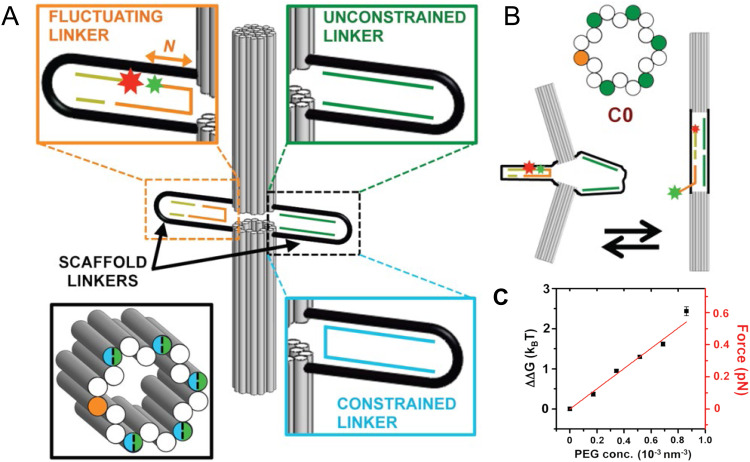
Environmental sensing with DNA origami FRET constructs. (A) FRET-DNA force sensor containing two large DNA barrels connected by one fluctuating (orange) and five unconstrained (green) or constrained (blue) scaffold linkers to control the conformational space. (B) Closed (left) and open (right) conformations of the C0 construct (without constrained linkers) result in a D–A (green and red stars) distance change. (C) The DNA-FRET sensor could quantify depletion forces induced by molecular crowding using different PEG_35_ concentrations. Reproduced with permission from ref. 432 Copyright 2017 American Chemical Society.

FRET-based DNA origami networks have also been applied as force, pH, and voltage sensors.^429,432,435^ Huboda *et al.* used DNA origami to design a DNA scaffold that consisted of two DNA barrels connected by six DNA linkers for use as a nanoscale force sensor, see Fig. 64A.^432^ One linker, the so-called fluctuating linker, could transiently hybridize to the Cy3 D and Cy5 A FRET probes, which were designed to provide thermally driven dissociation dynamics on the time scale of a few seconds. The other five linkers were either flexible (unconstrained linker) or non-flexible (constrained linker) depending on the hybridized staple strands. These linkers were used to shift the equilibrium to the closed or open state. This flexibility resulted in an equilibrium tuning range from ∼84% open (for 1 fluctuation and 5 unconstrained linkers – C0) to ∼14% open (for 1 fluctuation and 5 constrained linkers – C5) DNA constructs. Single-molecule FRET measurements of the C0 DNA scaffolds (Fig. 64B) attached to a quartz slide in a flow cell with different concentrations of 35 kDa PEG revealed different free energy barriers (ΔΔ*G*_PEG_) between the open and closed states. The compressive depletion forces from molecular crowding induced by the PEG molecules were then determined with a resolution of ∼100 fN by dividing ΔΔ*G*_PEG_ by the gap (or distance) difference between the open and the closed state (Fig. 64C). Transfection or direct microinjection of these DNA-FRET force sensors into cells could potentially provide a means to a direct quantification of intracellular depletion forces and molecular crowding in cellula.

**Fig. 65:**
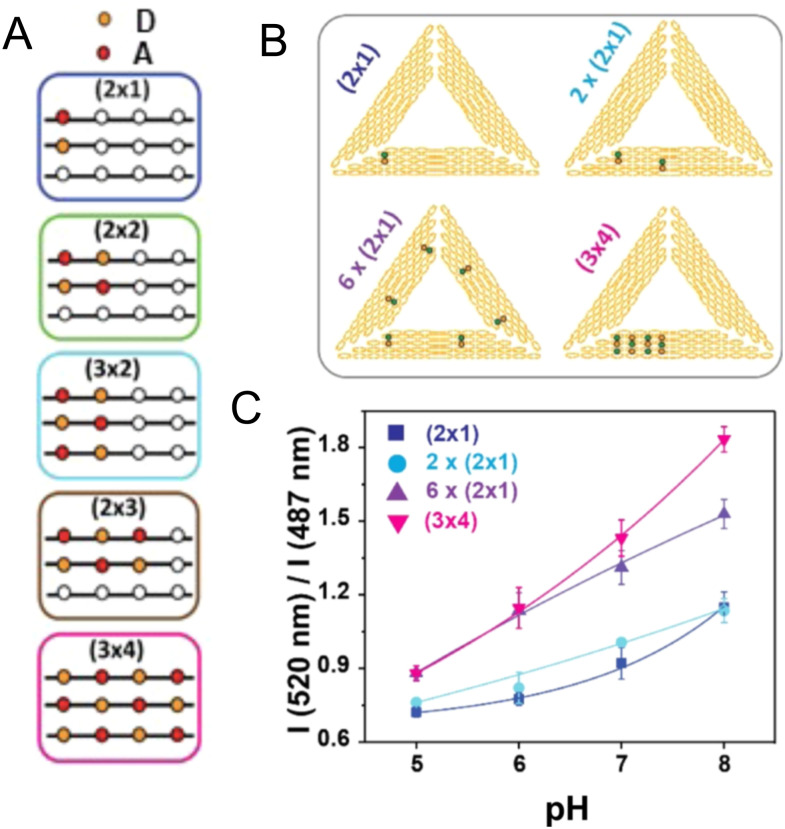
FRET pH sensor. (A) FRET-DNA pH sensor using different D–A arrangements with 12 possible D and A positions. (B) Different FRET arrangements (pH-inert C343 D in green and pH-sensitive FAM A in red) on triangular DNA origami were used for ratiometric pH sensing. (C) A 3 × 4 checkerboard arrangement resulted in the best pH sensing performance. Reproduced with permission from ref. 429 Copyright 2018 American Chemical Society.

Choi and coworkers used triangular DNA origami to create a pH sensor by attaching dye FRET pairs at specific distances and in specific D–A patterns to be able to tune fluorescence intensity and *E*_FRET_ without dye aggregation and the concomitant fluorescence quenching that it could induce (Fig. 65).^429^ D–A distances were ∼7.7 nm on the same dsDNA and ∼6.0 nm on the neighboring dsDNA. The authors found that a 3 × 4 checkerboard pattern with 6 D and 6 A provided the highest *E*_FRET_ and highest A/D fluorescence intensity ratio, which also corresponded well to theoretical estimations based on the D–A distances and the two fluorophores used (FAM as D and Cy3 as A). Taking advantage of the simple adaptability to different dye probes, they used a pH-inert C343 D and the pH-sensitive FAM A to convert the FRET DNA triangles into a ratiometric pH sensor. To evaluate if FRET benefits derived simply from the number of dyes or were also influenced by their arrangement, they tested 2 × 1, 2 × (2 × 1), and 6 × (2 × 1) patterns, in which the different FRET pairs could not interact with each other. These results were then compared with a 3 × 4 checkerboard pattern, which had the same amount of dyes as the 6 × (2 × 1) arrangement (Fig. 65B). Both the amount of dye and their advantageous arrangement improved the sensing performance with excellent pH sensitivity in the pH 5–8 range (Fig. 65C).^429^ The modular design of these FRET DNA origami structures suggests them as being imminently adaptable to many other FRET pairs and thus potentially amenable to other FRET-based sensing applications.

**Fig. 66:**
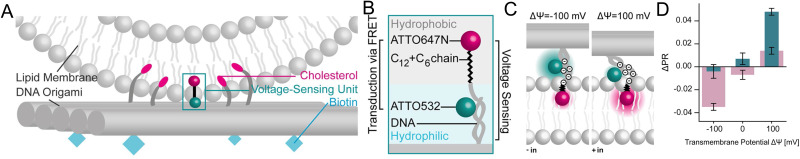
DNA-FRET voltage sensor including various functionalities on a single DNA origami platform. (A) Schematic of the sensor interface with the liposome membrane. (B) Voltage-sensing unit consisting of dsDNA with ATTO532 D and ATTO647N A dyes. (C) Probable working principle of the voltage-sensor, for which ATTO647N remains anchored in the hydrophobic core of the membrane, whereas the anionic ATTO532-DNA is influenced by the charge inside the liposome, which results in D–A distance changes. (D) The FRET distance changes can be used to distinguish transmembrane potentials with different sensor constructs (red: C_12_ linker on ATTO647N; green: C_12_ + C_6_ linker on ATTO647N). Reproduced with permission from ref. 435 Copyright 2021 American Chemical Society.

A DNA origami FRET voltage sensor designed to probe transmembrane potentials was prototyped by Ochmann *et al.* in a format that confirmed the sensing functionality using single-molecule TIRF microscopy on liposome samples.^435^ The 70 × 100 nm rectangular DNA origami included the voltage sensor and further cholesterol moieties for liposome binding along with biotin for attachment to Neutravidin-coated microscope cover slips (Fig. 66A). The voltage sensor component itself consisted of an ATTO532-ATTO647N D–A FRET pair within a dsDNA protruding vertically from the DNA origami, see Fig. 66B. The cationic dye ATTO647N was attached to one DNA strand *via* a C_12_-phosphate-C_6_ linker, such that its extreme hydrophobicity allowed it to penetrate into the lipid core of the membrane upon origami-liposome binding. The anionic dye ATTO532 was attached to the other DNA strand, such that it was placed closer to the origami platform. To verify the idea that a change in the transmembrane potential (Δ*Ψ*) should lead to a change of the relative positions of the oppositely charged dyes and a concomitant change in FRET, the authors induced a potassium ion gradient across the lipid membrane using valinomycin, a lipid-soluble molecule that binds K^+^ ions and transfers them across lipid bilayers, to convert it into a well-defined Δ*Ψ*. Changing from hyperpolarized (Δ*Ψ* = −100 mV) to depolarized (Δ*Ψ* = 100 mV) states was hypothesized to have resulted in the displacement of the anionic ATTO532-DNA outside the membrane (due to changing charges inside the liposome), whereas the ATTO647N remained at its anchored position within the membrane (Fig. 66C). The change in the proximity ratio (A emission divided by D + A emission upon D excitation) was only a few percent but could be distinguished by both the original voltage sensor and a shorter version where the linker of ATTO647N was shortened to a single C_12_ chain (Fig. 66D).^435^ In addition to FRET-based voltage sensing on the lipid membrane of liposomes, this study showcased the versatility of DNA origami in multiple ways including having several different molecular functionalities on the same DNA platform.

**Fig. 67:**
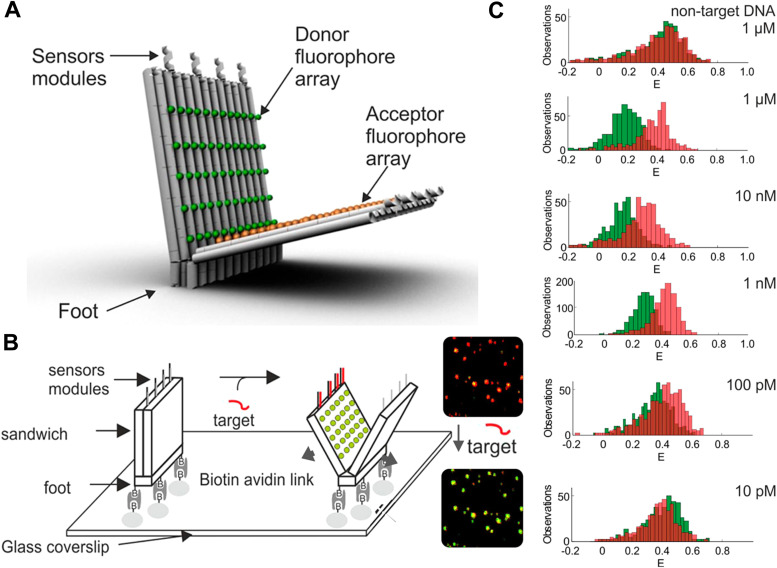
Biomarker sensing with multi-FRET DNA networks. (A) 3D model of a DNA origami beacon with a multi-dye FRET pair consisting of up to 60 Cy3 Ds (green spheres) and 60 Cy5 As (orange spheres). (B) Schematic of the attachment of the DNA origami beacon to a microscopy coverslip and beacon opening upon target binding to the sensor modules, which results in a color change (from FRET-sensitized red Cy5 to direct green Cy3 emission) in single-beacon imaging (right). (C) Image analysis with different target concentrations (red and green histograms represent FRET efficiency distributions before and after addition of DNA) resulted in a detection limit of ∼100 pM. Reproduced with permission from ref. 437 Copyright 2018 American Chemical Society.

Arguably the most common application of advanced FRET-DNA networks is in the sensing and quantification of biological targets, such as nucleic acids,^433,436,437^ proteins,^426,430,431^ or small molecules,^433^ for bioanalytical research and clinical diagnostics. In an interesting sensing derivative of a common single-DNA oligo technology, Selnihhin *et al.* translated the MB principle into multiple D–A FRET structures on a DNA origami.^437^ The DNA origami beacon consisted of two rectangular panels connected on the bottom and providing DNA sensing units on the top for target-specific opening and closing of the beacon (Fig. 67A). Each rectangular panel could host up to 60 Cy3 Ds or Cy5 As in 5 rows with 12 dyes per row and inter-dye distances of 4.3 nm (within one row) and 6.8 nm (between two rows). The four sensing units each consisted of two partially complementary ssDNA (one per rectangular panel) that hybridized and closed the origami beacon. Thus, in the closed conformation, efficient Cy3-to-Cy5 FRET could occur from the multiple Ds on one panel to the multiple As on the other panel leading to strong Cy5 sensitization when the Cy3 was excited. Specific target sequences acted to open the locked sensing units by toehold-mediated strand displacement such that the *E*_FRET_ decreased and Cy3 excitation mainly led to Cy3 emission *via* homoFRET. This sensing principle could be applied in solution to quantify targets down to 2 nM concentration after only 6 min. The concerted brightness of the multi D–A FRET system was also exploited to simultaneously image many single DNA origami beacons, which were attached *via* their biotin functionality to neutravidin-coated microscope cover slips and interrogated using conventional confocal microscopy (Fig. 67B). This approach was then used for digital target detection (counting of spots displaying different *E*_FRET_) and resulted in a detection limit down to a remarkable 100 pM (Fig. 67C).^437^ Similar to some of the other sensor configurations described above, this type of DNA origami beacon design should in principle be adaptable to many other FRET pairs and biological targets for multifunctional biosensing and bioimaging. Moreover, in the correct configuration with judicious choice of dyes, the structures could also exploit the benefits of plane-to-plane or sheet regime FRET to increase signal.

**Fig. 68:**
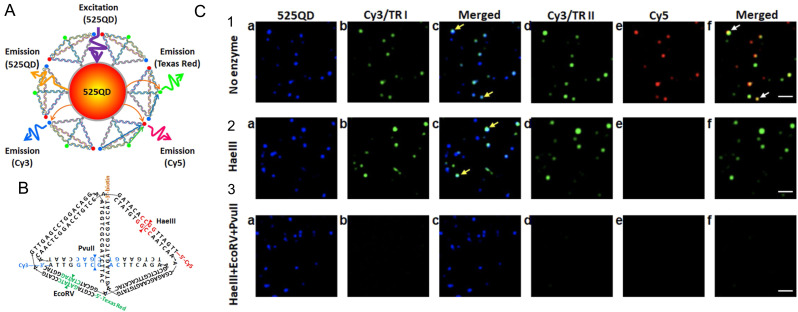
Multistep FRET network. (A) Assembly consisting of a central QD and biotin–streptavidin assembled DNA tetrahedrons with three different dyes. Excitation of the QD can excite all three dyes *via* direct QD-dye FRET or QD-dye-dye-dye FRET cascades. (B) The tetrahedron DNAs contain biotin and dye functionalities as well as specific cleavage sites for three endonucleases, which will specifically release the different dyes from the tetrahedron. (C) Fluorescence images of the 525QD-Cy3-Texas Red-Cy5 tetrahedral DNA in the (1) absence of any endonucleases and presence of (2) HaeIII and (3) HaeIII + EcoRV + PvuII. (a)–(c) Fuorescence images of the same field in the 525QD channel (500–550 nm) and the Cy3/Texas Red channel I (573–617 nm) are acquired simultaneously by using dual-view optics at an excitation wavelength of 405 nm. Then (d–f) fluorescence images in the Cy3/Texas Red channel II (565–605 nm) and Cy5 channel (672–712 nm) are acquired simultaneously. Fluorescence spots shown in the Cy3/Texas Red channel I/II (b, d, green color) result from FRET from the 525QD to Cy3/Texas Red (yellow arrows); fluorescence spots shown in the Cy5 channel (e, red color) result from FRET from the 525QD to Cy5 (white arrows). Fluorescence images with blended colors in (c) cyan indicate the colocalization of 525QD and Cy3/Texas Red; fluorescence images with blended colors in (f) yellow indicate the colocalization of Cy3/Texas Red and Cy5. The scale bar is 2 μm. Reproduced with permission from ref. 431 Copyright 2019 American Chemical Society.

To realize multiplexed biosensing on a single DNA nanostructure, Hu *et al.* attached tetrahedral DNA labeled with different dyes to a central QD.^431^ The DNA tetrahedra contained the dyes Cy3, Texas Red (TR), and Cy5 and a biotin for attachment to streptavidin-coated QDs (Fig. 68A). Up to 24 tetrahedrons with different edge lengths of 16 or 20 bp could be assembled to the central 525 nm emitting QD. Excitation of the QD led to FRET sensitization of all three dyes as well as to different FRET cascades resulting from the overall six different FRET pairs (QD-Cy3, QD-TR, QD-Cy5, Cy3-TR, Cy3–Cy5, TR-Cy5). Thus, in the fully assembled form, the FRET DNA network fluorescence spectra contained contributions from all four fluorophores. In addition to the dye and biotin functionalities, the DNA tetrahedron also contained specific substrate sequences that were amenable to cleavage by the restriction enzymes PvuII, EcoRV, and HaeIII (Fig. 68B). Each specific enzyme-substrate interaction released a specific dye from the tetrahedron (PvuII-Cy3; EcoRV-TR; HaeIII-Cy5) resulting in specific changes in the overall emission spectrum due to modification of the underlying FRET network.^431^ This principle was used for demonstrating multiplexed enzyme and inhibition assays and imaging (Fig. 68C) with only a single excitation wavelength and using only a single sensing entity (one multiplexed sensing assembly based on multiple DNA structures as attached to one QD).

The unique combination of interfacing QDs with dye-labeled DNA structures is also proving to be quite useful for creating different types of sensors. For example, Mathur and colleagues designed a modular QD-based reporter platform that is plugged in downstream of the transcription-translation (TX-TL) functionality in cell-free reactions.^445^ Addition of specific analyte to the TX-TL reaction activates a genetic circuit and results in the expression of a target enzyme whose activity signals the presence of that analyte. In the composite assemblies utilized for sensing, emissive QD Ds were paired to a dye-acceptor A on a unique DNA encoding a given restriction enzyme's cleavage site. Three sensors independently activated by the enzymes BamHI, EcoRI, and NcoI, were prototyped in mixed enzyme assays where two ultimately functioned together in a TX-TL cell-free reaction for multiplexed sensing. Specificity in these sensors arises from the expression of the different restriction enzymes in response to their target analyte, with each paired to a given QD-DNA construct that hosted only that enzyme's cleavage sequence. Thus only a specific QD-D dye-A pair is cleaved in response to analyte presence; QD amenability to multiplexed FRET allows multiple such sensors to work concurrently in the same reaction.^446,447^ Green *et al.* used a somewhat similar sensor design to assemble QD-DNA sensors for multiplexed monitoring of the CRISPR/Cas nucleases Cas13 and Cas12.^448^

**Fig. 69:**
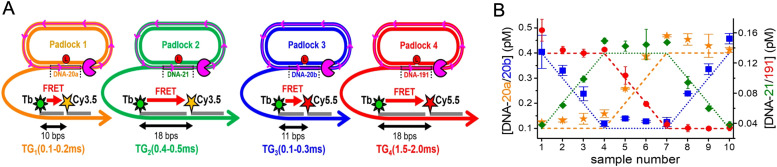
RCA-FRET DNA-scaffold biosensor. (A) Biosensor combining one Tb D with two different dye As (Cy3.5 and Cy5.5). Hybridizing the DNA probes at different distances (bps) inside the target-specific RCA products provides four distinguishable signals (two distinct TG detection windows for each of the two emission colors). (B) Multiplexed quantification of four different DNA targets at different subpicomolar concentrations in 10 different samples. Reproduced with permission from ref. 436 Copyright 2018 American Chemical Society.

In another system with strong potential for multiplexed diagnostics, Qiu *et al.* used target-primed rolling circle amplification (RCA) for combining amplified target detection with the isothermal construction of a DNA scaffold to accommodate both multicolor and multi-lifetime FRET probes.^436^ To facilitate this system, specific DNA padlock probes were first designed to hybridize to different targets. After padlock ligation, the targets served as primers for a polymerase to produce long ssDNA concatemers *via* RCA. The long repetitive sequence of the RCA product could then be used to hybridize thousands of different FRET probes at distinct positions (Fig. 69A). All FRET pairs contained the same long-lifetime Tb chelate D, which provided several narrow D emission bands over a broad spectral range and a long millisecond excited-state lifetime. Combining this Tb D with different dye A probes (Cy3.5 and Cy5.5) that hybridized at specific distances to the Tb D, the authors could tune both the FRET-sensitized A emission wavelength (color) and the FRET-sensitized fluorescence lifetime. This combination was used to produce four distinct spectrotemporal codes that could be quantified by simple time-gated (TG) intensity detection for the multiplexed analysis of four different DNA targets at subpicomolar concentrations from a single sample (Fig. 69B).^436^ Higher-order multiplexing *via* incorporation of more A dyes, more separations distances, or more lanthanide complexes, quantification of RNA biomarkers (*e.g.*, microRNA),^419^ or implementation into wash-free imaging or standard benchtop fluorescence plate readers,^449,450^ are amongst the versatile extensions of this multiplexed FRET-DNA scaffold biosensor design demonstrated to date.

**Fig. 70:**
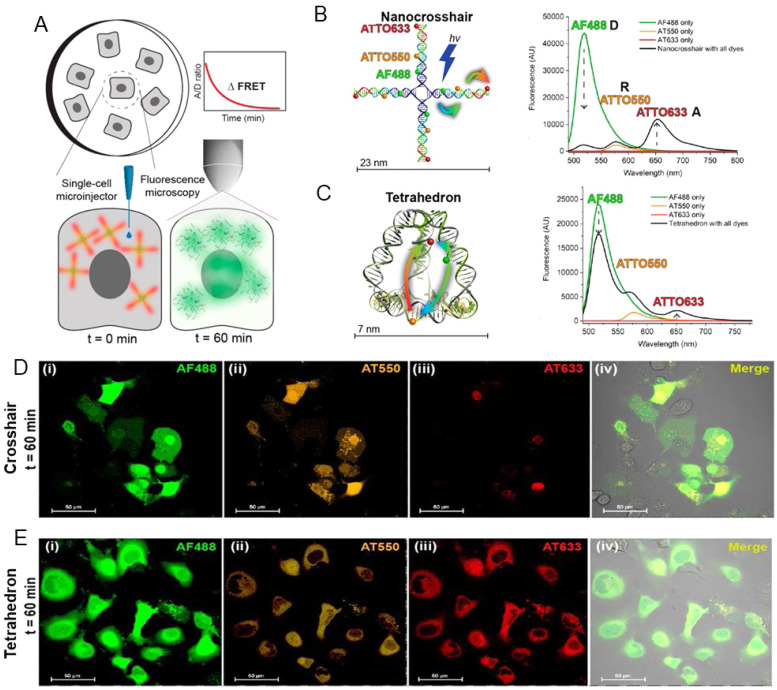
Determining the cytosolic stability of small DNA nanostructures in *cellula*. (A) Surface-adherent mammalian cells were individually injected with DNA structures into the cytosol. The fluorescence intensity and FRET between the three dyes was measured using confocal microscopy over the course of 60 min (B) DNA nanocrosshair with four copies of the 3-dye FRET network arranged linearly on each arm. Steady-state fluorescence spectra of the nanocrosshair with all dyes present *versus* individual dyes only (excitation wavelength 466 nm). (C) DNA tetrahedron with one copy of the three-dye FRET network arranged with one dye per edge. Steady-state fluorescence spectra of the tetrahedron with all dyes present *versus* individual dyes only (excitation wavelength 466 nm). Colored arrows in the schematics indicate D → Relay (R) and R → A FRET steps. Representative four-channel confocal image of COS-1 cells injected with (D) the nanocrosshair and (E) the tetrahedron after *t* = 60 min. Reproduced with permission from ref. 360 Copyright 2022 American Chemical Society.

Lastly, aside from the ET applications we focused on here, DNA and other nucleic acid structures are being actively pursued for a variety of direct biomedical applications including theranostics and for drug delivery, vaccines, and probes.^359,451,452^ Given how relatively new this technology is, almost nothing is known about the intracellular and *in vivo* stability of such DNA-based platforms and monitoring of multiFRET processes on DNA has proven quite useful to help elucidate some preliminary design rules.^360^ Determining both the short-term and long-term intracellular stability of DNA structures in cells can be significantly confounded by the complexities of using the cell's own endocytic uptake to deliver these materials. Mathur and coworkers addressed this issue *via* bypassing this endocytic uptake issue altogether and evaluated the structural stability of several common DNA structures directly in living cells. The DNA structures they utilized included a tetrahedron and a crosshair and these were labeled such that they each hosted a multistep FRET dye cascade. For experimental determinations, these were microinjected directly into the cytosol of both primary and transformed cell types. By monitoring with fluorescence microscopy, energy transfer loss was able to report on the structure's time-resolved breakdown directly *in cellula*. These results revealed a rapid degradation of the DNA crosshair within a 20 min timeframe, while that of the tetrahedron remained consistently intact for at least a 1 h post-injection time period, see the representative data shown in Fig. 70. The concomitant implementation of nuclease assays to evaluate each structure's *in vitro* stability in conjunction with an understanding of the tetrahedron's higher torsional rigidity confirmed both its higher stability and the reasons behind this. Such studies can help inform design parameters for future DNA nanostructures meant for similar applications while also being amenable to incorporating other types of non-standard nucleic acid materials including PNA and LNA. Given their non-canonical characteristics, the latter would be meant to impart more stability towards inherent nuclease activity.

## 7. Outlook

A common refrain heard about structural DNA technology and what it currently provides for assembly-wise, is that it is certainly a fascinating field of research, however, beyond its own innate abilities it has not led to anything solid in terms of products or further scientific breakthroughs. To a great extent, this is both unfair and untrue and presumably reflects impatience (from venture capitalists and research funders) with realizing the promise found within any new technology along with overlooking how much effort must be initially expended to cross the ‘Valley of Death’ and achieve full translation. Interestingly, of the start-up companies focused on DNA technology now coming on-line, a majority of their products utilize DNA and fluorescent dyes with quite a few also utilizing FRET between multiple fluorophores as part of their product lines in development.^453^ ThermoFisher now commercially offers NovaFluor dyes based on Phiton-labeling technology.^454^ This labeling system consist of multidye-labeled DNA structures that are attached to biomolecules such as antibodies, where judicious choice of dyes and their placement on the DNA allows for unique FRET-based fluorescent signatures that are tailored to avoid cross-excitation between laser lines and spectral spill over into other emission channels. Initial demonstrations of this approach utilized commercially available dye-labeled antibodies and extended their emission signatures to provide more optical channels with far less background in flow-cytometric analysis.^455^ Coming full circle, these are the very same type of benefits that the Mathies Lab sought to exploit in their ET primers almost 30 years ago.^233,234^ It is also likely that several FRET-based DNA-PAINT application kits for super-resolution microscopical imaging will soon become commercially available.^427,456,457^ In comparison to the entirety of DNA nanotechology in all its different manifestations, the subarea that combines nucleic acid scaffolds with fluorophores and ET represents just a relatively small contribution. However, the above examples, and others not mentioned here, represent the critical start of this technology's translation to wider use and it is well worth noting that despite their small share of the total they are at the forefront.

Changes are also expected in how DNA itself is synthesized. Several companies are nearing delivery of enzyme-based DNA synthesis technologies and making these commercially available to licensed vendors or acting as providers themselves.^458,459^ This will be a welcome addition since it bypasses the initial costly phosphoramidite synthesis and will be a far greener approach in the long run. It is, however, not currently clear how these technologies can implement insertion of modified and much bulkier dye-labeled bases into nascent oligonucleotides. The same applies to non-natural bases, which are increasingly being used in drug development. In line with this, new DNA modifications are constantly being developed and added to the library of commercially available products as they provide different functional groups and allow new types of modification chemistry to be used.^51^ This suggests that the traditional phosphoramidite chemistry is still going to be part of the commercial landscape for quite some time.

From a FRET-focused perspective, the development of these composite materials is proceeding in a far more dynamic manner. The unique combination of access to any designer nanoscale architecture in combination with all the processes and properties inherent to FRET is making these hybrid materials useful across a wide swath of research applications including especially as sensors and probes. Indeed, many of the examples described in the preceding section epitomize such applications where, for example, dye-labeled DNA structures either on their own or as interfaced with NPs such as QDs are providing for a variety of targeted sensors for monitoring force on the nanoscale,^432^ pH,^429^ voltage changes,^435^ multiplexed enzyme activity,^431,445,448^ along with providing for multiplexed diagnostics, and biomarker detection.^419,436,460^ The always growing library of available fluorescent materials, ranging from new dye families to noble metal clusters and NPs manifesting unique quantum confined or other complex photophysical properties such as upconversion will also contribute to new FRET-based applications and probes that utilize DNA scaffolds.^4,5,117,461,462^ Interestingly, DNA scaffolding often proves itself critically useful in helping to confirm that new fluorescent materials actually engage in FRET by providing a defined nanoscale ruler to do the requisite distance-dependent ET experiments.^248,463–467^

Complex FRET-based DNA structures also have strong inherent utility in other DNA technology-based applications including that of theranostic nanodevices. Theranostics refers to the integration of diagnosis and therapy in a single platform using nanomaterials and is meant here to describe futuristic nanomachines that function inside the body in a preventative or curative manner.^468–470^ Obviously, the versatility of DNA technology allows for both the assembly of the requisite nanoscale chassis in any desired shape along with the ability to display the active drug, targeting moiety, sensors, biologicals such as proteins, and other desirable cargo.^471,472^ The combination of multiple fluorophores and FRET on these structures can allow for LH to enable imaging as well as photodynamic therapy along with signaling of binding events by a concomitant reconfiguration of the dyes and their FRET signature. Judicious choice of dyes and their FRET processes can help these structures also function as deep tissue probes that harvest light and concentrate/focus it to a final A for imaging. Coupling luciferase or similar light generating enzymes to these structures can allow them to generate their own fluorescent excitation *in situ* with addition of appropriate substrate. Indeed, this very approach has been previously used to create self-illuminating QD conjugates for *in vivo* imaging in the tissue of small animal models.^473^ Recent demonstrations of DNA origami delivery to cells where the origami scaffold sequence encoded a gene that was subsequently expressed inside the cells suggests that the first steps towards the holistic application of these materials are already underway.^474,475^ Other embodiments could also cohost and power photosynthetic reaction centers or other photochemical reactants. Although this may sound like the stuff of science fiction, it is worth noting that all the components already exist, are functional, are either bioactive or biocompatible, and that the concept being described is just a cumulative integration of these components and their function. The ability to self-assemble many different variants of a given structure in a side-by-side format in great numbers will certainly allow for selection schemes that look to optimize some functionality or find other properties/activities of interest.

The outlook for applications for molecular aggregates formed by DNA scaffolds and templates are promising and broad. These applications include light harvesting,^187,189^ organic optoelectronics,^192^ and quantum information systems that employ quantum entanglement such as quantum computing.^199,202–204,206,207,476^ As described in detail, fundamental to these applications is exciton delocalization as mediated by *J*_*m*,*n*_, which is one of the key parameters in the molecular or Frenkel exciton Hamiltonian (eqn (14)). Since exciton delocalization involves delocalized wave functions that extend across a number of dyes in an aggregate and are easily collapsible by environmental factors, as opposed to localized on a single dye, DNA-templated dye aggregates could serve as sensitive diagnostic sensors for biological and biomedical applications. Overall, excitonic quantum computing may be the most broadly impactful of the applications and so we will briefly delve into this area to describe the key roadblocks that must be overcome.

Excitonic quantum computing has not been realized and, to date, only a limited amount of theoretical work has been published to explore its viability. Most of the theory has been centered on creating quantum gates employing exciton delocalization (*i.e.*, *J*_*m*,*n*_, so single exciton states). They show that the unitary transformations of quantum gates and small quantum computing networks can be implemented in dye aggregates by employing the *J*_*m*,*n*_ coupling.^203,204,206,476^ Using *J*_*m*,*n*_ to explore the viability relative to quantum computing seems reasonable because *J*_*m*,*n*_ is fundamental to the Frenkel Hamiltonian, is readily obtained both theoretically and experimentally, and its energy can be compared to room temperature as a first order metric to assess how long before the system decoheres (discussed later). However, excitonic quantum gates that only use *J*_*m*,*n*_ to perform operations have several limitations. Willard and coworkers astutely point out that one limitation to using a single exciton particle state to encode coherences and multi-particle entanglement is the system size-scaling problem.^204^ That is, their gates cannot exploit quantum parallelism; the ramification is that scalable quantum computing cannot be realized. To reduce or eliminate the scaling problem and much more efficiently encode coherence and multi-particle entanglement, exciton–exciton interactions can be employed. The theory of exciton–exciton interaction is certainly more complicated but potentially solves the scaling problem because it enables quantum parallelism and thus supports scalable quantum computing. Yurke and coworkers provided a methodology that examines both *J*_*m*,*n*_ interactions as well as exciton–exciton interactions described by the rarely studied *K*_*m,n*_ that yields correlated exciton entangled states and promotes scalable quantum computing.^206,207^ Excitonic quantum gate schemes devised by Yurke *et al.* show that dyes must have specifically oriented *J*_*m*,*n*_ (*i.e.*, *μ*_*m*_) and *K*_*m,n*_ (*i.e.*, Δ*d*_*m*_) with respect to each other.

**Fig. 71:**
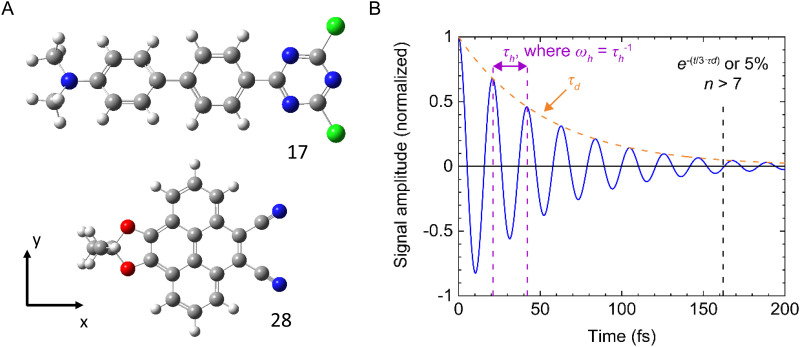
Examining theoretical dye structures. (A) Structure diagram for dye 17 and 28 from the work of Jacquemin.^477^ Light grey – hydrogen, dark grey – carbon, dark blue – nitrogen, green – chlorine, red – oxygen. Calculations on these structures were performed to obtain the magnitude and direction of Δ*d*_*m*_ and *μ*_*m*_. (B) Signal amplitude schematic of a dimer with hopping time, *τ*_h_ (“beat time” between the two exciton states) & dephasing time *τ*_d_. *J*_*m*,*n*_ of 98 meV (790 cm^−1^) gives *ω*_h_ ∼ 47 THz^222^ (eqn (23)) or *τ*_h_ of 21 fs. A *τ*_d_ ∼ 54 fs is obtain from the reduction of homogeneous broadening of Δ*E*_homog_ ∼ 18 THz observed in a DNA template rotaxane SQ dimer.^403^ Assuming 5% of measurable original signal amplitude (*i.e.*, e^(−*t*/[3×*τ*_d_])^) gives 3*τ*_d_ or 162 fs (time to be able to perform measureable calculations) & *n* > 7 measurable gate operations. *T* = 295 K.

The question is, do dyes exist that have specifically oriented *J*_*m*,*n*_ and *K*_*m,n*_ with respect to each other that meet the criteria of Yurke *et al.*^206,207^ or can they be realized, either theoretically or experimentally synthesized? To the best of our knowledge, no dyes have been experimentally realized that can meet these criteria. However, theoretical work on certain dyes suggests that it is plausible that such dyes can be created synthetically. Jacquemin calculated Δ*d*_*m*_ for several dyes that would meet the criteria of Yurke *et al.*; however, values of *μ*_*m*_ were not calculated.^477^ Jacquemin calculated a Δ*d*_*m*_ of 16.07 D for dye 17 (Fig. 71A). For the same dye, we performed similar calculations to determine both the magnitude and direction of Δ*d*_*m*_ and *μ*_*m*_. The calculations resulted in a Δ*d*_*m*_ of 16.15 D and a *μ*_*m*_ of 8.47 D with both along the -*x* direction (directions shown in Fig. 71A). The similar values of Δ*d*_*m*_ provides a degree of validation between the two approaches. Our calculations showed one dye, dye 28, for which Δ*d*_*m*_ and *μ*_*m*_ were perpendicular with values of 8.27 D (-*x* direction) and 3.65 D (*y* direction), respectively. Our value of Δ*d*_*m*_ was very similar to the Δ*d*_*m*_ of Jacquemin (7.80 D). The fact that Jacquemin presented two dyes for which Δ*d*_*m*_ and *μ*_*m*_ were either perpendicular or parallel indicates that the orientation and magnitude of Δ*d*_*m*_ and *μ*_*m*_ are tunable. Indeed, Ketteridge *et al.* showed that the orientation of both *μ*_*m*_ and Δ*d*_*m*_, thus *J*_*m*,*n*_ and *K*_*m,n*_, in SQ dyes can be tuned with the proper selection and location of dye substituents.^478^ Ideally, one would like *J*_*m*,*n*_ and *K*_*m,n*_ to be very large, say at least five times that of the thermal energy at room temperature (*k*_B_*T*_rm_ ∼ 26 meV). In an earlier section, we described several publications highlighting the tuning of *J*_*m*,*n*_ with substituents and hydrophobicity and steric hindrance,^65,378^ as well as several computational efforts that examine tuning *J*_*m*,*n*_ by altering substituents on dyes.^479,480^ More recently, guided by DFT and TD-DFT calculated Δ*d*_*m*_, Wright and coworkers custom-synthesized and characterized four custom SQ dyes.^481^ Each dye was synthesized with different types of substituents such that the Δ*d*_*m*_ of each dye differed. However, because actually measuring Δ*d*_*m*_ or *K*_*m,n*_ remains a challenge, the study focused on the time-dependent nature of delocalized excitons. Thus, at this time, dyes can be synthesized with a *J*_*m*,*n*_ or a specified Δ*d*_*m*_, but not with both. The growth in this direction is, however, promising and it will be exciting to see the progress in this area.

Let us assume that dyes in the future will be synthesized with specifically oriented *J*_*m*,*n*_ and *K*_*m,n*_ that meet Yurke's criteria. A major obstacle to constructing an excitonic quantum gate configured with such dyes is for that gate to operate at room temperature for a time period long enough to perform meaningful gate operations before the system dephases. That time period is known as the dephasing time, *τ*_d_. One would also need to know the time to perform one calculation and the number of calculations that can then be performed before the system dephases. For dye aggregates, to first order, we consider the time to perform one calculation as the time an exciton can be coherently exchanged between two dyes (*i.e.*, hop from one dye to a neighboring dye) in the aggregate. This hopping time we defined as *τ*_h_. The number of times, *n*, an exciton can be coherently exchanged between two dyes can be considered, to first order, as the number of calculations that can be performed before the system dephases. *n* can be described with both *τ*_d_ and *τ*_h_ in the following manner:

23 *n* = 2·*J*_*m*,*n*_·*c*·*τ*_d_ = *ω*_h_·*τ*_d_ = *τ*_d_/*τ*_h_

where *c* is the speed of light, *J*_*m*,*n*_ is expressed in wave numbers, and *ω*_*h*_ is the hopping frequency and is the inverse of *τ*_h_. From eqn (23), for a given *τ*_d_, we see that as *τ*_h_ decreases, *n* increases. We also see that *τ*_h_ is inversely proportional to *J*_*m*,*n*_, so the larger *J*_*m*,*n*_ is, the shorter is *τ*_h_. In Fig. 71B, both *τ*_d_ and *τ*_h_ are nicely visualized. The figure was generated using data from the work of Mass *et al.*^222^ and Barclay *et al.*^403^ that provided a *J*_*m*,*n*_ of 98 meV, a *τ*_d_ of 54 fs and a of *τ*_h_ 21 fs. The calculation assumes that 5% of the original signal amplitude is measurable which equates to 3*τ*_d_ or 162 fs and gives *n* a value of greater than 7. That is, to first order, there is time to perform at least 7 measurable gate operations before the system dephases. The ongoing work described in this review certainly demonstrates that values of *J*_*m*,*n*_, *τ*_d_, and *τ*_h_ have or likely will improve. While the obstacles are considerable (we have only described several) for using dye aggregates as a means to construct excitonic quantum gates, the outlook towards realizing this goal is promising.

In summary, given the pull coming from biomedical, diagnostics, and basic research along with the interest in accessing new applications like room-temperature quantum information processing, there is certainly a bright future for research and development efforts centered on the myriad fruitful combinations of nucleic acids, fluorophores, and energy/exciton transfer.

## Abbreviations

1/2/3D: One/two/three dimensional
A: Acceptor
A: Adenine base
AE: Antenna effect
AE_tot_: Total antenna effect
AFM: Atomic force microscopy
AG: Antenna gain
APPV: Poly[5-methoxy-2-(3-sulfopropoxy)-1,4-phenylenevinylene] polymer
BNA: Bridged nucleic acid
bp: Base pairs
BRET: Bioluminescent resonance energy transfer
C: Cytosine
CD: Circular dichroism
CFETs: Combinatorial FRET tags
C_*n*_: Carbon linker of n length
CPG: Controlled pore glass
CRISPR/Cas: Clustered regularly interspaced short palindromic repeats/CRISPR-associated
D: Donor
DFT: Density functional theory
DNA: Deoxyribonucleic acid
DNA-PAINT: DNA points accumulation for imaging in nanoscale topography
dNTPs: Deoxynucleoside triphosphates
DR: Dual rail
ds: Double-stranded
DX DNA: Cross-over
*E*_ae_
: Anywhere-to-end ET efficiency
*E*_ee_
: End-to-end ET efficiency
*E*_FRET_
: FRET efficiency
ET: Energy-transfer
→: Energy transfer step
EUA: Emergency use authorization
FAM: Fluorescein
FDA: U.S. Food and Drug Administration
ΔΔ*G*: Free energy barriers
FRET: Förster resonance energy transfer (FRET)
G: Guanine
GAFF: Generalized Amber force field
HPLC: High performance liquid chromatography
HJ: DNA Holliday junction
HOMO: Highest occupied molecular orbital
ISMB: In-stem molecular beacon
kDa: Kilodaltons
*κ*
: Dipole orientation factor
KRM: Kühn, Renger, and May
LBL: Layer-by-layer
LHC: Light harvesting cascade
LNA: Locked nucleic acid
Luc: Luciferase
LUMO: Lowest unoccupied molecular orbital
MB: Molecular beacon
MD: Molecular dynamics
mer: Oligomer
MGC: Malachite green chloride
MPW: Molecular photonic wires
NH_4_OH: Ammonium hydroxide
NHS: N-hydroxysuccinimide
NP: Nanoparticle
Ni^2+^-NTA: Nickel nitrilotriacetic acid
ODN: Oligodeoxynucleotides
OFRR: Optofluidic ring resonator
PDIs: π-conjugated perylene diimides
PA: Pyrrole-imidazole polyamide
PB: Pacific blue
pcPNA: Pseudocomplementary peptide nucleic acids
PEG: Polyethylene glycol
PNA: Peptide nucleic acid
PUF: Physically unclonable function
PVA: Polyvinyl alcohol
*Q*
: Quantum yield
QD: Quantum dot
*R*_0_
: Förster distance
RC: Reaction center
RNA: Ribonucleic acid
RT-qPCR: Reverse transcription quantitative PCR
SARS-CoV-2: Coronavirus that causes COVID-19
SAS: Species-associated spectra
smFRET: Single molecule FRET
SMFS: Single-molecule fluorescence spectroscopy
SQ: Squaraine
SR: Split rail
ss: Single-stranded
*τ*
: Excited state lifetime
2D: Two-dimensional
T: Thymine
TAMRA: Tetramethylrhodamine
TCA: Trichloroacetic acid
TEF: Terminal enhancement factor
TD-DFT: Time-dependent density functional theory
TFP: Teal fluorescent protein
TG: Time-gated
TIP3P: Transferable intermolecular potential 3P
TIRF: Total internal reflection fluorescence
TEM: Transmission electron microscopy
TIRF: Total internal reflection fluorescence
TO: Thiazole orange
TOTO: Thiazole orange homodimer
TR: Texas red dye
*Ψ*
: Transmembrane potential
Δ*Ψ*: Change in transmembrane potential
TX-TL: Transcription translation
UV-vis: Ultraviolet-visible
WGM: Whispering galley mode
XRD: X-Ray diffraction
YO: Oxazole yellow
YOYO-1: Oxazole yellow homodimer
YO-PRO: 1-4-[(3-methyl-2(3H)-benzoxazolylidene)methyl]-1-[3-(trimethylammonio)propyl]
ZnTPEP: Zn-tetra-(phenylethynyl) porphyrin

## Conflicts of interest

There are no conflicts to declare.

## Note added in proof

The authors note the publication of two relevant publications during the time this manuscript was in review. Prof. Schlau-Cohen and colleagues at MIT published an account covering primarily their efforts to engineer exciton dynamics on DNA scaffolds.^482^ Pramanik and Mukherjee published a review on bio-templated energy transfer system for constructing artificial light-harvesting antennae, white light generation, and photonic nanowires.^483^

## Supplementary Material

CS-052-D0CS00936A-s001
