# Supplementary material for: Pursuing excitonic energy transfer with programmable DNA-based optical breadboards
Source: Chem Soc Rev. 2023 Oct 24;52(22):7848–948. doi: 10.1039/d0cs00936a (PMC10642627; doi:10.1039/d0cs00936a)
Supplement: CS-052-D0CS00936A-s001 [file CS-052-D0CS00936A-s001.pdf]

Electronic Supplementary Material (ESI) for Chemical Society Reviews.
This journal is © The Royal Society of Chemistry 2023

SUPPORTING INFORMATION

Pursuing Excitonic Energy Transfer with Programmable DNA-based Optical Breadboards

Divita Mathur[1,8], Sebastián A. Díaz[2,8], Niko Hildebrandt[4,5], Ryan D. Pensack[6], Bernard Yurke[6], Austin Biaggne[6], Lan Li[6,7], Joseph S. Melinger[3], Mario G. Ancona[3,8], William B. Knowlton[6]*, and Igor L. Medintz[2]*

[1]Department of Chemistry
Case Western Reserve University
Cleveland OH 44106 USA

[2]Center for Bio/Molecular Science and Engineering, Code 6900
[3]Electronics Science and Technology Division, Code 6800
U.S. Naval Research Laboratory
Washington, DC 20375 USA

[4]Department of Chemistry
Seoul National University
Seoul 08826, South Korea

[5]Laboratoire COBRA
Université de Rouen Normandie
CNRS, INSA, Normandie Université,
76000 Rouen, France

[6]Micron School of Materials Science & Engineering
Boise State University,
Boise, ID 83725 USA

[7]Center for Advanced Energy Studies
Idaho Falls, ID 83401, USA

[8]Department of Electrical and Computer Engineering
Florida State University
Tallahassee, FL 32310, USA

[8]Equal contributions

*Corresponding Authors: bknowlton@boisestate.edu
igor.medintz@nrl.navy.mil

Keywords: DNA, Nanotechnology, Energy Transfer, Dye, FRET, Dipole, Donor, Acceptor, Exciton, Delocalization, Coherence, Photonic Wire

Supporting Table 1: Phosphoramidites available for in-synthesis modification of oligonucleotides. Reactive groups that are available as 3’ modification are usually present on the CPG whereas 5’, internal (int), and modified bases (dA, dC, dG, dT, dU) are available as phosphoramidites. *Commercially available. C_x - carbon linker of x length. TEG - triethylene glycol. Glen - Glen Research, Inc.

Reactive group/handle	Linkers	Purpose	Availability	Ref
Biotin*	C_6, TEG	Affinity for streptavidin	5' int 3' dT	1
Thiol*	C_3, C_6	Gold, maleimide	5' 3'	2
DBCO*	TEG	Azide click chemistry	5’ int dT	3
Phosphate*	None	Ligate another DNA	5' 3’	Glen
Amino*	C_6,C_{12}	Amide click chemistry	5' int dT	Glen
Alkyne*	C_8	Azide click chemistry	5’ int 3’	Glen
Maleimide*	C_2	Thiol click chemistry	5'	4
Methacrylate*	C_6	React with thiol, crosslink (expansion microscopy)	5'	5
Dendrimers*	C_2-C_4	DNA branching	5'	6
Aldehyde*	Backbone	Amine click chemistry	5'	7
Triazole	Ethynyl spacer	Reduce anionic charge, DNA and/or LNA ligation	Int	8-10
Cholesteryl*	TEG	Enhance cell permeability of DNA (10908328 ?)	5' int 3'	Glen
Ferrocene*	Nucleotide	Generate probes for electrochemical detection	dT	11, 12
Azobenzene*	D-threoninol	Photo-reactive modification	int	13
Triazine	Nucleotide	Reactive to 1-methylcyclopropenes	dU, dA	14
Cyclopropene	Nucleotide	Reactive to triazine	dU, dA	14
Ruthenium bipyridine	Nucleotide	Metallization, fluorescence	Int base	15-18

Terpyridine, Dipicolylamine* phenanthroline, dithioethers	Nucleotide	Metal coordination (Zn, Ni, Cu, Ag, Pb, etc) in DNA and LNA	5' int dG	19-23
Porphyrin	Backbone	Electron transfer, DNA aggregation	Int dC	24, 25
Galactose/carbohydrates	Nucleotide	Site-specific carbohydrate bioconjugation	Int dU	26, 27
Iodine/Bromine*	Nucleotide	Carbohydrate conjugation, photo cross-linking	dC dU	Glen
**Dyes**				
Cyanine (Cy3, Cy3.5, Cy5, Cy5.5)*	C_3/C_6	Attachment of Cy dye to DNA backbone via single or double phosphodiester bond	5’ int 3’	
Methylene Blue*	C_2/C_3	Attachment of MB to DNA via single or double phosphodiester bond	5’ Int 3’	
Black Hole, Blackberry™, and Dabcyl Quenchers*	C_2/C_6	Attachment to DNA via single phosphodiester terminal bond or via T nucleotide	5’ dT 3’	
Fluorescein*	C_6	Attachment to DNA via single/double phosphoramidite and T nucleotide	5’ Int dT 3’	
Rhodamine*	C_6	Attachment to DNA via dT or 3’ end single phosphodiester bond	dT 3’	
ATTO/Alexa Fluor™ dyes	C_6/C_{12}	Attachment to DNA via single phosphodiester bond	5’ 3’	

Supporting References:

1. R. T. Pon, *Tetrahedron Lett*, 1991, **32**, 1715-1718.
2. B. A. Connolly and P. Rider, *Nucleic Acids Res*, 1985, **13**, 4485-4502.
3. P. van Delft, N. J. Meeuwenoord, S. Hoogendoorn, J. Dinkelaar, H. S. Overkleeft, G. A. van der Marel and D. V. Filippov, *Org Lett*, 2010, **12**, 5486-5489.
4. A. Sanchez, E. Pedroso and A. Grandas, *Org Lett*, 2011, **13**, 4364-4367.
5. F. N. Rehman, M. Audeh, E. S. Abrams, P. W. Hammond, M. Kenney and T. C. Boles, *Nucleic Acids Res*, 1999, **27**, 649-655.
6. M. S. Shchepinov, K. U. Mir, J. K. Elder, M. D. Frank-Kamenetskii and E. M. Southern, *Nucleic Acids Res*, 1999, **27**, 3035-3041.
7. M. A. Podyminogin, E. A. Lukhtanov and M. W. Reed, *Nucleic Acids Res*, 2001, **29**, 5090-5098.
8. P. Kumar, L. Truong, Y. R. Baker, A. H. El-Sagheer and T. Brown, *ACS Omega*, 2018, **3**, 6976-6987.
9. I. Manuguerra, S. Croce, A. H. El-Sagheer, A. Krissanaprasit, T. Brown, K. V. Gothelf and A. Manetto, *Chem Commun*, 2018, **54**, 4529-4532.
10. A. H. El-Sagheer and T. Brown, *Acc Chem Res*, 2012, **45**, 1258-1267.
11. A. E. Navarro, N. Spinelli, C. Moustrou, C. Chaix, B. Mandrand and H. Brisset, *Nucleic Acids Res*, 2004, **32**, 5310-5319.
12. H. Brisset, A. E. Navarro, N. Spinelli, C. Chaix and B. Mandrand, *Biotechnol J*, 2006, **1**, 95-98.
13. H. Asanuma, T. Takarada, T. Yoshida, D. Tamaru, X. Liang and M. Komiyama, *Angew Chem Int Ed*, 2001, **40**, 2671-2673.
14. U. Reisacher, D. Ploschik, F. Ronicke, G. B. Cserep, P. Kele and H. A. Wagenknecht, *Chem Sci*, 2019, **10**, 4032-4037.
15. F. D. Lewis, S. A. Helvoigt and R. L. Letsinger, *Chem Commun*, 1999, DOI: 10.1039/A808491B, 327-328.
16. S. I. Khan, A. E. Beilstein, M. Sykora, G. D. Smith, X. Hu and M. W. Grinstaff, *Inorganic Chem*, 1999, **38**, 3922-3925.
17. D. J. Hurley and Y. Tor, *J Am Chem Soc*, 2002, **124**, 13231-13241.
18. D. J. Hurley and Y. Tor, *J Am Chem Soc*, 1998, **120**, 2194-2195.
19. J. S. Choi, C. W. Kang, K. Jung, J. W. Yang, Y.-G. Kim and H. Han, *J Am Chem Soc*, 2004, **126**, 8606-8607.
20. M. M. Knagge and J. J. Wilker, *Chem Commun,* 2007, DOI: 10.1039/b704741j, 3356-3358.
21. K. Wiederholt and L. W. McLaughlin, *Nucleic Acids Res*, 1999, **27**, 2487-2493.
22. A. Galeone, L. Mayol, G. Oliviero, D. Rigano and M. Varra, *Bioorganic & Medicinal Chem Lett*, 2001, **11**, 383-386.
23. M. Kalek, A. S. Madsen and J. Wengel, *J Am Chem Soc*, 2007, **129**, 9392-9400.
24. H. Morales-Rojas and E. T. Kool, *Organic Letters*, 2002, **4**, 4377-4380.
25. K. Berlin, R. K. Jain, M. D. Simon and C. Richert, *J Organic Chem*, 1998, **63**, 1527-1535.
26. T. L. Sheppard, C. H. Wong and G. F. Joyce, *Angew Chem Int Ed*, 2000, **39**, 3660-3663.
27. K. Matsuura, M. Hibino, M. Kataoka, Y. Hayakawa and K. Kobayashi, *Tetrahedron Lett*, 2000, **41**, 7529-7533.
